# Unveiling the intricacy of gapmer oligonucleotides through advanced tandem mass spectrometry approaches and scan accumulation for 2DMS†

**DOI:** 10.1039/d4an00484a

**Published:** 2024-07-18

**Authors:** Mohammed Rahman, Bryan P. Marzullo, Pui Yiu Lam, Mark P. Barrow, Stephen W. Holman, Andrew D. Ray, Peter B. O'Connor

**Affiliations:** a Department of Chemistry, University of Warwick Coventry CV4 7AL UK p.oconnor@warwick.ac.uk; b Department of Physics, University of Warwick Coventry CV4 7AL UK; c Chemical Development, Pharmaceutical Technology & Development, Operations, AstraZeneca AstraZeneca SK10 2NA UK; d New Modalities & Parental Development, Pharmaceutical Technology & Development, Operations, AstraZeneca Macclesfield SK10 2NA UK

## Abstract

Antisense oligonucleotides (ASOs) are crucial for biological applications as they bind to complementary RNA sequences, modulating protein expression. ASOs undergo synthetic modifications like phosphorothioate (PS) backbone and locked nucleic acid (LNA) to enhance stability and specificity. Tandem mass spectrometry (MS) techniques were employed to study gapmer ASOs, which feature a DNA chain within RNA segments at both termini, revealing enhanced cleavages with ultraviolet photodissociation (UVPD) and complementary fragment ions from collision-induced dissociation (CID) and electron detachment dissociation (EDD). 2DMS, a data-independent analysis technique, allowed for comprehensive coverage and identification of shared fragments across multiple precursor ions. EDD fragmentation efficiency correlated with precursor ion charge states, with higher charges facilitating dissociation due to intramolecular repulsions. An electron energy of 22.8 eV enabled electron capture and radical-based cleavage. Accumulating multiple scans and generating average spectra improved signal intensity, aided by denoising algorithms. Data analysis utilised a custom Python script capable of handling modifications and generating unique mass lists.

## Abstract 2

Multimodal tandem mass spectrometry sequencing of oligonucleotides using CID, EDD, IRMPD, and UVPD as fragmentation methods, and using 2-dimensional mass spectrometry with signal averaging to differentiate fragments from closely spaced precursors.

## Graphical abstract

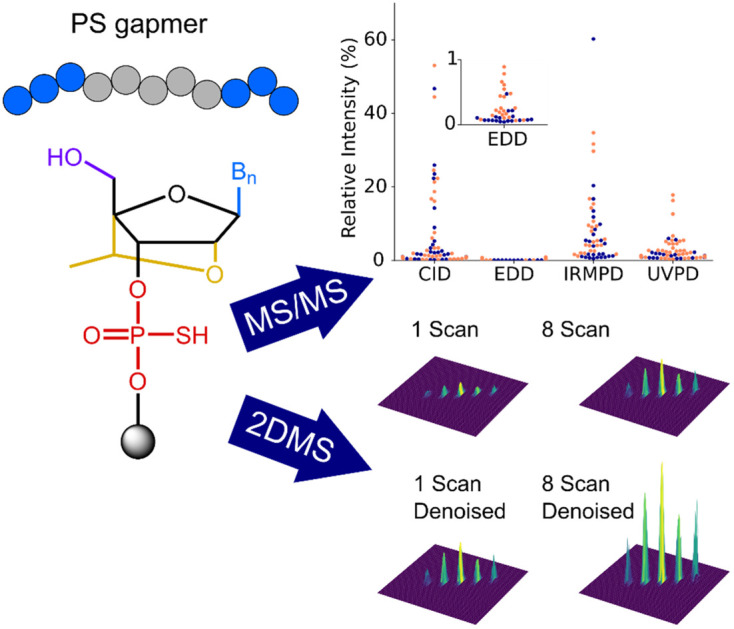

## 1. Introduction

Antisense oligonucleotides (ASOs) are short, synthetic nucleic acid molecules designed to selectively bind to RNA or DNA sequences, modulating gene expression and protein synthesis.^1–3^ Binding occurs through complementary base pairing as described by Watson and Crick,^4^ forming a duplex that can inhibit protein translation or the modulation of alternative splicing.^5–9^ ASOs offer several advantages over traditional small molecule drugs such as high target specificity, low off-target effects and the ability to modulate targets previously considered “undruggable”.^3,9^ Despite these advantages, some challenges are associated with ASOs, including the need for an effective delivery system, stability, and potential toxic or immune reactions.^5,8,9^ Therefore, there have been many modifications to ASOs to mitigate these challenges.^8,9^

ASOs can be chemically modified to improve their stability, specificity and pharmacokinetic properties.^3,9,10^ Firstly, a phosphorothioate (PS) backbone, where a non-bridging oxygen atom in the phosphodiester linkage is replaced by a sulfur atom, increases the stability of the ASO by increasing its resistance to nuclease degradation.^8–13^ Additionally, the incorporation of modifications at the 2′ carbon of the ribose sugar, such as 2′-*O*-methoxyl (OME), or locked nucleic acid (LNA) and constrained ethyl (cEt), results in the formation of a rigid structure.^13^ The presence of a bridging molecule (methylene for LNA and ethyl for cEt) that connects the 2′ oxygen of 4′ carbon, not only enhances the stability of the ASO but also helps to mitigate its immunostimulatory effects.^3,12–17^ Furthermore, a combination of modified and unmodified nucleotides,^18^ otherwise known as gapmer ASOs,^13,19–24^ combines the stability of modifications such as PS, OME or LNA, with the high target affinity of the unmodified chain.^3^ Current sequencing of oligonucleotides can be achieved using fluorescent probes,^25,26^ polymerase chain reaction,^27^ and mass spectrometry (MS).^28,29^

MS of oligonucleotides can follow two approaches, top-down or bottom-up.^30–42^ Bottom-up approaches involves the enzymatic digestion, typically using a ribonuclease or deoxyribonuclease, of the primary sequence, where the fragments analysed by mass spectrometry are less complex than the intact molecule.^30,31,43^ Nevertheless, the bottom-up approach may exhibit a bias towards the particular enzyme employed for digestion and can be prone to incomplete digestion, which highlights the need for a top-down approach.^32^ Given the limited number of nucleobases, enzymatic digestion can lead to the generation of identical fragments within the sequence, which adds complexity to the analysis. Top-down approaches offer a solution to the challenges associated with enzymatic digestion by providing primary sequence information without the need for digestion.^38^ Use of high-end instrumentation such as Fourier-transform-ion cyclotron resonance-mass spectrometry (FT-ICR-MS) allows for the analysis of complex mixtures using the top-down approach, where mass resolution, sensitivity, and mass accuracy are a requirement as demonstrated in previous studies.^36,37^ High mass resolution of FT-ICR-MS enables the determination of fine isotopic patterns, including bromine and sulfur atoms,^44^ which were previously used to study the uptake of ASOs in cells and tissues by nano secondary ionisation imaging.^45^

**Scheme 1:**
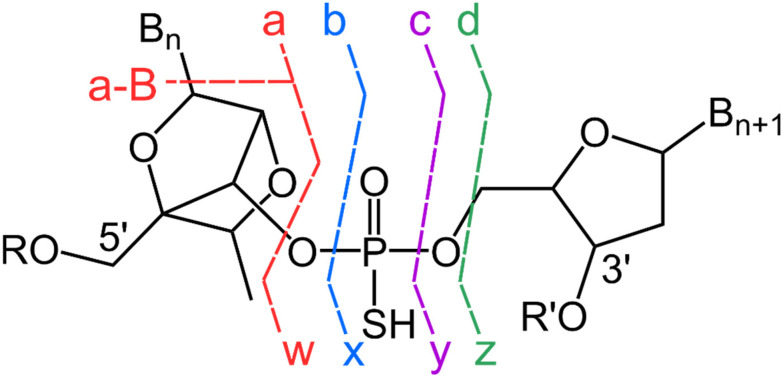
Nomenclature for oligonucleotide fragmentation based on McLuckey cleavages with a constrained ethyl (cEt) modification displayed on the 5′-end.^17,51^ Full atomic numbering is shown in ESI Fig. S1.†

FT-ICR-MS can be used in combination with a variety of fragmentation techniques such as collision-induced dissociation (CID),^38,46–52^ infrared multiphoton dissociation (IRMPD),^52–55^ ultraviolet photodissociation (UVPD),^52,56–59^ and electron-based fragmentation (ExD),^47,60–64^ where electron detachment dissociation (EDD)^49,65–69^ is used for multi-negatively charged species. Different fragmentation methods provide complementary information about oligonucleotide sequence, allowing for more confident structural characterisation. Fragmentation patterns of oligonucleotides are summarised by McLuckey cleavages (Scheme 1), where the backbone bonds of the nucleotide units are cleaved, leading to the generation of fragment ions that provide structural information about the sequence and modifications of the oligonucleotide.^51^ For example, CID and EDD predominantly leads to *a-B* (where *B* refers to a nucleobase) and the complementary *w* ions,^48,66^ whereas UVPD can display the full McLuckey cleavages albeit in low abundance,^70^Scheme 1.^51^

**Scheme 2:**
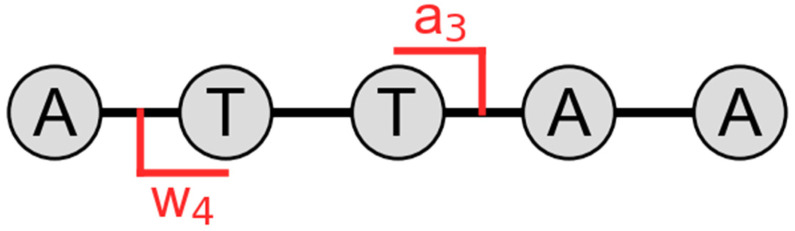
Labelling of internal fragments used in this study, adapted from Loo and co-workers,^71^ where the fragment ion is within the middle of the chain (a_3_w_4_).

Base loss occurs due to the cleavage of the glycosidic bond between the sugar and the base, which is typically observed as *a-B* fragments by CID.^50,51^ However, it is important to note that gas-phase rearrangements can lead to formation of *y-B* ions,^57^ whereas modifications such as methylphosphonate can limit the extent of base loss.^48^ For a highly charged precursor ion, the loss of a charged nucleobase was found to be in the order of A^−^ > T^−^ > G^−^ > C^−^,^51^ which suggests that the charged base loss (CBL) is driven by release of coulombic strain.^72^ Additionally, atypical fragmentation through formation of a cyclic intermediate resulting in internal loss of PO_3_^−^, and NCO^−^ from the base has been observed.^72^ Internal fragments, which occur due to cleavage of the backbone and contain neither termini (Scheme 2), was shown by Loo and co-workers to enhance sequence coverage by 15–20% on proteins,^73^ similarly a combination of internal fragments and termini fragments results in greater sequence coverage of oligonucleotides.^74^ Furthermore, current non-proprietary oligonucleotide mass calculators^28,75–77^ are restricted to McLuckey cleavages as described in Scheme 1. Therefore, oligonucleotide mass calculators have potential to include base loss of other ions such as *w* or *y*, and internal fragment calculations.

Tandem MS (MS/MS) usually requires the isolation of a desired precursor ion, followed by fragmentation. In contrast, a data-independent analysis (DIA)^78^ technique, such as two-dimensional mass spectrometry (2DMS),^39,40,52,54,58,79–84^ allows for the simultaneous analysis of multiple precursor ions without requiring precursor isolation or chromatographic separation.^85^ In 2DMS, product ions are correlated to the precursor ion by their modulation frequency and processed by 2D-FFT (fast Fourier-transform).^86^ The current approach for 2DMS is the summation of a single scan over *N t*_1_ rows.^83^ However, Barna and Lau, 1987, state that truncating the data optimises sensitivity for a typical 2D nuclear magnetic resonance (NMR) experiment, and that ‘*N t*_1_ points with 2*m* scans per point’ increases the signal-to-noise ratio (SNR) as opposed to ‘2*N t*_1_ points with *m* scans per point’.^87^ In 2DNMR, signal truncation often occurs due to signal decay over time,^87^ which is usually not a problem in 2DMS.^82^ However, in this case, the observed fragment ion signal intensity may not be consistent from one scan to another, particularly because of the different phase accrued.^82^ As a result, the potential for averaging the *m* scans over *N t*_1_ rows could enhance the performance of 2DMS.

This study will focus on characterising the sequence of gapmer ASOs, with a PS backbone and either a LNA or cEt modifications on both ends of the oligonucleotide chain by various fragmentation techniques in tandem with MS. Furthermore, a novel approach of employing 2DMS was applied to characterise the ASO, resulting in enhanced peak intensity through scan accumulation, as well as fragmentation efficiency from different charge states of oligonucleotides when using EDD. Data analysis was carried out using a custom-built Python script, which incorporates various types of neutral loss and McLuckey cleavages, including base loss apart from *a-B*, which to our knowledge has not been done on an open-source software before.

## 2. Experimental section

### 2.1 Chemicals

Antisense oligonucleotides danvatirsen (otherwise known as AZD9150)^20^ and MALAT-1, both 16-mer with a phosphorothioate backbone were obtained from AstraZeneca (Macclesfield, UK and Gothenburg, Sweden). Danvatirsen features cEt as a bridged linker on both ends of the nucleobase, while MALAT-1 incorporates LNA as the bridged linker on both ends of the nucleobase. Additionally, some of the bases of ASOs were modified by the incorporation of a methyl group, and these structures were addressed in a comprehensive review by Duffy *et al.*^88^ These ASOs were subsequently desalted using 10 μM ammonium acetate (Merck, Gillingham, UK) in HyperSep™ C18 plates from Fisher Scientific (Loughborough, UK). Compounds were collected in an 80 : 20 mixture of acetonitrile (VWR, U.S.A) and water (purified through a Millipore Direct-Q purification system (18.2 Ω; Merk Millipore, MA, U.S.A.)) and diluted to 5 μM with a 90 : 10 mixture of water and methanol (Merck, Gillingham, UK) with 0.1% triethylamine (Merck, Gillingham, UK).

### 2.2 Mass spectrometry

The two ASOs were ionised using as home-built nano-electrospray ion source (nESI)^89^ and analysed with a 12 T Bruker solariX FT-ICR mass spectrometer (Bruker Daltonik, GmbH, Bremen, Germany). A total volume of 10–15 μL for 1DMS and 25–30 μL for 2DMS was loaded into a glass capillary tip pulled by a Sutter P-97 Flaming/Brown micropipette puller (Sutter Instrument Co., Novato, Ca, USA), and the electrical connection was formed using a nichrome wire. Samples were ionised in negative mode where the [M − 8H]^8−^ was isolated using the quadrupole (isolation window of 5–10 *m*/*z*) and accumulated in the collision cell (∼0.1–0.5 seconds), ensuring full isotopic envelope with good precursor intensity. Ions were subjected to collisions using argon gas (∼6.5 × 10^−6^ mbar) in the collision cell at optimised collision voltages of −6 to −10 V for CID experiments, which were subsequently transferred and detected in the infinity cell.^90^ In-cell fragmentation methods such as IRMPD, UVPD and EDD were performed on the isolated and accumulated precursor ions. IRMPD was performed using a 10.6 μm continuous wave 25 W CO_2_ laser (Synrad Inc., Washington, USA) operated at 11–27.5% laser power with 0.06–0.1 s irradiation time. UVPD experiments were performed using a 193 nm (photon = 6.4 eV) ArF Excimer laser (ExciStar XS, Coherent) with a pulse energy of 2.6–3.4 mJ (1–2 shots), measured at the laser exit aperture. EDD was performed by irradiating the ions with electrons from a 1.5 A indirectly heated cathode with a bias of 22.4–22.8 eV for 0.2–0.5 s. All data were acquired with a 4 M (2^22^, 32-bit), 1.67 s transient, average resolving power >400 000 (fwhm) at *m*/*z* 400.

### 2.3 2DMS

(i) 2D-EDD experiments was performed using the method described by O'Connor and co-workers.^54,84^ Trapped ions were irradiated by electrons with a bias of 22.8 eV for 0.5 s. Data was acquired a 1 M (2^20^, 32-bit), 1.67 s transient in the fragment *m*/*z* (*x*) axis, and was summed over *N* = 2048 *t*_1_ rows yielding a 1.73 ms transient in the precursor *m*/*z* (*y*) axis. (iia) 2D-UVPD was performed using the same approach as the 2D-EDD, with 1 shot of 3 mJ laser energy and acquired with 1 M, 1.67 s transient in the fragment *m*/*z* (*x*) axis, which was subsequently scanned over *N* = 1024 *t*_1_ rows yielding a 0.865 ms transient in the precursor *m*/*z* (*y*) axis. Additionally, (iib) 2D-UVPD was accumulated and averaged for 8 scans for the same number of *x* and *y* data points in (iia). Accumulation was achieved by passing a list of increment delays (*t*_1_) which was constant for *m* = 8 scans, as shown in ESI Fig. S2.†

### 2.4 Data Processing

All spectra were processed and analysed using Bruker DataAnalysis 5.0 software (Bruker, Bremen, Germany). Peak picking was achieved using the in-built algorithm Smart Numerical Annotation Procedure (SNAP)™ with a quality factor >0.4 and average number of constituent elements for each oligonucleotide sequence. Peaks omitted from the algorithm were added manually to ensure full structure assignment. 2DMS was processed by SPIKE^86^ and analysed using an in-house LabView-based program, T2D. 2D-EDD was peak picked using SNAP™, whereas 2D-UVPD displayed the full isotopic patterns peak picked by T2D, which was due to the low intensity of the peaks, especially the *N* = 1024 *t*_1_ rows with *m* = 1 scan. Furthermore, 2DMS was internally calibrated using peaks assigned from the [M − 8H]^8−^ fragment *m*/*z* (*x*) axis, except for 2D-UVPD *N* = 1024 *t*_1_ rows with *m* = 1 scan, which was externally calibrated from the 2D-UVPD accumulated (*m* = 8) scans. 2D peaks were denoised during data processing using denoising algorithms (urQRd and sane), publicly available in SPIKE.^86^ All peaks were assigned using an in-house Python script, which can allow for modifications to nucleobases, phosphate backbone, and select neutral losses and is publicly available at https://github.com/MKRahman97/Oligonucleotide_mass_calculator. Internal fragments were calculated based on the methods proposed by Lantz *et al.*,^71^ and adapted for oligonucleotides.

## 3. Results and discussion

Desalting of the two ASOs, danvatirsen and MALAT-1, by C18 plates generated relatively clean MS spectra, which was analysed by nESI, ESI Fig. S3 and S4.† Some adducts were still present, such as sodium, di-sodium and a PO impurity within the PS backbone, which are highlighted in ESI Fig. S5 and S6.† Careful consideration of a charge state selection is critical to obtain a comprehensive sequence coverage.^91^ A lower charge-state reduces the extent of internal fragments when using CID, allowing for better sequence coverage,^51^ but at the expense of sensitivity due to the low abundance of lower charge state precursors from ESI.† Thus, to consistently compare the effect of fragmentation techniques of the two ASOs, the [M − 8H]^8−^ was isolated, which was in high abundance for both ASOs, ESI Fig. S7 and S8.† With the ability to apply different fragmentation methods, the effects of CID and EDD on MALAT-1 and danvatirsen was examined. Mass lists for the fragment ions of MALAT-1 and danvatirsen are available in ESI Tables S1–S4 and S5–S8† respectively.

### 3.1 Electron detachment dissociation and collision-induced dissociation of MALAT-1 and danvatirsen

**Fig. 1:**
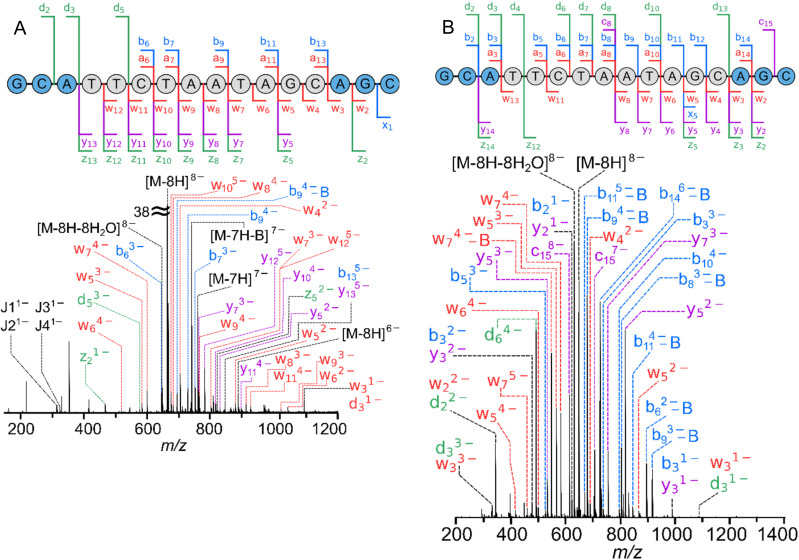
(A) EDD-MS/MS (22.4 eV bias with 0.2 s irradiation time) (B) CID-MS/MS (−6.2 V) and spectra of MALAT-1, [M − 8H]^8−^, with cleavage diagrams overlaid on top. Fragments generated from base and neutral loss were simplified to the McLuckey cleavages in the sequence diagram and internal cleavages are marked by *J*, see ESI Tables S1 and S2† for full peak list.

Complementary cleavages *a*/*w*, *d*/*z* and the non-complementary *b*/*y* ions were predominantly seen with EDD. For MALAT-1 (Fig. 1, ESI Tables S1 and S2†), cleavages were mostly localised to the *w*, *y*, and *z* ions, which could be due to the location of the charge, or the stability of the electron radical resulting from the breaking of the bond (Scheme 1). On the other hand, danvatirsen, exhibited a more balanced distribution of McLuckey cleavages, as evident in ESI Fig. S9 (Table S5).† Notably, the two ASOs demonstrated variations influenced by specific nucleobases present. In cases, where there were two or more ions that have the same *m*/*z* but with different chemical structures such as *b-H*_*2*_*O* and *a-2H*, both were taken as possible cleavages and are made evident in ESI Tables S1–S8.† Likewise for isomeric cleavages such as *d* and *w* ions that consist of the same nucleotide due to the symmetry of the sequence are represented by black dashed lines in Fig. 1.

Internal cleavages (Scheme 2), which contain neither termini, have a range of possibilities, especially when multiple nucleobases are present resulting in different cleavages with the same *m*/*z* such as *aw*, *bx*, *cy*, and *dz*. Note, these refer to cleavages of both *a* and *w* on the backbone with the charge retained in the middle (Scheme 2). Therefore, these were given the character *J* (and not *I* to confuse with intensity) to summarise the possibilities described in ESI Tables S1–S8.† Further fragmentation at the MS^3^ level or MS/2DMS^39^ is necessary for internal fragment discrimination. Due to the complexity of the spectra not all product ions are labelled in Fig. 1, although they are stated in ESI Tables S1 and S2.†

Fragmentation of both MALAT-1 (Fig. 1B and ESI Table 2†) and danvatirsen (ESI Fig. S10 and Table S6†) was conducted using CID with an optimised collision voltage (*i.e.*, a voltage that yielded an informative fragmentation pattern, with minimal secondary fragmentation occurring, whilst preserving ∼50% of the intensity of the precursor ion). McLuckey cleavages with high abundance was observed adjacent to the [M − 8H]^8−^ precursor ion, which dissipated in abundance similar to a pattern of a normal distribution. CID of these modified oligonucleotides did not retain the site-specific *a-B* product ions previously studied in literature.^50,92^ The absence of *a-B* fragments, along with the prevalence of *a*/*w*, *d*/*z*, and non-complementary *b*/*y* series, can be attributed to the reaction pathways discussed by Monn and Schurch.^48^ Remarkably, Fig. 1B (ESI Table 2†) displays an abundance of *b-B* ions when using CID, a phenomenon that has not previously been reported in literature. Consequently, it is possible that an alternative reaction pathway is responsible for these observations, which warrants further investigation. Additionally, there no non-proprietary software to calculate base losses for fragment ions such as *b-B*. Therefore, we have developed a program for identifying base losses from ions outside the typical *a-B* ion, as discussed in section 2.4.

MALAT-1 underwent extensive fragmentation by both CID- and EDD-MS/MS, as displayed in Fig. 1, resulting in the cleavage of almost all of its nucleotides except for one, which was located between the guanine and cytosine and consists of the LNA groups (Scheme 1). Generally, a high degree of fragmentation was observed for the middle of the DNA chain, whereas fragmentation was sparse for the LNA end groups due to the modifications within the sugar and linker groups. Similarly, the majority of the cleavages were located within the centre of the DNA chain for danvatirsen as well (ESI Fig. S9, S10 and Tables S5, S6†). In this case, complete sequence coverage was observed. Since the two ASOs consist of similar modifications (*i.e.* cEt for danvatirsen and LNA for MALAT-1), we speculate that the extent of sequence coverage may be dependent on the type of nucleobase in the oligonucleotide, which for EDD was previously shown to be G^−^ > T^−^ > C^−^ > A^−^.^67^ However, it is important to note the measurements from Kinet *et al.*,^67^ were obtained on oligonucleotides exclusively with the same nucleotide, whereas mixed oligonucleotides may exhibit a different effect.^68^ A reliable trend of nucleotide-based fragmentation cannot be determined by our data due to several variables such as modifications and combined effects from mixed nucleobases. The EDD fragmentation method reported by Hakansson and co-workers suggested that, due to the short timescale of EDD, fewer secondary cleavages by base loss was observed compared to CID.^68^ We observe similar trends in our MS/MS experiments, which is summarised in Table 1.

Table summarising total number of peaks and cleavages observed by four fragmentation methods for MALAT-1 and danvatirsen

**Table:** 

	CID	EDD	IRMPD	UVPD
	MALAT-1			
Total peaks^a^	120	63	143	109
Backbone-retained peaks^b^	3	6	5	5
Assigned peaks^c^	93	44	80	73
McLuckey cleavages^d^	28	18	26	24
McLuckey cleavages with neutral loss^e^	32	3	27	10
McLuckey cleavages with base loss^f^	21	12	13	15
Internal cleavages^g^	8	4	8	19
Unassigned peaks^h^	27	19	63	36
Assigned peaks^i^ (%)	78	70	56	67
Sequence coverage (%)	94	94	94	100
Fragmentation efficiency^j^ (%)	86	8	87	57

**Table:** 

	Danvatirsen			
Total peaks^a^	174	121	120	158
Backbone-retained peaks^b^	4	5	0	9
Assigned peaks^c^	91	87	66	148
McLuckey cleavages^d^	36	21	25	45
McLuckey cleavages with neutral loss^e^	17	3	8	13
McLuckey cleavages with base loss^f^	29	49	24	70
Internal cleavages^g^	6	9	9	13
Unassigned peaks^h^	83	34	54	10
Assigned peaks^i^ (%)	52	72	55	94
Sequence coverage (%)	100	100	100	100
Fragmentation efficiency^j^ (%)	83	28	88	56

a The total count of peaks observed in the mass spectrum after clustering the isotope pattern (note, the isolated precursor ion is included in this tally).

b Precursor ion fragmentation without backbone cleavage.

c The cumulative count of peaks due to McLuckey cleavages, McLuckey cleavages with either base loss or neutral loss, and internal cleavages.

d Total number of peaks attributed to McLuckey cleavages (Scheme 1).

e The count of McLuckey cleavages accompanied by neutral losses such as water, ammonia, and two atoms.

f The total number of McLuckey cleavages involving the loss of a nucleobase from the fragment ion.

g Internal fragments exhibit McLuckey cleavages on both ends of the chain and contain neither termini (Scheme 2).

h Unassigned peaks that do not correspond to McLuckey cleavages or internal fragments.

i The ratio of assigned peaks to the total number of peaks, expressed as a percentage.

j Fragmentation efficiency, which was determined using eqn (1).^55^

### 3.2 Photodissociation using ultraviolet photon dissociation and infrared multiphoton dissociation of MALAT-1 and danvatirsen

**Fig. 2:**
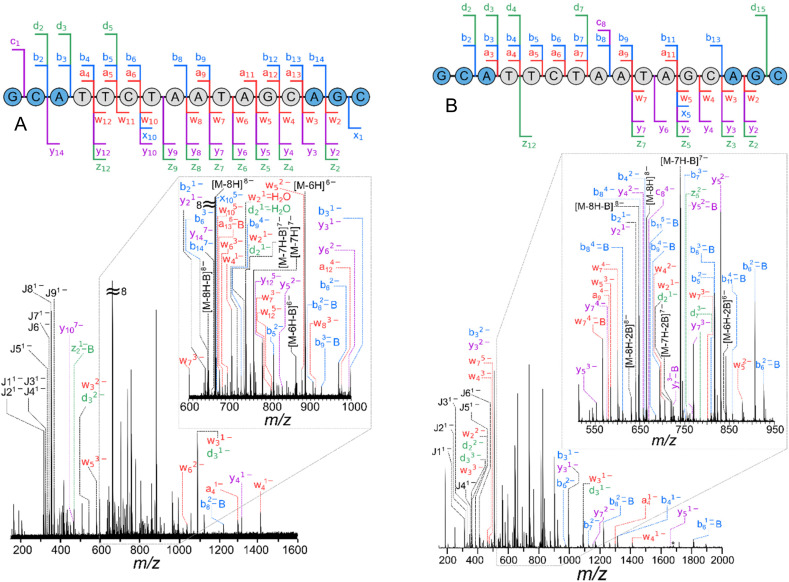
(A) UVPD-MS/MS (2.6 mJ with 1 shot) and (B) IRMPD-MS/MS (11% laser power for 0.1 s) spectra of MALAT-1, [M − 8H]^8−^, with cleavage diagrams overlaid on top. Fragments generated from base and neutral loss were simplified to the McLuckey cleavages in the sequence diagram and internal cleavages are marked by *J*, see ESI Tables S3 and S4† for full peak list.

In UVPD and IRMPD experiments, which are both photon-based dissociation methods, the number and intensity of fragments are moderated by their irradiation time and laser power. Typically, the method involves increasing the laser power to enhance the proportion of backbone cleavages until the intensity of the product ions reaches a plateau.^70,93^ Therefore, the laser power and exposure time was adjusted until the intensity of the product ions were fairly consistent for both MALAT-1 (Fig. 2 and ESI Tables S3 and S4†) and danvatirsen (ESI Fig. S11, S12 and Tables S7, S8†). Numerous internal fragments, denoted by *J*, were observed, as well as *a*, *b*, *d*, *w*, *y*, and *z* ions for both methods, as shown in Fig. 2, albeit with limited *c* and *x* ions as previously shown in the literature.^70^

Peak intensities for all assigned McLuckey cleavages are summarised in ESI Fig. S14,† which shows the relative intensity (*y*-axis) of peak densities (*i.e.* number of peaks) across the *x*-axis, specified for each fragmentation technique. UVPD evidently shows fragment ion intensity similar to CID and superior ion intensity to EDD (ESI Fig. S14†). In addition, complete sequence coverage was seen for MALAT-1, solely from UVPD, with a new bond cleavage across the cytosine guanine residue denoted by the *c*_1_ ion. The emergence of charge-reduced precursor ions [M − 7H]^7−^ and [M − 6H]^6−^, originating from the isolated [M − 8H]^8−^ precursor ion, was observed when electron detachment occurs in conjunction with a proton.^64^ As a result, these precursor ions undergo secondary fragmentation, which generates additional product ions and increases the extent of sequence coverage. Finally, UVPD at 193 nm also produces the most internal fragments (*J*), which contain neither termini, underscoring the ease with which these bonds can break. These internal fragments exhibit similarities to those reported in prior literature when solely using UVPD; however, when using a combination of UVPD and CID, termed electron photodetachment dissociation (EPD) on a charged-reduced precursor ion can reduce the yield of internal fragments.^70,94^

Conspicuously, product ions of the highest intensities were seen using IRMPD (as shown in Fig. 2 and relative peak intensities in ESI Fig. S14†), which can be attributed to the enhanced photon absorption at 10.6 μm from the phosphate groups.^53,95^ Nonresonant ion activation was found to reduce the uninformative base loss from the precursor when using CID into *a-B* and *w* fragment ions.^93^ These base loss cleavages do not provide any sequence information as the backbone is retained.^93^ Nevertheless, IRMPD of these modified oligonucleotides increased the number of the uninformative base loss ions, as well as generating charge reduced anions with base loss. The number of internal fragments was comparable to UVPD, where base loss of A and T is dominant for both techniques. IRMPD, like CID and EDD displayed the same sequence coverage with the absence of the G and C cleavage on the 5′ end. A full comparison of all four techniques for both oligonucleotides is discussed in section 3.3.

### 3.3 Comparison of CID, EDD, IRMPD, and UVPD for MALAT-1 and danvatirsen

By taking a multimodal fragmentation approach using CID, EDD, IRMPD and UVPD, complementary information can be obtained, allowing for more confident structural characterisation of an oligonucleotide. Each technique has its advantages and limitations, and in some cases yields complex spectra with numerous ambiguous and unassigned peaks. The total number of peaks, McLuckey cleavages (Scheme 1), McLuckey cleavages with neutral losses (such as water and ammonia, which is shown in ESI Tables S1–S8†), McLuckey cleavages with base loss and internal (which refer to base losses for all product ions *i.e.* the ones outside the standard *a-B*, and cleavages on both sides of the sequence, otherwise known as internal fragments), unassigned peaks, and fragmentation efficiency are summarised in Table 1. Fragmentation efficiency was calculated from eqn (1) reported by Brodbelt and co-workers,^55^ where *F*_*i*_ is the abundance of each product ion and *P* is the intensity of the precursor ion after ion activation.1
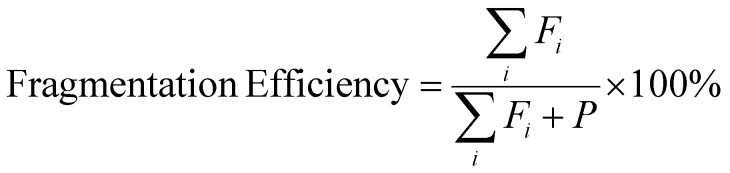


McLuckey cleavages (Scheme 1) break the oligonucleotide across its phosphate backbone, generating fragment ions that help to elucidate the exact sequence of the oligonucleotide. Generally, a consistent number of McLuckey cleavages was observed across the four different fragmentation techniques. However, when accounting for the neutral losses, a significant distinction of the number of cleavages between techniques was evident. Neutral losses such as water or ammonia from the McLuckey cleavages can occur (ESI Tables S1–S8†) which was shown to be significant by the sheer number of cleavages in Table 1. Possible neutral losses can be attributed to the pathways described by Wan *et al.*^92^ Fragmentation of danvatirsen displayed the largest increase of neutral losses when using UVPD, which was not observed for MALAT-1, and this could be attributed to the differences in the backbone *i.e.*, LNA for MALAT-1 and cEt for danvatirsen.

Internal fragments previously aided in structural elucidation of unknown peaks, which enhanced the sequence coverage observed of proteins.^71^ Due to the limited number of nucleotides (5), and relatively large number of amino acids (20), short stretches of nucleotides tend to be repeated. Although the number of repeated nucleotides can be reduced by modifications to the nucleobase, sugar, and linker, it is still not adequate to eliminate the possibility of repeated nucleotides within the assigned internal fragments. Consequently, within each peak, there exist numerous potential assignments for internal fragments. To simplify the analysis of isomeric ions generated from a given *m*/*z* value, multiple possible fragment ions were grouped together and counted as one. This approach was adopted due to the challenge of determining the specific combination responsible for each peak. Base loss and internal fragments peaks were similar across CID and both photodissociation techniques, where the fewest were observed with EDD. Interestingly, the occurrence of base loss in EDD is not universally observed; however, prior literature suggests that the presence of a base can significantly impact the fragmentation pattern.^67^ While it cannot be completely ruled out, it is possible that any base loss events are present at levels below the detection threshold due to the low fragmentation efficiency when using EDD.

Characterising oligonucleotides involves assigning the product ions from a known sequence in tandem MS experiments. However, fragmentation of these modified oligonucleotides yields intricate mass spectra, which can have complications to analyse particularly when peaks are closely spaced. By achieving high mass resolution, ions of similar *m*/*z* values can be distinguished, as well as improving mass accuracy and precision. In addition, high mass resolution allows for the detection of fine isotopic peaks, which can be used to determine the elemental composition of a compound.^44^ For example, the correct number of sulfur atoms can be calculated solely from the ratio of the resolved ^34^S peak area to the monoisotopic peak area as described in ESI Fig. S13†.^44^ A total of four sulfur atoms was calculated, which was expected for a *w*_4_^2−^ fragment ion with a phosphorothioate backbone.

Ideally, there should be sufficient peaks for full structural characterisation, without additional complexity. Danvatirsen exhibited complete sequence coverage using any of the dissociation techniques (as shown in ESI Fig. S9–S12†), consequently, the technique of choice can be determined by the proportion of assigned peaks, which for CID and IRMPD was limited to ∼50%. Contrastingly, product ions from EDD and UVPD display the most McLuckey cleavages, with UVPD resulting in ∼94% assignment coverage. Although fewer peaks were observed when using EDD, the structural information observed from McLuckey cleavages remained intact, which simplifies data interpretation. Base loss exclusively occurred within the DNA sequence and was not observed in the LNA strands for both oligos, consistent with findings in existing literature.^96^

Complete sequence coverage determined by tandem MS of MALAT-1, previously discussed in sections 3.1 and 3.2, was limited by a G and C cleavage within the cEt group at the 5′ end. Interestingly, CID and IRMPD both exhibited a high quantity of peaks and greatest fragmentation efficiency (Table 1) but did not fully characterise the sequence, whereas full sequence coverage was observed solely by UVPD. In situations where there was minimal fragmentation efficiency (based on the intensity of fragment ions) as shown by EDD, the ratio of peaks assigned was comparable to UVPD, which makes EDD a viable technique for oligonucleotide characterisation.

**Fig. 3:**
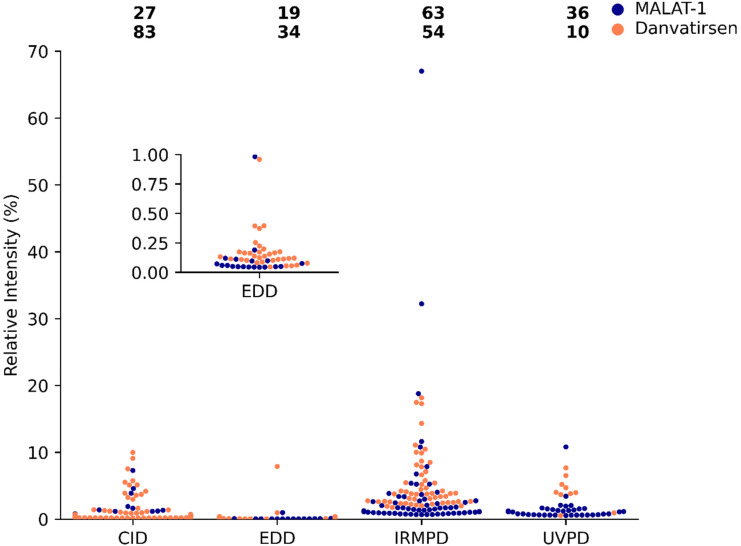
Beeswarm plot of the relative intensities for each unassigned peak of MALAT-1 (navy) and danvatirsen (orange) produced by tandem MS using CID, EDD, IRMPD, and UVPD. Each point represents a single peak, which is evenly spaced in the *x*-axis to prevent overlap, where 50–60% of the peaks are plotted due to the large number of peaks. Zoom-in on EDD is provided due to low abundance of peaks. Total number of peaks is overlaid on top, see Table 1. Similar plot for assigned peaks can be found in ESI Fig. S14.†

Although the majority of the fragment ions can be assigned as McLuckey cleavages, and McLuckey cleavages with losses discussed in Table 1, the number of unassigned peaks cannot be ignored. Relative peak intensities for the unassigned peaks generated by the four fragmentation techniques are summarised in Fig. 3.

Without appropriate validation, peaks outside the standard McLuckey cleavages cannot be conclusively assigned and have been noted in ESI Tables S1–S8† with their respective *m*/*z*. Differences in relative peak intensities of the unassigned peaks in Fig. 3 compared to the assigned McLuckey cleavages in ESI Fig. S14† were found to be minimal. Similar peak intensities indicate that alternative reaction pathways could occur for these modified oligonucleotides to yield fragment ions of similar stabilities to McLuckey ions, which could be due to the different location of charge sites or secondary fragments from McLuckey cleavages.^48^

Fragmentation efficiency between CID and IRMPD were found to be similar for both oligonucleotides as shown in Table 1. As a result, it is anticipated that the peak intensities will be comparable in values. Conversely, Fig. 3 (fragment ions that remain unassigned) illustrates that the relative peak intensity of IRMPD is substantially greater, which suggests that majority of the product ions produced fall outside the standard McLuckey cleavage and currently remain unknown. Where CID and UVPD both have comparable relative peak intensities (Fig. 3), the fragmentation efficiency of UVPD is lower (Table 1). Hence similar peak intensities for both unknowns and assigned peaks (ESI Fig. S14†) were seen by UVPD. Although, negligible peak intensities with relative abundances of <2% were observed when using EDD, the majority of the fragment ions can be assigned to McLuckey cleavages (where ESI Fig. S14† shows no significant enhancement of relative intensity for the assigned peaks). Therefore, it can be concluded that the majority flux of product ions yields McLuckey cleavages and its analogues when using CID or UVPD.

### 3.4 Two-dimensional electron detachment dissociation mass spectrometry of danvatirsen

2DMS is a data independent analysis technique that can correlate the fragments to the precursor ion based on their modulation frequencies. Therefore, tandem MS spectra of multiple precursor ions can be obtained simultaneously. Given that complete sequence coverage was achieved regardless of the dissociation technique, danvatirsen was suited for this study. Furthermore, to avoid complications of ion suppression from a complex mixture of oligonucleotides, this study will be focusing on the multiple charge states of a single oligonucleotide. Currently, 2DMS is restricted to electron- and laser-based fragmentation to avoid the use of collision gas for CID in the ICR cell. As discussed in section 3.3, IRMPD was found to produce complex spectra without providing additional structural information, and therefore is no longer considered in this study. Thus, fragmentation using EDD and UVPD (section 3.5) was investigated in tandem with 2DMS.

Initial examination of the 2D-EDD mass spectrum obtained displayed three high intensity fragment lines, corresponding to the three most abundant charge states of danvatirsen. The autocorrelation line (ESI Fig. S16A and Table S9†) displays all the precursors, and in this case eight different charge states were visible; however, the signal was lost in the noise for all precursors with a lower relative abundance than [M − 5H]^5−^. Additional precursor ions observed in 2DMS, as opposed to 1DMS (refer to ESI Fig. S3†), may be attributed to potential oxidative degradation occurring when using nESI during the extended runtime of 2DMS experiments (ESI Fig. S16†),^97^ which typically span 1–2 hours, or due to inherent instability of the sample in solution.

A decrease in precursor ion intensity (ESI Fig. S16A and Table S9†) leads to a corresponding decrease in the intensity of the resulting fragments extracted from the fragment line (ESI Fig. S16B–D and Tables S10–12†). Through comparison of the same charge state, [M − 8H]^8−^, between the 2DMS (ESI Fig. S16D†) and 1DMS (ESI Fig. S9†) experiments, greater sequence coverage is observed in 1DMS, and this could be attributed to the enhanced signal of the fragments in 1DMS. A total of 250 scans were accumulated and averaged in the 1DMS, whereas 2048 scan lines were incremented across all precursor ions in 2DMS. Further, due to the increment delay in the 2D pulse sequence,^82^ the precursor ion may not always undergo consistent fragmentation. However, full sequence coverage can be observed in the 2DMS when accounting for all the charge states.

Fragmentation across multiple charge states was shown to be inconsistent (ESI Fig. S16†) an effect previously seen with 2DMS when a common evolution pulse is used for different precursor ions.^98^ Since the evolution pulse is required for precursor ion modulation and subsequent separation in the *y*-dimension, the pulse used can impact the level of fragmentation (ESI Fig. S17†) which was exclusively carried out for the [M − 8H]^8−^ precursor ion. Therefore, an average pulse was used for the whole spectrum,^84^ to limit the bias for a particular precursor ion.

The vertical lines in 2DMS displays common fragments shared across different precursor ions. One example can be seen in ESI Fig. S16E,† which displays *w*_8_^3−^ fragment shared across multiple charge states of the same precursor ion. Although this is not entirely unexpected, the method can be expanded to identify overlapping sequences within different ASOs. For example, an ASO with the sequence AATTA and another with the sequence AAT can be recognised as having the same subsequence even when they occur in distinct ASO, which can become valuable in evaluating similar sequences with an impurity. This approach is similar to a precursor ion or product ion scan. However, conducting such experiments would necessitate multiple iterations and could consume a significant amount of time. Furthermore, employing a 2DMS plot facilitates improved visualisation of shared fragments and enables the identification of additional details, such as neutral losses (*e.g.*, water) for each precursor ion.

### 3.5 Effect of charge state on EDD fragmentation efficiency

**Fig. 4:**
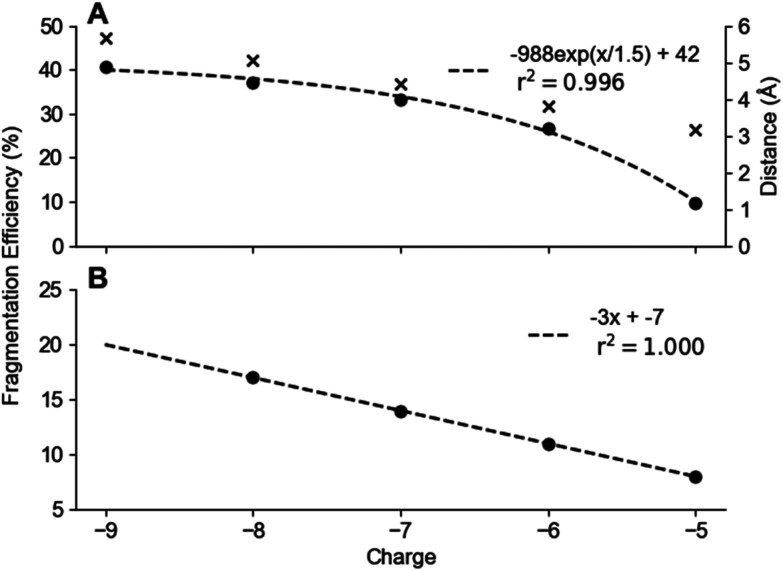
Fragmentation efficiency for each charge state of danvatirsen when using EDD at 22.8 eV bias for an irradiation time of 0.5 s in a (A) 1DMS experiment and (B) 2DMS experiment. Distance of closest approach for an electron to the precursor ion of a particular charge state is marked by a cross (‘*x*’).

An important observation of 2D-EDD-MS (ESI Fig. S16†) was the low abundance of fragment ions, particularly for the lower charge states. It was then investigated the trends of charge states in both 2DMS and 1DMS by maintaining the EDD bias to an optimised value of 22.8 eV for danvatirsen, which can be seen in Fig. 4. Fragmentation efficiency from precursor ions with charge states ranging from [M − 9H]^9−^ to [M − 5H]^5−^ was measured for tandem MS experiments. Whereas, for 2DMS a smaller range of [M − 8H]^9−^ to [M − 5H]^5−^ was used, since fragment ion intensity of those generated from the [M − 9H]^9−^ precursor ion was within the noise peaks.

The model of EDD described by Budnik *et al.*, is considered the standard for this mechanism.^65^ Electron detachment creates a charge-reduced intermediate state, which undergoes non-ergodic dissociation at electron energies >10 eV.^65^ As shown in Fig. 4A, highly charged precursor ions yield rich fragmentation, which reduces for lower charge states; an observation which is not too dissimilar to ECD of peptides and proteins.^99^ Therefore, it can be concluded that a densely charged precursor ion promotes strong intramolecular repulsions, which in turn facilitates dissociation.^66^

Furthermore, a more recent study of EDD of modified oligonucleotides by Karasawa *et al.*, reported a similar trend, which uses a neutral electron-nitrogen plasma to lessen the coulombic repulsion.^66^ Since, the electron beam experiences repulsive coulombic forces from the precursor anion, a higher charge state would reduce the fragmentation efficiency, as shown by EDD of 61-mer RNA.^69^ However, the trend in Fig. 4 suggests otherwise, which could be due to the 100-fold increase in dissociation efficiency of a PS backbone compared to the standard PO backbone,^66^ hence diminishing the effects of coulombic repulson.

By considering the distance of closest approach, as determined by the equation *q*_1_*q*_2_/4π*ε*_0_*V* (where q is the electron charge, *ε*_0_ is vacuum permittivity and V is the electron bias), the electron distances can be calculated. The manifestation of coulombic repulsion becomes apparent as the charge states exhibit electron proximity ranging from approximately 3.16 Å to 5.68 Å (Fig. 4A), with the smaller charge states demonstrating comparatively shorter electron proximity. Furthermore, this observation confirms the presence of significant intramolecular repulsions in the higher charge states, causing their dissociation when an electron is within ∼5 Å of them.

In the 1DMS analysis of danvatirsen (Fig. 4A), precursor ions with lower charge states result in exponentially lower fragmentation efficiency. In general, higher charge states demonstrate greater propensity for dissociation compared to lower charge states, thereby elucidating this observed trend. Conversely, fragmentation efficiency decreases linearly with decreasing charge state when 2DMS is used. One explanation for this phenomenon resides in the time-modulation used in 2DMS,^82^ which can significantly impact the level of fragmentation due to the varying positions of precursor ions from one scan to another. Thus, providing different trends when compared to 1DMS. Additionally, the fragmentation efficiency observed in 2DMS is approximately half of the standard 1DMS (Fig. 4) despite the large number of scan lines, thus confirming precursor ions outside or bordering the fragmentation zone of the electron beam will not undergo efficient dissociation. A potential solution to this problem was proposed by developing a 2DMS method, which accumulates and averages the data instead of incrementing over *N* scan lines.

### 3.6 Accumulate two-dimensional ultraviolet photodissociation mass spectrometry of danvatirsen

**Fig. 5:**
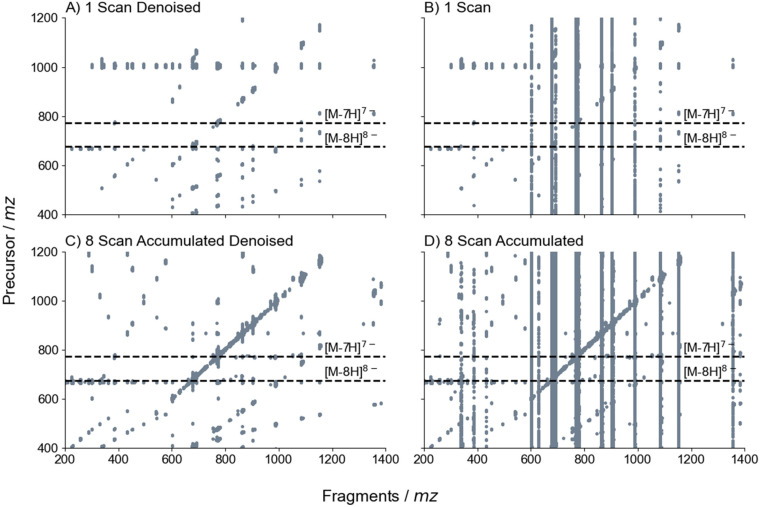
2D-UVPD-MS of danvatirsen performed with 3 shots of 2 mJ using ArF laser at 193 nm when (A) a single scan (1 M *x*-axis by 1024 *y*-axis data points) was denoised using sane (rank = 10) and (B) prior to denoising. Subsequently, (C) accumulated and averaged (1 M *x*-axis by 1024 *y*-axis data points) for 8 scans with denoising sane (rank = 10) and (D) prior to denoising. Fragment lines (*x*-axis) corresponding to the [M − 8H]^8−^ and [M − 7H]^7−^ precursors are available in ESI Fig. S18 and S19,† respectively.

Complete sequence coverage was observed for both gapmer oligonucleotides when using UVPD, which was discussed in section 3.3. Consequently, UVPD coupled to 2DMS was a logical progression, especially with the recent success of 2D-UVPD-MS when applied to other classes of molecules.^58^ We therefore investigated the effects of 2D-UVPD-MS of danvatirsen as shown in Fig. 5, similar to 2D-EDD-MS discussed in section 3.5.

The methodology behind section 3.5 outlines the typical approach for conducting 2DMS experiments, where a single scan is summed over *N* scan lines (*t*_1_ rows), which corresponds to the number of increment delays (*t*_1_) as described in Marzullo *et al.*^58^ If the electrospray is stable, it is anticipated that the precursor ion will remain intact, consequently, generating *t*_1_ noise (scintillation noise), Fig. 5B and D, which can be removed by appropriate denoising algorithms,^100^ as shown in Fig. 5A and C.

A qualitative measurement displays that denoising was successful based on the suppression of the vertical streaks in Fig. 5A (ESI Table S14†) and Fig. 5C (ESI Table S18†), where denoising was applied. However, in the standard 2DMS approach using 1024 *t*_1_ rows (Fig. 5A),^58^ the paucity of fragments is readily apparent. As prior research has indicated, laser- and electron-based dissociation have distinct fragmentation zones.^84^ One straightforward experiment was conducted to compare the relationship between the evolution pulse (*i.e.* first pulse applied to separate the precursor ions) and the yield of fragment ions after dissociation, which can be seen in ESI Fig. S17.† A gaussian-shaped laser beam and wide fragmentation regions due to the hallow-cathode was observed, which is consistent with previous observations.^84^ An optimised evolution pulse was determined between 20–30 V_pp_ for 2D-UVPD-MS experiments. One can speculate that the scarcity of fragment ions observed in Fig. 5A is possible due to the short pulse length of the UV laser, illustrating a need to accumulate multiple scans per *t*_1_ increment.

A novel 2DMS pulse sequence was devised by keeping *t*_1_ at a constant value across multiple scans (*m* = 8), accumulating data from each scan, and subsequently computing the average. The process involved incrementing *t*_1_ and repeating the iteration, as illustrated in ESI Fig. S2.† SNR was shown to enhance when accumulating 8 scans by the greater number of fragment lines displayed in Fig. 5C (ESI Table S18†). Mass spectra for the respective fragment lines are shown in ESI Fig. S18,† which highlights that this approach increases the number of peaks with improved sequence coverage compared to analysing a single scan alone (60% to 70% sequence coverage). For this case, the [M − 7H]^7−^ displayed the greatest SNR, thus fragments were extracted and compared between the four data sets in Fig. 5, with fragment ions commonly observed across multiple data sets reported in Table 2. Moreover, by accumulating and averaging, the noise harmonic observed at *m*/*z* 1000 (1/2 of high mass cut-off at *m*/*z* 2000) is made redundant (Fig. 5A and B), whilst the third harmonic (at *m*/*z* 667) grew in amplitude, which suggests that the former is random noise in the data.

**Table:** Signal-to-noise ratio of common fragments and precursor with and without *t*_1_ denoising (sane rank 10)

Peak assignment	*w*_8_^3−^	*w*_12_^4−^	[M − 7H]^7−^
Denoised 1 scan	39.6	13.1	1237.5
Denoised 8 scan	65.9	37.7	1147.7
1 scan	11.6	3.8	719.8
8 scan	20.1	11.5	915.6

Denoising is also valuable for eliminating false positives, which can pose a significant challenge in data analysis. All *m*/*z* values were reported in ESI Tables S13–S20† for the denoised and raw data to highlight the difficulty in identifying the appropriate fragment ion. Given that acceptable calibration was only attainable from spectra exhibiting the highest SNR, an approach was adopted involving the accumulation of 8 scans in 2D with denoising; subsequently, the remaining spectra were externally calibrated based on the calibration obtained from the accumulated dataset. By comparing the raw (*i.e.*, no denoising) (ESI Fig. S19 and Tables S16, S20†) and denoised (ESI Fig. S18 and Tables S14, S18†) extracted fragment lines of the [M − 7H]^7−^ precursor ion, a reduction of ∼25% “noisy” peaks were observed, which improves the quality of the mass spectra. Additionally, by accounting for mass accuracy (ESI Tables S13–S20† with average mass errors of <1 ppm) with the application of the denoising approach, it is anticipated that there will be a decrease in the number of false positives.

The SNR exhibits a square root relationship with the number of scans, as the signal is amplified while the random noise averages out in the time domain.^101^ Therefore, a similar effect is anticipated in the time domain (*y*-axis) of 2DMS. Table 2 shows the SNR enhancement of fragment ions by approximately 1.7 to 3-fold when acquiring 8 times as many scans per *t*_1_ increment. Variation in peak intensity can be attributed to the degree of fragmentation and stability of the pulses. Nevertheless, a greater number of fragment ions were observed. Furthermore, denoising was shown to be crucial, as it resulted in a signal enhancement greater than 3-fold and allows for the identification of low-intensity peaks that may be missed in noisy spectra (SNR of 3.8 is within the noise threshold). As a result, SNR enchantment of 6 to 10-fold is expected when coupling multiple-scan accumulations and denoising (one outcome of this is an improvement in sequence coverage, increasing from 50% to 69%).

Nevertheless, a limitation of this approach is the natural increase in accumulation time required for accumulating scans, which in turn necessitates a larger sample volume. However, with the utilisation of nESI, the increase in sample volume can be restricted to approximately 10–15 μL. In addition, the computational processing time required for such analyses can be significant, depending on the available computational power. However, the extensive structural information obtained through 2DMS can greatly enhance the confidence and reliability of the obtained results. Overall, a SNR increase of threefold of the square root of number of scans was observed. Interestingly, the precursors are more similar in SNR, and this could be attributed to the calculations from sane denoising.

Also increasing the number of *t*_1_ rows (scan lines) increases the resolution in the precursor ion dimension, analogous to increasing the transient length in 1DMS. Higher resolution implies narrower peaks, and since peak area is constant, the overall SNR is improved. If a stable signal is observed throughout the whole 2DMS, improvements to SNR would be expected by increasing the number of *t*_1_ rows. However, for low intensity fragments (*e.g.*, *w*_12_^4−^ from Table 2), the level of SNR would reach a limit as the number of scan lines is increased (ESI section 2†). Simulations for 2DMS are shown in ESI section 2 (Fig. S20–S26)† with the respective Python source code.

In 2DMS, an ideal transient would exhibit a lack of noise in both the *x*-axis and *y*-axis, with the *y*-axis being unaffected by decay since it relies on the stability of the electrospray (ESI Fig. S20†). However, noise present in the *x*-axis can impact the observed signal in the *y*-axis, as it is indirectly observed through the *x*-axis (ESI Fig. S21 and 22†). By accumulating scans, it becomes possible to enhance the signal rather than acquiring multiple transients that are enhanced with noise. Furthermore, by averaging multiple scans, the overall data size is reduced (by the number of scans), which greatly reduces computational time for data processing and data storage requirements.

Consequently, the utilisation of 2DMS can be expanded to explore and detect the diverse impurities that may emerge during synthesis.^102,103^ Accumulating scans in 2DMS, as showcased in ESI Fig. S27,† elucidates the presence of a compound roughly 2.3 Da lower than the [M − 7H]^7−^ precursor, which is indicative of a mass difference of about 16 Da, and this can be attributed to a PO impurity. Therefore, by accumulating the spectra, the issue of low-intensity fragment ions being overlooked is effectively addressed, enabling their detection and analysis, and unlocking the full potential of 2DMS.

### 3.7 Data analysis using in-house Python script

Throughout this study, theoretical *m*/*z* values for various product ions were generated using a custom Python script developed in-house. This was necessary as there are no existing open-source software options that adequately consider modifications and calculate masses with base losses for fragment ions, except for *a-B* fragment ions. Hence, it became evident that the development of our own program was essential to fulfil this specific requirement. Fragment ions were verified by cross-checking standard McLuckey cleavages with RoboOligo,^75^ before being used to automate assignment from a Bruker mzXML file. Code availability for the generation of custom mass lists of oligonucleotides is presented in section 2.4.

Firstly, the program for calculating fragment ions is highly flexible, allowing users to modify the nucleobases as they please and create new codes to represent modified nucleobases, as displayed in ESI Fig. S27.† Additionally, various backbones, including PO and PS are viable options. To account for a locked nucleobase (where the chemical formula of the linker can also be modified) a special character “d” (derivatised) was employed. Internal fragment calculations proposed by Loo and co-workers,^73^ were included into this approach. To save computational time, internal fragments were calculated from the initial McLuckey cleavages, with base losses and neutral losses taken into consideration at the end. Finally, by setting a low and high cut-off mass with a maximum number of charge states, the computational time was significantly reduced, generating a mass list within seconds.

Next, automatic mass assignment was performed by first taking into account the charge of each ion and then prioritising masses with low (sub-ppm) mass errors. In cases where multiple fragment ions shared the same *m*/*z* value, all potential combinations were taken into account. Likewise, fragment ions with identical *m*/*z* but distinct neutral losses, such as *b-H*_*2*_*O* and *a-2H*, were considered. It is important for the user to select the correct neutral loss to avoid incorrect assignment. Furthermore, the approach allows the user to customise the neutral losses (and adducts) as they see fit. Charged base loss (CBL)^72^ was not observed with danvatirsen or MALAT-1. However, the calculation was left for the user to explore the potential possibilities. During the analysis of the mass spectrum, additional adducts were observed *e.g.*, sodium adducts, as shown in ESI Fig. S5 and S6.† Consequently, calculations were conducted in a similar manner to the neutral loss calculations, but instead of deducting masses, the masses were summed. Our approach has the potential to include amino acids as well, since the user is not limited to the standard nucleotides. We propose an extension to the source code to include a nucleotide backbone with amino acids as bases and *vice versa*, an application presently unavailable in any existing software program.

## 4. Conclusion

Multimodal activation techniques and 2DMS was employed using an FT-ICR. The FTICR resolving power and mass accuracy advantages were clearly helpful in this research, but the key transformative capability of the instrument in this context is the ability to perform so many different fragmentation methods all on the same instrument, thereby saving resources for the analysis of two gapmer oligonucleotides, namely MALAT-1 and danvatirsen. These modified oligonucleotides exhibited extensive fragmentation, leading to a significantly higher number of cleavages compared to biological counterparts. A consequence of richer product ion spectra is the increased challenge of characterising the fragment ions. Using 1DMS and selecting a specific precursor ion, it was demonstrated that UVPD generated sufficient assignable product ions to enable full characterization of the sequence of both oligonucleotides, with the lowest number of unassigned peaks. Fragment ions with the greatest peak intensities were observed when using IRMPD; however only half of them were successfully assigned. Equivalent sequence coverage and a similar number of assigned fragment ions were observed by both CID and EDD. This study was extended to 2DMS analysis fragmenting all precursor ion charges states of the oligonucleotides. However, CID is not currently compatible with 2DMS, and thus only EDD and UVPD were evaluated.

2D-EDD-MS enabled complete sequence coverage when accounting for all charge states. Precursor lines (vertical lines) in 2DMS identify common fragments among different precursors, which can help to decipher shared regions within the sequence. As a result, we propose 2DMS as a method for potential oligonucleotide sequencing. Higher charge states were shown to exhibit greater fragmentation efficiency when using EDD. We suggest that this enhanced dissociation from the phosphorothioate backbone can counteract the effects of coulombic repulsion.

A pilot study was conducted using 2D-UVPD-MS with accumulation on danvatirsen, which demonstrated its feasibility. Although 2DMS did not replicate the superior sequence coverage observed in 1DMS, we noticed an enhancement of sequence coverage by acquiring multiple scans. Three-fold signal enhancement with the square root of the number of scans was observed when scans were accumulated, averaged and subsequently denoised with existing denoising algorithms, which allows for future 2DMS experiments to be acquired with greater SNR.

Lastly, an advanced non-proprietary software is showcased, enabling oligonucleotide mass calculations with virtually unlimited possibilities for user-defined modifications. This program accounts for base losses for fragment ions outside the standard *a-B*, neutral losses, charged base loss, and adducts.

## Data availability

FTICR datasets are very large, and the datasets in this manuscript sum to about 1 terabyte of data. So far, the public raw data repositories are unable to handle such dataset size. To get around this issue, we create peaklists, and section 2.4 in the experimental section of the manuscript discusses the methodologies used to create such peaklists from the raw datasets. The peaklists are published in the ESI† alongside the paper. Peaks are included whether we can assign them or not, which is why the ESI† is 86 pages long.

Furthermore, if anyone is interested, all of the raw transient data in this report are available upon request from the authors.

The source code used for data analysis is publicly available at https://github.com/MKRahman97/Oligonucleotide_mass_calculator.

## Conflicts of interest

SWH and ADR are employees of AstraZeneca and hold stock ownership or stock interests in the company.

## Supplementary Material

AN-149-D4AN00484A-s001
