# Supplementary material for: Unveiling the intricacy of gapmer oligonucleotides through advanced tandem mass spectrometry approaches and scan accumulation for 2DMS
Source: Analyst. 2024 Jul 18;149(18):4687–701. doi: 10.1039/d4an00484a (PMC11382339; doi:10.1039/d4an00484a)
Supplement: AN-149-D4AN00484A-s001 [file AN-149-D4AN00484A-s001.pdf]

Electronic Supplementary Material (ESI) for Analyst.
This journal is © The Royal Society of Chemistry 2024

Supporting Information - Unveiling the intricacy of gapmer oligonucleotides through advanced tandem mass spectrometry approaches and scan accumulation for 2DMS

Mohammed Rahman[1,2], Bryan P. Marzullo[1], Pui Yiu Lam[1], Mark Barrow[1], Stephen W. Holman[3], Andrew D. Ray[4] and Peter B. OConnor[1]

[1]Department of Chemistry, University of Warwick, Coventry, CV4 7AL, UK

[2]Department of Physics, University of Warwick, Coventry, CV4 7AL, UK

[3]Chemical Development, Pharmaceutical Technology & Development, Operations, AstraZeneca, Macclesfield, SK10 4TF, UK

[4]New Modalities Parental Development, Pharmaceutical Technology & Development, Operations, AstraZeneca, Macclesfield, SK10 4TF, UK

The Supporting information encompasses Supporting experimental mass spectra and modelled spectra, complete with detailed interpretations and mass tables. Additionally, it provides extra experimental details and the Python code for simulation, facilitating future researchers in reproducing these results.

Table of Contents

Section 1: Supporting Experimental Section 6

Section 2: Supporting Figures 6

- Supporting Figure S1 Nomenclature for Oligonucleotide fragmentation based on Mcluckey cleavages based on Scheme 1, where the locked nucleic acid forms a bridge between the 2 carbon and 4 carbon. 7
- Supporting Figure S2 2D Pulse sequence, where the increment delay was fixed by the number of scans and subsequently averaged after accumulation. 7
- Supporting Figure S3 MS spectrum of danvatirsen after desalting using HyperSep™ C18 plates. 8
- Supporting Figure S4 MS spectrum of MALAT-1 after desalting HyperSep™ C18 plates. 8
- Supporting Figure S5 MS spectrum of danvatirsen after desalting HyperSep™ C18 plates highlighting the $[M-8H]^{8-}$ and a phosphate impurity in the phosphorothioate backbone, with the simulated spectrum (bottom). 9
- Supporting Figure S6 MS spectrum of MALAT-1 after desalting HyperSep™ C18 plates highlighting the $[M-8H]^{8-}$, a phosphate impurity in the phosphorothioate backbone, sodium and disodium adducts, with the simulated spectrum (bottom).9
- Supporting Figure S7 MS spectrum of danvatirsen, $[M-8H]^{8-}$, with the simulated spectrum (red). 10
- Supporting Figure S8 MS spectrum of MALAT-1, $[M-8H]^{8-}$, with the simulated spectrum (red). 10

Supporting Figure S9 EDD-MS/MS spectrum of danvatirsen, $[M-8H]^{8-}$, at 22.8 eV bias for 0.5 s irradiation time, with cleavage diagrams overlayed on top. 11
Supporting Figure S10 CID-MS/MS spectrum of danvatirsen, $[M-8H]^{8-}$, at -9.2 V, with cleavage diagrams overlayed on top. 12
Supporting Figure S11 UVPD-MS/MS spectrum of danvatirsen, $[M-8H]^{8-}$, using 2 shots of 3.4 mJ laser power, with cleavage diagrams overlayed on top. 13
Supporting Figure S12 IRMPD-MS/MS spectrum of danvatirsen, $[M-8H]^{8-}$, at 27.5 % laser power for 0.06 s irradiation time, with cleavage diagrams overlayed on top. 14
Supporting Figure S13 Isotope pattern of w_4^{2-} ion ($C_{43}H_{54}N_{16}O_{23}P_4S_4$) of MALAT-1, obtained from UVPD-MS/MS (Supporting Table S4), where the peak areas (%) are overlaid on top. The A+2 peak corresponds to a molecule with one ^{34}S atom (shaded) and two ^{13}C atoms, and its fine isotopic pattern allows for the resolution of these isotopes. This observation confirms the presence of four sulfur atoms, as expected for a phosphorothioate backbone. 15
Supporting Figure S14 Beeswarm plot of the relative intensities for each McLuckey cleavage and its analogues of MALAT-1 (Navy) and danvatirsen (Orange) produced by Tandem MS using CID, EDD, IRMPD, and UVPD. Each point represents a single peak, which is evenly spaced in the *x*-axis to prevent overlap, where 20-50% of the peaks are plotted due to the large number of peaks. Zoom-in on EDD is provided due to low abundance of peaks. Total number of peaks is overlayed on top, see Table 1. 16
Supporting Figure S15 The presence of an additional oxygen in danvatirsen indicates the occurrence of oxidative degradation. The observed low intensity suggests that the precursor is inherently unstable and undergoes degradation upon exposure to oxygen. 17
Supporting Figure S16 2D-EDD-MS of danvatirsen performed with 22.8 eV bias for 0.5 s irradiation time (1M x-axis by 2048 y-axis data points) 18
Supporting Figure S16A Autocorrelation line (equivalent to 1DMS, mass list available in Supporting Table S9) 18
Supporting Figure S16B 2DEDD-MS Fragment line extracted from the $[M-6H]^{6-}$ precursor in Figure 4 19
Supporting Figure S16C 2DEDD-MS Fragment line extracted from the $[M-7H]^{7-}$ precursor in Figure 4 19
Supporting Figure S16D Fragments from the $[M-8H]^{8-}$ precursor ion (Supporting Table S10), with the sequence coverage overlaid on top. 20
Supporting Figure S16E 2DEDD-MS vertical line, which corresponds to a W_8^{3-} fragment from the autocorrelation line at m/z 922.426746, marked by an asterisk (*)...21
Supporting Figure S17 Fragmentation efficiency of $[M-8H]^{8-}$ danvatirsen vs. amplitude of the initial evolution pulse (Supporting Figure S2) with a 1 µs pulse length when using A) laser-based dissociation (from IR laser) and B) electron-based dissociation (from a hallow-cathode lamp operating at 22.8 eV) 22
Supporting Figure S18A Extracted 2DUVPD-MS/MS fragments of $[M-7H]^{7-}$ precursor with denoising sane rank 10 of a single scan (top, ~350 peaks) and 8 scan accumulated (bottom, ~520 peaks). 23
Supporting Figure S18B Cleavage diagram of the 2DUVPD-MS/MS fragments of $[M-7H]^{7-}$ after a single scan with denoising. 23

Supporting Figure S18C Cleavage diagram of the 2DUVPD-MS/MS fragments of $[M-7H]^{7-}$ after 8 scan accumulated with denoising. 23
Supporting Figure S19A Extracted 2DUVPD-MS/MS fragments of $[M-7H]^{7-}$ precursor without denoising of a single scan (top, ~460 peaks) and 8 scan accumulated (bottom, ~700 peaks)..........24
Supporting Figure S19B Cleavage diagram of the 2DUVPD-MS/MS fragments of $[M-7H]^{7-}$ after a single scan with denoising. 24
Supporting Figure S19C Cleavage diagram of the 2DUVPD-MS/MS fragments of $[M-7H]^{7-}$ after 8 scan accumulated with denoising. 24
Section 2: Simulation of 2DMS..........25
Supporting Figure S20 An ideal transient would have a steady decay in the A) x-dimension, typical of a 1DMS. Transient in B) y-dimension would remain constant assuming the spray is consistent and there is no electrostatic buildup occurring within the source or ion optics. 25
Supporting Figure S21 Transient in A) x-dimension and B) y-dimension with added noise (~ 20%), ideally there would be little to no noise. 25
Supporting Figure S22 Transient in A) x-dimension and B) y-dimension with 8-fold increase in noise compared to Supporting Figure S22, emulating the spectra of low intensity peaks. 26
Supporting Figure S23 A) Sum of transients generated from 2DMS (using noise threshold defined in Supporting Figure S23) and the B) Contour plot after 2DFFT, subsequent C) summation of Fourier Transformed spectra with the respective S/N calculation. Signal was determined by the maximum signal and noise adjacent to the peak was averaged..........27
Supporting Figure S24 Equivalent spectra as S24 but with twice the number of rows (N = 64)..........28
Supporting Figure S25 Equivalent spectra as S24 but with four times the number of rows (N = 128)..........29
Supporting Figure S26 Equivalent spectra as S24 but with eight times the number of rows (N = 256)..........30
Supporting Figure S27 Zoom region of 8 scan accumulated 2DMS of danvatirsen, the presence of two distinct precursors is evident, as indicated by the presence of two intense contours—one corresponding to the $[M-7H]^{7-}$ precursor and the other to a PO impurity...........31
Python code for simulation: 32
Section 3: Code for generating oligonucleotide mass list. 34
Supporting Figure S27 Define the inputs for the oligonucleotide mass calculator by customising the elemental formula for the coloured regions. 34
Section 4: Tables of peak lists..........35
Table S1 showing peak list, signal-to-noise ratio (S/N), elemental composition, and possible assignments with mass errors (ppm) of the $[M-8H]^{8-}$ MALAT-1 by EDD MS/MS, internal fragments marked by character (J), base losses are shown inside the parenthesis. 35
Table S2 showing peak list, signal-to-noise ratio (S/N), elemental composition, and possible assignments with mass errors (ppm) of the $[M-8H]^{8-}$ MALAT-1 by CID MS/MS, internal fragments marked by character (J), base losses are shown inside the parenthesis. 38

Table S3 showing peak list, signal-to-noise ratio (S/N), elemental composition, and possible assignments with mass errors (ppm) of the $[M-8H]^{8-}$ MALAT-1 by IRMPD MS/MS, internal fragments marked by character (J), base losses are shown inside the parenthesis. 44
Table S4 showing peak list, signal-to-noise ratio (S/N), elemental composition, and possible assignments with mass errors (ppm) of the $[M-8H]^{8-}$ MALAT-1 by UVPD MS/MS, internal fragments marked by character (J), base losses are shown inside the parenthesis. 50
Table S5 showing peak list, signal-to-noise ratio (S/N), elemental composition, and possible assignments with mass errors (ppm) of the $[M-8H]^{8-}$ danvatirsen by EDD MS/MS, internal fragments marked by character (J), base losses are shown inside the parenthesis. 56
Table S6 showing peak list, signal-to-noise ratio (S/N), elemental composition, and possible assignments with mass errors (ppm) of the $[M-8H]^{8-}$ danvatirsen by CID MS/MS, internal fragments marked by character (J), base losses are shown inside the parenthesis. 61
Table S7 showing peak list, signal-to-noise ratio (S/N), elemental composition, and possible assignments with mass errors (ppm) of the $[M-8H]^{8-}$ danvatirsen by IRMPD MS/MS, internal fragments marked by character (J), base losses are shown inside the parenthesis. 67
Table S8 showing peak list, signal-to-noise ratio (S/N), elemental composition, and possible assignments with mass errors (ppm) of the $[M-8H]^{8-}$ danvatirsen by UVPD MS/MS, internal fragments marked by character (J), base losses are shown inside the parenthesis. 72
Table S9 showing peak list, signal-to-noise ratio (S/N), elemental composition, and possible assignments with mass errors (ppm) of the autocorrelation line of danvatirsen by 2D-EDD-MS...79
Table S10 showing peak list, signal-to-noise ratio (S/N), elemental composition, and possible assignments with mass errors (ppm) of the $[M-8H]^{8-}$ danvatirsen precursor ion by 2D-EDD-MS. 85
Table S11 showing peak list, signal-to-noise ratio (S/N), elemental composition, and possible assignments with mass errors (ppm) of the $[M-7H]^{7-}$ danvatirsen precursor ion by 2D-EDD-MS. 87
Table S12 showing peak list, signal-to-noise ratio (S/N), elemental composition, and possible assignments with mass errors (ppm) of the $[M-6H]^{6-}$ danvatirsen precursor ion by 2D-EDD-MS. 88
Table S13 showing peak list, intensity, elemental composition, and possible assignments with mass errors (ppm) of danvatirsen using 2D-UVPD-MS of one scan denoised (sane rank 10) $[M-8H]^{8-}$ precursor ion (note, noise peaks were included to highlight the effect of denoising)..90
Table S14 showing peak list, intensity, elemental composition, and possible assignments with mass errors (ppm) of danvatirsen using 2D-UVPD-MS of one scan denoised (sane rank 10) $[M-7H]^{7-}$ precursor ion (note, noise peaks were included to highlight the effect of denoising)..95
Table S15 showing peak list, intensity, elemental composition, and possible assignments with mass errors (ppm) of danvatirsen using 2D-UVPD-MS of one scan and no denoising of $[M-8H]^{8-}$ precursor ion (note, noise peaks were included to highlight the effect of denoising). 102

338.157835 102
Table S16 showing peak list, intensity, elemental composition, and possible assignments with mass errors (ppm) of danvatirsen using 2D-UVPD-MS of one scan and no denoising of $[M-7H]^{7-}$ precursor ion (note, noise peaks were included to highlight the effect of denoising). 111
Table S17 showing peak list, intensity, elemental composition, and possible assignments with mass errors (ppm) of danvatirsen using 2D-UVPD-MS of eight scans accumulated denoised (sane rank 10) $[M-8H]^{8-}$ precursor ion (note, noise peaks were included to highlight the effect of denoising).................................120
Table S18 showing peak list, intensity, elemental composition, and possible assignments with mass errors (ppm) of danvatirsen using 2D-UVPD-MS of eight scans accumulated denoised (sane rank 10) $[M-7H]^{7-}$ precursor ion (note, noise peaks were included to highlight the effect of denoising).................................132
Table S19 showing peak list, intensity, elemental composition, and possible assignments with mass errors (ppm) of danvatirsen using 2D-UVPD-MS of eight scans accumulated and no denoising of $[M-8H]^{8-}$ precursor ion (note, noise peaks were included to highlight the effect of denoising). 142
Table S20 showing peak list, intensity, elemental composition, and possible assignments with mass errors (ppm) of danvatirsen using 2D-UVPD-MS of eight scans accumulated and no denoising of [M-7H]7- precursor ion (note, noise peaks were included to highlight the effect of denoising). 152
Supporting References...166

Section 1: Supporting Experimental Section

The denoising algorithm of sane (rank = 10) was used, which is a memory efficient development of the proposed uncoiled random QR denoising (urQRd) algorithm,[1] and is publicly available from SPIKE.[2] To extract the signal from the noise, the original dataset is truncated by randomly sampling the original matrix, breaking up any patterns that could contribute to the noise. This truncated dataset is then subjected to QR decomposition using a specified rank, allowing for the separation of signal and noise. After QR decomposition is applied, the resulting orthogonal matrix (Q) is multiplied by the truncated data set to reconstruct the original data with the noise removed.[1] Subsequently, QR decomposition is iterated to successive rows (t_1 rows or scan lines) until the entire matrix is factorised; this iterative approach allows for parallel processing of rows. Furthermore, the rank used in QR, which is the number of linearly independent rows/columns in the matrix used for the reconstruction of the original dataset, was previously shown to affect the quality of denoising by Chiron *et al.*, where an optimised signal-to-noise ratio (SNR) gain was observed by selecting a rank (rank is doubled for urQRd) equivalent to the number of expected precursor ions observed in the *y*-axis.[1] Due to the number of precursor ions observed in Section 3.5, a logical rank of 10 was used for the denoising of danvatirsen.

Section 2: Supporting Figures

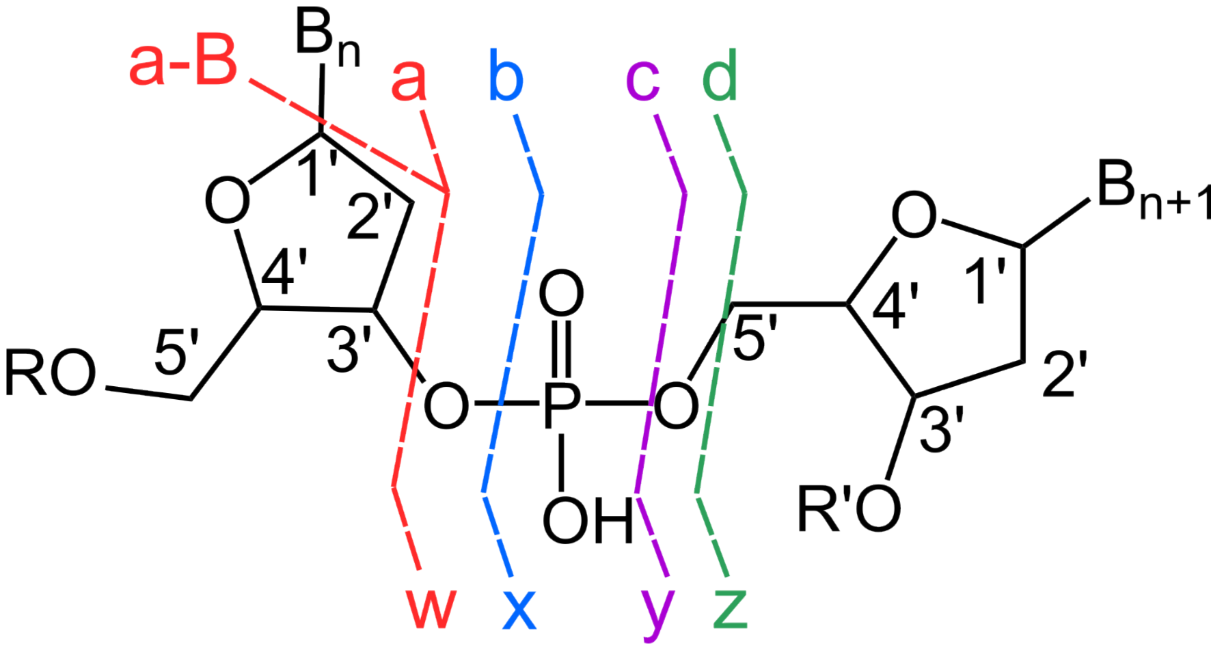

Supporting Figure S1 Nomenclature for Oligonucleotide fragmentation based on Mcluckey cleavages based on Scheme 1, where the locked nucleic acid forms a bridge between the 2 carbon and 4 carbon.

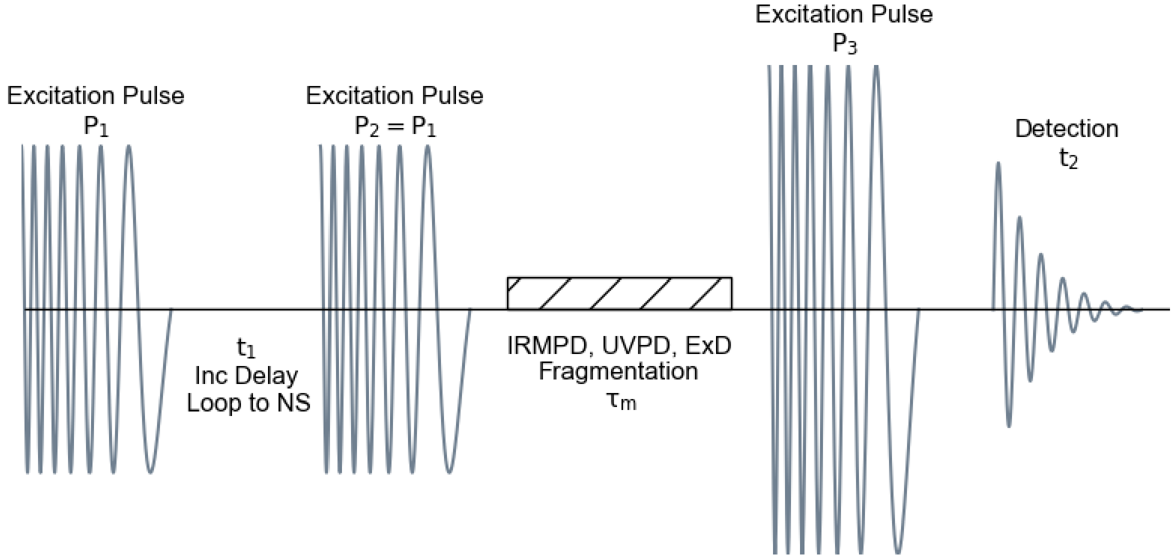

Supporting Figure S2 2D Pulse sequence, where the increment delay was fixed by the number of scans and subsequently averaged after accumulation.

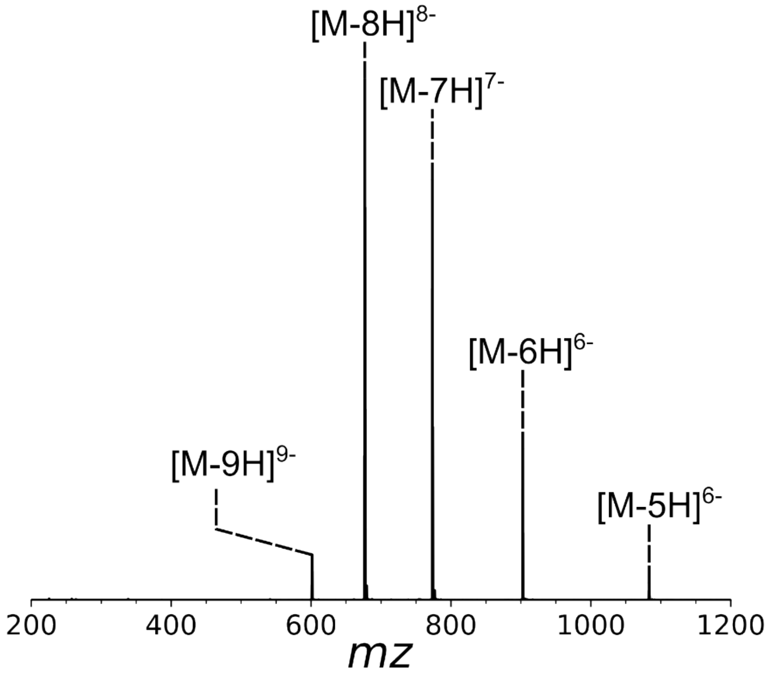

Supporting Figure S3 MS spectrum of danvatirsen after desalting using HyperSep™ C18 plates.

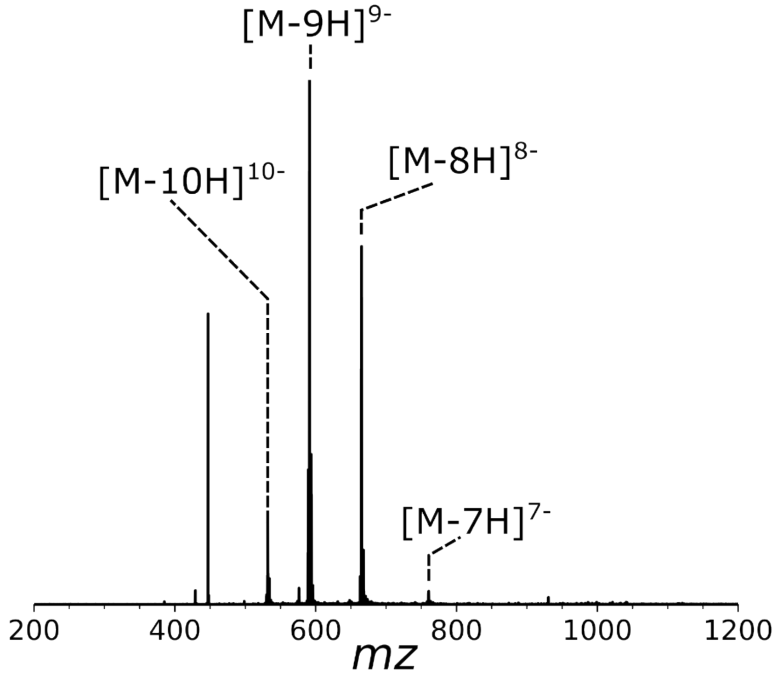

Supporting Figure S4 MS spectrum of MALAT-1 after desalting HyperSep™ C18 plates.

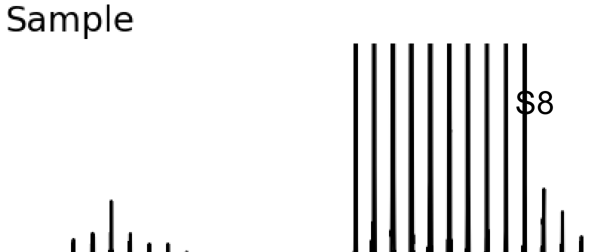

Supporting Figure S5 MS spectrum of danvatirsen after desalting HyperSep™ C18 plates highlighting the [M-8H]$^{8-}$ and a phosphate impurity in the phosphorothioate backbone, with the simulated spectrum (bottom).

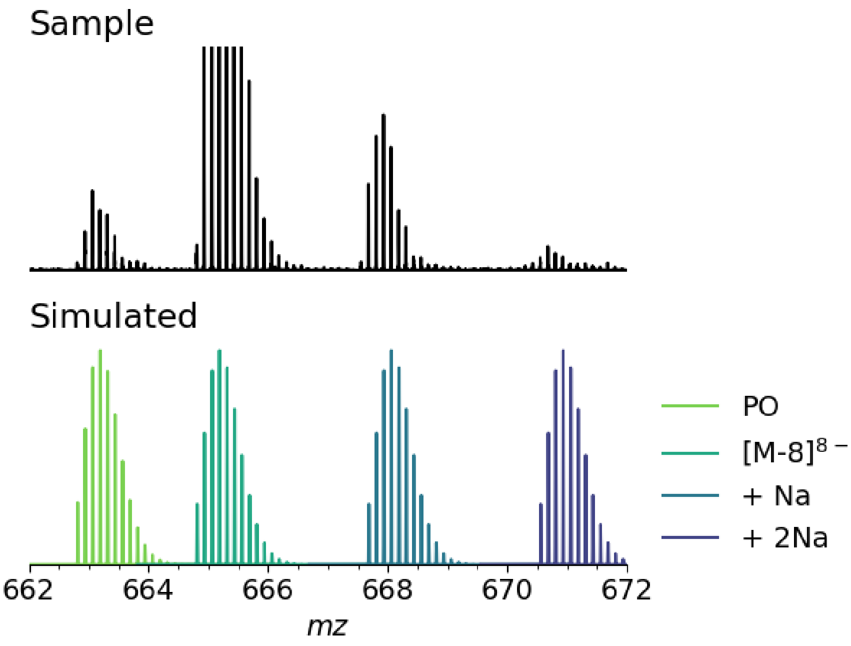

Supporting Figure S6 MS spectrum of MALAT-1 after desalting HyperSep™ C18 plates highlighting the [M-8H]$^{8-}$, a phosphate impurity in the phosphorothioate backbone, sodium and disodium adducts, with the simulated spectrum (bottom).

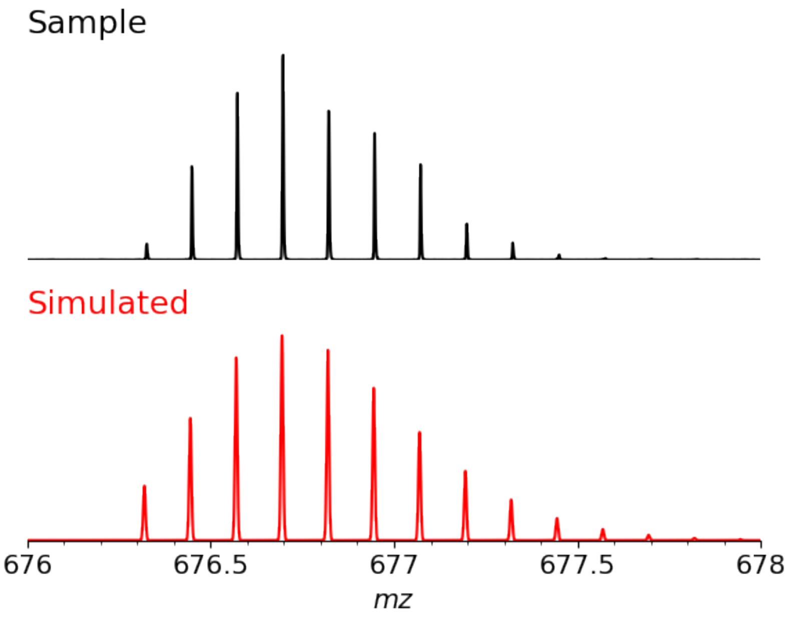

Supporting Figure S7 MS spectrum of danvatirsen, $[M-8H]^{8-}$, with the simulated spectrum (red).

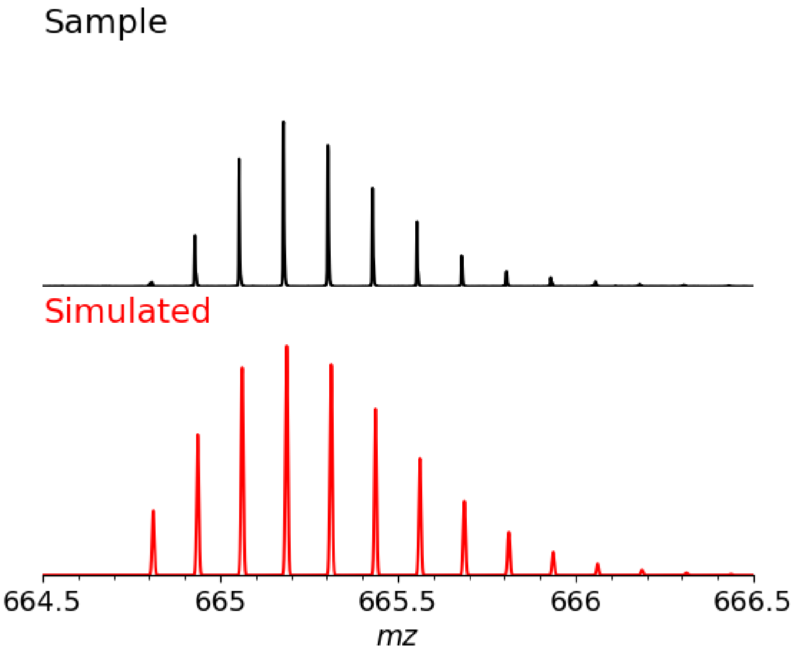

Supporting Figure S8 MS spectrum of MALAT-1, $[M-8H]^{8-}$, with the simulated spectrum (red).

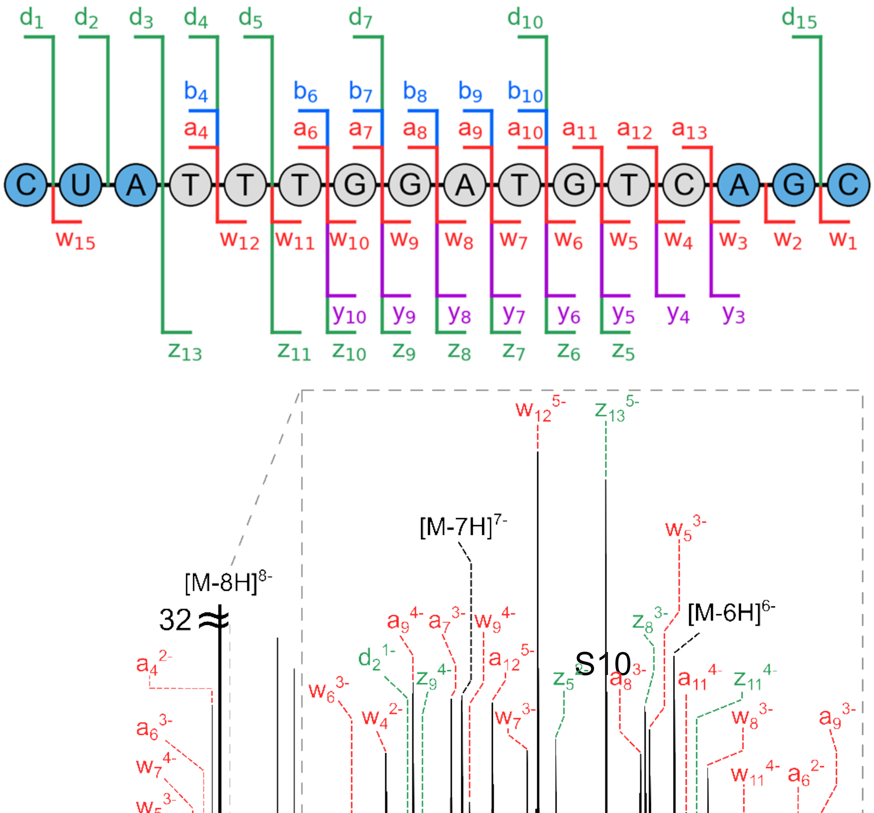

Supporting Figure S9 EDD-MS/MS spectrum of danvatirsen, [M-8H]$^{8-}$, at 22.8 eV bias for 0.5 s irradiation time, with cleavage diagrams overlayed on top.

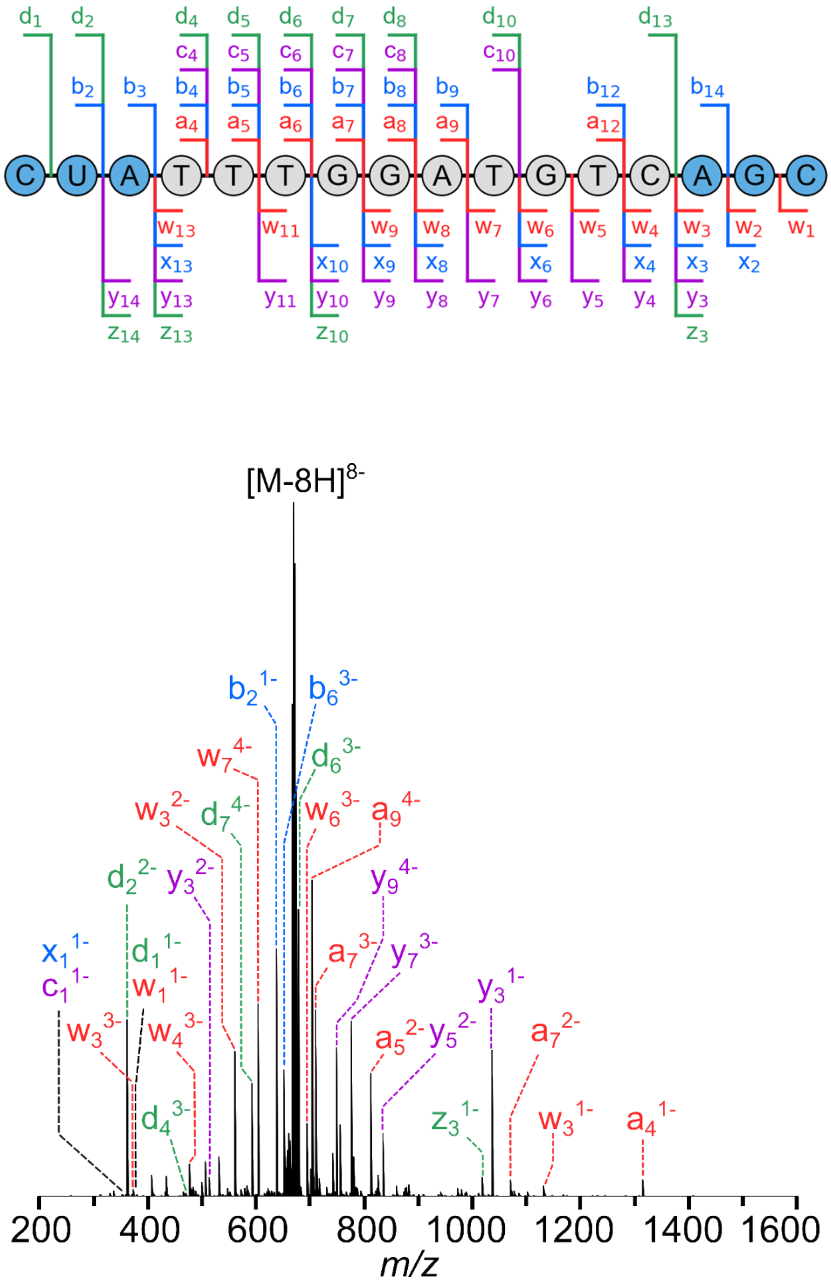

Supporting Figure S10 CID-MS/MS spectrum of danvatirsen, [M-8H]$^{8-}$, at -9.2 V, with cleavage diagrams overlayed on top.

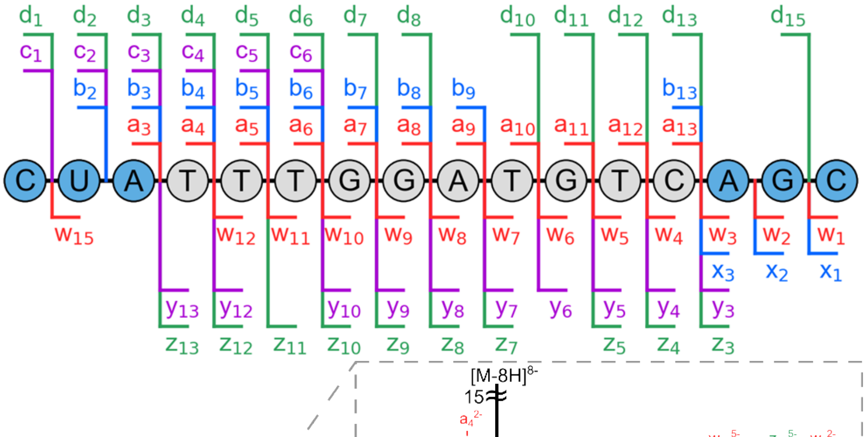

Supporting Figure S11 UVPD-MS/MS spectrum of danvatirsen, $[M-8H]^{8-}$, using 2 shots of 3.4 mJ laser power, with cleavage diagrams overlayed on top.

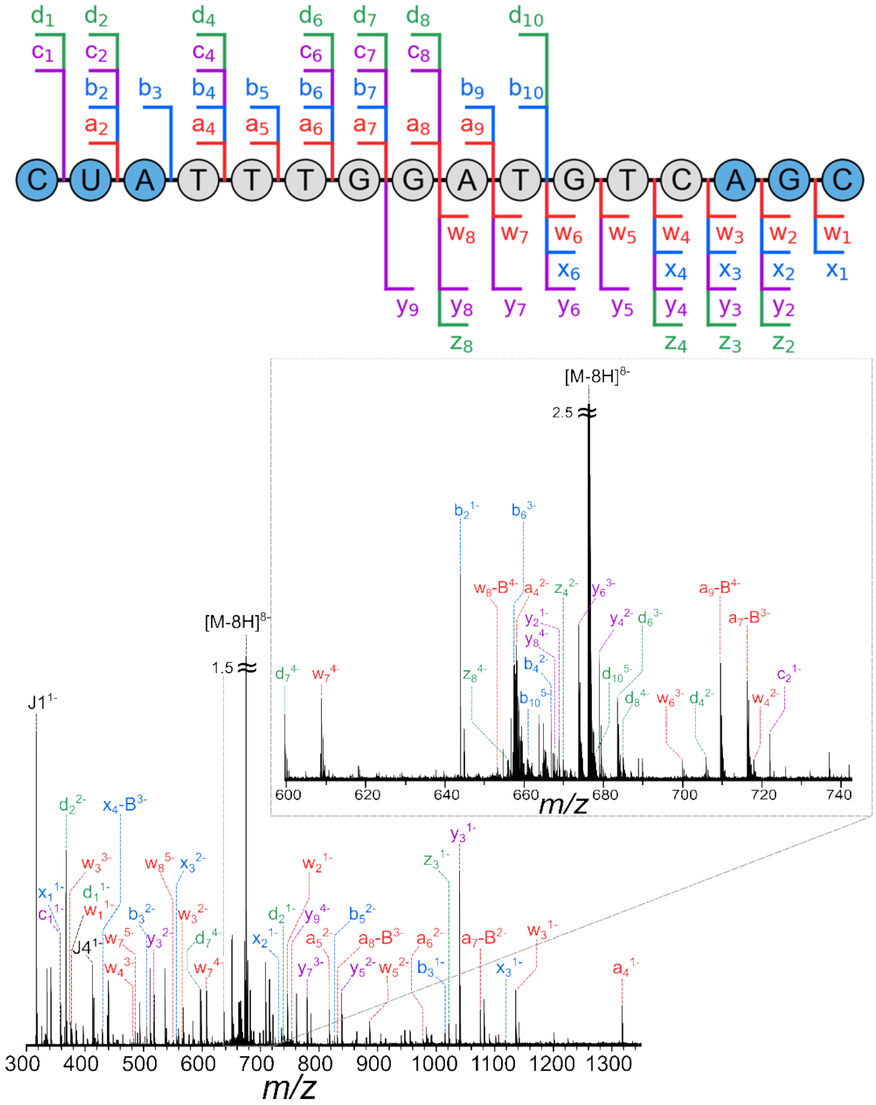

Supporting Figure S12 IRMPD-MS/MS spectrum of danvatirsen, $[M-8H]^{8-}$, at 27.5 % laser power for 0.06 s irradiation time, with cleavage diagrams overlayed on top.

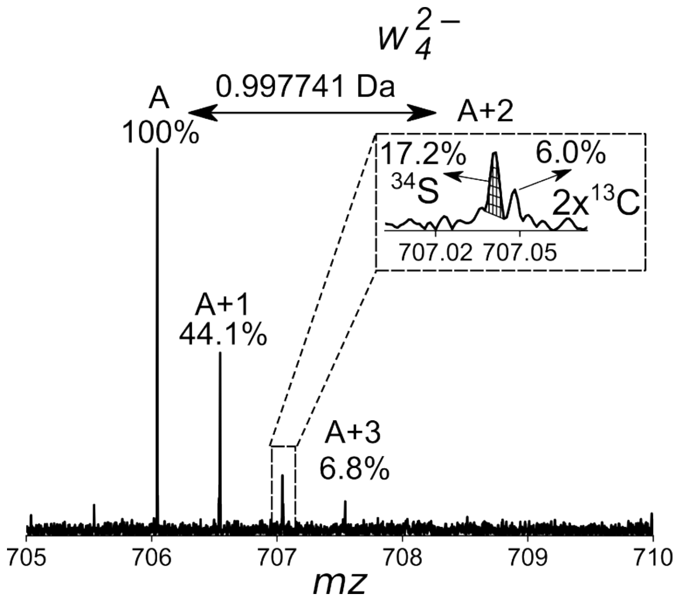

Supporting Figure S13 Isotope pattern of w_4^{2-} ion ($C_{43}H_{54}N_{16}O_{23}P_4S_4$) of MALAT-1, obtained from UVPD-MS/MS (Supporting Table S4), where the peak areas (%) are overlaid on top. The A+2 peak corresponds to a molecule with one ^{34}S atom (shaded) and two ^{13}C atoms, and its fine isotopic pattern allows for the resolution of these isotopes. This observation confirms the presence of four sulfur atoms, as expected for a phosphorothioate backbone.

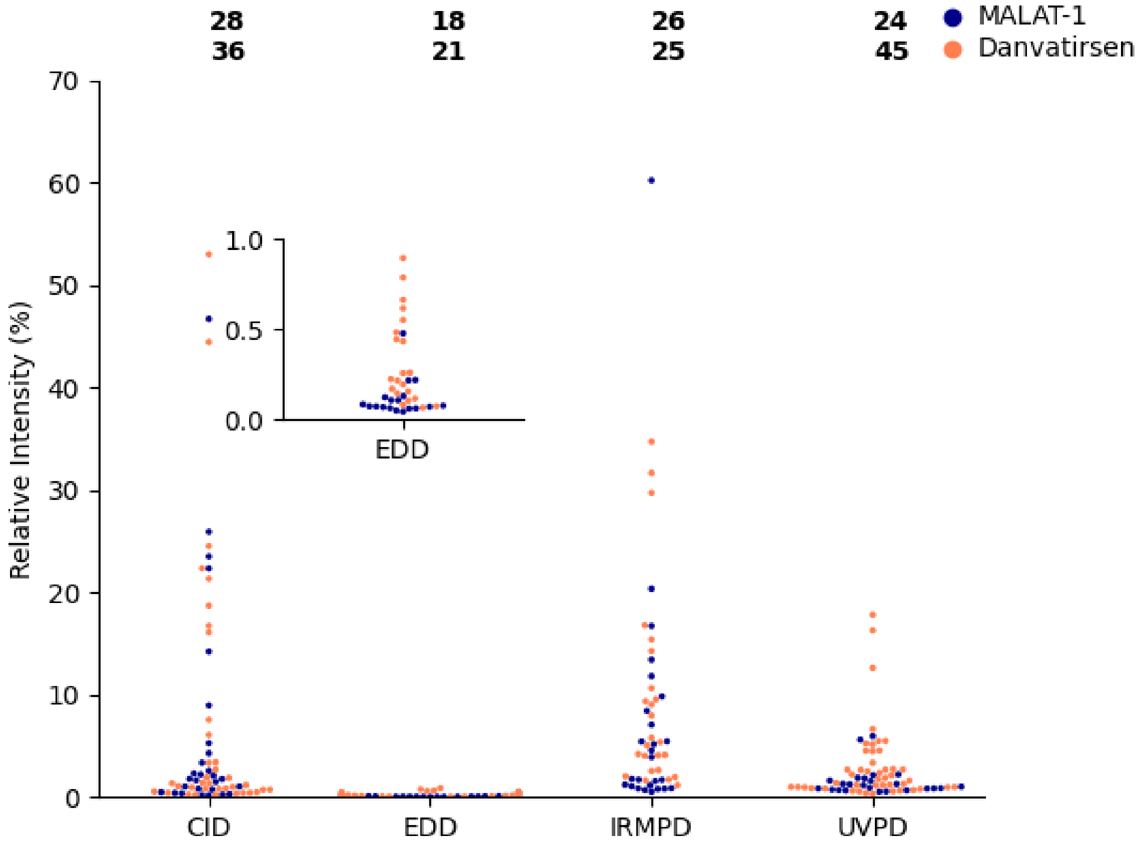

Supporting Figure S14 Beeswarm plot of the relative intensities for each McLuckey cleavage and its analogues of MALAT-1 (Navy) and danvatirsen (Orange) produced by Tandem MS using CID, EDD, IRMPD, and UVPD. Each point represents a single peak, which is evenly spaced in the *x*-axis to prevent overlap, where 20-50% of the peaks are plotted due to the large number of peaks. Zoom-in on EDD is provided due to low abundance of peaks. Total number of peaks is overlayed on top, see Table 1.

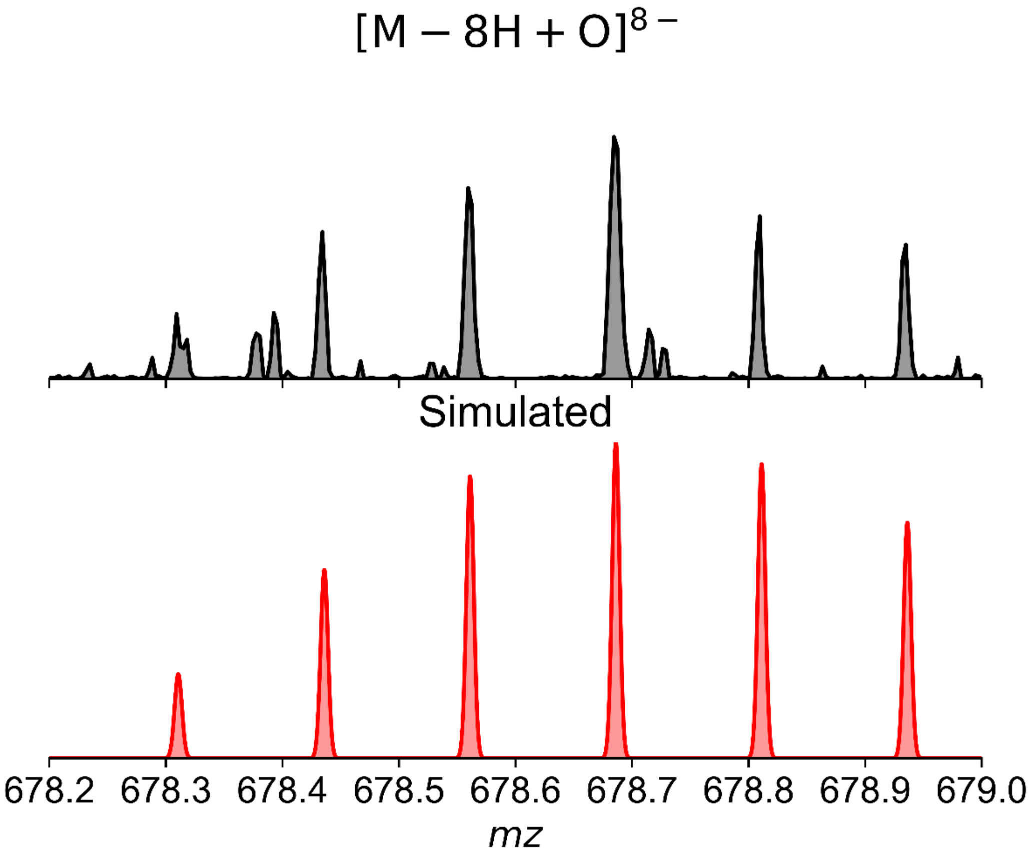

Supporting Figure S15 The presence of an additional oxygen in danvatirsen indicates the occurrence of oxidative degradation. The observed low intensity suggests that the precursor is inherently unstable and undergoes degradation upon exposure to oxygen.

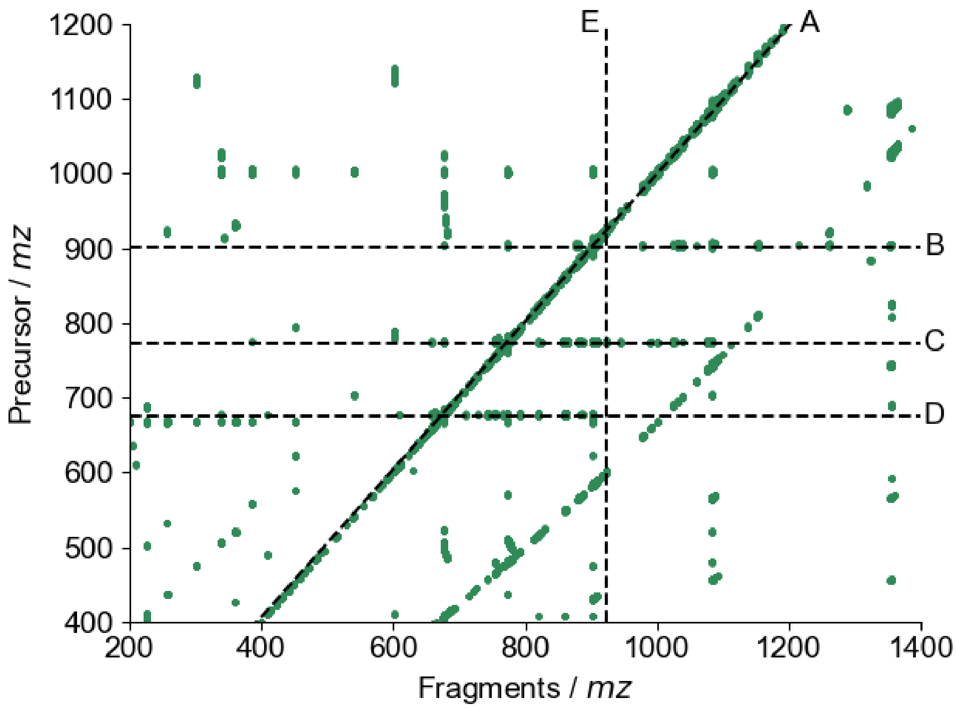

Supporting Figure S16 2D-EDD-MS of danvatirsen performed with 22.8 eV bias for 0.5 s irradiation time (1M x-axis by 2048 y-axis data points)

Supporting Figure S16A Autocorrelation line (equivalent to 1DMS, mass list available in Supporting Table S9)

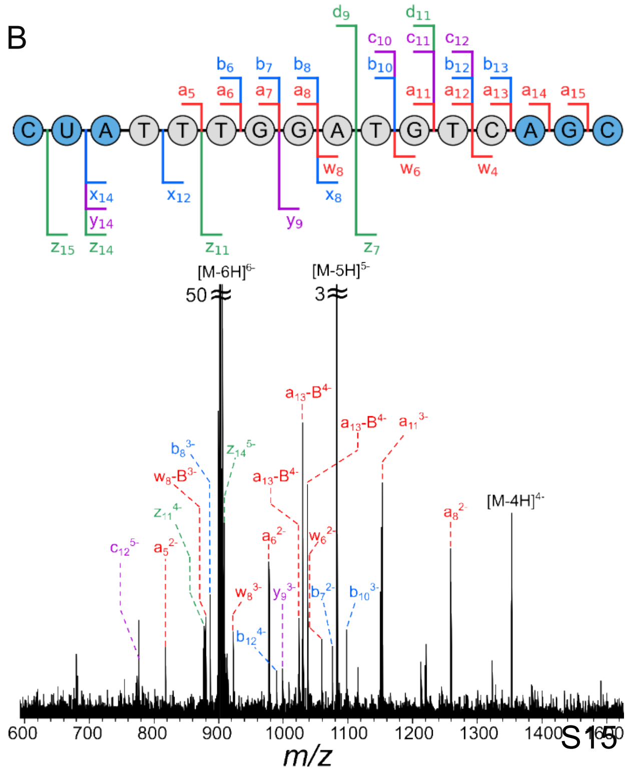

Supporting Figure S16B 2DEDD-MS Fragment line extracted from the [M-6H]$^{6-}$ precursor in Figure 4

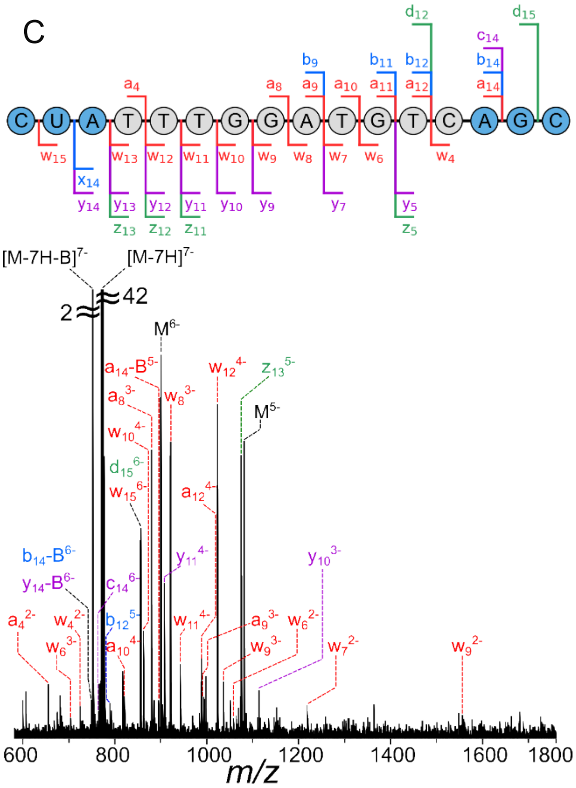

Supporting Figure S16C 2DEDD-MS Fragment line extracted from the [M-7H]$^{7-}$ precursor in Figure 4

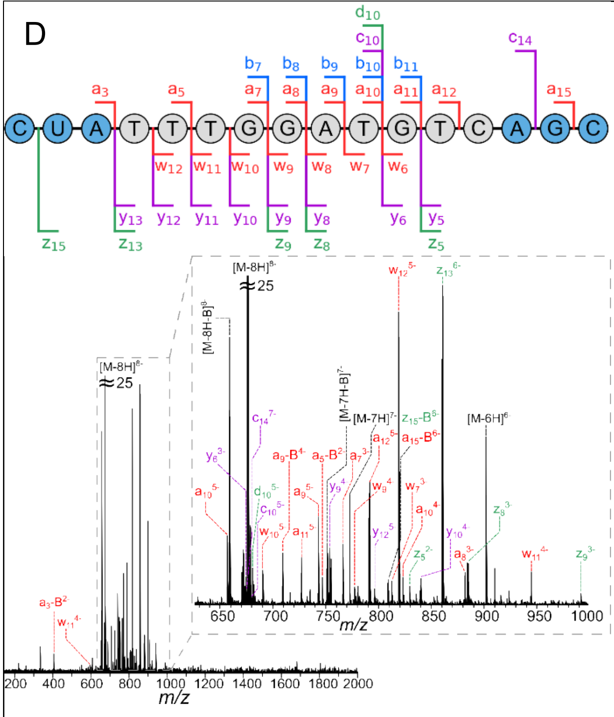

Supporting Figure S16D Fragments from the [M-8H]$^{8-}$ precursor ion (Supporting Table S10), with the sequence coverage overlaid on top.

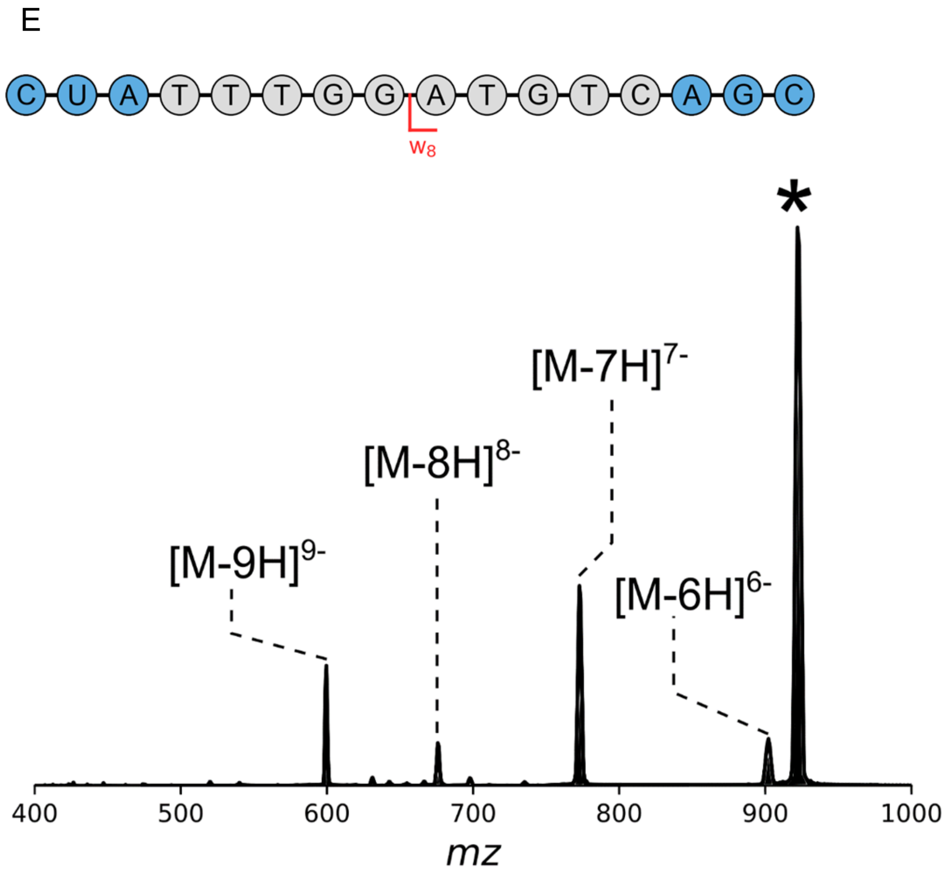

Supporting Figure S16E 2DEDD-MS vertical line, which corresponds to a W_8^{3-} fragment from the autocorrelation line at m/z 922.426746, marked by an asterisk (*).

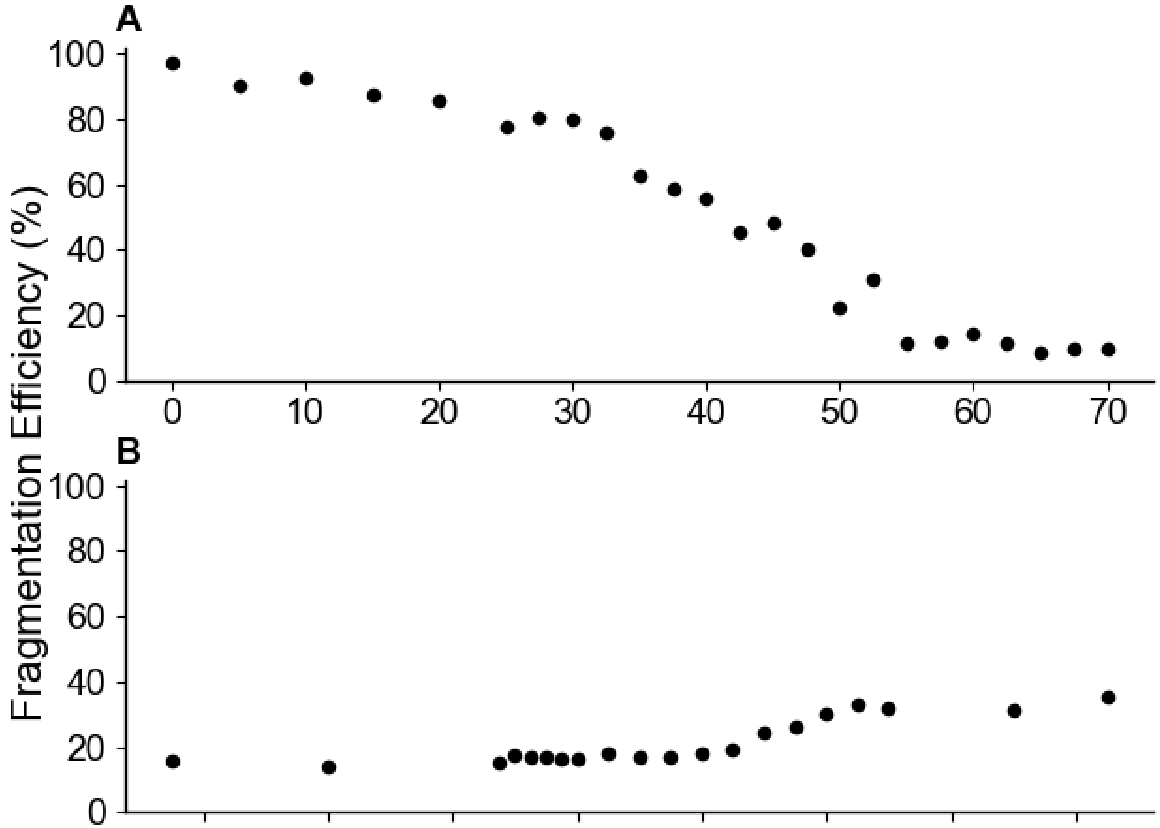

Supporting Figure S17 Fragmentation efficiency of $[M-8H]^{8-}$ danvatirsen vs. amplitude of the initial evolution pulse (Supporting Figure S2) with a 1 µs pulse length when using A) laser-based dissociation (from IR laser) and B) electron-based dissociation (from a hallow-cathode lamp operating at 22.8 eV)

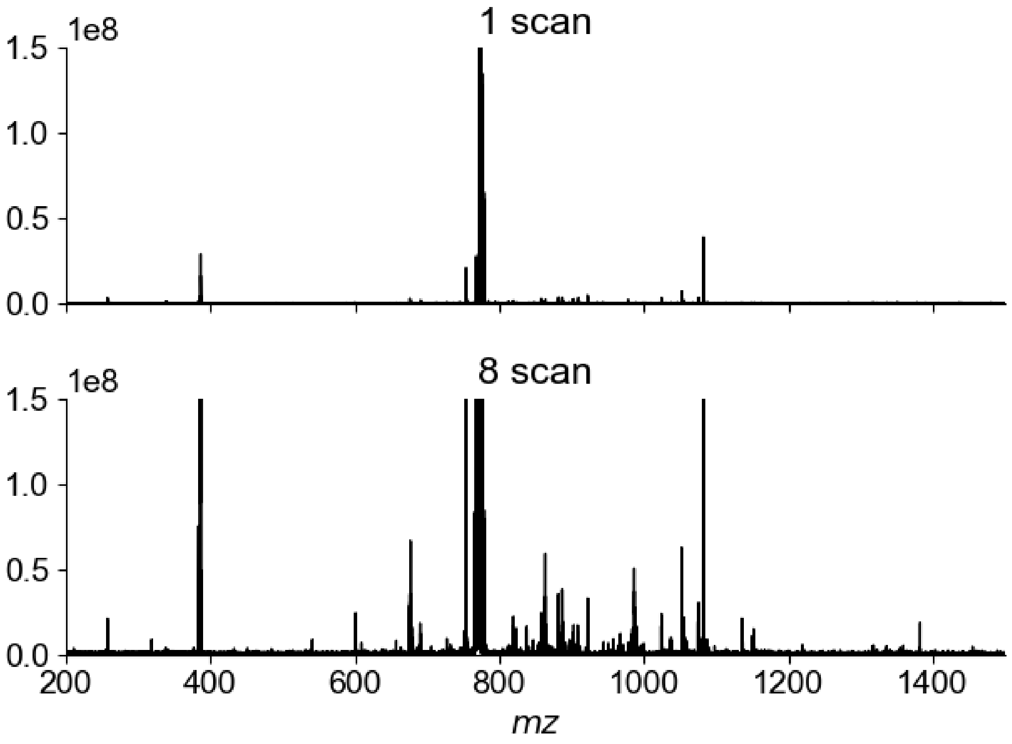

Supporting Figure S18A Extracted 2DUVPD-MS/MS fragments of $[M-7H]^{7-}$ precursor with denoising sane rank 10 of a single scan (top, ~350 peaks) and 8 scan accumulated (bottom, ~520 peaks).

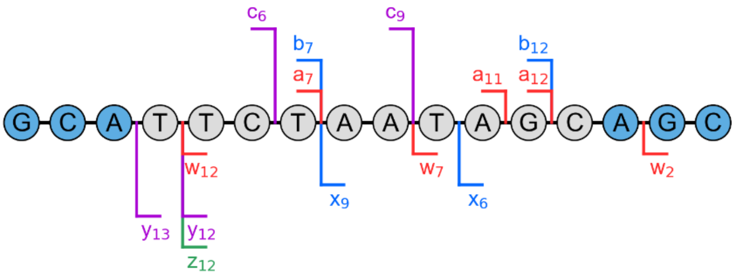

Supporting Figure S18B Cleavage diagram of the 2DUVPD-MS/MS fragments of $[M-7H]^{7-}$ after a single scan with denoising.

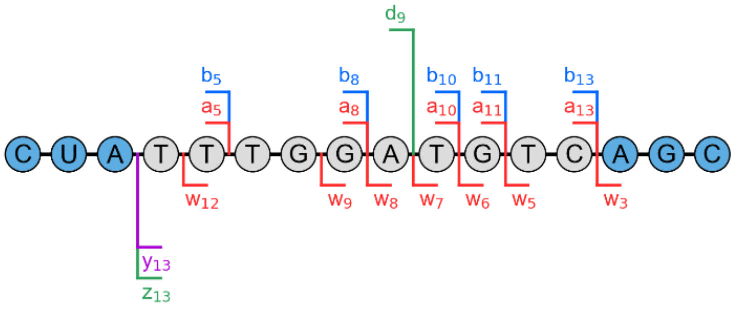

Supporting Figure S18C Cleavage diagram of the 2DUVPD-MS/MS fragments of $[M\text{-}7H]^{7-}$ after 8 scan accumulated with denoising.

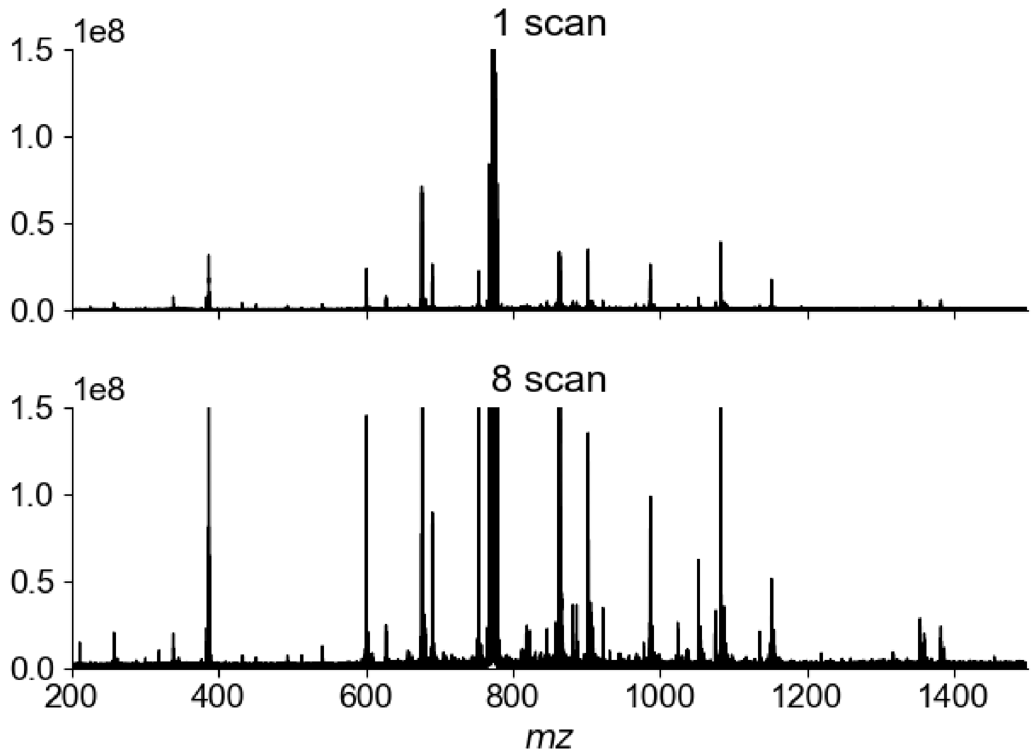

Supporting Figure S19A Extracted 2DUVPD-MS/MS fragments of $[M\text{-}7H]^{7-}$ precursor without denoising of a single scan (top, ~460 peaks) and 8 scan accumulated (bottom, ~700 peaks).

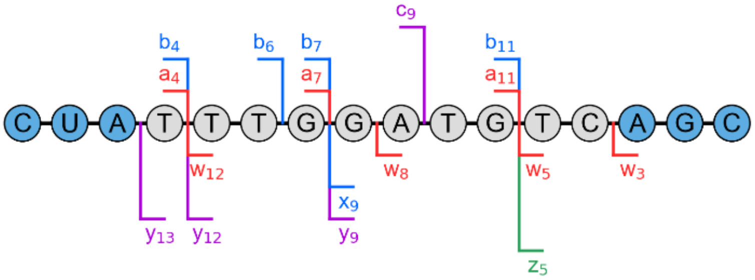

Supporting Figure S19B Cleavage diagram of the 2DUVPD-MS/MS fragments of $[M\text{-}7H]^{7-}$ after a single scan with denoising.

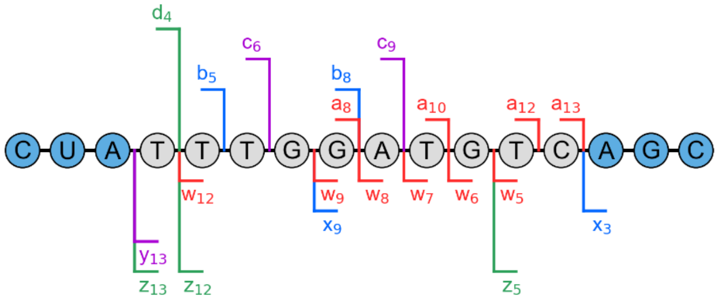

Supporting Figure S19C Cleavage diagram of the 2DUVPD-MS/MS fragments of $[M-7H]^{7-}$ after 8 scan accumulated with denoising.

Section 2: Simulation of 2DMS

Expansion of Section 3.6 in the main text. Figures first show the x- and y- dimension of a 2DMS plot.

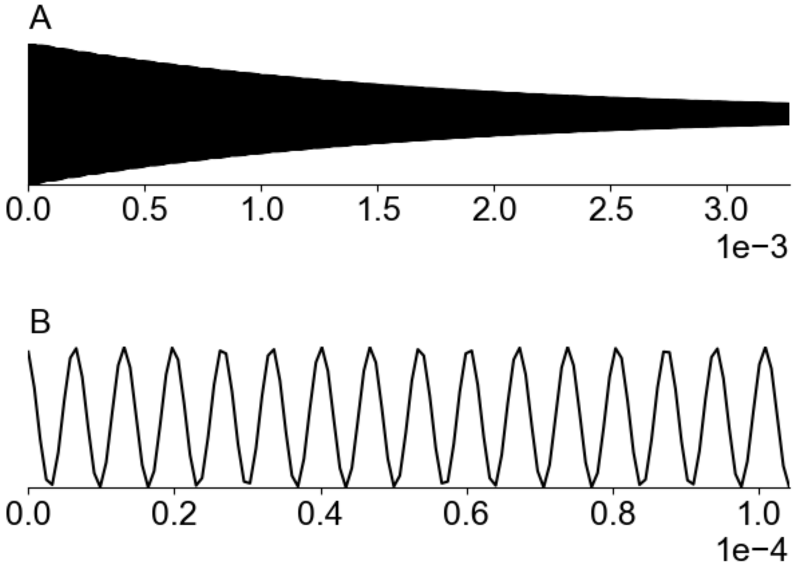

Supporting Figure S20 An ideal transient would have a steady decay in the A) x-dimension, typical of a 1DMS. Transient in B) y-dimension would remain constant assuming the spray is consistent and there is no electrostatic buildup occurring within the source or ion optics.

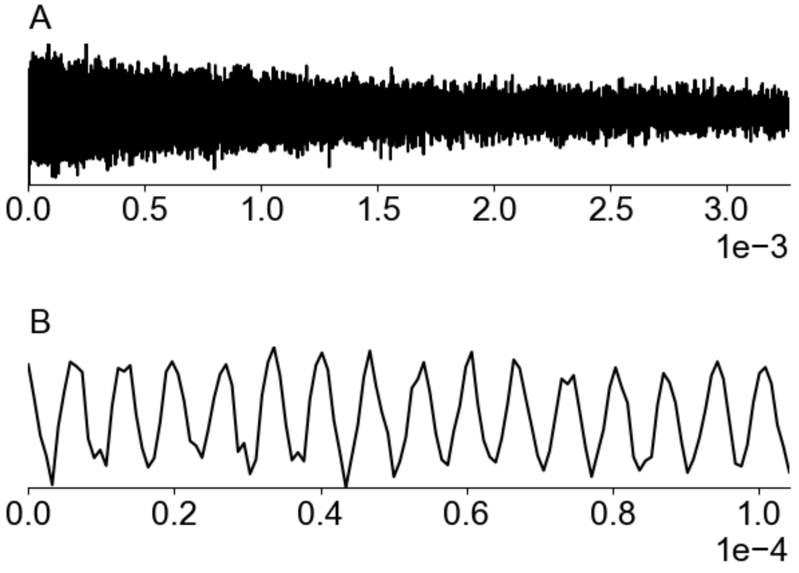

Supporting Figure S21 Transient in A) x-dimension and B) y-dimension with added noise (~ 20%), ideally there would be little to no noise.

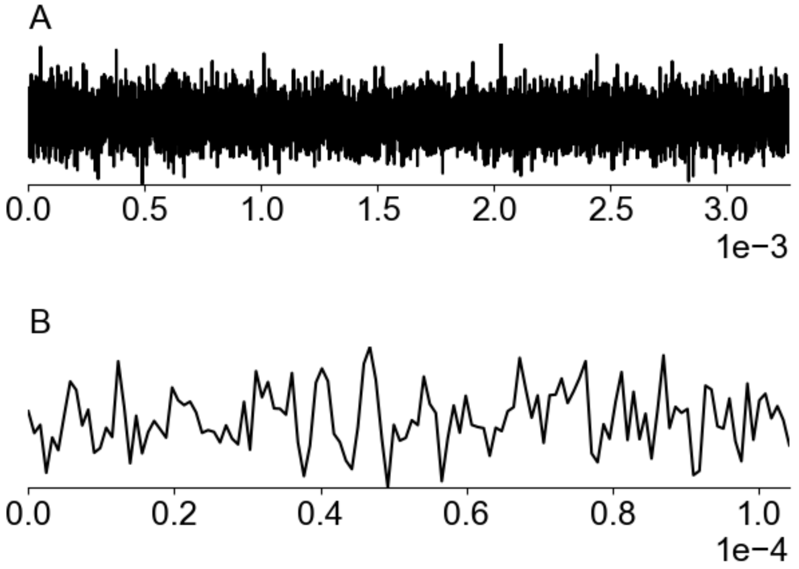

Supporting Figure S22 Transient in A) x-dimension and B) y-dimension with 8-fold increase in noise compared to Supporting Figure S22, emulating the spectra of low intensity peaks.

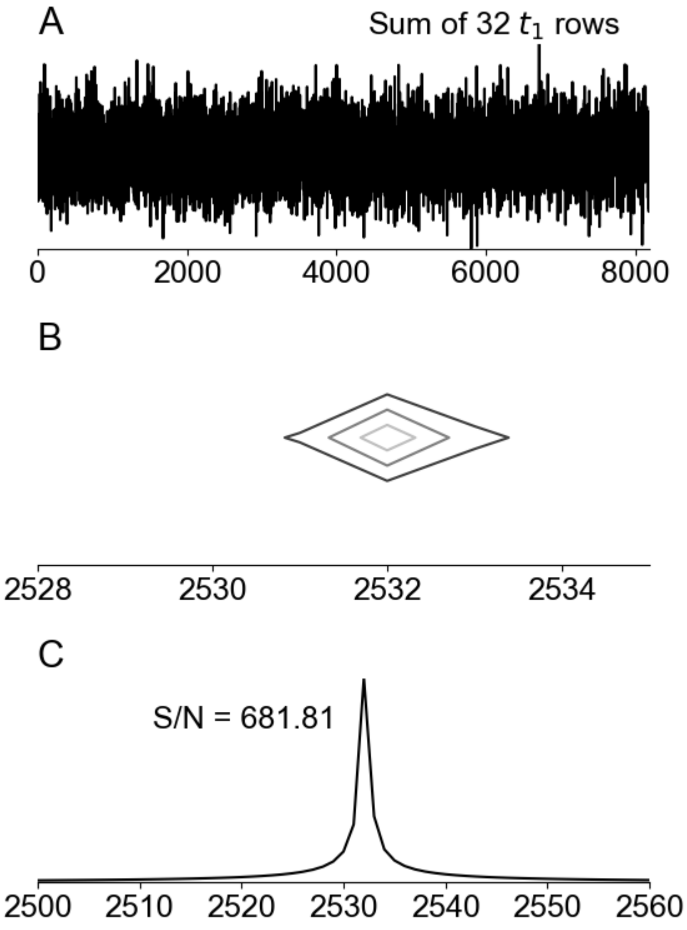

Supporting Figure S23 A) Sum of transients generated from 2DMS (using noise threshold defined in Supporting Figure S23) and the B) Contour plot after 2DFFT, subsequent C) summation of Fourier Transformed spectra with the respective S/N calculation. Signal was determined by the maximum signal and noise adjacent to the peak was averaged.

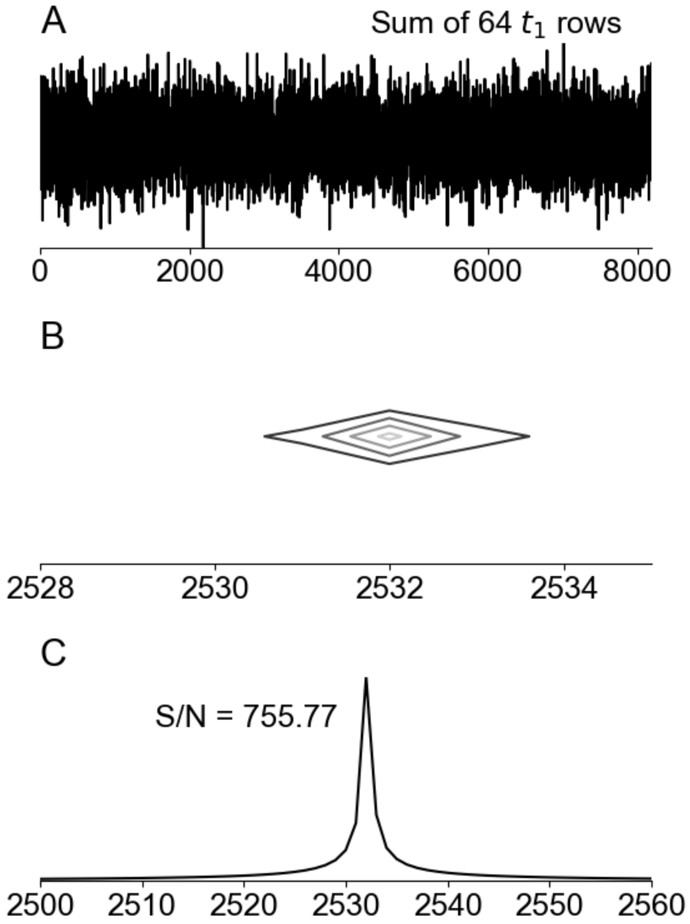

Supporting Figure S24 Equivalent spectra as S24 but with twice the number of rows (*N* = 64)

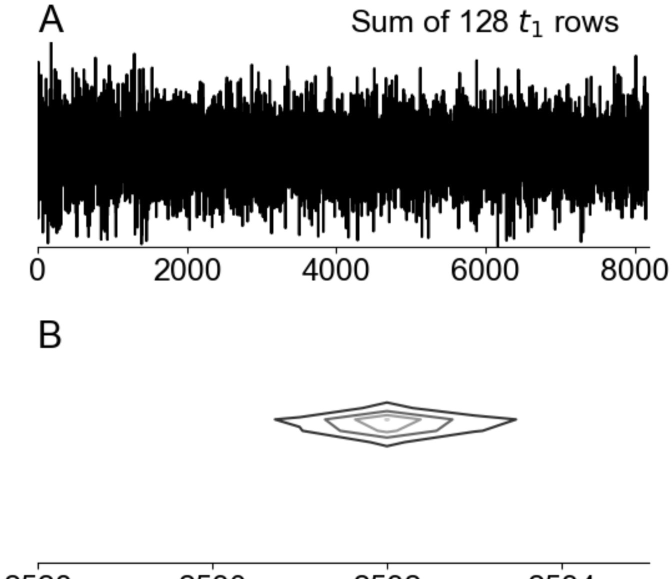

Supporting Figure S25 Equivalent spectra as S24 but with four times the number of rows (*N* = 128)

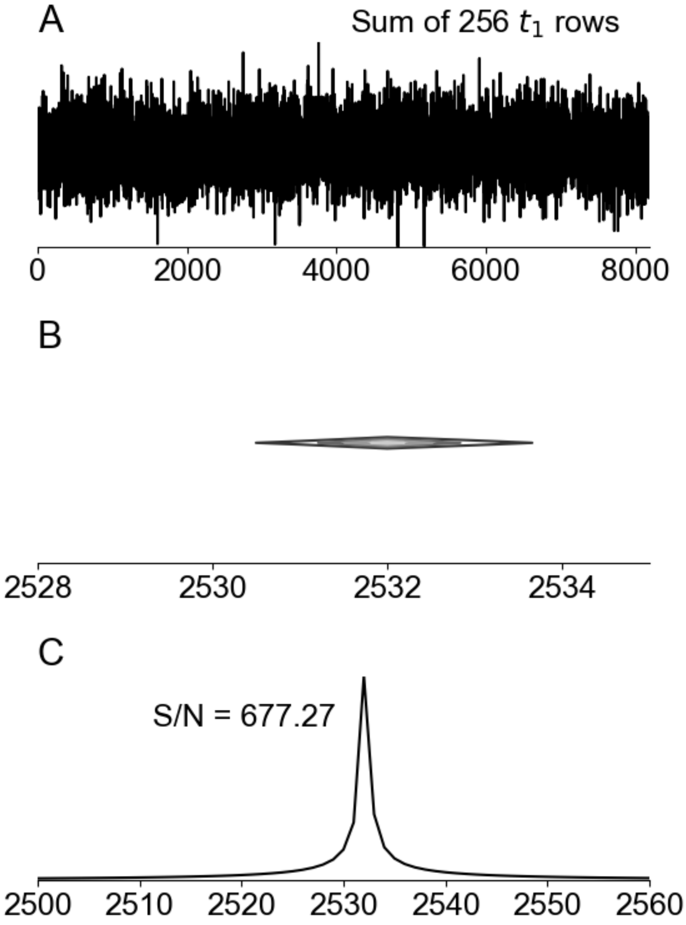

Supporting Figure S26 Equivalent spectra as S24 but with eight times the number of rows (*N* = 256)

By comparing the signal-to-noise ratio (SNR) between Supporting Figures S24 to S26, it demonstrates that acquiring more scan limes (t_1 row) can improve the SNR. However, beyond a certain threshold, the SNR will reach a plateau where further increases in the number of scans will only result in the acquisition of more noise, which is observed in Supporting Figure S26. We therefore propose that taking multiple scans at the peak of SNR, and taking the average will lead to the greatest signal enhancement instead of acquiring more scan lines (t_1 row). Python code for simulating 2DMS is shown in page S32.

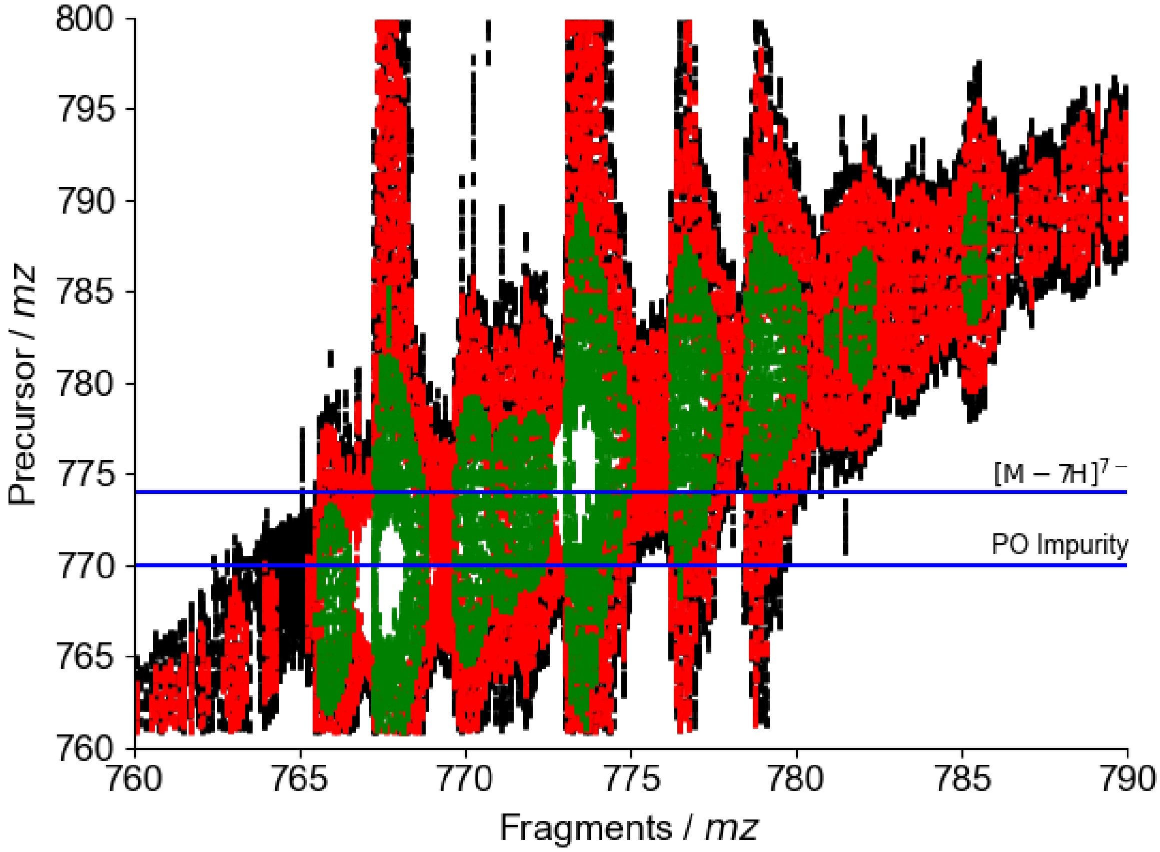

Supporting Figure S27 Zoom region of 8 scan accumulated 2DMS of danvatirsen, the presence of two distinct precursors is evident, as indicated by the presence of two intense contours—one corresponding to the [M-7H]$^{7-}$ precursor and the other to a PO impurity.

Python code for simulation:

```
import numpy as np
import pandas as pd
import matplotlib.pyplot as plt
import sys

lowmass = 147
highmass = 1000
fidres = 8192 # 20=1m, 22=4m, 23=8m
noise_level = 0.2

lowmassy = 300
fidresy = 256

def mz_to_f(data, num_charge = 1):
    # data is freq or mass
    q = 1.60217662e-19 * num_charge
    B = 12 # 12T magnet
    mol = 6.0221409e+23 *1000 #account for kg
    mz_fs = (q * B * mol) / (2 * np.pi * data)
    return mz_fs

def calculate_frequency_domain(nyq, number_data_points):
    #nyq rate freq * data points
    qntpoints = np.arange(0, (number_data_points))
    factor_distancy = (nyq) / (number_data_points)
    frequency_domain = qntpoints * factor_distancy
    return frequency_domain


nyq_rate = mz_to_f(lowmass) # get nyq rate
samp_rate = 2*nyq_rate # sampling rate is 2* nyq rate
transient_length =fidres/samp_rate #num data points / sampling rate =
transient length
nyq_ratey = mz_to_f(lowmassy) # get nyq rate
samp_ratey = 2*nyq_ratey # sampling rate is 2* nyq rate
transient_lengthy = fidresy/samp_ratey

sample_freq_622 = mz_to_f(622) #select a mass e.g. 622 and convert to
frequency
print(nyq rate: %.2f\nfid len: %.2f\nsample freq: %.2f%(nyq_rate,
transient_length,sample_freq_622))
tx = np.linspace(0,transient_length,fidres) #x dimension
ty = np.linspace(0,transient_lengthy,fidresy) #y dimenstion

X,Y = np.meshgrid(tx,ty) #create 2D array of x and y data points

xdim = np.exp(1j * sample_freq_622 * np.pi * X) * np.exp(sample_freq_622*-X
* 0.002) # xdim and damping transient
ydim = np.exp(1j * sample_freq_622 * np.pi * Y) #ydim with no damping added
f =  xdim*ydim

#Add noise in x and y dimension
f_noise = f + (np.random.normal(0,noise_level,size=np.shape(f))) #+ (1j *
np.random.normal(0,noise_level,size=np.shape(f)))

ft = np.fft.rfft2(f) # 2d fft take real spectrum
ft = np.fft.fftshift(ft) #reorder the fft data set so peaks are centered

fig, axes = plt.subplots(3, figsize=(6,8))
```

```
axes[0].plot(np.sum(f_noise,axis=0),c=k) #Sum all the rows and plot the FID
# Annotate the plot
axes[0].plot([],[],  ,  label=Sum of %s $t_{1}$ rows%fidresy)
axes[0].set_title(A, loc=left)
axes[0].set_ylabel(Intensity)
axes[0].legend(frameon=False,loc=best, bbox_to_anchor=(0.2, 0.5, 0.8, 0.8))



data_points_x = len(ft[0])
ft = np.sqrt((np.power(ft.real, 2)) + (np.power(ft.imag, 2)))

#Plot 2D contour plot
axes[1].contour(ft,cmap=gray,levels=4)
axes[1].set_yticks(np.arange(0, 10+1, 2))
axes[1].set_ylim([int(fidresy)/1.8,int(fidresy)/1.5])
axes[1].set_xlim([2528,2535]) #select range to see peak
axes[1].set_title(B, loc=left)
axes[1].set_ylabel(Index / y)

ft1d = np.sum(ft[:, :], axis=0) #Sum all FTs
sig = np.max(ft1d) # select the max signal
noise = np.mean(ft1d[2000:2400]) # mean noise (magnitude mode) of noise
adjacent to signal

SN = sig/noise
print(Sig = %.2f \nNoise  = %.2f\nSN=%.2f%(sig,noise,sig/noise))

# Plot the summated FTs
axes[2].plot(ft1d,c=k)
axes[2].plot([],[],  , label=S/N = %.2f%(SN))
axes[2].set_xlim([2500,2560]) #adjust range to see peak
axes[2].legend(frameon=False)
axes[2].set_title(C, loc=left)
axes[2].set_ylabel(Intensity)

plt.show()
```

Section 3: Code for generating oligonucleotide mass list.

Available at https://github.com/MKRahman97/Oligonucleotide_mass_calculator.

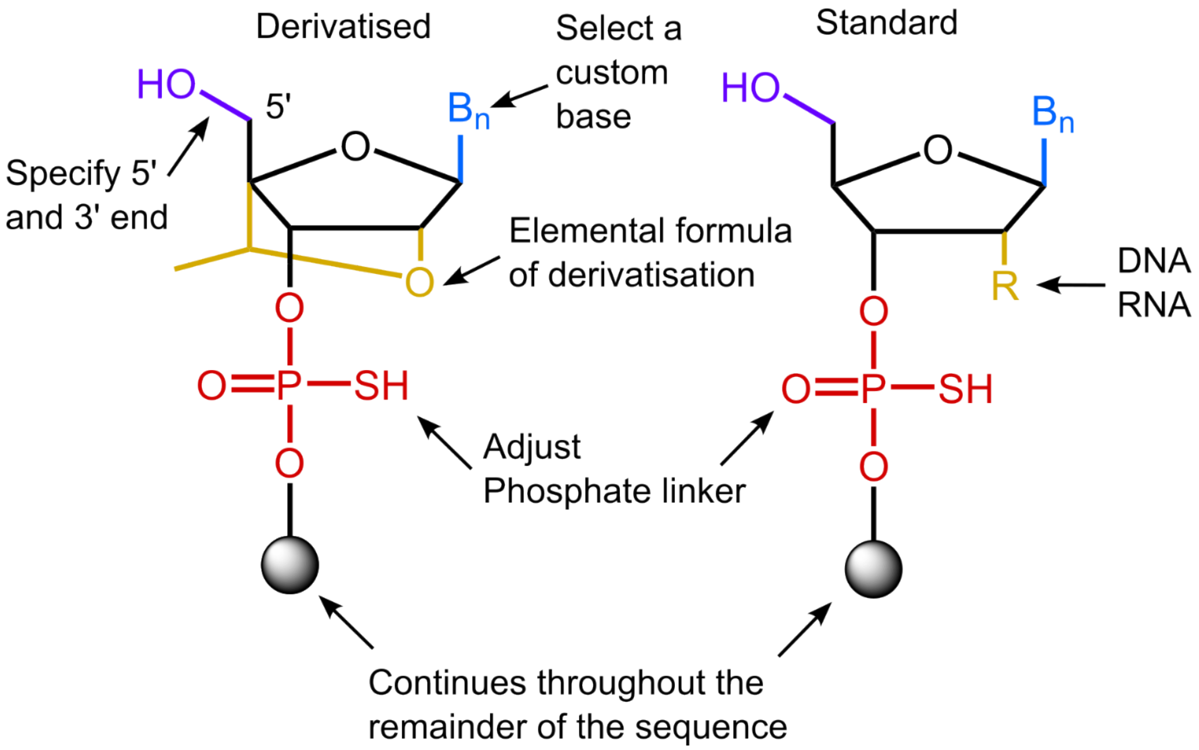

Supporting Figure S27 Define the inputs for the oligonucleotide mass calculator by customising the elemental formula for the coloured regions.

Section 4: Tables of peak lists

Table S1 showing peak list, signal-to-noise ratio (S/N), elemental composition, and possible assignments with mass errors (ppm) of the $[M\text{-}8H]^{8-}$ MALAT-1 by EDD MS/MS, internal fragments marked by character (J), base losses are shown inside the parenthesis.

Measured mz	S/N	Elemental Composition	Theoretical *mz*	Error (ppm)	Charge	Assignment	
165.949499	27.7						
317.024092	33.6	$C_{10}H_{13}N_3O_5PS$	317.024077	-0.048	-1	w11(mC)b6 - H2O, w4(mC)b13 - H2O, x1 - OCH2	J1
319.015912	26.6	$C_{10}H_{13}N_2O_6PS$	319.015918	0.019	-1	x10(T)b7, y10(T)c7, z10(T)d7, b5(T)x12, x7(T)b10, c5(T)y12, a7(T)w10, z7(T)d10, d5(T)z12, a5(T)w12, b7(T)x10, c7(T)y10, d7(T)z10, a10(T)w7, b10(T)x7, c10(T)y7, d10(T)z7, y7(T)c10, d4(T)z13, w7(T)a10, z13(T)d4, x12(T)b5, c4(T)y13, y12(T)c5, w12(T)a5, w13(T)a4, y13(T)c4, b4(T)x13, x13(T)b4, z12(T)d5, w10(T)a7, a4(T)w13	J2
327.019667	35.9	$C_{10}H_{11}N_5O_4PS$	327.019661	-0.019	-1	w8(A)b9 - H2O, x5a12, w9(A)b8 - H2O, w6(A)b11 - H2O	J3
328.027493	19.0	$C_{10}H_{12}N_5O_4PS$	328.027486	-0.021	-1	x5a12 + 1H, a8(A)w9, w9(A)a8, x9(A)b8, y9(A)c8, z9(A)d8, w8(A)a9, y8(A)c9, z8(A)d9, w6(A)a11, x6(A)b11, y6(A)c11, z6(A)d11, b8(A)x9, c12(G)z5, c8(A)y9, z5(G)c12, d8(A)z9, a9(A)w8, b9(A)x8, c9(A)y8,	J4

						x8(A)b9, a11(A)w6, b11(A)x6, d9(A)z8, c11(A)y6, a12(G)x5, d11(A)z6
332.406213	15.8					
332.468783	34.4					
356.904022	356.8					
416.841796	23.1					
418.904416	64.2					
470.054137	16.8	$C_{16}H_{18}N_5O_8PS$	470.054094	-0.092	-1	z2 - B(dC) - OCH2
521.043300	11.0	$C_{63}H_{78}N_{26}O_{32}P_6S_6$	521.043359	0.114	-4	w6
546.038427	16.4					
577.374574	18.6	$C_{53}H_{66}N_{17}O_{30}P_5S_5$	577.374495	-0.138	-3	d5
585.381470	15.1	$C_{53}H_{66}N_{21}O_{28}P_5S_5$	585.381984	0.879	-3	w5
601.048682	25.7	$C_{73}H_{91}N_{28}O_{38}P_7S_7$	601.049158	0.791	-4	w7
631.422997	11.1					
645.736608	26.0	$C_{63}H_{77}N_{20}O_{32}P_5S_5$	645.736204	-0.625	-3	b6 - H2O, a6 - 2H
647.929731	33.2	$C_{166}H_{214}N_{60}O_{84}P_{15}S_{15}$	647.934574	7.475	-8	M - 8H2O
654.923646	10.5					
656.948065	11.8					
661.064312	12.5					
664.810898	11192.9	$C_{166}H_{205}N_{60}O_{84}P_{15}S_{15}$	664.811336	0.659	-8	M - 8H
676.256998	24.0	$C_{103}H_{128}N_{40}O_{52}P_{10}S_{10}$	676.256415	-0.862	-5	w10
683.307803	27.8	$C_{83}H_{103}N_{33}O_{42}P_8S_8$	683.307848	0.066	-4	w8
694.809922	24.6	$C_{88}H_{109}N_{27}O_{46}P_8S_8$	694.809890	-0.046	-4	b9 - B(dG) - 2H, b9 - B(A) - H2O
706.061335	52.9	$C_{43}H_{54}N_{16}O_{23}P_4S_4$	706.061776	0.624	-2	w4
712.468903	19.7	$C_{113}H_{139}N_{39}O_{56}P_{10}S_{10}$	712.468947	0.062	-5	b11 - H2O, a11 - 2H
721.483760	14.5	$C_{156}H_{190}N_{55}O_{80}P_{15}S_{15}$	721.483651	-0.151	-7	M - 7H - NH3 - 2T
728.573657	27.7	$C_{93}H_{114}N_{32}O_{46}P_8S_8$	728.573513	-0.198	-4	b9 - H2O, a9 - 2H
737.061618	18.3	$C_{22}H_{28}N_8O_{13}P_2S_2$	737.061973	0.482	-1	w2, d2
740.492242	259.4	$C_{161}H_{199}N_{55}O_{84}P_{15}S_{15}$	740.490806	-1.939	-7	M - 7H - 4H - dA
752.411054	34.9	$C_{73}H_{90}N_{22}O_{38}P_6S_6$	752.410602	-0.600	-3	a7 - 2H, b7 - H2O
759.784631	150.7	$C_{166}H_{204}N_{60}O_{84}P_{15}S_{15}$	759.784305	-0.429	-7	M - 7H - 4H
763.750269	24.6	$C_{73}H_{88}N_{28}O_{35}P_6S_6$	763.749952	-0.415	-3	z7 - 2H, y7 - H2O
765.566883	5.2	$C_{93}H_{115}N_{38}O_{46}P_9S_9$	765.566539	-0.449	-4	w9
778.479254	10.3					
781.274275	62.2	$C_{123}H_{152}N_{45}O_{60}P_{11}S_{11}$	781.278079	4.869	-5	z12 - 2H, y12 - H2O
801.733790	7.4	$C_{73}H_{91}N_{28}O_{38}P_7S_7$	801.734636	1.055	-3	w7
804.068697	7.8	$C_{123}H_{155}N_{45}O_{63}P_{12}S_{12}$	804.068889	0.239	-5	w12
809.076385	23.7					
817.083648	30.1	$C_{103}H_{125}N_{40}O_{49}P_9S_9$	817.083825	0.216	-4	y10 - H2O, z10 - 2H
821.600305	8.7	$C_{53}H_{63}N_{21}O_{25}P_4S_4$	821.599588	-0.873	-2	y5 - H2O, z5 - 2H
830.604948	14.2	$C_{53}H_{65}N_{21}O_{26}P_4S_4$	830.604871	-0.092	-2	y5

842.733073	10.0					
845.282848	14.2	$C_{133}H_{165}N_{47}O_{66}P_{12}S_{12}$	845.282718	-0.154	-5	z13 - 2H, y13 - H2O, a13 - 2H, b13 - H2O
861.752810	13.6					
863.906735	7.5	$C_{161}H_{198}N_{55}O_{84}P_{15}S_{15}$	863.905849	-1.025	-6	M - 8H - dA
873.427857	15.8	$C_{83}H_{100}N_{33}O_{39}P_{7}S_{7}$	873.428206	0.400	-3	z8 - 2H, y8 - H2O
878.575916	11.4	$C_{53}H_{66}N_{21}O_{28}P_{5}S_{5}$	878.576614	0.795	-2	w5
886.414777	24.6	$C_{166}H_{203}N_{60}O_{84}P_{15}S_{15}$	886.414931	0.174	-6	M - 8H
896.843821	5.5	$C_{113}H_{139}N_{43}O_{54}P_{10}S_{10}$	896.843619	-0.225	-4	y11 - H2O, z11 - 2H
911.414020	8.6	$C_{83}H_{103}N_{33}O_{42}P_{8}S_{8}$	911.412890	-1.240	-3	w8
925.582584	13.4	$C_{113}H_{143}N_{43}O_{57}P_{11}S_{11}$	925.584089	1.626	-4	w11 + 1H
926.747840	23.1	$C_{88}H_{109}N_{27}O_{46}P_{8}S_{8}$	926.748945	1.193	-3	b9 - B(A) - H2O, b9 - B(dG) - 2H
968.603923	28.3					
971.430996	26.1					
976.843282	10.1					
983.105775	15.1	$C_{93}H_{112}N_{38}O_{43}P_{8}S_{8}$	983.106460	0.696	-3	z9 - 2H, y9 - H2O
1021.425317	12.6	$C_{93}H_{116}N_{38}O_{46}P_{9}S_{9}$	1021.427086	1.732	-3	w9 + 1H
1043.094482	6.9	$C_{63}H_{78}N_{26}O_{32}P_{6}S_{6}$	1043.093995	-0.467	-2	w6
1094.091304	12.2	$C_{33}H_{40}N_{13}O_{18}P_{3}S_{3}$	1094.091650	0.317	-1	w3, d3
1129.117194	9.9					
			Abs mean error	0.710		
			Abs mean std dev	0.898		

Table S2 showing peak list, signal-to-noise ratio (S/N), elemental composition, and possible assignments with mass errors (ppm) of the [M-8H]$^{8-}$ MALAT-1 by CID MS/MS, internal fragments marked by character (J), base losses are shown inside the parenthesis.

Measured *mz*	S/N	Elemental Composition	Theoretical *mz*	Error (ppm)	Charge	Assignment	
319.015918	231.5	$C_{10}H_{13}N_2O_6PS$	319.015918	0.000	-1	x10(T)b7, y10(T)c7, z10(T)d7, b5(T)x12, x7(T)b10, c5(T)y12, a7(T)w10, z7(T)d10, d5(T)z12, a5(T)w12, b7(T)x10, c7(T)y10, d7(T)z10, a10(T)w7, b10(T)x7, c10(T)y7, d10(T)z7, y7(T)c10, d4(T)z13, w7(T)a10, z13(T)d4, x12(T)b5, c4(T)y13, y12(T)c5, w12(T)a5, w13(T)a4, y13(T)c4, b4(T)x13, x13(T)b4, z12(T)d5, w10(T)a7, a4(T)w13	J1
328.027433	81.8	$C_{10}H_{12}N_5O_4PS$	328.027486	0.163	-1	x5a12 + 1H, a8(A)w9, w9(A)a8, x9(A)b8, y9(A)c8, z9(A)d8, w8(A)a9, y8(A)c9, z8(A)d9, w6(A)a11, x6(A)b11, y6(A)c11, z6(A)d11, b8(A)x9, c12(G)z5, c8(A)y9, z5(G)c12, d8(A)z9, a9(A)w8, b9(A)x8, c9(A)y8, x8(A)b9, a11(A)w6, b11(A)x6, d9(A)z8, c11(A)y6, a12(G)x5, d11(A)z6	J2
337.026466	41.6	$C_{10}H_{15}N_2O_7PS$	337.026483	0.050	-1	y13(T)d4 + 2H, y12(T)d5 + 2H, y10(T)d7 + 2H, y7(T)d10 + 2H, b4(T)w13 + 2H,	J3

						w12(T)b5 + 1H, b5(T)w12 + 2H, b7(T)w10 + 2H, d7(T)y10 + 2H, d10(T)y7 + 2H, b10(T)w7 + 2H, w7(T)b10 + 1H, w13(T)b4 + 1H, w10(T)b7 + 1H, d4(T)y13 + 2H, d5(T)y12 + 2H	
344.022387	48.4	$C_{10}H_{12}N_5O_5PS$	344.022400	0.037	-1	d12(G)z5, x5(G)b12, y5(G)c12, w5(G)a12, y6(A)d11, z5(G)d12, b8(A)w9, y9(A)d8, d8(A)y9, c12(G)y5, b9(A)w8, d9(A)y8, b12(G)x5, a12(G)w5, y8(A)d9, b11(A)w6, d11(A)y6	J4
346.026698	39.2	$C_{11}H_{14}N_3O_6PS$	346.026817	0.345	-1	z15(dC)d2, d2(dC)z15, c2(dC)y15, b2(dC)x15, a2(dC)w15, w15(dC)a2, x15(dC)b2, w1 - H2O, y15(dC)c2	J5
352.984562	163.8						
356.022292	336.9	$C_{11}H_{12}N_5O_5PS$	356.022400	0.303	-1	b14(dA)x3, z3(dA)d14, d3(dA)z14, w14(dA)a3, a14(dA)w3, c14(dA)y3, y3(dA)c14, x3(dA)b14, x2a15 + 1H, c3(dA)y14, a15(dG)x2, b3(dA)x14, z2(dG)c15, a3(dA)w14, c15(dG)z2, x14(dA)b3, y14(dA)c3, z14(dA)d3, w3(dA)a14, d14(dA)z3	J6
364.025674	64.3	$C_{33}H_{40}N_{13}O_{18}P_3S_3$	364.025699	0.070	-3	d3, w3	
368.027117	1926.7	$C_{22}H_{28}N_8O_{13}P_2S_2$	368.027348	0.628	-2	w2, d2	
368.528995	254.0	$C_{48}H_{56}N_{15}O_{24}P_4S_4$	368.529122	0.344	-4	a5 - B(T) - H2O	
371.493430	138.2						
390.027761	88.7	$C_{11}H_{14}N_5O_7PS$	390.027880	0.305	-1	d15(dG)y2 + 2H, w2b15 + 1H, y2d15 + 2H, b15(dG)w2 + 2H, d1	J7
398.982293	24.7						
414.959406	131.5	$C_{10}H_{14}N_2O_8P_2S_2$	414.959405	-0.001	-1	x12(T)d5 + 1H, x13(T)d4 + 1H, d10(T)x7, c10(T)w7, c7(T)w10,	J8

						d7(T)x10, x10(T)d7 + 1H, d5(T)x12, c5(T)w12, x7(T)d10 + 1H, w13(T)c4, w12(T)c5, d4(T)x13, w10(T)c7, c4(T)w13, w7(T)c10
417.052764	114.1					
420.511784	605.3					
421.013507	99.0					
426.064272	43.2					
438.784921	62.2	$C_{53}H_{66}N_{21}O_{28}P_5S_5$	438.784669	-0.573	-4	w5
453.626744	13.5	$C_{68}H_{86}N_{23}O_{38}P_7S_7$	453.626972	0.503	-5	w7 - B(dA)
470.054300	11.4	$C_{16}H_{18}N_5O_8PS$	470.054094	-0.439	-1	z2 - B(dC) - OCH2
479.008386	45.6					
480.638072	70.5	$C_{73}H_{91}N_{28}O_{38}P_7S_7$	480.637871	-0.417	-5	w7
487.280148	19.9	$C_{58}H_{73}N_{21}O_{32}P_6S_6$	487.279736	-0.847	-4	w6 - B(dA)
494.985659	99.6					
498.570125	140.2	$C_{33}H_{39}N_{13}O_{16}P_2S_2$	498.570443	0.638	-2	b3, y3
503.997015	56.8					
512.539187	28.9	$C_{63}H_{80}N_{20}O_{35}P_6S_6$	512.538847	-0.663	-4	d6
513.999498	283.0					
519.434148	25.1	$C_{78}H_{98}N_{28}O_{42}P_8S_8$	519.433924	-0.431	-5	w8 - B(A)
521.042959	74.7	$C_{63}H_{78}N_{26}O_{32}P_6S_6$	521.043359	0.767	-4	w6
535.032067	29.4	$C_{48}H_{61}N_{16}O_{27}P_5S_5$	535.032181	0.214	-3	x5 - B(dA) + 1H, w5 - B(dG)
537.536547	12.1	$C_{33}H_{38}N_{13}O_{17}P_3S_3$	537.536904	0.665	-2	w3 - H2O, d3 - H2O
545.055150	31.2					
545.393193	73.2	$C_{53}H_{65}N_{17}O_{28}P_4S_4$	545.393333	0.257	-3	b5
546.542130	55.3	$C_{33}H_{40}N_{13}O_{18}P_3S_3$	546.542187	0.104	-2	w3, d3
553.400379	270.0	$C_{53}H_{65}N_{21}O_{26}P_4S_4$	553.400822	0.801	-3	y5
558.431030	27.5					
563.291658	22.3					
567.284655	1117.4	$C_{68}H_{86}N_{23}O_{38}P_7S_7$	567.285534	1.550	-4	w7 - B(dA)
577.063475	7.3	$C_{73}H_{90}N_{28}O_{36}P_6S_6$	577.063286	-0.328	-4	y7
579.378084	15.4	$C_{53}H_{64}N_{21}O_{27}P_5S_5$	579.378462	0.653	-3	w5 - H2O
585.381229	743.9	$C_{53}H_{66}N_{21}O_{28}P_5S_5$	585.381984	1.289	-3	w5
594.044080	10.2	$C_{108}H_{137}N_{38}O_{57}P_{11}S_{11}$	594.043247	-1.402	-6	w11 - B(A)
601.048363	647.3	$C_{73}H_{91}N_{28}O_{38}P_7S_7$	601.049158	1.323	-4	w7
604.050724	23.8	$C_{58}H_{70}N_{17}O_{31}P_5S_5$	604.049900	-1.364	-3	b6 - B(mC) - H2O, b6 - B(T) - NH3
612.551382	25.2	$C_{78}H_{97}N_{22}O_{42}P_7S_7$	612.551199	-0.298	-4	b8 - B(A) - H2O, b8 - B(dG) - 2H
616.552784	5.3	$C_{113}H_{142}N_{43}O_{57}P_{11}S_{11}$	616.552329	-0.738	-6	w11
618.060884	14.2	$C_{58}H_{72}N_{21}O_{30}P_5S_5$	618.060910	0.042	-3	y6 - B(dA)
623.254115	5.7	$C_{98}H_{124}N_{29}O_{53}P_9S_9$	623.253208	-1.456	-5	b10 - B(A)

625.558645	12.3	$C_{78}H_{97}N_{28}O_{40}P_7S_7$	625.558353	-0.467	-4	y8 - B(A)
631.046723	862.7	$C_{155}H_{189}N_{57}O_{79}P_{15}S_{15}$	631.047711	1.565	-8	c15 - 2H
631.547134	16.0	$C_{38}H_{51}N_{11}O_{22}P_4S_4$	631.544896	-3.544	-2	w4 - B(dG) + 2H
641.117889	137.6	$C_{22}H_{27}N_8O_{11}PS$	641.118485	0.930	-1	b2, y2
646.934278	20.8					
647.929370	923.4	$C_{166}H_{214}N_{60}O_{84}P_{15}S_{15}$	647.934574	8.030		M - 8H2O
649.544672	29.5	$C_{78}H_{98}N_{28}O_{42}P_8S_8$	649.544225	-0.688	-4	w8 - B(A)
658.085694	74.2					
658.090197	143.5	$C_{43}H_{53}N_{16}O_{21}P_3S_3$	658.090032	-0.251	-2	y4
659.322365	27.1	$C_{83}H_{102}N_{33}O_{40}P_7S_7$	659.321977	-0.589	-4	y8
663.079559	39.3	$C_{63}H_{77}N_{26}O_{30}P_5S_5$	663.079076	-0.729	-3	y6
663.748775	8.0	$C_{63}H_{79}N_{26}O_{30}P_5S_5$	663.750959	3.291	-3	y6 + 2H
664.809983	1777.2	$C_{166}H_{205}N_{60}O_{84}P_{15}S_{15}$	664.811336	2.036	-8	M - 8H
665.850084	46.5	$C_{103}H_{128}N_{34}O_{54}P_{10}S_{10}$	665.850692	0.912	-5	d10 - H2O
670.050543	8.6	$C_{83}H_{102}N_{27}O_{44}P_8S_8$	670.048739	-2.692	-4	c8 - 2H
670.301131	47.1	$C_{83}H_{103}N_{27}O_{44}P_8S_8$	670.300695	-0.651	-4	d8 - H2O
677.717028	14.8	$C_{63}H_{78}N_{20}O_{34}P_6S_6$	677.717366	0.499	-3	d6 - H2O
678.805739	8.6	$C_{83}H_{101}N_{33}O_{41}P_8S_8$	678.805207	-0.784	-4	w8 - H2O
683.807106	20.3					
683.809895	33.2					
685.458357	154.9	$C_{108}H_{134}N_{34}O_{56}P_{10}S_{10}$	685.458048	-0.450	-5	b11 - B(dG) - 2H, b11 - B(A) - H2O
689.057419	14.9	$C_{63}H_{76}N_{26}O_{31}P_6S_6$	689.056716	-1.021	-3	w6 - H2O
694.809383	1615.6	$C_{88}H_{109}N_{27}O_{46}P_8S_8$	694.809890	0.729	-4	b9 - B(dG) - 2H, b9 - B(A) - H2O
697.056953	32.6	$C_{43}H_{52}N_{16}O_{22}P_4S_4$	697.056494	-0.658	-2	w4 - H2O
697.549218	18.1	$C_{43}H_{51}N_{15}O_{23}P_4S_4$	697.548501	-1.028	-2	w4 - NH3, d4 - H2O
703.476698	8.4	$C_{151}H_{184}N_{53}O_{78}P_{15}S_{15}$	703.477519	1.167	-7	M - 7H - NH3 - 3T
706.061808	88.1	$C_{43}H_{54}N_{16}O_{23}P_4S_4$	706.061776	-0.045	-2	w4
713.054218	5.2	$C_{108}H_{137}N_{38}O_{57}P_{11}S_{11}$	713.053351	-1.216	-5	w11 - B(A)
716.071767	5.4	$C_{113}H_{141}N_{39}O_{57}P_{10}S_{10}$	716.071060	-0.988	-5	b11
720.225655	9.0	$C_{133}H_{166}N_{47}O_{68}P_{13}S_{13}$	720.224967	-0.955	-6	w13 - H2O, d13 - H2O
721.341468	476.6	$C_{155}H_{189}N_{57}O_{79}P_{15}S_{15}$	721.341281	-0.260	-7	c15 - 2H
724.736138	47.2	$C_{68}H_{85}N_{23}O_{36}P_6S_6$	724.735309	-1.144	-3	y7 - B(dA)
728.068317	18.7	$C_{68}H_{83}N_{25}O_{35}P_6S_6$	728.067170	-1.575	-3	y7 - B(mC)

736.091701	22.5					
738.494095	23.5					
741.230883	11.6	$C_{139}H_{172}N_{47}O_{71}P_{13}S_{13}$	741.230249	-0.856	-6	b14 - B(A) - H2O, b14 - B(G) - 2H
747.091318	70.6					
751.265622	5.8	$C_{118}H_{146}N_{39}O_{60}P_{11}S_{11}$	751.265000	-0.827	-5	b12 - B(G) - H2O, z12 - B(A) - NH3
752.411492	50.9	$C_{73}H_{90}N_{22}O_{38}P_{6}S_{6}$	752.410602	-1.183	-3	a7 - 2H, b7 - H2O
755.079986	18.4	$C_{48}H_{60}N_{16}O_{25}P_{4}S_{4}$	755.080166	0.239	-2	z5 - B(dA)
756.716866	14.6	$C_{68}H_{86}N_{23}O_{38}P_{7}S_{7}$	756.716471	-0.522	-3	w7 - B(dA)
763.740349	19.5	$C_{144}H_{177}N_{52}O_{71}P_{13}S_{13}$	763.739332	-1.331	-6	b14 - H2O, y14 - H2O, z14 - 2H, a14 - 2H
768.077326	9.8	$C_{48}H_{58}N_{18}O_{25}P_{4}S_{4}$	768.075415	-2.489	-2	y5 - B(dC)
769.753799	374.0	$C_{73}H_{90}N_{28}O_{36}P_{6}S_{6}$	769.753474	-0.422	-3	y7
774.816636	7.5	$C_{98}H_{122}N_{29}O_{52}P_{9}S_{9}$	774.815688	-1.223	-4	b10 - B(dG) - 2H, b10 - B(A) - H2O
784.392792	8.0	$C_{73}H_{91}N_{22}O_{40}P_{7}S_{7}$	784.391765	-1.309	-3	d7 - H2O
801.734165	9.8	$C_{73}H_{91}N_{28}O_{38}P_{7}S_{7}$	801.734636	0.587	-3	w7
808.579758	35.9	$C_{103}H_{127}N_{34}O_{52}P_{9}S_{9}$	808.579312	-0.552	-4	a10 - 2H, b10 - H2O
809.587847	59.1	$C_{53}H_{63}N_{17}O_{27}P_{4}S_{4}$	809.588355	0.627	-2	b5 - H2O, a5 - 2H
815.009010	22.5					
817.070655	396.8	$C_{78}H_{97}N_{22}O_{42}P_{7}S_{7}$	817.070691	0.044	-3	b8 - B(dG) - 2H, b8 - B(A) - H2O
818.594763	21.6	$C_{53}H_{65}N_{17}O_{28}P_{4}S_{4}$	818.593637	-1.376	-2	b5
823.811700	281.0					
824.020052	29.3					
830.603910	1217.8	$C_{53}H_{65}N_{21}O_{26}P_{4}S_{4}$	830.604871	1.157	-2	y5
841.733835	6.4	$C_{156}H_{191}N_{56}O_{79}P_{15}S_{15}$	841.734803	1.150	-6	M - 6H - H2O - 2T
857.074832	80.4	$C_{108}H_{134}N_{34}O_{56}P_{10}S_{10}$	857.074379	-0.528	-4	b11 - B(A) - H2O, b11 - B(dG) - 2H
862.089961	12.0	$C_{83}H_{102}N_{27}O_{42}P_{7}S_{7}$	862.088856	-1.282	-3	b8 - H2O, a8 - 2H
878.578075	42.5	$C_{53}H_{66}N_{21}O_{28}P_{5}S_{5}$	878.576614	-1.663	-2	w5
885.063632	34.8					
906.577844	312.0	$C_{58}H_{70}N_{17}O_{31}P_{5}S_{5}$	906.578488	0.710	-2	b6 - B(mC) - H2O, b6 - B(T) - NH3
907.582273	24.4	$C_{57}H_{70}N_{18}O_{31}P_{5}S_{5}$	907.580025	-2.477	-2	b6 - B(T) - CH3
926.748741	265.9	$C_{88}H_{109}N_{27}O_{46}P_{8}S_{8}$	926.748945	0.220	-3	b9 - B(A) - H2O, b9 - B(dG) - 2H
942.158461	18.0					
969.109520	12.6	$C_{63}H_{77}N_{20}O_{32}P_{5}S_{5}$	969.107944	-1.627	-2	a6 - 2H, b6 - H2O
980.138069	18.9	$C_{33}H_{37}N_{13}O_{15}P_{2}S_{2}$	980.137597	-0.481	-1	y3 - H2O, a3 - 2H, b3 - H2O, z3 - 2H
998.148489	154.5	$C_{33}H_{39}N_{13}O_{16}P_{2}S_{2}$	998.148162	-	-1	b3, y3

				0.328		
999.153265	35.9					
1094.091955	11.8	$C_{33}H_{40}N_{13}O_{18}P_3S_3$	1094.091650	-0.278	-1	w3, d3
1226.110994	8.8	$C_{78}H_{97}N_{22}O_{42}P_7S_7$	1226.109675	-1.075	-2	b8 - B(dG) - 2H, b8 - B(A) - H2O
			Abs mean error	0.856		
			Abs mean std dev	0.609		

Table S3 showing peak list, signal-to-noise ratio (S/N), elemental composition, and possible assignments with mass errors (ppm) of the $[M-8H]^{8-}$ MALAT-1 by IRMPD MS/MS, internal fragments marked by character (J), base losses are shown inside the parenthesis.

Measured *mz*	S/N	Elemental Composition	Theoretical *mz*	Error (ppm)	Charge	Assignment	
150.042285	32.6						
192.973096	974.9						
193.976452	49.3						
194.968900	38.5						
211.165591	50.0						
255.994508	233.5						
319.015934	130.4	$C_{10}H_{13}N_2O_6PS$	319.015918	-0.050	-1	x10(T)b7, y10(T)c7, z10(T)d7, b5(T)x12, x7(T)b10, c5(T)y12, a7(T)w10, z7(T)d10, d5(T)z12, a5(T)w12, b7(T)x10, c7(T)y10, d7(T)z10, a10(T)w7, b10(T)x7, c10(T)y7, d10(T)z7, y7(T)c10, d4(T)z13, w7(T)a10, z13(T)d4, x12(T)b5, c4(T)y13, y12(T)c5, w12(T)a5, w13(T)a4, y13(T)c4, b4(T)x13, x13(T)b4, z12(T)d5, w10(T)a7, a4(T)w13	J1
328.027483	55.5	$C_{10}H_{12}N_5O_4PS$	328.027486	0.008	-1	x5a12 + 1H, a8(A)w9, w9(A)a8, x9(A)b8, y9(A)c8, z9(A)d8, w8(A)a9, y8(A)c9, z8(A)d9, w6(A)a11, x6(A)b11, y6(A)c11, z6(A)d11, b8(A)x9, c12(G)z5, c8(A)y9, z5(G)c12, d8(A)z9, a9(A)w8, b9(A)x8, c9(A)y8, x8(A)b9, a11(A)w6, b11(A)x6, d9(A)z8, c11(A)y6,	J2

						a12(G)x5, d11(A)z6	
337.026480	63.5	$C_{10}H_{15}N_2O_7PS$	337.026483	0.008	-1	y13(T)d4 + 2H, y12(T)d5 + 2H, y10(T)d7 + 2H, y7(T)d10 + 2H, b4(T)w13 + 2H, w12(T)b5 + 1H, b5(T)w12 + 2H, b7(T)w10 + 2H, d7(T)y10 + 2H, d10(T)y7 + 2H, b10(T)w7 + 2H, w7(T)b10 + 1H, w13(T)b4 + 1H, w10(T)b7 + 1H, d4(T)y13 + 2H, d5(T)y12 + 2H	J 3
346.026794	24.6	$C_{11}H_{14}N_3O_6PS$	346.026817	0.067	-1	z15(dC)d2, d2(dC)z15, c2(dC)y15, b2(dC)x15, a2(dC)w15, w15(dC)a2, x15(dC)b2, w1 - H2O, y15(dC)c2	J 4
352.984577	147.9						
356.022396	67.0	$C_{11}H_{12}N_5O_5PS$	356.022400	0.011	-1	b14(dA)x3, z3(dA)d14, d3(dA)z14, w14(dA)a3, a14(dA)w3, c14(dA)y3, y3(dA)c14, x3(dA)b14, x2a15 + 1H, c3(dA)y14, a15(dG)x2, b3(dA)x14, z2(dG)c15, a3(dA)w14, c15(dG)z2, x14(dA)b3, y14(dA)c3, z14(dA)d3, w3(dA)a14, d14(dA)z3	J 5
364.025690	17.8	$C_{33}H_{40}N_{13}O_{18}P_3S_3$	364.025699	0.024	-3	d3, w3	
368.027344	193.6	$C_{22}H_{28}N_8O_{13}P_2S_2$	368.027348	0.010	-2	w2, d2	
371.493471	39.7						
390.027942	26.0	$C_{11}H_{14}N_5O_7PS$	390.027880	-0.158	-1	d15(dG)y2 + 2H, w2b15 + 1H, y2d15 + 2H, b15(dG)w2 + 2H, d1	J 6
398.982201	32.0						
414.959366	94.5	$C_{10}H_{14}N_2O_8P_2S_2$	414.959405	0.094	-1	x12(T)d5 + 1H, x13(T)d4 + 1H, d10(T)x7, c10(T)w7, c7(T)w10, d7(T)x10, x10(T)d7 + 1H, d5(T)x12, c5(T)w12, x7(T)d10 + 1H, w13(T)c4, w12(T)c5, d4(T)x13, w10(T)c7, c4(T)w13, w7(T)c10	J 7
417.052648	138.6						

418.055928	22.0						
420.511859	100.6						
432.969924	52.9	$C_{10}H_{16}N_2O_9P_2S_2$	432.969970	0.107	-1	d7(T)w10 + 2H, w7(T)d10 + 2H, d10(T)w7 + 2H, d5(T)w12 + 2H, w13(T)d4 + 2H, d4(T)w13 + 2H, w10(T)d7 + 2H, w12(T)d5 + 2H	J8
442.059113	76.2						
453.626957	18.6	$C_{68}H_{86}N_{23}O_{38}P_7S_7$	453.626972	0.033	-5	w7 - B(dA)	
470.054057	64.3	$C_{16}H_{18}N_5O_8PS$	470.054094	0.080	-1	z2 - B(dC) - OCH2	
470.372095	41.4	$C_{43}H_{54}N_{16}O_{23}P_4S_4$	470.372092	-0.006	-3	w4	
479.008402	41.4						
480.637956	29.5	$C_{73}H_{91}N_{28}O_{38}P_7S_7$	480.637871	-0.176	-5	w7	
494.985500	84.1						
495.988907	15.3						
498.570350	37.8	$C_{33}H_{39}N_{13}O_{16}P_2S_2$	498.570443	0.186	-2	b3, y3	
503.997091	41.2						
512.996053	1684.0						
513.999466	266.3						
515.000378	33.9						
522.007646	25.7						
538.002603	80.7						
545.054822	97.0						
553.400759	129.9	$C_{53}H_{65}N_{21}O_{26}P_4S_4$	553.400822	0.114	-3	y5	
554.065887	23.1						
567.285847	577.0	$C_{68}H_{86}N_{23}O_{38}P_7S_7$	567.285534	-0.551	-4	w7 - B(dA)	
577.063399	23.4	$C_{73}H_{90}N_{28}O_{36}P_6S_6$	577.063286	-0.195	-4	y7	
580.056287	16.7	$C_{92}H_{113}N_{32}O_{46}P_8S_8$	580.055790	-0.857	-5	a9 - CH3	
585.382052	234.5	$C_{53}H_{66}N_{21}O_{28}P_5S_5$	585.381984	-0.117	-3	w5	
586.047104	46.8	$C_{127}H_{157}N_{44}O_{66}P_{12}S_{12}$	586.046745	-0.612	-7	b13 - B(mC) - CH3	
597.305980	13.9						
601.049588	476.6	$C_{73}H_{91}N_{28}O_{38}P_7S_7$	601.049158	-0.716	-4	w7	
604.384341	63.6		604.385842	2.484	-3	a6 - B(mC)	
612.551467	20.7	$C_{78}H_{97}N_{22}O_{42}P_7S_7$	612.551199	-0.437	-4	b8 - B(A) - H2O, b8 - B(dG) - 2H	
618.060944	23.4	$C_{58}H_{72}N_{21}O_{30}P_5S_5$	618.060910	-0.055	-3	y6 - B(dA)	
630.922877	57.2	$C_{156}H_{194}N_{50}O_{84}P_{15}S_{15}$	630.921734	-1.811	-8	M - 8H - 4H - 2dA	
631.547891	6.5	$C_{38}H_{51}N_{11}O_{22}P_4S_4$	631.544896	-4.742	-2	w4 - B(dG) + 2H	
641.118365	271.4	$C_{22}H_{27}N_8O_{11}PS$	641.118485	0.187	-1	b2, y2	
642.121779	60.6						
647.927175	876.3	$C_{161}H_{200}N_{55}O_{84}P_{15}S_{15}$	647.929524	3.625		M - 8H - dA	
650.818018	5.0	$C_{83}H_{104}N_{27}O_{43}P_7S_7$	650.817464	-0.852	-4	b8	
658.089902	87.0	$C_{43}H_{53}N_{16}O_{21}P_3S_3$	658.090032	0.198	-2	y4	
658.447318	29.8						

658.581878	38.6	$C_{43}H_{52}N_{15}O_{22}P_3S_3$	658.582040	0.246	-2	b4
661.046256	18.2					
663.061388	15.2					
664.811623	507.8	$C_{166}H_{205}N_{60}O_{84}P_{15}S_{15}$	664.811336	-0.432	-8	M - 8H
670.551939	16.1	$C_{83}H_{104}N_{27}O_{44}P_8S_8$	670.552651	1.063	-4	c8
677.513599	13.5					
685.458267	31.5	$C_{108}H_{134}N_{34}O_{56}P_{10}S_{10}$	685.458048	-0.320	-5	b11 - B(dG) - 2H, b11 - B(A) - H2O
685.857091	22.7					
694.810341	110.0	$C_{88}H_{109}N_{27}O_{46}P_8S_8$	694.809890	-0.649	-4	b9 - B(dG) - 2H, b9 - B(A) - H2O
697.056874	7.0	$C_{43}H_{52}N_{16}O_{22}P_4S_4$	697.056494	-0.546	-2	w4 - H2O
697.548430	7.1	$C_{43}H_{51}N_{15}O_{23}P_4S_4$	697.548501	0.101	-2	w4 - NH3, d4 - H2O
703.619510	13.9	$C_{150}H_{183}N_{54}O_{78}P_{15}S_{15}$	703.619697	0.266	-7	d15 - B(T) - NH3, d15 - B(mC) - H2O
706.061275	49.4	$C_{43}H_{54}N_{16}O_{23}P_4S_4$	706.061776	0.710	-2	w4
719.051311	121.0	$C_{22}H_{26}N_8O_{12}P_2S_2$	719.051408	0.135	-1	d2 - H2O, w2 - H2O
719.738330	14.4	$C_{68}H_{86}N_{23}O_{35}P_6S_6$	719.739612	1.781	-3	z7 - B(dA) + 1H
721.198492	49.7	$C_{156}H_{194}N_{50}O_{84}P_{15}S_{15}$	721.197307	-1.643	-7	M - 7H - 4H - 2dA
724.734891	34.4	$C_{68}H_{85}N_{23}O_{36}P_6S_6$	724.735309	0.577	-3	y7 - B(dA)
736.091738	18.6					
740.635248	2133.5		740.634781	-0.631		M - 7H - 3H - dA
740.901728	12.7					
743.994048	19.1					
746.087364	246.2					
752.411435	6.7	$C_{73}H_{90}N_{22}O_{38}P_6S_6$	752.410602	-1.108	-3	a7 - 2H, b7 - H2O
755.080559	31.7	$C_{48}H_{60}N_{16}O_{25}P_4S_4$	755.080166	-0.520	-2	z5 - B(dA)
759.032567	36.6					
762.082306	50.2					
763.085669	14.8					
768.075457	19.8	$C_{48}H_{58}N_{18}O_{25}P_4S_4$	768.075415	-0.054	-2	y5 - B(dC)
769.753990	146.0	$C_{73}H_{90}N_{28}O_{36}P_6S_6$	769.753474	-0.671	-3	y7
775.384608	19.9					
784.391943	5.7	$C_{73}H_{91}N_{22}O_{40}P_7S_7$	784.391765	-0.227	-3	d7 - H2O
801.734667	16.1	$C_{73}H_{91}N_{28}O_{38}P_7S_7$	801.734636	-0.039	-3	w7
803.051979	15.5	$C_{48}H_{61}N_{16}O_{27}P_5S_5$	803.051909	-0.087	-2	x5 - B(dA) + 1H, w5 - B(dG)
808.043286	16.6	$C_{47}H_{57}N_{19}O_{26}P_5S_5$	808.043413	0.158	-2	w5 - B(T) - CH3
809.587794	19.2	$C_{53}H_{63}N_{17}O_{27}P_4S_4$	809.588355	0.693	-2	b5 - H2O, a5 - 2H
815.008762	52.2					
816.012031	16.0					
817.071087	764.6	$C_{78}H_{97}N_{22}O_{42}P_7S_7$	817.070691	-0.484	-3	b8 - B(dG) - 2H, b8 - B(A) - H2O
817.400742	47.9	$C_{78}H_{96}N_{21}O_{43}P_7S_7$	817.398696	-2.503	-3	b8 - B(A) - NH3
822.616164	20.5					
823.311133	137.8					

830.604221	1243.4	$C_{53}H_{65}N_{21}O_{26}P_4S_4$	830.604871	0.782	-2	y5
841.566828	26.9	$C_{156}H_{194}N_{50}O_{84}P_{15}S_{15}$	841.564737	-2.485	-6	M - 6H - 4H - 2dA
842.030939	386.4				-1	
849.571298	15.7	$C_{107}H_{132}N_{34}O_{55}P_{10}S_{10}$	849.571738	0.517	-4	a11 - B(dG) - CH3
857.075161	17.3	$C_{108}H_{134}N_{34}O_{56}P_{10}S_{10}$	857.074379	-0.913	-4	b11 - B(A) - H2O, b11 - B(dG) - 2H
862.423395	13.4					
878.576205	43.4	$C_{53}H_{66}N_{21}O_{28}P_5S_5$	878.576614	0.465	-2	w5
881.731105	53.4				-3	
905.046742	21.1					
906.577853	230.1	$C_{58}H_{70}N_{17}O_{31}P_5S_5$	906.578488	0.700	-2	b6 - B(mC) - H2O, b6 - B(T) - NH3
917.569871	15.9					
926.749680	64.8	$C_{88}H_{109}N_{27}O_{46}P_8S_8$	926.748945	-0.793	-3	b9 - B(A) - H2O, b9 - B(dG) - 2H
930.590531	71.8					
940.067833	13.6					
942.158090	24.9					
946.591909	21.0	$C_{118}H_{149}N_{42}O_{59}P_{11}S_{11}$	946.592515	0.640	-4	z12 - B(mC) + 2H
969.108930	5.3	$C_{63}H_{77}N_{20}O_{32}P_5S_5$	969.107944	-1.017	-2	a6 - 2H, b6 - H2O
978.113951	7.0	$C_{63}H_{79}N_{20}O_{33}P_5S_5$	978.113226	-0.741	-2	b6
980.137463	8.4	$C_{33}H_{37}N_{13}O_{15}P_2S_2$	980.137597	0.136	-1	y3 - H2O, a3 - 2H, b3 - H2O, z3 - 2H
996.068657	14.2					
998.147479	106.3	$C_{33}H_{39}N_{13}O_{16}P_2S_2$	998.148162	0.684	-1	b3, y3
1056.115251	14.9					
1076.080991	7.5	$C_{33}H_{38}N_{13}O_{17}P_3S_3$	1076.081085	0.087	-1	d3 - H2O, w3 - H2O
1091.117304	95.8					
1092.120898	36.9					
1094.091042	155.4	$C_{33}H_{40}N_{13}O_{18}P_3S_3$	1094.091650	0.556	-1	w3, d3
1129.120305	8.0	$C_{73}H_{90}N_{22}O_{38}P_6S_6$	1129.119541	-0.676	-2	b7 - H2O, a7 - 2H
1155.134480	8.4	$C_{73}H_{90}N_{28}O_{36}P_6S_6$	1155.133849	-0.546	-2	y7
1172.038629	42.9					
1173.041770	14.8					
1178.138361	24.8					
1226.110802	55.5	$C_{78}H_{97}N_{22}O_{42}P_7S_7$	1226.109675	-0.919	-2	b8 - B(dG) - 2H, b8 - B(A) - H2O
1266.123334	32.9					
1300.161235	9.0	$C_{43}H_{50}N_{15}O_{21}P_3S_3$	1300.160792	-0.341	-1	y4 - NH3, a4 - 2H, b4 - H2O
1318.17153	73.2	$C_{43}H_{52}N_{15}O_{22}P_3S_3$	1318.17135	-	-1	b4

4			6	0.135		
1413.130356	10.6	$C_{43}H_{54}N_{16}O_{23}P_4S_4$	1413.130828	0.334	-1	w4
1620.183988	8.5	$C_{53}H_{63}N_{17}O_{27}P_4S_4$	1620.183986	-0.001	-1	b5 - H2O, a5 - 2H
1662.219357	10.3	$C_{53}H_{65}N_{21}O_{26}P_4S_4$	1662.217018	-1.407	-1	y5
1718.221268	30.3					
1719.225249	21.2					
1814.167183	15.6	$C_{58}H_{70}N_{17}O_{31}P_5S_5$	1814.164253	-1.615	-1	b6 - B(mC) - H2O, b6 - B(T) - NH3
1880.103565	8.2					
1894.193199	21.2	$C_{118}H_{149}N_{42}O_{59}P_{11}S_{11}$	1894.192306	-0.472	-2	z12 - B(mC) + 2H
1895.195325	12.2					
			Abs mean error	0.438		
			Abs mean std dev	0.367		

Table S4 showing peak list, signal-to-noise ratio (S/N), elemental composition, and possible assignments with mass errors (ppm) of the $[M-8H]^{8-}$ MALAT-1 by UVPD MS/MS, internal fragments marked by character (J), base losses are shown inside the parenthesis.

Measured *mz*	S/N	Elemental Composition	Theoretical *mz*	Error (ppm)	Charge	Assignment	
158.925256	16.1						
165.949398	60.0						
174.902431	14.4						
191.965070	26.7	$C_5H_6O_4PS$	191.965165	0.495	-1	c1 - B(dG) - OCH2	J1
192.972898	48.8						
208.890182	31.3						
317.024071	39.5	$C_{10}H_{13}N_3O_5PS$	317.024077	0.019	-1	w11(mC)b6 - H2O, w4(mC)b13 - H2O, x1 - OCH2	J2
319.015911	177.2	$C_{10}H_{13}N_2O_6PS$	319.015918	0.024	-1	x10(T)b7, y10(T)c7, z10(T)d7, b5(T)x12, x7(T)b10, c5(T)y12, a7(T)w10, z7(T)d10, d5(T)z12, a5(T)w12, b7(T)x10, c7(T)y10, d7(T)z10, a10(T)w7, b10(T)x7, c10(T)y7, d10(T)z7, y7(T)c10, d4(T)z13, w7(T)a10, z13(T)d4, x12(T)b5, c4(T)y13, y12(T)c5, w12(T)a5, w13(T)a4, y13(T)c4, b4(T)x13, x13(T)b4, z12(T)d5, w10(T)a7, a4(T)w13	J3
320.019263	19.8						
327.019665	39.6	$C_{10}H_{11}N_5O_4PS$	327.019661	-0.011	-1	w8(A)b9 - H2O, x5a12, w9(A)b8 - H2O, w6(A)b11 - H2O	J4
328.027496	73.0	$C_{10}H_{12}N_5O_4PS$	328.027486	-0.032	-1	x5a12 + 1H, a8(A)w9, w9(A)a8, x9(A)b8, y9(A)c8, z9(A)d8, w8(A)a9, y8(A)c9, z8(A)d9, w6(A)a11, x6(A)b11, y6(A)c11, z6(A)d11, b8(A)x9,	J5

						c12(G)z5, c8(A)y9, z5(G)c12, d8(A)z9, a9(A)w8, b9(A)x8, c9(A)y8, x8(A)b9, a11(A)w6, b11(A)x6, d9(A)z8, c11(A)y6, a12(G)x5, d11(A)z6	
337.026479	13.7	$C_{10}H_{15}N_2O_7PS$	337.026483	0.010	-1	y13(T)d4 + 2H, y12(T)d5 + 2H, y10(T)d7 + 2H, y7(T)d10 + 2H, b4(T)w13 + 2H, w12(T)b5 + 1H, b5(T)w12 + 2H, b7(T)w10 + 2H, d7(T)y10 + 2H, d10(T)y7 + 2H, b10(T)w7 + 2H, w7(T)b10 + 1H, w13(T)b4 + 1H, w10(T)b7 + 1H, d4(T)y13 + 2H, d5(T)y12 + 2H	J6
343.014577	37.6	$C_{10}H_{11}N_5O_5PS$	343.014575	-0.005	-1	w5b12 - H2O, c1 - OCH2, w6(A)b11 - 2H, w8(A)b9 - 2H, w14(dA)b3 - OCH2, w3(dA)b14 - OCH2, w9(A)b8 - 2H	J7
344.022429	31.7	$C_{10}H_{12}N_5O_5PS$	344.022400	-0.084	-1	d12(G)z5, x5(G)b12, y5(G)c12, w5(G)a12, y6(A)d11, z5(G)d12, b8(A)w9, y9(A)d8, d8(A)y9, c12(G)y5, b9(A)w8, d9(A)y8, b12(G)x5, a12(G)w5, y8(A)d9, b11(A)w6, d11(A)y6	J8
346.026822	55.7	$C_{11}H_{14}N_3O_6PS$	346.026817	-0.014	-1	z15(dC)d2, d2(dC)z15, c2(dC)y15, b2(dC)x15, a2(dC)w15, w15(dC)a2, x15(dC)b2, w1 - H2O, y15(dC)c2	J9
356.022419	75.0	$C_{11}H_{12}N_5O_5PS$	356.022400	-0.052	-1	b14(dA)x3, z3(dA)d14, d3(dA)z14, w14(dA)a3, a14(dA)w3, c14(dA)y3, y3(dA)c14, x3(dA)b14, x2a15 + 1H, c3(dA)y14,	J10

						a15(dG)x2, b3(dA)x14, z2(dG)c15, a3(dA)w14, c15(dG)z2, x14(dA)b3, y14(dA)c3, z14(dA)d3, w3(dA)a14, d14(dA)z3	
356.904043	300.9						
364.037375	21.8	$C_{11}H_{16}N_3O_7PS$	364.037382	0.019	-1	b2(dC)w15 + 2H, d2(dC)y15 + 2H, y15d2 + 2H, w15b2 + 1H, w1	J11
372.017416	32.2	$C_{11}H_{12}N_5O_6PS$	372.017315	-0.270	-1	z2(dG)d15, w2(dG)a15, d1 - H2O, y3(dA)d14, d3(dA)y14, y14(dA)d3, b3(dA)w14, x2(dG)b15, d15(dG)z2, y2(dG)c15, b14(dA)w3, c15(dG)y2, b15(dG)x2, a15(dG)w2, d14(dA)y3	J12
390.027876	31.2	$C_{11}H_{14}N_5O_7PS$	390.027880	0.010	-1	d15(dG)y2 + 2H, w2b15 + 1H, y2d15 + 2H, b15(dG)w2 + 2H, d1	J13
397.998234	15.2						
407.993867	40.9						
413.975373	13.0	$C_{10}H_{15}N_3O_7P_2S_2$	413.975390	0.041	-1	x4(mC)d13 + 1H, x11(mC)d6 + 1H, w4(mC)c13, w11(mC)c6, d13(mC)x4, c13(mC)w4, d6(mC)x11, c6(mC)w11	J14
414.959452	50.4						
416.841900	39.4						
418.904458	89.8						
423.971034	23.3	$C_{10}H_{13}N_5O_6P_2S_2$	423.970973	-0.145	-1	d9(A)x8, c9(A)w8, c12(G)x5, c8(A)w9, x5(G)c12 + 1H, x6(A)d11 + 1H, d8(A)x9, c11(A)w6, x8(A)d9 + 1H, d11(A)x6, w9(A)c8, w6(A)c11, x9(A)d8 + 1H, w8(A)c9	J15
432.970120	15.9	$C_{10}H_{16}N_2O_9P_2S_2$	432.969970	-0.346	-1	d7(T)w10 + 2H, w7(T)d10 + 2H, d10(T)w7 + 2H, d5(T)w12 + 2H, w13(T)d4 + 2H, d4(T)w13 + 2H, w10(T)d7 + 2H, w12(T)d5 + 2H	J16

441.970371	16.2	$C_{11}H_{15}N_3O_8P_2S_2$	441.970304	-0.153	-1	w15c2, x15d2 + 1H, d2(dC)x15, c2(dC)w15	J17
451.965933	24.5	$C_{11}H_{13}N_5O_7P_2S_2$	451.965888	-0.100	-1	w14(dA)c3, w3(dA)c14, x3(dA)d14 + 1H, x14(dA)d3 + 1H, c15(dG)x2, d14(dA)x3, c14(dA)w3, x2(dG)c15 + 1H, d3(dA)x14, c3(dA)w14	J18
467.960836	23.9	$C_{11}H_{13}N_5O_8P_2S_2$	467.960802	-0.072	-1	x2d15 + 1H, d3(dA)w14, d14(dA)w3, c15(dG)w2, d15(dG)x2, w2(dG)c15, w3(dA)d14, w14(dA)d3	J19
469.046360	24.6	$C_{103}H_{127}N_{40}O_{50}P_9S_9$	469.046290	-0.148	-7	y10	
470.054270	17.9	$C_{16}H_{18}N_5O_8PS$	470.054094	-0.375	-1	z2 - B(dC) - OCH2	
478.833365	14.0						
512.996253	51.0						
522.007742	13.4						
545.534472	13.6	$C_{33}H_{38}N_{13}O_{18}P_3S_3$	545.534362	-0.201	-2	w3 - 2H, d3 - 2H	
546.542400	12.4	$C_{33}H_{40}N_{13}O_{18}P_3S_3$	546.542187	-0.389	-2	w3, d3	
585.381633	13.2	$C_{53}H_{66}N_{21}O_{28}P_5S_5$	585.381984	0.600	-3	w5	
586.555417	5.2	$C_{38}H_{44}N_{13}O_{19}P_3S_3$	586.555294	-0.209	-2	z4 - B(dC) - 2H, y4 - B(T) - NH3, b4 - B(T) - H2O	
601.049458	16.1	$C_{73}H_{91}N_{28}O_{38}P_7S_7$	601.049158	-0.499	-4	w7	
638.055123	27.1						
641.118702	21.0	$C_{22}H_{27}N_8O_{11}PS$	641.118485	-0.339	-1	b2, y2	
645.737182	14.0	$C_{63}H_{77}N_{20}O_{32}P_5S_5$	645.736204	-1.514	-3	b6 - H2O, a6 - 2H	
647.804911	23.9	$C_{161}H_{199}N_{55}O_{84}P_{15}S_{15}$	647.803546	-2.108	-8	M - 8H - 4H - dA	
657.062398	14.4	$C_{144}H_{179}N_{52}O_{72}P_{13}S_{13}$	657.062754	0.542	-7	y14, b14	
663.061700	12.1						
664.686358	947.3	$C_{166}H_{204}N_{60}O_{84}P_{15}S_{15}$	664.685358	-1.504	-8	M - 8H - 4H	
672.049124	10.5	$C_{103}H_{123}N_{40}O_{51}P_{10}S_{10}$	672.049607	0.719	-5	x10 - 4H	
673.057134	10.8	$C_{103}H_{128}N_{40}O_{51}P_{10}S_{10}$	673.057432	0.443	-5	x10 + 1H	
676.256468	33.9	$C_{103}H_{128}N_{40}O_{52}P_{10}S_{10}$	676.256415	-0.079	-5	w10	
683.558773	18.4	$C_{128}H_{159}N_{44}O_{65}P_{12}S_{12}$	683.559204	0.630	-6	a13 - B(mC)	
689.559898	22.4						
695.060777	8.1	$C_{63}H_{78}N_{26}O_{32}P_6S_6$	695.060238	-0.776	-3	w6	
703.074287	26.9						
706.061369	54.4	$C_{43}H_{54}N_{16}O_{23}P_4S_4$	706.061776	0.576	-2	w4	
712.669882	12.8						

719.051272	20.1	$C_{22}H_{26}N_8O_{12}P_2S_2$	719.051408	0.190	-1	d2 - H2O, w2 - H2O
728.573632	10.2	$C_{93}H_{114}N_{32}O_{46}P_8S_8$	728.573513	-0.163	-4	b9 - H2O, a9 - 2H
737.062121	16.5	$C_{22}H_{28}N_8O_{13}P_2S_2$	737.061973	-0.201	-1	w2, d2
740.064886	6.8	$C_{113}H_{142}N_{43}O_{57}P_{11}S_{11}$	740.064251	-0.858	-5	w11
740.492052	70.3	$C_{161}H_{199}N_{55}O_{84}P_{15}S_{15}$	740.490806	-1.683	-7	M - 7H - 4H - dA
752.074736	40.1	$C_{48}H_{62}N_{12}O_{28}P_4S_4$	752.074215	-0.693	-2	b5 - B(dA) + 2H
754.463203	11.0	$C_{118}H_{146}N_{39}O_{61}P_{11}S_{11}$	754.463983	1.034	-5	y12 - B(A) - NH3, b12 - B(G) - 2H, b12 - B(A) - H2O
759.784521	80.0	$C_{166}H_{204}N_{60}O_{84}P_{15}S_{15}$	759.784305	-0.284	-7	M - 7H - 4H
763.750391	13.2	$C_{73}H_{88}N_{28}O_{35}P_6S_6$	763.749952	-0.575	-3	z7 - 2H, y7 - H2O
781.274362	36.5	$C_{123}H_{152}N_{45}O_{60}P_{11}S_{11}$	781.278079	4.758	-5	z12 - 2H, y12 - H2O
801.734371	17.5	$C_{73}H_{91}N_{28}O_{38}P_7S_7$	801.734636	0.330	-3	w7
804.069509	9.6	$C_{123}H_{155}N_{45}O_{63}P_{12}S_{12}$	804.068889	-0.771	-5	w12
808.327478	25.6					
809.589322	25.1	$C_{53}H_{63}N_{17}O_{27}P_4S_4$	809.588355	-1.195	-2	b5 - H2O, a5 - 2H
817.010652	23.2					
821.600798	8.4	$C_{53}H_{63}N_{21}O_{25}P_4S_4$	821.599588	-1.472	-2	y5 - H2O, z5 - 2H
830.605343	5.9	$C_{53}H_{65}N_{21}O_{26}P_4S_4$	830.604871	-0.568	-2	y5
842.030972	26.5					
845.483803	10.6					
849.571789	9.5	$C_{107}H_{132}N_{34}O_{55}P_{10}S_{10}$	849.571738	-0.060	-4	a11 - B(dG) - CH3
861.753172	14.3					
864.073179	20.0	$C_{161}H_{199}N_{55}O_{84}P_{15}S_{15}$	864.073820	0.742	-6	M - 6H - 4H - dA
866.566024	7.2	$C_{53}H_{66}N_{17}O_{30}P_5S_5$	866.565381	-0.742	-2	d5
873.428024	9.4	$C_{83}H_{100}N_{33}O_{39}P_7S_7$	873.428206	0.208	-3	z8 - 2H, y8 - H2O
878.576005	15.9	$C_{53}H_{66}N_{21}O_{28}P_5S_5$	878.576614	0.693	-2	w5
886.581952	104.1	$C_{166}H_{204}N_{60}O_{84}P_{15}S_{15}$	886.582902	1.072	-6	M - 6H - 4H
906.578071	7.7	$C_{58}H_{70}N_{17}O_{31}P_5S_5$	906.578488	0.460	-2	b6 - B(mC) - H2O, b6 - B(T) - NH3
911.747433	15.0	$C_{83}H_{104}N_{33}O_{42}P_8S_8$	911.748832	1.535	-3	w8 + 1H
926.749561	5.2	$C_{88}H_{109}N_{27}O_{46}P_8S_8$	926.748945	-0.665	-3	b9 - B(A) - H2O, b9 - B(dG) - 2H
968.604133	20.1					
969.108598	17.6	$C_{63}H_{77}N_{20}O_{32}P_5S_5$	969.107944	-0.675	-2	a6 - 2H, b6 - H2O
971.766255	13.2					
972.101125	13.5					
976.592047	9.2	$C_{123}H_{149}N_{44}O_{61}P_{11}S_{11}$	976.591509	-0.551	-4	a12 - 4H
977.085164	19.1					
983.105901	9.1	$C_{93}H_{112}N_{38}O_{43}P_8S_8$	983.106460	0.568	-3	z9 - 2H, y9 -

						H2O
986.117343	8.7	$C_{63}H_{75}N_{26}O_{29}P_5S_5$	986.116969	-0.379	-2	y6 - H2O, z6 - 2H
995.122638	15.1	$C_{63}H_{77}N_{26}O_{30}P_5S_5$	995.122252	-0.388	-2	y6
998.148057	17.6	$C_{33}H_{39}N_{13}O_{16}P_2S_2$	998.148162	0.105	-1	b3, y3
1023.079811	10.5					
1037.689256	9.0					
1043.094569	5.1	$C_{63}H_{78}N_{26}O_{32}P_6S_6$	1043.093995	-0.550	-2	w6
1060.103697	26.7					
1061.106877	9.4					
1094.090911	42.6	$C_{33}H_{40}N_{13}O_{18}P_3S_3$	1094.091650	0.676	-1	w3, d3
1128.616298	10.3					
1129.119593	23.7					
1226.109271	9.7	$C_{78}H_{97}N_{22}O_{42}P_7S_7$	1226.109675	0.329	-2	b8 - B(dG) - 2H, b8 - B(A) - H2O
1300.160351	6.7	$C_{43}H_{50}N_{15}O_{21}P_3S_3$	1300.160792	0.339	-1	y4 - NH3, a4 - 2H, b4 - H2O
1317.187220	18.7	$C_{43}H_{53}N_{16}O_{21}P_3S_3$	1317.187341	0.092	-1	y4
1318.190619	8.5					
1413.130663	8.1	$C_{43}H_{54}N_{16}O_{23}P_4S_4$	1413.130828	0.117	-1	w4
			Abs mean error	0.654		
			Abs mean std dev	0.771		

Table S5 showing peak list, signal-to-noise ratio (S/N), elemental composition, and possible assignments with mass errors (ppm) of the [M-8H]$^{8-}$ danvatirsen by EDD MS/MS, internal fragments marked by character (J), base losses are shown inside the parenthesis.

Measured *mz*	S/N	Elemental Composition	Theoretical *mz*	Error (ppm)	Charg e	Assignmen t	
318.008280	90.6	$C_{10}H_{12}N_2O_6PS$	318.008093	- 0.590	-1	y4d13 - NH2, y5(T)c12 - 1H, z5(T)d12 - 1H, x5(T)b12 - 1H, w4b13 - NH3, w7(T)b10 - H2O, d4(T)z13 - 1H, d6(T)z11 - 1H, c4(T)y13 - 1H, c5(T)y12 - 1H, d5(T)z12 - 1H, a6(T)w11 - 1H, b6(T)x11 - 1H, w5(T)a12 - 1H, b4(T)x13 - 1H, c6(T)y11 - 1H, a5(T)w12 - 1H, x7(T)b10 - 1H, a4(T)w13 - 1H, w7(T)a10 - 1H, w5(T)b12 - H2O, y7(T)c10 - 1H, z7(T)d10 - 1H, b5(T)x12 - 1H	J 1
319.016114	99.6	$C_{10}H_{13}N_2O_6PS$	319.015918	- 0.610	-1	a5(T)w12, a4(T)w13, b4(T)x13, c4(T)y13, d4(T)z13, w4b13 - NH2, z5(T)d12, b5(T)x12, x5(T)b12, y5(T)c12, d6(T)z11, c5(T)y12, w7(T)a10, d5(T)z12, a6(T)w11, c6(T)y11, b6(T)x11, y7(T)c10, z7(T)d10, w5(T)a12, x7(T)b10	J 2
327.019835	17.7	$C_{10}H_{11}N_5O_4PS$	327.019661	- 0.530	-1	w8b9 - H2O, x8(A)b9 - 1H, z6(G)c11 - 1H, c8(G)z9 - 1H, a8(G)x9 - 1H, y8(A)c9 - 1H, z8(A)d9 - 1H, c7(G)z10 - 1H, a7(G)x10 - 1H, x6a11, b9(A)x8 - 1H, w8(A)a9 - 1H, a9(A)w8 - 1H	J 3
328.027641	18.4	$C_{10}H_{12}N_5O_4PS$	328.027486	- 0.470	-1	z6(G)c11, y8(A)c9, c8(G)z9, a8(G)x9, c7(G)z10, a7(G)x10,	J 4

						a9(A)w8, x8(A)b9, w8(A)a9, b9(A)x8, z8(A)d9	
328.027641	18.4	$C_{10}H_{11}N_5O_4PS$	328.027486	0.470	-1		
343.014692	115.7	$C_{10}H_{11}N_5O_5PS$	343.014575	- 0.340	-1	w6b11 - H2O, d7(G)z10 - 1H, c7(G)y10 - 1H, z6(G)d11 - 1H, b7(G)x10 - 1H, a7(G)w10 - 1H, d8(G)z9 - 1H, x6(G)b11 - 1H, y6(G)c11 - 1H, w8b9 - 2H, y8(A)d9 - 1H, w6(G)a11 - 1H, a8(G)w9 - 1H, b9(A)w8 - 1H, b8(G)x9 - 1H, c8(G)y9 - 1H	J5
344.022520	58.5	$C_{10}H_{12}N_5O_5PS$	344.022400	- 0.350	-1	b9(A)w8, w6(G)a11, d8(G)z9, c8(G)y9, b8(G)x9, a8(G)w9, c7(G)y10, b7(G)x10, d7(G)z10, a7(G)w10, x6(G)b11, y8(A)d9, y6(G)c11, z6(G)d11, w8b9 - 1H	J6
344.022520	58.5	$C_{10}H_{11}N_5O_5PS$	344.022400	0.350	-1		
361.026609	16.0	$C_{12}H_{14}O_7N_2PS$	361.026482	- 0.350	-1	w1 - NH3, d1 - NH3	
370.038056	21.2	$C_{12}H_{14}N_5O_5PS$	370.038050	- 0.020	-1	c3(dA)y14, a3(dA)w14, w3(dA)a14, y3(dA)c14, z2(dG)c15, b3(dA)x14, z3(dA)d14, x3(dA)b14, d3(dA)z14	J7
370.038056	21.2	$C_{12}H_{13}N_5O_5PS$	370.038050	0.020	-1		
378.053049	31.4	$C_{12}H_{17}O_7N_3PS$	378.053032	- 0.040	-1	d1, w1	
398.982245	31.3	$C_9H_7N_9O_4P_2S$	398.982243	0.010	-1		
409.522859	60.2	$C_{21}H_{38}N_4O_{16}P_7$	409.522856	0.010	-2		
414.959337	18.1	$C_8H_{13}N_5O_5P_4S$	414.959336	0.000	-1		
414.959337	18.1	$C_{10}H_{14}N_2O_8P_2S_2$	414.959405	0.160	-1	c6(T)w11, d4(T)x13, d6(T)x11, c4(T)w13, w7(T)c10, c5(T)w12, d5(T)x12, w5(T)c12	J8
423.988646	18.2	$C_7H_{17}N_6O_5PS_4$	423.988638	0.020	-1		
432.969837	23.6	$C_{21}H_{10}N_2OPS_3$	432.969837	0.000	-1		
450.004224	22.9	$C_7H_{19}N_9O_2P_3S_3$	450.004219	0.010	-1		
457.976441	6.2	$C_9H_8N_{12}O_3P_2S_2$	457.976447	0.010	-2		
472.094704	15.8	$C_8H_7N_{23}OP$	472.094703	0.000	-1		
484.387762	26.4	$C_{46}H_{57}N_{16}O_{23}P_4S_4$	484.387742	- 0.040	-3	w4	
491.530627	6.6	$C_{33}H_{67}N_2$	491.530974	0.710	-2		

512.995973	33.8	$C_5H_3N_{22}O_3P_2S$	512.995991	0.040	-1	
554.558438	46.6	$C_{36}H_{44}O_{19}N_{10}P_3S_3$	554.558508	0.130	-2	d3 - 3H
567.565546	8.6	$C_{36}H_{44}O_{18}N_{13}P_3S_3$	567.565662	0.200	-2	w3
591.061951	23.4	$C_{56}H_{70}N_{18}O_{29}P_5S_5$	591.062140	0.320	-3	w5
595.075252	3.9	$C_{41}H_{49}N_{10}O_{20}P_3S_3$	595.075528	0.460	-2	y4 - B(dG) - NH3, b4 - B(T) - H2O, a4 - B(T)
609.308003	48.5	$C_{76}H_{94}N_{25}O_{40}P_7S_7$	609.308004	0.000	-4	w7
639.038898	18.5	$C_{29}H_{20}N_7OP_3S_2$	639.038910	0.020	-1	
647.548037	3.0	$C_{41}H_{53}N_7O_{25}P_4S_4$	647.547044	-1.530	-2	d4 - B(dA)
651.407623	9.6	$C_{66}H_{79}N_{16}O_{34}P_5S_5$	651.408424	1.230	-3	a6 - 4H
651.744473	24.8	$C_{66}H_{80}N_{16}O_{34}P_5S_5$	651.744548	0.110	-3	b6 - H2O, a6
652.406790	2.9	$C_{36}H_{64}NO_3P_2S$	652.408764	3.030	-1	
652.409297	2.9	$C_{66}H_{80}N_{15}O_{35}P_5S_5$	652.408495	-1.230	-3	b6 - NH2
657.593024	115.5	$C_{46}H_{54}N_{12}O_{22}P_3S_3$	657.593080	0.080	-2	a4 - 3H
658.097096	47.3	$C_{46}H_{55}N_{12}O_{22}P_3S_3$	658.096992	-0.160	-2	b4 - H2O, a4
658.591164	12.1	$C_{46}H_{54}N_{11}O_{23}P_3S_3$	658.589274	-2.870	-2	b4 - NH3
660.819049	16.9	$C_{86}H_{102}N_{26}O_{44}P_7S_7$	660.819337	0.440	-4	a8 - 5H
664.045342	47.0	$C_{10}H_{20}N_{16}O_{11}P_4$	664.045342	0.000	-1	
668.087356	4.0	$C_{66}H_{79}N_{23}O_{31}P_5S_5$	668.087348	-0.010	-3	z6, y6 - H2O
668.551047	4.5	$C_{43}H_{68}N_6$	668.551095	0.070	-2	
674.067670	73.2	$C_{172}H_{207}N_{56}O_{87}P_{15}S_{15}$	674.067673	0.000	-8	M - 8H - H2O
676.317903	5102.3	$C_{172}H_{209}N_{56}O_{88}P_{15}S_{15}$	676.318994	1.610	-8	M - 8H
676.940402	402.1	$C_4H_{10}N_{23}O_5P_7$	676.940408	0.010	-1	
681.057404	14.4	$C_{106}H_{129}N_{33}O_{57}P_{10}S_{10}$	681.056526	-1.290	-5	d10 - 6H
682.225587	12.7	$C_{126}H_{153}N_{44}O_{66}P_{12}S_{12}$	682.225114	-0.690	-6	w12 - 7H
682.724923	3.1	$C_3H_8N_4O_{17}P_9S$	682.724923	0.000	-2	
690.863221	18.7	$C_{106}H_{128}N_{40}O_{54}P_{10}S_{10}$	690.862316	-1.310	-5	w10 - 6H
691.262475	10.1	$C_{29}H_{43}N_{10}O_4S_3$	691.263637	1.680	-5	
691.566315	5.8	$C_{27}H_{77}N_{14}P_2S$	691.565707	0.880	-2	
691.566556	24.5	$C_{86}H_{106}N_{30}O_{44}P_8S_8$	691.566694	0.200	-4	w8
692.315180	5.5	$C_{23}H_{64}N_6O_3P_4S_3$	692.315800	0.900	-2	
695.562624	17.4	$C_{37}H_{81}N_2O_5P_2$	695.562621	0.000	-2	
706.071190	5.1	$C_{48}H_{20}OP_2S$	706.071559	0.520	-1	
706.071868	12.9	$C_{66}H_{82}N_{23}O_{34}P_6S_6$	706.072032	0.230	-3	w6
715.073812	5.7	$C_{46}H_{58}N_{12}O_{25}P_4S_4$	715.074018	0.290	-2	d4
721.022577	12.4	$C_{23}HN_{25}O_2P_2$	721.022577	0.000	-1	
721.022740	4.1	$C_{14}H_{21}N_{19}O_3P_6S$	721.022667	0.100	-2	
727.085199	32.6	$C_{46}H_{58}N_{16}O_{23}P_4S_4$	727.085251	0.070	-2	w4
735.206725	3.1	$C_{160}H_{195}O_{84}N_{53}P_{15}S_{15}$	735.207567	1.150	-7	w15, d15
740.086474	20.5	$C_{24}H_{32}O_{14}N_5P_2S_2$	740.086791	0.430	-1	d2
743.079104	64.9	$C_{96}H_{114}N_{31}O_{48}P_8S_8$	743.078164	-1.270	-4	a9 - 5H
743.577455	19.4	$C_{96}H_{114}N_{30}O_{49}P_8S_8$	743.576124	-1.790	-4	b9 - NH3
747.082548	11.3	$C_{22}H_{54}NO_2PS_{11}$	747.082548	0.000	-6	
749.335613	10.7	$C_{96}H_{115}N_{35}O_{46}P_8S_8$	749.335600	-0.020	-4	z9, y9 - H2O
754.214385	2.2	$C_{29}H_{62}NOP_6S_4$	754.214546	0.210	-1	

754.867596	5.3	$C_{116}H_{141}N_{42}O_{60}P_{11}S_{11}$	754.866955	-0.850	-5	w11 - 6H
765.093008	27.3	$C_{24}H_{31}O_{13}N_{8}P_{2}S_{2}$	765.093273	0.350	-1	w2
766.419627	51.0	$C_{76}H_{91}N_{21}O_{39}P_{6}S_{6}$	766.418499	-1.470	-3	a7 - 4H
772.792196	62.6	$C_{172}H_{208}N_{56}O_{88}P_{15}S_{15}$	772.792017	-0.230	-7	M - 9H
774.761375	9.7	$C_{76}H_{92}N_{25}O_{37}P_{6}S_{6}$	774.761746	0.480	-3	y7 - H2O, z7
777.573379	20.3	$C_{96}H_{117}N_{35}O_{49}P_{9}S_{9}$	777.572294	-1.400	-4	w9 - 5H
791.276961	55.7	$C_{126}H_{151}N_{40}O_{65}P_{11}S_{11}$	791.276290	-0.850	-5	a12 - 6H
804.739265	8.0	$C_{76}H_{95}N_{21}O_{42}P_{7}S_{7}$	804.739124	-0.180	-3	d7
812.411861	33.1	$C_{76}H_{94}N_{25}O_{40}P_{7}S_{7}$	812.410672	-1.460	-3	w7 - 4H
818.108874	17.8	$C_{41}H_{36}N_{5}P_{4}S_{3}$	818.108881	0.010	-1	
818.872474	122.4	$C_{126}H_{154}N_{44}O_{66}P_{12}S_{12}$	818.871593	-1.080	-5	w12 - 6H
823.085037	17.3	$C_{106}H_{127}N_{33}O_{54}P_{9}S_{9}$	823.083963	-1.300	-4	a10 - 5H
823.583285	5.5	$C_{106}H_{127}N_{32}O_{55}P_{9}S_{9}$	823.581923	-1.650	-4	b10 - NH3
829.617160	3.5	$C_{56}H_{67}N_{18}O_{26}P_{4}S_{4}$	829.616184	-1.180	-2	z5 - 3H
830.119776	35.3	$C_{56}H_{68}N_{18}O_{26}P_{4}S_{4}$	830.119822	0.060	-2	y5 - H2O, z5
833.120823	17.7	$C_{8}H_{8}N_{43}O_{4}PS$	833.120823	0.000	-1	
835.593028	7.2	$C_{106}H_{127}N_{40}O_{51}P_{9}S_{9}$	835.593020	-0.010	-4	z10, y10 - H2O
839.844984	9.5	$C_{106}H_{128}N_{40}O_{52}P_{9}S_{9}$	839.843841	-1.360	-4	y10 - 5H
851.823838	4.8	$C_{106}H_{131}N_{33}O_{57}P_{10}S_{10}$	851.824295	0.540	-4	d10
855.084521	4.4	$C_{136}H_{165}N_{43}O_{70}P_{12}S_{12}$	855.084125	-0.460	-5	a13 - 6H
858.092009	16.8	$C_{19}H_{18}N_{28}O_{6}P_{4}$	858.092007	0.000	-1	
858.092103	3.7	$C_{25}H_{22}N_{25}P_{6}$	858.092121	0.020	-2	
860.086105	116.5	$C_{136}H_{164}N_{46}O_{69}P_{12}S_{12}$	860.085422	-0.790	-5	z13 - 6H
861.886908	2.6	$C_{105}H_{189}N_{43}O_{88}P_{14}S_{13}$	861.887187	0.320	-5	
863.830516	7.3	$C_{106}H_{129}N_{40}O_{54}P_{10}S_{10}$	863.829713	-0.930	-4	w10 - 5H
875.085776	3.7	$C_{56}H_{71}N_{14}O_{31}P_{5}S_{5}$	875.085889	0.130	-2	d5
881.429716	38.3	$C_{86}H_{103}N_{26}O_{44}P_{7}S_{7}$	881.428391	-1.500	-3	a8 - 4H
882.094080	8.4	$C_{86}H_{103}N_{25}O_{45}P_{7}S_{7}$	882.092338	-1.980	-3	b8 - NH3
884.440693	48.1	$C_{86}H_{104}N_{30}O_{41}P_{7}S_{7}$	884.440183	-0.580	-3	y8 - H2O, z8
885.105249	8.4	$C_{86}H_{104}N_{29}O_{42}P_{7}S_{7}$	885.104130	-1.260	-3	y8 - NH2
887.096956	35.8	$C_{56}H_{71}N_{18}O_{29}P_{5}S_{5}$	887.096848	-0.120	-2	w5
899.094468	4.2	$C_{172}H_{209}N_{56}O_{87}P_{15}S_{15}$	899.092747	-1.910	-6	M - 6H - H2O
901.591283	71.5	$C_{172}H_{208}N_{56}O_{88}P_{15}S_{15}$	901.590596	-0.760	-6	M - 9H
901.758566	74.1	$C_{172}H_{209}N_{56}O_{88}P_{15}S_{15}$	901.758566	0.000	-6	M - 8H

905.589165	11.2	$C_{32}H_{78}N_{18}O_{10}P$	905.589141	0.030	-1	
908.419775	2.4	$C_{84}H_{105}N_{30}O_{43}P_8S_8$	908.421163	1.530	-3	w8 - OC2H2
909.341954	16.2	$C_{116}H_{139}N_{38}O_{59}P_{10}S_{10}$	909.341382	-0.630	-4	a11 - 5H
915.348509	8.6	$C_{116}H_{139}N_{42}O_{57}P_{10}S_{10}$	915.346999	-1.650	-4	z11 - 5H
922.425147	28.0	$C_{86}H_{107}N_{30}O_{44}P_8S_8$	922.424867	-0.300	-3	w8
943.836734	9.0	$C_{116}H_{142}N_{42}O_{60}P_{11}S_{11}$	943.835512	-1.290	-4	w11 - 5H
977.618677	15.1	$C_{66}H_{80}N_{16}O_{34}P_5S_5$	977.616548	-2.180	-2	a6 - 3H
989.347530	13.2	$C_{126}H_{152}N_{40}O_{65}P_{11}S_{11}$	989.347181	-0.350	-4	a12 - 5H
991.107124	30.9	$C_{96}H_{115}N_{31}O_{48}P_8S_8$	991.106645	-0.480	-3	a9 - 4H
991.442065	40.5	$C_{96}H_{116}N_{31}O_{48}P_8S_8$	991.442404	0.340	-3	b9 - H2O, a9
999.448856	11.1	$C_{96}H_{116}N_{35}O_{46}P_8S_8$	999.449893	1.040	-3	y9 - H2O, z9
1002.130936	12.8	$C_{66}H_{79}N_{23}O_{31}P_5S_5$	1002.131022	0.090	-2	z6 - 3H
1037.100430	9.8	$C_{96}H_{118}N_{35}O_{49}P_9S_9$	1037.098818	-1.550	-3	w9 - 4H
1040.195836	3.0	$C_{36}H_{44}O_{16}N_{13}P_2S_2$	1040.195661	-0.170	-1	y3
1059.612671	9.1	$C_{66}H_{83}N_{23}O_{34}P_6S_6$	1059.611961	-0.670	-2	w6
1075.110347	6.3	$C_{71}H_{88}N_{16}O_{38}P_6S_6$	1075.110320	-0.030	-2	b7 - B(G) - H2O, a7 - B(G)
1136.138618	22.5	$C_{36}H_{45}O_{18}N_{13}P_3S_3$	1136.138600	-0.020	-1	w3
1150.134243	7.4	$C_{76}H_{92}N_{21}O_{39}P_6S_6$	1150.131386	-2.480	-2	a7 - 3H
1219.624522	12.0	$C_{76}H_{96}N_{25}O_{40}P_7S_7$	1219.623558	-0.790	-2	w7
1317.202369	9.3	$C_{46}H_{56}N_{12}O_{22}P_3S_3$	1317.201810	-0.420	-1	b4 - H2O, a4
			Abs mean error	0.770		
			Abs mean std dev	0.671		

Table S6 showing peak list, signal-to-noise ratio (S/N), elemental composition, and possible assignments with mass errors (ppm) of the $[M-8H]^{8-}$ danvatirsen by CID MS/MS, internal fragments marked by character (J), base losses are shown inside the parenthesis.

Measured *mz*	S/N	Elemental Composition	Theoretical *mz*	Error (ppm)	Charge	Assignment	
319.016302	16.9	$C_{10}H_{13}N_2O_6PS$	319.015918	-1.200	-1	a5(T)w12, a4(T)w13, b4(T)x13, c4(T)y13, d4(T)z13, w4b13 - NH2, z5(T)d12, b5(T)x12, x5(T)b12, y5(T)c12, d6(T)z11, c5(T)y12, w7(T)a10, d5(T)z12, a6(T)w11, c6(T)y11, b6(T)x11, y7(T)c10, z7(T)d10, w5(T)a12, x7(T)b10	J1
337.026752	22.2	$C_5H_{11}N_{10}O_2P_2S$	337.026788	0.110	-3		
344.022589	97.6	$C_{10}H_{12}N_5O_5PS$	344.0224	-0.550	-1	b9(A)w8, w6(G)a11, d8(G)z9, c8(G)y9, b8(G)x9, a8(G)w9, c7(G)y10, b7(G)x10, d7(G)z10, a7(G)w10, x6(G)b11, y8(A)d9, y6(G)c11, z6(G)d11, w8b9 - 1H	J2
344.022589	97.6	$C_5H_8N_{13}P_2S$	344.022706	0.340	-1		
359.999701	17.0	$C_{15}H_6NO_{10}$	359.999719	0.050	-1		
361.026451	20.5	$C_{12}H_{14}O_7N_2PS$	361.026482	0.090	-1	w1 - NH3, d1 - NH3	
369.539755	1044.1	$C_{24}H_{31}O_{14}N_5P_2S_2$	369.539757	0.010	-2	d2	
370.038087	803.4	$C_{12}H_{14}N_5O_5PS$	370.03805	-0.100	-1	c3(dA)y14, a3(dA)w14, w3(dA)a14, y3(dA)c14, z2(dG)c15, b3(dA)x14, z3(dA)d14, x3(dA)b14, d3(dA)z14	J3
378.041426	19.5	$C_{36}H_{43}O_{18}N_{13}P_3S_3$	378.041349	-0.200	-3	w3	
378.053060	20.1	$C_{12}H_{17}O_7N_3PS$	378.053032	-0.070	-1	d1, w1	
414.959683	143.4	$C_{10}H_{14}N_2O_8P_2S_2$	414.959405	-0.670	-1	c6(T)w11, d4(T)x13, d6(T)x11, c4(T)w13, w7(T)c10, c5(T)w12, d5(T)x12,	J4

						w5(T)c12
442.059229	134.0	$C_{14}H_{23}NO_9P_3$	442.059116	0.260	-1	
476.380460	18.8	$C_{46}H_{57}N_{12}O_{25}P_4S_4$	476.380253	- 0.430	-3	d4
484.387972	145.0	$C_{46}H_{57}N_{16}O_{23}P_4S_4$	484.387742	- 0.480	-3	w4
487.245048	46.6	$C_{76}H_{93}N_{25}O_{40}P_7S_7$	487.244948	- 0.210	-5	w7
491.530667	55.1	$C_{24}H_{44}NO_{30}P_3S_2$	491.53067	0.010	-2	
494.985108	88.0	$C_{10}H_4N_{14}O_7PS$	494.98512	0.030	-1	
500.043990	30.3	$C_2H_5N_{27}P_3$	500.043958	0.060	-2	
507.090852	79.9	$C_{36}H_{44}O_{17}N_{10}P_2S_2$	507.090677	- 0.350	-2	b3
512.996265	503.1	$C_3H_6N_{20}O_6PS_2$	512.996371	0.210	-1	
519.593932	79.5	$C_{36}H_{43}O_{16}N_{13}P_2S_2$	519.593918	- 0.030	-2	y3
538.002826	548.2	$C_3H_5N_{23}O_5PS_2$	538.002853	0.050	-1	
545.054238	18.1	$C_{33}H_5N_8O_2$	545.054095	0.260	-1	
545.055739	12.1	$C_{32}H_{21}OP_4$	545.054837	1.650	-2	
553.051453	38.0	$C_{86}H_{105}N_{30}O_{44}P_8S_8$	553.0519	0.810	-5	w8
558.559663	68.2	$C_{36}H_{42}O_{17}N_{13}P_3S_3$	558.56038	1.280	-2	x3 - 3H, w3 - H2O
561.104992	9.2	$C_{40}H_{18}O_2P$	561.10499	0.000	-1	
567.565418	614.7	$C_{36}H_{44}O_{18}N_{13}P_3S_3$	567.565662	0.430	-2	w3
571.545809	12.0	$C_{33}H_{72}N_4OP$	571.544924	1.550	-4	
578.042940	17.9	$C_{71}H_{87}N_{22}O_{39}P_7S_7$	578.043276	0.580	-4	w7 - B(mC)
579.316507	8.6	$C_{57}H_{98}N_{13}O_{71}P_7$	579.31607	0.750	-2	
579.316876	31.2	$C_{76}H_{93}N_{21}O_{40}P_6S_6$	579.316515	- 0.620	-4	b7
585.322011	9.8	$C_{76}H_{93}N_{25}O_{38}P_6S_6$	585.322132	0.210	-4	y7
586.112564	43.6	$C_{17}H_{19}N_{17}O_2P_3$	586.112598	0.060	-1	
588.520297	19.7	$C_{31}H_{62}N_{11}$	588.519515	1.330	-1	
591.062123	41.4	$C_{56}H_{70}N_{18}O_{29}P_5S_5$	591.06214	0.030	-3	w5
598.799595	439.5	$C_{76}H_{92}N_{21}O_{41}P_7S_7$	598.799746	0.250	-4	d7 - H2O, c7 - 5H
606.546232	16.3	$C_{111}H_{136}N_{37}O_{60}P_{11}S_{11}$	606.546713	0.790	-6	w11 - B(A)
607.473110	7.8	$C_{131}H_{157}N_{44}O_{68}P_{13}S_{13}$	607.47395	1.380	-7	x13 - B(T) - H2O
609.306996	855.9	$C_{76}H_{94}N_{25}O_{40}P_7S_7$	609.308004	1.650	-4	w7
611.118205	9.9	$C_{17}H_{31}N_4O_{16}S_2$	611.118197	0.010	-1	
617.080897	6.9	$C_{39}H_{14}N_4O_3P$	617.080901	0.010	-2	
622.056857	16.6	$C_{96}H_{117}N_{35}O_{49}P_9S_9$	622.057835	1.570	-5	w9
626.909298	27.6	$C_{136}H_{167}N_{43}O_{73}P_{13}S_{13}$	626.909629	0.530	-7	d13
629.055178	45.9	$C_{116}H_{141}N_{42}O_{60}P_{11}S_{11}$	629.055795	0.980	-6	w11
629.585589	17.1	$C_{38}H_{73}N_6O$	629.585135	0.720	-2	
629.888011	6.7	$C_{30}H_2NO_{10}P_2S$	629.888014	0.000	-2	
632.404431	16.0	$C_{61}H_{74}N_{20}O_{31}P_5S_5$	632.404566	0.210	-3	y6 - B(mC)
633.817908	12.8	$C_{81}H_{100}N_{25}O_{42}P_7S_7$	633.817198	- 1.120	-4	y8 - B(A)
635.057846	27.9	$C_{100}H_{118}N_{35}O_{50}P_9S_9$	635.058383	0.850	-5	z10 - B(G) - CH3
635.251314	9.6	$C_{29}H_{43}N_6O_4S_3$	635.251341	0.040	-1	
636.315657	8.2	$C_{81}H_{98}N_{27}O_{41}P_7S_7$	636.316094	0.690	-4	y8 - B(mC)
639.038094	23.0	$C_{21}H_{35}O_8S_7$	639.03824	0.230	-1	
640.555532	7.8	$C_3HO_8P_4S_{11}$	640.555521	0.020	-8	
644.142043	112.3	$C_{24}H_{32}N_5O_{12}PS$	644.143303	1.956	-1	b2
647.445705	12.7	$C_{39}H_{60}N_4O_2P$	647.445938	0.360	-1	
650.644193	21.0	$C_{101}H_{123}N_{28}O_{56}P_{10}S_{10}$	650.644969	1.190	-5	c10 - 6H, d10 - B(A) - H2O

657.431926	51.4	$C_{166}H_{200}N_{51}O_{88}P_{15}S_{15}$	657.433269	2.040	-8	M - 8H - CH4 - A, M - 8H - CH4 - dA
657.747255	247.9	$C_{66}H_{82}N_{16}O_{35}P_5S_5$	657.747887	0.961	-3	b6
659.436168	189.3	$C_{167}H_{204}N_{51}O_{88}P_{15}S_{15}$	659.437182	1.540	-8	M - 8H - A, M - 8H - dA
660.686436	10.4	$C_{167}H_{202}N_{53}O_{87}P_{15}S_{15}$	660.68663	0.290	-8	M - 8H - mC, M - 8H - dC
665.570689	120.3	$C_{77}H_{128}N_{12}O_{55}P_{14}S_4$	665.570486	0.300	-4	
667.099483	244.0	$C_6H_{10}N_{34}OP_3$	667.099516	0.050	-2	
668.077100	8.3	$C_8H_{15}N_{25}O_7PS_2$	668.077081	0.030	-4	
669.147209	105.3	$C_{27}H_{36}N_4O_8P_3S$	669.14722	0.020	-1	
671.123176	6.5	$C_{30}H_{24}N_8O_7PS$	671.123177	0.000	-2	
671.873093	88.2	$C_{106}H_{128}N_{40}O_{52}P_9S_9$	671.875073	2.950	-5	y10
672.300599	10.5	$C_{24}H_{42}N_{13}O_8S$	672.30055	0.070	-1	
674.089461	1718.4	$C_{66}H_{81}N_{23}O_{32}P_5S_5$	674.09087	2.090	-3	y6
674.754557	30.8	$C_{71}H_{207}N_{24}O_{12}P_{12}S_2$	674.754488	0.100	-1	
674.754914	125.9	$C_{71}H_{207}N_{24}O_{12}P_{12}S_2$	674.754488	0.630	-3	
676.316933	3240.6	$C_{172}H_{209}N_{56}O_{88}P_{15}S_{15}$	676.318994	3.050	-8	M - 8H
676.814953	323.4	$C_{10}H_{10}N_{10}O_6P_9S$	676.814956	0.000	-1	
677.654698	677.3	$C_{106}H_{128}N_{33}O_{56}P_{10}S_{10}$	677.655868	1.730	-5	d10 - H2O, c10 - 6H
679.112368	178.9	$C_7H_{21}N_{24}O_9P_2S$	679.112477	0.160	-1	
679.112403	1440.4	$C_{46}H_{57}N_{16}O_{21}P_3S_3$	679.113508	1.630	-2	y4
683.724256	1004.2	$C_{66}H_{81}N_{16}O_{36}P_6S_6$	683.725528	1.860	-3	d6 - H2O, c6 - 4H
685.057039	132.6	$C_{86}H_{104}N_{26}O_{46}P_8S_8$	685.057165	0.180	-4	d8 - H2O, c8 - 5H
687.063626	48.8	$C_{86}H_{104}N_{30}O_{43}P_8S_8$	687.064053	0.620	-4	x8 - 5H, w8 - H2O
687.260687	22.3	$C_{106}H_{126}N_{40}O_{53}P_{10}S_{10}$	687.260093	-0.860	-5	x10 - 7H
689.051115	45.7	$C_{11}H_{25}N_{12}O_{15}P_4$	689.05183	1.040	-1	
689.077193	11.4	$C_{37}H_{23}O_{10}P_2$	689.077195	0.000	-1	
700.068793	233.2	$C_{66}H_{80}N_{23}O_{33}P_6S_6$	700.068511	-0.400	-3	w6 - H2O, x6 - 4H
701.397176	5.7	$C_{23}H_{49}N_{20}O_4S$	701.397185	0.010	-1	
703.517556	14.0	$C_{43}H_{77}OP_2S$	703.517586	0.040	-2	
706.069608	115.3	$C_9H_{25}N_{23}O_2P_5S_2$	706.069658	0.070	-1	
706.571653	26.7	$C_{46}H_{57}N_{12}O_{24}P_4S_4$	706.572648	1.410	-2	c4
709.565242	1469.6	$C_{91}H_{110}N_{26}O_{48}P_8S_8$	709.56636	1.570	-4	b9 - B(A) - H2O, a9 - B(A)
716.070118	98.5	$C_{71}H_{86}N_{16}O_{38}P_6S_6$	716.068513	-2.240	-3	a7 - 4H
716.404583	666.2	$C_{71}H_{87}N_{16}O_{38}P_6S_6$	716.404454	-0.180	-3	a7 - B(G)
718.080894	115.6	$C_{46}H_{56}N_{16}O_{22}P_4S_4$	718.079969	-1.290	-2	w4 - H2O, x4 - 3H
720.074873	18.0	$C_{91}H_{112}N_{30}O_{47}P_8S_8$	720.074618	-0.350	-4	y9 - B(A)
722.573475	21.8	$C_{91}H_{110}N_{32}O_{46}P_8S_8$	722.573514	0.050	-4	y9 - B(mC)
726.000900	7.4	$C_{33}H_{23}N_5OP_3S_4$	726.00038	0.720	-1	
730.414408	7.2	$C_{38}H_{71}NO_2P_3S_2$	730.414457	0.070	-1	
731.374546	5.2	$C_{32}H_{64}N_2O_{10}PS_2$	731.374549	0.000	-3	
732.727678	7.1	$C_{136}H_{165}N_{46}O_{71}P_{13}S_{13}$	732.728432	1.030	-6	w13 - H2O, x13 - 7H
735.879743	46.2	$C_{116}H_{141}N_{42}O_{58}P_{10}S_{10}$	735.879712	-0.040	-5	y11
739.412986	12.4	$C_{49}H_{51}N_6O$	739.412984	0.000	-1	

741.978350	15.8	$C_4H_{12}N_{31}P_8$	741.979839	2.010	-1	
747.082836	185.8	$C_{24}H_{29}O_{12}N_8P_2S_2$	747.082708	-0.170	-1	w2 - H2O, x2 - 2H
751.357716	23.8	$C_{167}H_{204}N_{51}O_{87}P_{15}S_{15}$	751.357427	-0.380	-7	M - 8H - dG, M - 8H - G
753.837454	606.0	$C_{96}H_{117}N_{35}O_{47}P_8S_8$	753.838241	1.040	-4	y9
756.069827	5.6	$C_{143}H_{173}O_{74}N_{46}P_{13}S_{13}$	756.069657	-0.220	-6	y14 - B(dA) - H2O, b14 - 8H, z14 - B(dA)
759.990140	5.9	$C_{12}H_{14}N_{19}O_{13}S_4$	759.99068	0.710	-1	
762.082244	296.4	$C_{18}H_{20}N_{17}O_{14}S_2$	762.082254	0.010	-1	
766.753748	12.4	$C_{76}H_{92}N_{21}O_{39}P_6S_6$	766.754257	0.660	-3	a7, b7 - H2O
767.996171	7.6	$C_{15}H_9N_{21}O_{12}P_3$	767.995789	0.500	-1	
772.756870	23.9	$C_{76}H_{94}N_{21}O_{40}P_6S_6$	772.757779	1.180	-3	b7
773.321818	9.0	$C_{96}H_{116}N_{35}O_{48}P_9S_9$	773.321472	-0.450	-4	w9 - H2O, x9 - 5H
778.578321	21.4	$C_{148}H_{178}O_{74}N_{51}P_{13}S_{13}$	778.578739	0.540	-6	z14, y14 - H2O
780.764421	694.9	$C_{76}H_{94}N_{25}O_{38}P_6S_6$	780.765268	1.080	-3	y7
781.429049	37.5	$C_{23}H_{54}N_{22}O_7P$	781.428893	0.200	-1	
782.097432	21.5	$C_{46}H_{28}NO_4P_4$	782.09743	0.000	-1	
787.089053	166.9	$C_{15}H_{28}N_{14}O_{18}PS_2$	787.089053	0.000	-1	
789.597782	14.5	$C_{44}H_{84}N_6P_3$	789.597581	0.250	-2	
791.477932	46.9	$C_{126}H_{152}N_{40}O_{65}P_{11}S_{11}$	791.477745	-0.240	-5	b12 - H2O, a12
797.606186	6.7	$C_{16}H_6NO_7P_6S_9$	797.606188	0.000	-2	
798.734813	32.1	$C_{76}H_{93}N_{21}O_{41}P_7S_7$	798.73542	0.760	-3	d7 - H2O, c7 - 4H
813.081499	12.6	$C_{57}H_{19}O_3P_2$	813.081492	0.010	-1	
818.108344	388.6	$C_{56}H_{68}N_{14}O_{28}P_4S_4$	818.108589	0.300	-2	b5 - H2O, a5
818.109338	135.7	$C_6H_{12}N_{33}O_{17}$	818.10944	0.120	-1	
819.105046	20.7	$C_{56}H_{68}N_{13}O_{29}P_4S_4$	819.104509	-0.660	-2	b5 - NH2
827.113825	24.3	$C_{56}H_{70}N_{14}O_{29}P_4S_4$	827.113871	0.060	-2	b5
831.414901	93.3	$C_{81}H_{99}N_{21}O_{43}P_7S_7$	831.414346	-0.670	-3	a8 - B(G)
831.734304	8.0	$N_{23}O_{12}P_2S_8$	831.73432	0.020	-1	
839.125676	198.5	$C_{56}H_{70}N_{18}O_{27}P_4S_4$	839.125105	-0.680	-2	y5
860.286133	7.0	$C_{136}H_{165}N_{46}O_{69}P_{12}S_{12}$	860.286877	0.860	-5	z13, y13 - H2O
865.022312	44.7	$C_{23}H_{42}N_2O_{15}P_9$	865.022925	0.710	-1	
866.080752	16.3	$C_{56}H_{69}N_{14}O_{30}P_5S_5$	866.080333	-0.480	-2	c5 - 3H, d5 - H2O
877.582481	17.3	$C_{16}HN_3O_{13}P_{13}S$	877.582463	0.020	-2	
878.078549	7.1	$C_{47}H_8N_{15}O_6$	878.078747	0.230	-2	
881.747870	9.4	$CH_3N_{27}O_7P_7S_5$	881.748114	0.280	-3	
881.763772	45.1	$C_{86}H_{104}N_{26}O_{44}P_7S_7$	881.764149	0.430	-3	a8, b8 - H2O
887.097606	42.7	$C_{56}H_{71}N_{18}O_{29}P_5S_5$	887.096848	-0.850	-2	w5
890.443697	7.1	$C_{86}H_{106}N_{30}O_{42}P_7S_7$	890.443522	-0.200	-3	y8
906.136910	11.3	$C_{13}H_{24}N_{35}O_5P_4S$	906.137632	0.800	-1	
915.148178	12.6	$C_{38}H_{39}N_6O_{13}S_4$	915.146344	2.000	-1	
941.052742	9.5	$C_{23}H_{45}N_6O_{16}P_8S$	941.05192	0.870	-1	
946.425379	22.0	$C_{91}H_{111}N_{26}O_{48}P_8S_8$	946.424239	-1.200	-3	a9 - B(A), b9 - B(A) - H2O
953.105694	5.3	$C_{36}H_{61}O_3P_4S_9$	953.106308	0.640	-2	
966.059216	7.2	$C_{14}H_7N_{43}O_4P_3S$	966.060522	1.350	-1	
975.073500	5.5	$C_{18}H_{31}N_{20}O_{18}S_5$	975.073424	0.080	-1	
978.120325	29.6	$C_{66}H_{81}N_{16}O_{34}P_5S_5$	978.120186	-	-2	b6 - H2O, a6

				0.140		
984.069486	32.2	$C_{25}H_{53}N_7O_{10}P_{11}S$	984.069393	0.090	-1	
987.125702	7.3	$C_{66}H_{83}N_{16}O_{35}P_5S_5$	987.125469	-0.240	-2	b6
992.075975	15.1	$C_{16}H_{38}N_{19}O_{17}S_7$	992.074353	1.640	-1	
993.081995	22.4	$C_{31}H_{51}N_6O_9P_{10}S$	993.081992	0.000	-1	
1000.575325	5.1	$C_{21}H_7N_2O_{15}P_6S_9$	1000.576404	1.080	-2	
1015.189284	16.8	$C_{36}H_{45}O_{17}N_{10}P_2S_2$	1015.18863	-0.640	-1	b3
1022.165799	10.1	$C_{34}H_{64}N_7O_3P_{12}S$	1022.164828	0.950	-1	
1022.185296	71.2	$C_{36}H_{42}O_{15}N_{13}P_2S_2$	1022.184547	-0.730	-1	y3 - H2O, z3
1026.092495	5.1	$C_{66}H_{82}N_{16}O_{36}P_6S_6$	1026.09193	-0.550	-2	c6 - 3H, d6 - H2O
1034.085684	11.4	$C_{22}H_{46}N_{19}O_7P_6S_5$	1034.086236	0.530	-1	
1039.089600	8.2	$C_{27}H_{26}N_{30}O_3P_3S_4$	1039.090534	0.900	-1	
1040.194770	505.5	$C_{36}H_{44}O_{16}N_{13}P_2S_2$	1040.195112	0.330	-1	y3
1041.201247	49.1	$C_{18}H_{35}N_{28}O_{21}P_2$	1041.201228	0.020	-1	
1075.111078	62.5	$C_{71}H_{88}N_{16}O_{38}P_6S_6$	1075.11032	-0.710	-2	b7 - B(G) - H2O, a7 - B(G)
1082.107037	26.7	$C_{33}H_{62}N_9O_2P_{14}S$	1082.107935	0.830	-1	
1091.095415	5.4	$C_{54}H_{30}N_{14}P_7$	1091.09467	0.680	-1	
1091.120228	16.1	$C_{32}H_{51}O_{33}S_4$	1091.120092	0.120	-1	
1102.154273	6.5	$C_{24}H_{70}N_{15}O_2P_{10}S_6$	1102.154287	0.010	-1	
1107.113799	19.9	$C_{30}H_7N_{46}S_3$	1107.112942	0.770	-1	
1118.128842	22.3	$C_{36}H_{43}O_{17}N_{13}P_3S_3$	1118.128036	-0.720	-1	w3 - H2O, x3 - 2H
1136.141301	28.1	$C_{36}H_{45}O_{18}N_{13}P_3S_3$	1136.1386	-2.377	-1	w3
1150.638005	11.1	$C_{76}H_{93}N_{21}O_{39}P_6S_6$	1150.635024	-2.590	-2	b7 - H2O, a7
1169.050290	9.8	$C_{80}H_{23}N_2P_2S_3$	1169.05041	0.100	-1	
1171.651547	10.6	$C_{76}H_{95}N_{25}O_{38}P_6S_6$	1171.65154	-0.010	-2	y7
1178.052399	12.9	$C_{79}H_{27}NP_3S_3$	1178.052398	0.000	-1	
1208.151636	6.6	$C_{40}H_{49}N_{11}O_{21}P_3S_3$	1208.148497	-2.600	-1	y4 - B(dA) - CH4
1225.175013	10.2	$C_{87}H_{31}N_4P_2S$	1225.175016	0.000	-1	
1234.163527	5.4	$C_{86}H_{29}NO_8P$	1234.163627	0.080	-1	
1247.629162	10.4	$C_{82}H_{92}N_2O_3PS_2$	1247.629248	0.070	-2	
1317.201301	55.7	$C_{46}H_{56}N_{12}O_{22}P_3S_3$	1317.201261	-0.030	-1	b4 - H2O, a4
			Abs mean error	1.879		

Abs mean std dev	6.545

Table S7 showing peak list, signal-to-noise ratio (S/N), elemental composition, and possible assignments with mass errors (ppm) of the $[M-8H]^{8-}$ danvatirsen by IRMPD MS/MS, internal fragments marked by character (J), base losses are shown inside the parenthesis.

Measured *mz*	S/N	Elemental Composition	Theoretical *mz*	Error (ppm)	Charge	Assignment	
319.015936	326.8	$C_{10}H_{13}N_2O_6PS$	319.015918	-0.060	-1	a5(T)w12, a4(T)w13, b4(T)x13, c4(T)y13, d4(T)z13, w4b13 - NH2, z5(T)d12, b5(T)x12, x5(T)b12, y5(T)c12, d6(T)z11, c5(T)y12, w7(T)a10, d5(T)z12, a6(T)w11, c6(T)y11, b6(T)x11, y7(T)c10, z7(T)d10, w5(T)a12, x7(T)b10	J1
344.022388	80.3	$C_{10}H_{12}N_5O_5PS$	344.022400	0.040	-1	b9(A)w8, w6(G)a11, d8(G)z9, c8(G)y9, b8(G)x9, a8(G)w9, c7(G)y10, b7(G)x10, d7(G)z10, a7(G)w10, x6(G)b11, y8(A)d9, y6(G)c11, z6(G)d11, w8b9 - 1H	J2
360.042454	57.4	$C_{12}H_{15}O_6N_3PS$	360.042467	0.040	-1	x1 - 2H, c1 - 2H, d1 - H2O, w1 - H2O	J3
361.026462	103.0	$C_{12}H_{14}O_7N_2PS$	361.026482	0.060	-1	w1 - NH3, d1 - NH3	
369.539723	146.9	$C_{24}H_{31}O_{14}N_5P_2S_2$	369.539757	0.090	-2	d2	
378.041314	42.8	$C_{36}H_{43}O_{18}N_{13}P_3S_3$	378.041349	0.090	-3	w3	
378.053018	56.8	$C_{12}H_{17}O_7N_3PS$	378.053032	0.040	-1	d1, w1	
386.032911	54.4	$C_{12}H_{14}N_5O_6PS$	386.032965	0.140	-1	b3(dA)w14, z2(dG)d15, y2(dG)c15, x2(dG)b15, w3b14 - 1H, w2(dG)a15, y3(dA)d14, d3(dA)y14	J5
386.032911	54.4	$C_{11}H_{19}NO_8P_3$	386.032901	0.030	-1		
388.048703	15.6	$C_{12}H_{15}N_5O_6PS$	388.048615	0.230	-1		
398.982222	48.1	$C_{10}H_{13}N_2O_9P_2S$	398.982249	0.070	-1		
414.959383	192.4	$C_{10}H_{14}N_2O_8P_2S_2$	414.959405	0.050	-1	c6(T)w11, d4(T)x13, d6(T)x11, c4(T)w13, w7(T)c10, c5(T)w12, d5(T)x12,	J6

						w5(T)c12	
414.959383	192.4	$C_{10}H_{13}N_2O_8P_2S_2$	414.959405	0.050	-1		
414.959455	79.7	$C_{10}H_{14}N_2O_8P_2S_2$	414.959405	-0.120	-1	c6(T)w11, d4(T)x13, d6(T)x11, c4(T)w13, w7(T)c10, c5(T)w12, d5(T)x12, w5(T)c12	J7
417.052741	47.6	$C_{15}H_{18}N_2O_8PS$	417.052697	0.110	-1		
421.955313	9.9	$C_{10}H_{11}N_5O_6P_2S_2$	421.955323	0.020	-1	d8(G)x9 - H2O, x6(G)c11 - 1H, c8(G)w9 - H2O, w8(A)d9 - H2O, c8(G)x9 - 2H, d7(G)x10 - H2O, w8c9 - 2H, w6(G)c11 - H2O, x8(A)d9 - 1H, c7(G)x10 - 2H, c7(G)w10 - H2O	J8
423.988698	29.9	$C_9H_{18}NO_{10}P_4$	423.988667	0.070	-1		
431.693867	41.3	$C_{41}H_{47}N_{12}O_{21}P_4S_4$	431.694284	0.970	-3	x4 - B(mC) - NH2	
432.028283	20.2	$C_{79}H_{18}N_{11}O_7S_2$	432.028285	0.010	-3		
439.965857	72.8	$C_{10}H_{13}N_5O_7P_2S_2$	439.965888	0.070	-1	d8(G)x9, w6(G)c11, c7(G)w10, w8(A)d9, d7(G)x10, c8(G)w9	J9
439.965857	72.8	$C_{13}H_{22}NP_8$	439.965869	0.030	-1		
442.059196	63.4	$C_{14}H_{23}NO_9P_3$	442.059116	0.180	-1		
450.004372	11.1	$C_{12}H_{14}N_5O_8P_2S$	450.004381	0.020	-1		
458.079170	15.2	$C_{17}H_{21}O_8N_3PS$	458.079247	0.170	-1	z2 - B(dG) - OC2H2, a2 - B(dU) - OC2H2	J10
465.981472	26.4	$C_{11}H_{20}NO_9P_4S$	465.981474	0.000	-1		
465.981472	26.4	$C_{12}H_{15}N_5O_7P_2S_2$	465.981538	0.140	-1	d3(dA)x14, w3c14, c3(dA)w14	J11
484.387795	18.7	$C_{46}H_{57}N_{16}O_{23}P_4S_4$	484.387742	-0.110	-3	w4	
487.244768	11.1	$C_{76}H_{93}N_{25}O_{40}P_7S_7$	487.244948	0.370	-5	w7	
494.985578	98.3	$C_{12}H_{11}N_{12}OP_4S$	494.985546	0.060	-1		
504.014912	22.3	$C_{17}H_{30}NS_8$	504.014943	0.060	-1		
507.090706	17.8	$C_{36}H_{44}O_{17}N_{10}P_2S_2$	507.090677	-0.060	-2	b3	
512.996156	173.0	$C_{16}H_{22}O_9P_5$	512.995741	0.810	-1		
519.593954	21.5	$C_{36}H_{43}O_{16}N_{13}P_2S_2$	519.593918	-0.070	-2	y3	
519.992079	47.1	$C_{18}H_{26}NOP_8$	519.992084	0.010	-1		
538.002646	166.9	$C_{16}H_{21}N_3O_8P_5$	538.002223	0.790	-1		
540.549121	38.0	$O_2P_3S_{13}$	540.548589	0.990	-2		
553.052098	14.6	$C_{86}H_{105}N_{30}O_{44}P_8S_8$	553.051900	-0.360	-5	w8	
558.560494	7.4	$C_{36}H_{42}O_{17}N_{13}P_3S_3$	558.560380	-0.200	-2	x3 - 3H, w3 - H2O	
561.106223	15.4	$C_{24}H_{30}N_4O_2P_5$	561.106235	0.020	-1		
567.565807	8.1	$C_{36}H_{44}O_{18}N_{13}P_3S_3$	567.565662	-0.260	-2	w3	
570.056437	81.7	$C_{28}H_4N_{13}O_3$	570.056555	0.210	-2		

586.112617	53.0	$C_{19}H_{31}N_3O_{12}P_3$	586.112608	0.020	-1	
586.112701	21.6	$C_{23}H_{34}N_5OP_2S_4$	586.112693	0.010	-1	
598.799952	38.6	$C_{76}H_{92}N_{21}O_{41}P_7S_7$	598.799746	-0.340	-4	d7 - H2O, c7 - 5H
600.072847	33.0	$C_{32}H_{11}N_9O_3P$	600.072796	0.090	-2	
609.308293	35.2	$C_{76}H_{94}N_{25}O_{40}P_7S_7$	609.308004	-0.470	-4	w7
618.537086	7.6	$C_2N_2O_2P_9S_8$	618.536953	0.220	-4	
639.039020	70.6	$C_{16}H_{24}N_{12}O_4PS_5$	639.039014	0.010	-1	
644.143261	111.3	$C_{24}H_{31}O_{12}N_5PS$	644.143303	0.070	-1	b2
645.127354	15.8	$C_{28}H_{34}N_4O_4P_5$	645.127364	0.020	-1	
653.047679	7.3	$C_{79}H_{96}N_{28}O_{42}P_8S_8$	653.048137	0.700	-4	w8 - B(T) - C2H4
656.070613	11.3	$C_{84}H_{99}N_{30}O_{41}P_7S_7$	656.070356	-0.390	-4	z8 - C2H4
657.748183	53.5	$C_{66}H_{82}N_{16}O_{35}P_5S_5$	657.747887	-0.450	-3	b6
658.079247	9.3	$C_{11}H_{15}N_{23}O_6P_3$	658.079400	0.230	-3	
658.097320	50.1	$C_{46}H_{55}N_{12}O_{22}P_3S_3$	658.096992	-0.500	-2	b4 - H2O, a4
659.684766	10.0	$C_5H_5N_9O_2P_{11}S_3$	659.684765	0.000	-2	
661.868677	7.3	$C_{106}H_{128}N_{33}O_{55}P_9S_9$	661.867718	-1.450	-5	b10 - 6H
664.045544	89.8	$C_{23}H_{34}N_3O_4P_8$	664.045576	0.050	-2	
665.214662	30.4	$C_{25}H_{47}N_6O_3P_6$	665.214085	0.870	-1	
667.102431	29.9	$C_{46}H_{57}N_{12}O_{23}P_3S_3$	667.102274	-0.240	-2	b4
667.580824	15.2	$C_{86}H_{105}N_{30}O_{42}P_7S_7$	667.580822	0.000	-4	y8
668.551347	33.1	$C_6N_2O_4P_7S_9$	668.551330	0.020	-1	
669.149799	21.5	$C_{24}H_{30}O_{11}N_8PS$	669.149786	-0.020	-1	y2
670.026527	25.8	$C_{11}H_{13}N_{23}O_3P_5$	670.026530	0.000	-3	
670.108240	28.0	$C_{46}H_{55}N_{16}O_{20}P_3S_3$	670.108225	-0.020	-2	y4 - H2O, z4
674.091205	62.4	$C_{66}H_{81}N_{23}O_{32}P_5S_5$	674.090870	-0.500	-3	y6
674.422158	14.4	$C_{22}H_{66}N_{11}O_4P_2S_2$	674.422140	0.030	-3	
676.193679	371.6	$C_{172}H_{208}N_{56}O_{88}P_{15}S_{15}$	676.193016	-0.980	-8	M - 9H
677.854638	6.6	$C_{106}H_{127}N_{32}O_{57}P_{10}S_{10}$	677.852671	-2.900	-5	d10 - NH3
679.113558	57.2	$C_{46}H_{57}N_{16}O_{21}P_3S_3$	679.113508	-0.070	-2	y4
680.111376	6.5	$C_{79}H_{48}N_2O_{15}S_3$	680.111390	0.020	-2	
683.725850	34.3	$C_{66}H_{81}N_{16}O_{36}P_6S_6$	683.725528	-0.470	-3	d6 - H2O, c6 - 4H
684.390895	7.8	$C_{66}H_{81}N_{15}O_{37}P_6S_6$	684.389475	-2.080	-3	d6 - NH2
685.056832	11.8	$C_{86}H_{104}N_{26}O_{46}P_8S_8$	685.057165	0.490	-4	d8 - H2O, c8 - 5H
690.061253	11.5	$C_{22}H_{26}N_7O_{11}P_2S_2$	690.061245	0.010	-1	
700.068444	8.3	$C_{66}H_{80}N_{23}O_{33}P_6S_6$	700.068511	0.100	-3	w6 - H2O, x6 - 4H
706.068897	10.7	$C_{46}H_{56}N_{12}O_{24}P_4S_4$	706.068736	-0.230	-2	d4 - H2O, c4 - 3H
709.566719	59.4	$C_{91}H_{110}N_{26}O_{48}P_8S_8$	709.566360	-0.510	-4	b9 - B(A) - H2O, a9 - B(A)
709.814353	19.3	$C_{91}H_{109}N_{25}O_{49}P_8S_8$	709.812364	-2.800	-4	b9 - B(A) - NH3
716.404357	44.6	$C_{71}H_{87}N_{16}O_{38}P_6S_6$	716.404454	0.140	-3	a7 - B(G)

718.079869	8.7	$C_{46}H_{56}N_{16}O_{22}P_4S_4$	718.079969	0.140	-2	w4 - H2O, x4 - 3H
722.076223	11.3	$C_{24}H_{30}O_{13}N_5P_2S_2$	722.076226	0.000	-1	d2 - H2O, c2 - 2H
726.001493	18.2	$C_{41}H_{11}N_7P_3S$	726.001499	0.010	-1	
732.071754	12.4	$C_{24}H_{28}O_{12}N_7P_2S_2$	732.071809	0.080	-1	x2 - NH2
737.075746	14.2	$C_{21}H_{31}N_{10}O_8P_6$	737.075754	0.010	-1	
740.086792	8.4	$C_{24}H_{32}O_{14}N_5P_2S_2$	740.086791	0.000	-1	d2
747.083103	40.1	$C_{24}H_{29}O_{12}N_8P_2S_2$	747.082708	-0.530	-1	w2 - H2O, x2 - 2H
753.587785	10.2	$C_{96}H_{116}N_{35}O_{47}P_8S_8$	753.586285	-1.990	-4	y9 - 5H
780.765634	34.0	$C_{76}H_{94}N_{25}O_{38}P_6S_6$	780.765268	-0.470	-3	y7
818.108671	21.5	$C_{56}H_{68}N_{14}O_{28}P_4S_4$	818.108589	-0.100	-2	b5 - H2O, a5
827.113486	5.7	$C_{56}H_{70}N_{14}O_{29}P_4S_4$	827.113871	0.470	-2	b5
831.414376	20.8	$C_{81}H_{99}N_{21}O_{43}P_7S_7$	831.414346	-0.040	-3	a8 - B(G)
839.124994	32.6	$C_{56}H_{70}N_{18}O_{27}P_4S_4$	839.125105	0.130	-2	y5
840.015255	53.8	$C_{27}H_{44}NO_7P_6S_5$	840.015254	0.000	-1	
840.128425	17.9	$C_{86}H_{199}N_3O_4P_{10}S$	840.128425	0.000	-2	
887.097144	14.2	$C_{56}H_{71}N_{18}O_{29}P_5S_5$	887.096848	-0.330	-2	w5
888.125468	6.5	$C_{67}H_{14}N_5$	888.125469	0.000	-1	
906.135901	8.6	$C_{15}H_{42}N_{17}O_{16}P_2S_4$	906.135901	0.000	-1	
941.051857	18.8	$C_{22}H_{51}N_2O_{18}P_{10}$	941.051856	0.000	-1	
947.139794	28.3	$C_{42}H_{27}N_8O_{19}$	947.139794	0.000	-1	
947.139937	11.2	$C_{15}H_{44}N_{22}O_{10}PS_7$	947.139885	0.050	-1	
956.151449	28.0	$C_{35}H_{60}NO_9P_2S_8$	956.151449	0.000	-1	
956.151596	10.6	$C_{18}H_{47}N_5O_{35}PS$	956.151540	0.060	-1	
978.120379	14.2	$C_{66}H_{81}N_{16}O_{34}P_5S_5$	978.120186	-0.200	-2	b6 - H2O, a6
984.068731	32.2	$C_{15}H_{45}N_{23}O_2P_{11}S_2$	984.068731	0.000	-1	
984.068849	11.8	$C_{12}H_{32}N_{19}O_{26}P_2S_2$	984.068802	0.050	-1	
993.080976	7.5	$C_{29}H_{33}N_{14}O_{16}S_5$	993.080800	0.180	-1	
1015.188568	8.4	$C_{36}H_{45}O_{17}N_{10}P_2S_2$	1015.188630	0.060	-1	b3
1022.183413	13.6	$C_{36}H_{42}O_{15}N_{13}P_2S_2$	1022.184547	1.110	-1	y3 - H2O, z3
1034.084549	12.9	$C_{19}H_{44}N_{15}O_{19}P_8$	1034.084434	0.110	-1	
1040.195451	106.0	$C_{36}H_{44}O_{16}N_{13}P_2S_2$	1040.195112	-0.330	-1	y3
1075.110901	20.1	$C_{71}H_{88}N_{16}O_{38}P_6S_6$	1075.110320	-0.540	-2	b7 - B(G) - H2O, a7 - B(G)
1091.117316	7.9	$C_{35}H_{37}N_{24}OP_6S_3$	1091.117551	0.220	-1	
1102.150010	5.8	$C_{25}H_{64}N_5O_{24}P_4S_5$	1102.150075	0.060	-1	
1107.112242	6.5	$C_{26}H_{65}N_{14}O_2P_{10}S_6$	1107.112088	0.140	-1	
1118.128645	9.6	$C_{36}H_{43}O_{17}N_{13}P_3S_3$	1118.128036	-0.540	-1	w3 - H2O, x3 - 2H
1136.137582	31.2	$C_{36}H_{45}O_{18}N_{13}P_3S_3$	1136.138600	0.900	-1	w3
1141.120459	37.2	$C_{79}H_{17}N_8O_2S$	1141.120067	0.340	-1	
1201.152696	7.3	$C_{87}H_{31}O_2P_2S$	1201.152549	0.120	-1	
1317.20130	59.1	$C_{46}H_{56}N_{12}O_{22}P_3S_3$	1317.20126	-	-1	b4 - H2O, a4

2	1	0.030
	Abs mean error	0.407
	Abs mean std dev	0.582

Table S8 showing peak list, signal-to-noise ratio (S/N), elemental composition, and possible assignments with mass errors (ppm) of the $[M-8H]^{8-}$ danvatirsen by UVPD MS/MS, internal fragments marked by character (J), base losses are shown inside the parenthesis.

Measured *mz*	S/N	Elemental Composition	Theoretical *mz*	Error (ppm)	Charge	Assignment	
165.949534	243.1	$CH_3N_3OP_3$	165.949446	0.530	-1		
317.024069	45.9	$C_{10}H_{13}N_3O_5PS$	317.024077	0.020	-1	w4(mC)a13 - 1H, w4b13 - H2O, z4(mC)d13 - 1H, x4(mC)b13 - 1H, y4(mC)c13 - 1H	J1
319.015954	290.6	$C_{10}H_{13}N_2O_6PS$	319.015918	-0.110	-1	a5(T)w12, a4(T)w13, b4(T)x13, c4(T)y13, d4(T)z13, w4b13 - NH2, z5(T)d12, b5(T)x12, x5(T)b12, y5(T)c12, d6(T)z11, c5(T)y12, w7(T)a10, d5(T)z12, a6(T)w11, c6(T)y11, b6(T)x11, y7(T)c10, z7(T)d10, w5(T)a12, x7(T)b10	J2
328.027552	46.2	$C_{10}H_{12}N_5O_4PS$	328.027486	-0.200	-1	z6(G)c11, y8(A)c9, c8(G)z9, a8(G)x9, c7(G)z10, a7(G)x10, a9(A)w8, x8(A)b9, w8(A)a9, b9(A)x8, z8(A)d9	J3
343.014588	303.8	$C_{10}H_{11}N_5O_5PS$	343.014575	-0.040	-1	w6b11 - H2O, d7(G)z10 - 1H, c7(G)y10 - 1H, z6(G)d11 - 1H, b7(G)x10 - 1H, a7(G)w10 - 1H, d8(G)z9 - 1H, x6(G)b11 - 1H, y6(G)c11 - 1H, w8b9 - 2H, y8(A)d9 - 1H, w6(G)a11 - 1H, a8(G)w9 - 1H, b9(A)w8 - 1H, b8(G)x9 - 1H, c8(G)y9 - 1H	J4
360.042496	44.3	$C_{12}H_{15}O_6N_3PS$	360.042467	-0.080	-1	x1 - 2H, c1 - 2H, d1 - H2O, w1 - H2O	
361.026562	68.3	$C_{12}H_{14}O_7N_2PS$	361.026482	-0.220	-1	w1 - NH3, d1 - NH3	
370.038128	95.1	$C_{12}H_{14}N_5O_5PS$	370.038050	-0.210	-1	c3(dA)y14, a3(dA)w14,	J6

						w3(dA)a14, y3(dA)c14, z2(dG)c15, b3(dA)x14, z3(dA)d14, x3(dA)b14, d3(dA)z14	
378.053085	105.8	$C_{12}H_{17}O_7N_3PS$	378.053032	-0.140	-1	d1, w1	
386.032973	114.9	$C_{12}H_{14}N_5O_6PS$	386.032965	-0.020	-1	b3(dA)w14, z2(dG)d15, y2(dG)c15, x2(dG)b15, w3b14 - 1H, w2(dG)a15, y3(dA)d14, d3(dA)y14	J7
398.982326	98.2	$C_8H_{16}O_{12}PS_2$	398.982629	0.760	-1		
414.959499	83.3	$C_{10}H_{14}N_2O_8P_2S_2$	414.959405	-0.230	-1	c6(T)w11, d4(T)x13, d6(T)x11, c4(T)w13, w7(T)c10, c5(T)w12, d5(T)x12, w5(T)c12	J8
423.988720	160.6	$C_9H_{18}NO_{10}P_4$	423.988667	0.130	-1		
439.965913	118.1	$C_{10}H_{13}N_5O_7P_2S_2$	439.965888	-0.060	-1	d8(G)x9, w6(G)c11, c7(G)w10, w8(A)d9, d7(G)x10, c8(G)w9	J9
450.004400	171.2	$C_{11}H_{20}NO_{10}P_4$	450.004317	0.190	-1		
450.004488	70.5	$C_{11}H_{20}NO_{10}P_4$	450.004317	0.380	-1		
465.981595	107.3	$C_{12}H_{15}N_5O_7P_2S_2$	465.981538	-0.120	-1	d3(dA)x14, w3c14, c3(dA)w14	J10
481.976495	78.0	$C_{12}H_{15}N_5O_8P_2S_2$	481.976452	-0.090	-1	d3w14, w2(dG)c15, w3(dA)d14	J11
484.387651	47.9	$C_{46}H_{57}N_{16}O_{23}P_4S_4$	484.387742	0.190	-3	w4	
512.996331	115.6	$C_{17}H_{26}O_4P_5S_2$	512.996610	0.540	-1		
554.558523	156.9	$C_{36}H_{44}O_{19}N_{10}P_3S_3$	554.558508	-0.030	-2	d3 - 3H	
566.557816	60.3	$C_{36}H_{42}O_{18}N_{13}P_3S_3$	566.557837	0.040	-2	w3 - 4H	
567.565906	42.0	$C_{36}H_{44}O_{18}N_{13}P_3S_3$	567.565662	-0.430	-2	w3	
591.062499	33.8	$C_{56}H_{70}N_{18}O_{29}P_5S_5$	591.062140	-0.610	-3	w5	
595.075718	53.1	$C_{41}H_{49}N_{10}O_{20}P_3S_3$	595.075528	-0.320	-2	y4 - B(dG) - NH3, b4 - B(T) - H2O, a4 - B(T)	
603.302333	5.4	$C_{76}H_{94}N_{21}O_{42}P_7S_7$	603.302387	0.090	-4	d7	
609.308403	104.9	$C_{76}H_{94}N_{25}O_{40}P_7S_7$	609.308004	-0.650	-4	w7	
639.039335	59.3	$C_{25}H_{20}O_{18}P$	639.039274	0.100	-1		
644.143460	66.5	$C_{24}H_{31}O_{12}N_5PS$	644.143303	-0.240	-1	b2	
651.744552	8.1	$C_9H_{15}NO_7P_{13}$	651.744306	0.380	-1		
651.744755	48.8	$C_{66}H_{80}N_{16}O_{34}P_5S_5$	651.744365	-0.600	-3	b6 - H2O, a6	
657.593238	234.3	$C_{46}H_{54}N_{12}O_{22}P_3S_3$	657.593080	-0.240	-2	a4 - 3H	
658.097591	90.1	$C_{46}H_{55}N_{12}O_{22}P_3S_3$	658.096992	-0.910	-2	b4 - H2O, a4	
659.311877	43.4	$C_{167}H_{203}N_{51}O_{88}P_{15}S_1$	659.311204	-	-8	M - 9H - A, M -	

		5		1.020		9H - dA
664.045786	113.5	$C_{15}H_{18}N_7O_{23}$	664.045953	0.250	-1	
674.091273	9.1	$C_{66}H_{81}N_{23}O_{32}P_5S_5$	674.090870	-0.600	-3	y6
676.068145	888.2	$C_{172}H_{207}N_{56}O_{88}P_{15}S_{15}$	676.067037	-1.640	-8	M - 10H
679.113654	27.1	$C_{46}H_{57}N_{16}O_{21}P_3S_3$	679.113508	-0.220	-2	y4
682.226086	28.9	$C_{126}H_{153}N_{44}O_{66}P_{12}S_{12}$	682.225023	-1.560	-6	w12 - 7H
689.560130	25.1	$C_{86}H_{106}N_{26}O_{47}P_8S_8$	689.559806	-0.470	-4	d8
691.064015	18.8	$C_{106}H_{129}N_{40}O_{54}P_{10}S_{10}$	691.063771	-0.350	-5	w10
691.567183	38.9	$C_{86}H_{106}N_{30}O_{44}P_8S_8$	691.566694	-0.710	-4	w8
705.399926	18.8	$C_{66}H_{80}N_{23}O_{34}P_6S_6$	705.400149	0.320	-3	w6 - 5H
706.072882	22.6	$C_{66}H_{82}N_{23}O_{34}P_6S_6$	706.072032	-1.200	-3	w6
709.566728	16.5	$C_{91}H_{110}N_{26}O_{48}P_8S_8$	709.566360	-0.520	-4	b9 - B(A) - H2O, a9 - B(A)
716.405055	13.7	$C_{71}H_{87}N_{16}O_{38}P_6S_6$	716.404454	-0.840	-3	a7 - B(G)
722.076174	75.3	$C_{24}H_{30}O_{13}N_5P_2S_2$	722.076226	0.070	-1	d2 - H2O, c2 - 2H
727.085530	70.0	$C_{46}H_{58}N_{16}O_{23}P_4S_4$	727.085251	-0.380	-2	w4
732.072024	14.8	$C_{24}H_{28}O_{12}N_7P_2S_2$	732.071809	-0.290	-1	x2 - NH2
735.207237	9.7	$C_{160}H_{195}O_{84}N_{53}P_{15}S_{15}$	735.207567	0.450	-7	w15, d15
740.086718	36.5	$C_{24}H_{32}O_{14}N_5P_2S_2$	740.086791	0.100	-1	d2
743.079448	94.2	$C_{96}H_{114}N_{31}O_{48}P_8S_8$	743.078027	-1.910	-4	a9 - 5H
747.082583	62.3	$C_{24}H_{29}O_{12}N_8P_2S_2$	747.082708	0.170	-1	w2 - H2O, x2 - 2H
749.084961	17.4	$C_{96}H_{114}N_{35}O_{46}P_8S_8$	749.083644	-1.760	-4	z9 - 5H
750.063031	7.0	$C_{116}H_{141}N_{38}O_{62}P_{11}S_{11}$	750.062351	-0.910	-5	d11 - 6H
751.500569	11.1	$C_{167}H_{205}N_{51}O_{87}P_{15}S_{15}$	751.501402	1.110	-7	M - 7H - G, M - 7H - dG
753.786050	15.1	$C_{167}H_{205}N_{51}O_{88}P_{15}S_{15}$	753.786390	0.450	-7	M - 7H - dA, M - 7H - A
753.838417	5.3	$C_{96}H_{117}N_{35}O_{47}P_8S_8$	753.838241	-0.230	-4	y9
754.867520	9.0	$C_{116}H_{141}N_{42}O_{60}P_{11}S_{11}$	754.866845	-0.890	-5	w11 - 6H
765.093245	164.7	$C_{24}H_{31}O_{13}N_8P_2S_2$	765.093273	0.040	-1	w2
766.754841	143.0	$C_{76}H_{92}N_{21}O_{39}P_6S_6$	766.754257	-0.760	-3	a7, b7 - H2O
772.792573	211.2	$C_{172}H_{208}N_{56}O_{88}P_{15}S_{15}$	772.791939	-0.820	-7	M - 9H
774.762409	16.0	$C_{76}H_{92}N_{25}O_{37}P_6S_6$	774.761746	-0.860	-3	y7 - H2O, z7
777.573549	35.6	$C_{96}H_{117}N_{35}O_{49}P_9S_9$	777.572157	-1.790	-4	w9 - 5H
780.765759	15.7	$C_{76}H_{94}N_{25}O_{38}P_6S_6$	780.765268	-0.630	-3	y7
791.277281	129.4	$C_{126}H_{151}N_{40}O_{65}P_{11}S_{11}$	791.276180	-1.390	-5	a12 - 6H

796.282183	8.8	$C_{126}H_{152}N_{44}O_{63}P_{11}S_{11}$	796.282238	0.070	-5	y12 - H2O, z12
804.739567	15.7	$C_{76}H_{95}N_{21}O_{42}P_{7}S_{7}$	804.738941	-0.780	-3	d7
812.747049	67.8	$C_{76}H_{95}N_{25}O_{40}P_{7}S_{7}$	812.746430	-0.760	-3	w7
818.108812	85.4	$C_{56}H_{68}N_{14}O_{28}P_{4}S_{4}$	818.108589	-0.270	-2	b5 - H2O, a5
819.072842	210.4	$C_{126}H_{155}N_{44}O_{66}P_{12}S_{12}$	819.073048	0.250	-5	w12
823.085229	40.3	$C_{106}H_{127}N_{33}O_{54}P_{9}S_{9}$	823.083826	-1.700	-4	a10 - 5H
830.120351	70.1	$C_{56}H_{68}N_{18}O_{26}P_{4}S_{4}$	830.119822	-0.640	-2	y5 - H2O, z5
831.414997	15.4	$C_{81}H_{99}N_{21}O_{43}P_{7}S_{7}$	831.414346	-0.780	-3	a8 - B(G)
833.276084	7.7	$C_{131}H_{160}N_{41}O_{69}P_{12}S_{12}$	833.275978	-0.130	-5	y13 - B(A) - H2O, z13 - B(A), b13 - 7H
835.593335	9.9	$C_{106}H_{127}N_{40}O_{51}P_{9}S_{9}$	835.593020	-0.380	-4	z10, y10 - H2O
839.125443	12.8	$C_{56}H_{70}N_{18}O_{27}P_{4}S_{4}$	839.125105	-0.400	-2	y5
840.095798	42.7	$C_{106}H_{129}N_{40}O_{52}P_{9}S_{9}$	840.095661	-0.160	-4	y10
851.824789	24.3	$C_{106}H_{131}N_{33}O_{57}P_{10}S_{10}$	851.824295	-0.580	-4	d10
855.084729	13.2	$C_{136}H_{165}N_{43}O_{70}P_{12}S_{12}$	855.084015	-0.830	-5	a13 - 6H
860.286768	230.1	$C_{136}H_{165}N_{46}O_{69}P_{12}S_{12}$	860.286877	0.130	-5	z13, y13 - H2O
863.578875	16.5	$C_{106}H_{128}N_{40}O_{54}P_{10}S_{10}$	863.577620	-1.450	-4	w10 - 6H
866.080330	5.2	$C_{56}H_{69}N_{14}O_{30}P_{5}S_{5}$	866.080333	0.000	-2	c5 - 3H, d5 - H2O
870.103012	11.9	$C_{31}H_{34}O_{15}N_{7}P_{2}S_{2}$	870.103503	0.560	-1	z3 - B(dA) - NH3, a3 - 3H
871.328786	6.4	$C_{111}H_{133}N_{33}O_{58}P_{10}S_{10}$	871.326936	-2.120	-4	a11 - 6H, a11 - B(A) - H2O
875.086573	12.6	$C_{56}H_{71}N_{14}O_{31}P_{5}S_{5}$	875.085615	-1.090	-2	d5
876.582999	32.2	$C_{167}H_{204}N_{51}O_{87}P_{15}S_{15}$	876.583574	0.660	-6	M - 8H - dG, M - 6H - H2O - A, M - 8H - G, M - 6H - H2O - dA
877.672545	10.2	$C_{136}H_{167}N_{43}O_{73}P_{13}S_{13}$	877.673261	0.820	-5	d13 - 7H
879.249058	18.7	$C_{167}H_{204}N_{51}O_{88}P_{15}S_{15}$	879.249393	0.380	-6	M - 8H - dA, M - 8H - A
881.429630	106.8	$C_{86}H_{103}N_{26}O_{44}P_{7}S_{7}$	881.428208	-1.610	-3	a8 - 4H
882.093894	29.4	$C_{86}H_{103}N_{25}O_{45}P_{7}S_{7}$	882.092155	-1.970	-3	b8 - NH3
884.440406	75.2	$C_{86}H_{104}N_{30}O_{41}P_{7}S_{7}$	884.440000	-0.460	-3	y8 - H2O, z8
887.097547	156.8	$C_{56}H_{71}N_{18}O_{29}P_{5}S_{5}$	887.096848	-0.790	-2	w5
901.758316	1240.9	$C_{172}H_{209}N_{56}O_{88}P_{15}S_{15}$	901.758475	0.180	-6	M - 8H
908.420078	11.7	$C_{84}H_{105}N_{30}O_{43}P_{8}S_{8}$	908.421163	1.190	-3	w8 - OC2H2
909.341915	32.6	$C_{116}H_{139}N_{38}O_{59}P_{10}S_{10}$	909.341245	-0.740	-4	a11 - 5H

915.347980	17.5	$C_{116}H_{139}N_{42}O_{57}P_{10}S_{10}$	915.346862	-1.220	-4	z11 - 5H
919.078457	12.1	$C_{86}H_{105}N_{26}O_{47}P_8S_8$	919.076950	-1.640	-3	d8 - 5H
922.425236	56.4	$C_{86}H_{107}N_{30}O_{44}P_8S_8$	922.424684	-0.600	-3	w8
943.836443	17.2	$C_{116}H_{142}N_{42}O_{60}P_{11}S_{11}$	943.835375	-1.130	-4	w11 - 5H
946.090206	10.9	$C_{91}H_{110}N_{26}O_{48}P_8S_8$	946.088297	-2.020	-3	a9 - 4H
978.120869	11.3	$C_{23}H_{62}N_9O_5P_{14}$	978.120608	0.270	-1	
978.121170	76.1	$C_{66}H_{81}N_{16}O_{34}P_5S_5$	978.120186	-1.010	-2	b6 - H2O, a6
979.118137	14.7	$C_{66}H_{81}N_{15}O_{35}P_5S_5$	979.116107	-2.070	-2	b6 - NH2
984.069143	84.8	$C_{60}H_{74}N_{18}O_{34}P_6S_6$	984.068789	-0.360	-2	w6 - B(dA) - CH4
987.125573	6.9	$C_{66}H_{83}N_{16}O_{35}P_5S_5$	987.125469	-0.110	-2	b6
989.096010	18.6	$C_{126}H_{151}N_{40}O_{65}P_{11}S_{11}$	989.095087	-0.930	-4	a12 - 6H
991.107283	28.7	$C_{96}H_{115}N_{31}O_{48}P_8S_8$	991.106462	-0.830	-3	a9 - 4H
992.107347	11.5	$C_{96}H_{116}N_{30}O_{49}P_8S_8$	992.106350	-1.010	-3	b9 - NH2
1005.453424	6.9	$C_{96}H_{118}N_{35}O_{47}P_8S_8$	1005.453414	-0.010	-3	y9
1011.640013	9.6	$C_{66}H_{82}N_{23}O_{32}P_5S_5$	1011.639943	-0.070	-2	y6
1015.188877	38.5	$C_{36}H_{45}O_{17}N_{10}P_2S_2$	1015.188630	-0.240	-1	b3
1017.584330	5.3	$C_{126}H_{154}N_{40}O_{68}P_{12}S_{12}$	1017.583600	-0.720	-4	d12 - 6H
1022.184624	15.6	$C_{36}H_{42}O_{15}N_{13}P_2S_2$	1022.184547	-0.080	-1	y3 - H2O, z3
1023.589122	20.7	$C_{126}H_{154}N_{44}O_{66}P_{12}S_{12}$	1023.589217	0.090	-4	w12 - 6H
1026.092807	6.2	$C_{66}H_{82}N_{16}O_{36}P_6S_6$	1026.091930	-0.850	-2	c6 - 3H, d6 - H2O
1029.290242	12.5	$C_{160}H_{195}O_{84}N_{53}P_{15}S_{15}$	1029.290374	0.130	-5	w15 - 7H, d15 - 7H
1035.098077	13.7	$C_{66}H_{84}N_{16}O_{37}P_6S_6$	1035.097212	-0.840	-2	d6
1037.100473	27.5	$C_{96}H_{118}N_{35}O_{49}P_9S_9$	1037.098635	-1.770	-3	w9 - 4H
1040.195332	54.0	$C_{36}H_{44}O_{16}N_{13}P_2S_2$	1040.195112	-0.210	-1	y3
1059.611495	31.9	$C_{66}H_{83}N_{23}O_{34}P_6S_6$	1059.611687	0.180	-2	w6
1068.854587	9.1	$C_{136}H_{165}N_{43}O_{70}P_{12}S_{12}$	1068.854882	0.280	-4	a13 - 6H
1075.111214	72.7	$C_{71}H_{88}N_{16}O_{38}P_6S_6$	1075.110320	-0.830	-2	b7 - B(G) - H2O, a7 - B(G)
1075.607996	20.2	$C_{136}H_{166}N_{46}O_{69}P_{12}S_{12}$	1075.610415	2.250	-4	y13 - H2O, z13
1082.310489	7.0	$C_{172}H_{210}N_{56}O_{88}P_{15}S_{15}$	1082.311626	1.050	-5	M - 7H
1082.511374	11.5	$C_{172}H_{211}N_{56}O_{88}P_{15}S_{15}$	1082.513191	1.680	-5	M - 6H
1093.12265	6.5	$C_{36}H_{44}O_{18}N_{10}P_3S_3$	1093.12155	-	-1	c3 - 2H, d3 - H2O

2			3	1.010		
1097.447004	17.6	$C_{106}H_{127}N_{33}O_{54}P_9S_9$	1097.444918	-1.900	-3	a10 - 5H
1111.131984	60.8	$C_{36}H_{46}O_{19}N_{10}P_3S_3$	1111.132119	0.120	-1	d3
1114.124144	5.7	$C_{106}H_{127}N_{40}O_{51}P_9S_9$	1114.123843	-0.270	-3	z10 - 4H
1118.128198	36.1	$C_{36}H_{43}O_{17}N_{13}P_3S_3$	1118.128036	-0.140	-1	w3 - H2O, x3 - 2H
1118.128440	13.1	$C_{36}H_{43}O_{17}N_{13}P_3S_3$	1118.128036	-0.360	-1	w3 - H2O, x3 - 2H
1136.138881	204.5	$C_{36}H_{45}O_{18}N_{13}P_3S_3$	1136.138600	-0.250	-1	w3
1149.629852	14.1	$C_{76}H_{91}N_{21}O_{39}P_6S_6$	1149.627199	-2.310	-2	a7 - 4H
1150.635260	46.9	$C_{76}H_{93}N_{21}O_{39}P_6S_6$	1150.635024	-0.210	-2	b7 - H2O, a7
1151.774132	7.5	$C_{106}H_{129}N_{40}O_{54}P_{10}S_{10}$	1151.772585	-1.340	-3	w10 - 5H
1162.646106	9.2	$C_{76}H_{93}N_{25}O_{37}P_6S_6$	1162.646258	0.130	-2	z7, y7 - H2O
1171.650797	10.8	$C_{76}H_{95}N_{25}O_{38}P_6S_6$	1171.651540	0.630	-2	y7
1191.158307	46.8	$C_{41}H_{50}N_{10}O_{20}P_3S_3$	1191.158333	0.020	-1	b4 - B(T) - H2O, a4 - B(T), y4 - B(dG) - NH3
1212.456499	7.0	$C_{116}H_{139}N_{38}O_{59}P_{10}S_{10}$	1212.454811	-1.390	-3	a11 - 5H
1216.164854	13.1	$C_{41}H_{49}N_{13}O_{19}P_3S_3$	1216.164816	-0.030	-1	y4 - B(mC) - H2O, z4 - B(mC)
1218.617852	8.1	$C_{76}H_{94}N_{25}O_{40}P_7S_7$	1218.615459	-1.960	-2	w7 - 4H
1219.623741	26.4	$C_{76}H_{96}N_{25}O_{40}P_7S_7$	1219.623284	-0.370	-2	w7
1247.623771	12.8	$C_{81}H_{100}N_{21}O_{43}P_7S_7$	1247.625158	1.110	-2	a8 - B(G), b8 - B(G) - H2O
1258.782897	6.6	$C_{116}H_{143}N_{42}O_{60}P_{11}S_{11}$	1258.782925	0.020	-3	w11 - 4H
1317.202472	71.2	$C_{46}H_{56}N_{12}O_{22}P_3S_3$	1317.201261	-0.920	-1	b4 - H2O, a4
1319.195835	8.8	$C_{46}H_{56}N_{11}O_{23}P_3S_3$	1319.193101	-2.070	-1	b4 - NH2
1322.647289	11.4	$C_{86}H_{104}N_{26}O_{44}P_7S_7$	1322.645950	-1.010	-2	a8 - 3H
1335.212568	8.6	$C_{46}H_{58}N_{12}O_{23}P_3S_3$	1335.211825	-0.560	-1	b4
1341.225921	9.6	$C_{46}H_{56}N_{16}O_{20}P_3S_3$	1341.223727	-1.640	-1	y4 - H2O, z4
1359.234300	10.2	$C_{46}H_{58}N_{16}O_{21}P_3S_3$	1359.234292	-0.010	-1	y4
1384.139056	9.8	$C_{86}H_{108}N_{30}O_{44}P_8S_8$	1384.140665	1.160	-2	w8
1413.145275	13.1	$C_{46}H_{57}N_{12}O_{24}P_4S_4$	1413.144748	-0.370	-1	d4 - H2O, c4 - 2H
1431.155028	6.7	$C_{46}H_{59}N_{12}O_{25}P_4S_4$	1431.155312	0.200	-1	d4
1455.177389	27.3	$C_{46}H_{59}N_{16}O_{23}P_4S_4$	1455.177779	0.270	-1	w4
1637.224401	14.4	$C_{56}H_{69}N_{14}O_{28}P_4S_4$	1637.224454	0.030	-1	b5 - H2O, a5

1650.143453	9.0	$C_{51}H_{65}N_{15}O_{28}P_5S_5$	1650.142061	-0.840	-1	w5 - B(mC)
1775.200496	11.7	$C_{56}H_{72}N_{18}O_{29}P_5S_5$	1775.200973	0.270	-1	w5
			Abs mean error	0.703		
			Abs mean std dev	0.616		

Table S9 showing peak list, signal-to-noise ratio (S/N), elemental composition, and possible assignments with mass errors (ppm) of the autocorrelation line of danvatirsen by 2D-EDD-MS.

Measured *mz*	S/N	Elemental Composition	Theoretical *mz*	Error (ppm)	Charge	Assignment
319.011609	21.6	$C_{31}H_{36}O_{19}N_4P_3S_3$	319.012174	1.770	-3	d3 - B(dA) - NH3
319.016031	217.3	$C_{10}H_{13}N_2O_6PS$	319.015918	-0.350	-1	bTx
327.019861	40.3	$C_{31}H_{36}O_{17}N_8P_3S_3$	327.019846	-0.050	-3	d3 - 5H, x3 - 4H, w3 - B(dA) - H2O
343.014726	149.6	$C_{10}H_{11}N_5O_5PS$	343.014575	-0.440	-1	bGy
344.022551	141.0	$C_{10}H_{12}N_5O_5PS$	344.022400	-0.440	-1	cGy
360.042633	26.3	$C_{12}H_{15}O_6N_3PS$	360.042467	-0.460	-1	x1 - 2H, c1 - 2H, d1 - H2O, w1 - H2O
361.026830	28.4	$C_{24}H_{28}O_{14}N_4P_2S_2$	361.026757	-0.200	-2	d2 - NH3
370.038402	32.9	$C_{61}H_{71}N_{18}O_{30}P_5S_5$	370.038161	-0.650	-5	z6 - 6H
377.045457	27.1	$C_{49}H_{60}N_{16}O_{24}P_4S_4$	377.045541	0.220	-4	y5 - B(T) - OC2H2
378.053268	86.1	$C_{12}H_{17}O_7N_3PS$	378.053032	-0.620	-1	d1, w1
386.033273	34.6					
398.982483	154.5					
414.959681	99.6					
423.988989	94.1					
439.966411	60.3					
450.004856	99.1					
465.981949	87.7					
484.388097	261.1	$C_{46}H_{57}N_{16}O_{23}P_4S_4$	484.387925	-0.360	-3	w4
512.996699	245.1					
529.302659	100.9	$C_{66}H_{81}N_{23}O_{34}P_6S_6$	529.302342	-0.600	-4	w6
538.003140	126.7					
540.853737	61.9	$C_{172}H_{207}N_{56}O_{88}P_{15}S_{15}$	540.853740	0.010	-10	M-10H
554.559063	60.4	$C_{36}H_{44}O_{19}N_{10}P_3S_3$	554.558782	-0.510	-2	d3 - 3H
567.566334	132.7	$C_{36}H_{44}O_{18}N_{13}P_3S_3$	567.565936	-0.700	-2	w3
591.062641	65.9	$C_{56}H_{70}N_{18}O_{29}P_5S_5$	591.062323	-0.540	-3	w5
595.076172	43.2	$C_{41}H_{49}N_{10}O_{20}P_3S_3$	595.075802	-0.620	-2	y4 - B(dG) - NH3, b4 - B(T) - H2O, a4 - B(T)
601.060434	282.4	$C_{172}H_{208}N_{56}O_{88}P_{15}S_{15}$	601.060519	0.140	-9	M-9H
603.725617	140.4	$C_{61}H_{72}N_{14}O_{31}P_5S_5$	603.726534	1.520	-3	a6 - B(T) - H2O
604.059080	146.5	$C_{59}H_{75}N_{11}O_{35}P_5S_5$	604.058021	-1.750	-3	b6 - B(dA) - C2H2
606.055353	63.8	$C_{61}H_{73}N_{11}O_{34}P_5S_5$	606.054500	-1.410	-3	a6 - 5H
606.388959	24.3	$C_{115}H_{136}N_{38}O_{60}P_{10}S_{10}$	606.389586	1.030	-6	b11 - CH4
609.308611	130.8	$C_{76}H_{94}N_{25}O_{40}P_7S_7$	609.308141	-0.770	-4	w7
609.559517	78.2	$C_{116}H_{135}N_{42}O_{57}P_{10}S_{10}$	609.559541	0.040	-6	z11 - 9H
610.058898	28.4	$C_{61}H_{73}N_{13}O_{33}P_5S_5$	610.058244	-1.070	-3	a6 - B(dC), b6 - B(dC) - H2O, b6 - B(T) - NH3
622.058333	21.4	$C_{96}H_{117}N_{35}O_{49}P_9S_9$	622.057945	-0.620	-5	w9
629.055744	16.1	$C_{116}H_{141}N_{42}O_{60}P_{11}S_{11}$	629.055795	0.080	-6	w11
638.056137	13.1	$C_{100}H_{117}N_{35}O_{51}P_9S_9$	638.055911	-0.350	-5	z10 - B(A) - CH4
639.040176	29.4					
644.143859	27.3	$C_{24}H_{31}O_{12}N_5PS$	644.143852	-0.010	-1	b2
647.043640	23.1	$C_{41}H_{52}N_7O_{25}P_4S_4$	647.043132	-0.780	-2	d4 - 3H
651.409120	19.7	$C_{66}H_{79}N_{16}O_{34}P_5S_5$	651.408607	-0.790	-3	a6 - 4H
651.744654	42.3	$C_{66}H_{80}N_{16}O_{34}P_5S_5$	651.744548	-0.160	-3	b6 - H2O, a6
656.048613	11.7	$C_{80}H_{96}N_{28}O_{42}P_8S_8$	656.048274	-0.520	-4	w8 - B(T) - CH4
657.593725	251.9	$C_{46}H_{54}N_{12}O_{22}P_3S_3$	657.593354	-0.560	-2	a4 - 3H
658.096335	143.0	$C_{46}H_{55}N_{12}O_{22}P_3S_3$	658.096992	1.000	-2	b4 - H2O, a4
658.588802	17.6	$C_{46}H_{54}N_{11}O_{23}P_3S_3$	658.589000	0.300	-2	b4 - NH3

658.598913	49.3					
659.092455	11.5	$C_{46}H_{55}N_{11}O_{23}P_3S_3$	659.092912	0.690	-2	b4 - NH2
660.819106	24.9	$C_{86}H_{102}N_{26}O_{44}P_7S_7$	660.819337	0.350	-4	a8 - 5H
661.070612	38.3	$C_{86}H_{103}N_{26}O_{44}P_7S_7$	661.071293	1.030	-4	a8, b8 - H2O
661.570282	16.6	$C_{86}H_{103}N_{25}O_{45}P_7S_7$	661.569390	-1.350	-4	b8 - NH2
664.046582	95.9					
665.051031	11.7	$C_{143}H_{171}O_{75}N_{49}P_{14}S_{14}$	665.050479	-0.830	-7	w14 - 10H
668.552340	14.1					
670.108765	15.8	$C_{46}H_{55}N_{16}O_{20}P_3S_3$	670.108499	-0.400	-2	y4 - H2O, z4
675.198922	10.0	$C_{148}H_{178}O_{77}N_{47}P_{14}S_{14}$	675.198830	-0.140	-7	c14 - NH2
676.319005	461.9	$C_{172}H_{209}N_{56}O_{88}P_{15}S_{15}$	676.318994	-0.020	-8	M-8H
677.694657	46.5					
679.192056	400.0					
682.393701	11.6	$C_{126}H_{154}N_{44}O_{66}P_{12}S_{12}$	682.393085	-0.900	-6	w12
682.814854	13.8	$C_{86}H_{103}N_{30}O_{42}P_8S_8$	682.813505	-1.970	-4	x8 - H2O
683.059470	14.9	$C_{86}H_{102}N_{29}O_{43}P_8S_8$	683.059509	0.060	-4	x8 - NH3
683.559509	8.3	$C_{85}H_{102}N_{30}O_{43}P_8S_8$	683.560140	0.920	-4	x8 - CH3
684.060317	112.5	$C_{106}H_{126}N_{40}O_{52}P_{10}S_{10}$	684.061110	1.160	-5	x10 - H2O
684.559706	74.2	$C_{84}H_{102}N_{30}O_{44}P_8S_8$	684.559006	-1.020	-4	w8 - C2H4
685.058545	18.9	$C_{86}H_{104}N_{26}O_{46}P_8S_8$	685.057302	-1.810	-4	d8 - H2O, c8 - 5H
685.805414	8.8	$C_{85}H_{103}N_{26}O_{47}P_8S_8$	685.804074	-1.950	-4	d8 - CH3
689.729726	12.0	$C_{66}H_{83}N_{16}O_{37}P_6S_6$	689.729232	-0.720	-3	d6
691.063386	10.4	$C_{106}H_{129}N_{40}O_{54}P_{10}S_{10}$	691.063771	0.560	-5	w10
691.567090	68.1	$C_{86}H_{106}N_{30}O_{44}P_8S_8$	691.566831	-0.370	-4	w8
692.067493	54.5	$C_{64}H_{80}N_{23}O_{33}P_6S_6$	692.068511	1.470	-3	w6 - OC2H2
706.072783	27.2	$C_{66}H_{82}N_{23}O_{34}P_6S_6$	706.072215	-0.800	-3	w6
712.736751	8.1	$C_{134}H_{164}N_{46}O_{69}P_{12}S_{12}$	712.737851	1.540	-6	y13 - OC2H2
715.074533	23.5	$C_{46}H_{58}N_{12}O_{25}P_4S_4$	715.074292	-0.340	-2	d4
722.076030	21.3	$C_{24}H_{30}O_{13}N_5P_2S_2$	722.076226	0.270	-1	d2 - H2O, c2 - 2H
727.085857	41.7	$C_{46}H_{58}N_{16}O_{23}P_4S_4$	727.085525	-0.460	-2	w4
727.271462	7.1	$C_{116}H_{138}N_{38}O_{59}P_{10}S_{10}$	727.271541	0.110	-5	a11 - 6H
727.472231	16.2	$C_{116}H_{139}N_{38}O_{59}P_{10}S_{10}$	727.473106	1.200	-5	a11, b11 - H2O
727.872499	13.5	$C_{116}H_{139}N_{37}O_{60}P_{10}S_{10}$	727.871584	-1.260	-5	b11 - NH2
732.071730	5.4	$C_{24}H_{28}O_{12}N_7P_2S_2$	732.071809	0.110	-1	x2 - NH2
740.087631	18.2	$C_{24}H_{32}O_{14}N_5P_2S_2$	740.087340	-0.390	-1	d2
743.078355	13.6	$C_{96}H_{114}N_{31}O_{48}P_8S_8$	743.078164	-0.260	-4	a9 - 5H
743.329545	41.9	$C_{96}H_{115}N_{31}O_{48}P_8S_8$	743.329984	0.590	-4	b9 - H2O, a9
743.580405	26.4	$C_{49}H_{61}N_{11}O_{27}P_4S_4$	743.579407	-1.340	-2	b5 - B(dC) - OC2H2
747.083523	17.6	$C_{50}H_{60}N_{13}O_{25}P_4S_4$	747.083654	0.180	-2	z5 - B(dG) - CH3
747.569553	12.6	$C_{51}H_{59}N_{10}O_{27}P_4S_4$	747.569771	0.290	-2	a5 - B(dC) - NH2
747.903351	6.0	$C_{141}H_{174}O_{75}N_{43}P_{13}S_{13}$	747.902000	-1.810	-6	b14 - B(dA) - OC2H2
749.076596	8.0	$C_{24}H_{29}O_{13}N_7P_2S_2$	749.075098	-2.000	-1	w2 - NH2
749.335900	13.0	$C_{96}H_{115}N_{35}O_{46}P_8S_8$	749.335737	-0.220	-4	z9, y9 - H2O
749.585787	12.4	$C_{49}H_{61}N_{13}O_{26}P_4S_4$	749.585024	-1.020	-2	y5 - B(dG) - C2H4, z5 - B(dA) - C2H2
753.786011	26.1	$C_{167}H_{205}N_{51}O_{88}P_{15}S_{15}$	753.786390	0.500	-7	M - 7H - dA, M - 7H - A
754.786605	16.5	$C_{167}H_{202}N_{54}O_{86}P_{15}S_{15}$	754.785885	-0.950	-7	M - 9H - T, M - 9H - dU
754.929698	12.4	$C_{167}H_{203}N_{54}O_{86}P_{15}S_{15}$	754.929782	0.110	-7	M - 8H - dU, M - 8H - T
755.088803	6.4	$C_{51}H_{62}N_{12}O_{26}P_4S_4$	755.087399	-1.860	-2	b5 - B(T) - H2O, y5 - B(dG) - NH3, a5 - B(T)
756.090214	18.6	$C_{50}H_{62}N_{13}O_{26}P_4S_4$	756.088936	-1.690	-2	y5 - B(dG) - CH3
756.232424	9.0	$C_{143}H_{172}O_{75}N_{45}P_{13}S_{13}$	756.233659	1.630	-6	b14 - 8H, y14 - B(dA) - NH3

756.376017	6.8	$C_{71}H_{84}N_{18}O_{40}P_7S_7$	756.377233	1.610	-3	c7 - 6H
757.068598	12.1	$C_{143}H_{169}O_{72}N_{49}P_{13}S_{13}$	757.067763	-1.100	-6	z14 - 9H
759.087846	6.9	$C_{51}H_{60}N_{15}O_{24}P_4S_4$	759.088997	1.520	-2	z5 - B(T) - NH2
765.094292	36.5	$C_{24}H_{31}O_{13}N_8P_2S_2$	765.093822	-0.620	-1	w2
766.418642	23.3	$C_{76}H_{91}N_{21}O_{39}P_6S_6$	766.418499	-0.190	-3	a7 - 4H
766.754132	81.9	$C_{76}H_{92}N_{21}O_{39}P_6S_6$	766.754257	0.160	-3	a7, b7 - H2O
768.088457	7.1	$C_{50}H_{60}N_{16}O_{25}P_4S_4$	768.088265	-0.250	-2	y5 - B(T) - CH4
771.227056	21.3	$C_{142}H_{171}O_{74}N_{49}P_{14}S_{14}$	771.226315	-0.960	-6	x14 - B(T) - CH3
773.079860	315.8	$C_{172}H_{210}N_{56}O_{88}P_{15}S_{15}$	773.079889	0.040	-7	M - 7H
774.223221	145.8	$C_{142}H_{171}O_{76}N_{48}P_{14}S_{14}$	774.224017	1.030	-6	w14 - B(mC) - CH3
774.760340	13.6	$C_{76}H_{92}N_{25}O_{37}P_6S_6$	774.761746	1.810	-3	y7 - H2O, z7
775.097355	7.6	$C_{51}H_{62}N_{16}O_{25}P_4S_4$	775.096090	-1.630	-2	y5 - 4H
777.077618	224.0	$C_{148}H_{179}O_{76}N_{48}P_{13}S_{13}$	777.076902	-0.920	-6	b14 - 8H
777.824516	21.1	$C_{96}H_{118}N_{35}O_{49}P_9S_9$	777.824250	-0.340	-4	w9
778.074925	16.3	$C_{148}H_{175}O_{74}N_{51}P_{13}S_{13}$	778.074918	-0.010	-6	z14 - 9H
783.069009	13.0	$C_{99}H_{121}N_{28}O_{54}P_9S_9$	783.068383	-0.800	-4	a10 - B(A) - C2H2
785.066365	22.3	$C_{74}H_{92}N_{21}O_{40}P_7S_7$	785.067840	1.880	-3	c7 - OC2H2
791.276129	6.9	$C_{126}H_{151}N_{40}O_{65}P_{11}S_{11}$	791.276180	0.070	-5	a12 - 6H
791.476939	27.1	$C_{126}H_{152}N_{40}O_{65}P_{11}S_{11}$	791.477745	1.020	-5	b12 - H2O, a12
791.877687	26.1	$C_{126}H_{152}N_{39}O_{66}P_{11}S_{11}$	791.876223	-1.850	-5	b12 - NH2
804.739029	8.5	$C_{76}H_{95}N_{21}O_{42}P_7S_7$	804.738941	-0.110	-3	d7
805.073927	8.4	$C_{100}H_{119}N_{37}O_{51}P_9S_9$	805.075064	1.410	-4	y10 - B(mC) - CH3
812.746917	40.4	$C_{76}H_{95}N_{25}O_{40}P_7S_7$	812.746613	-0.370	-3	w7
817.605408	36.4	$C_{56}H_{67}N_{14}O_{28}P_4S_4$	817.604950	-0.560	-2	a5 - 3H
818.108472	83.1	$C_{56}H_{68}N_{14}O_{28}P_4S_4$	818.108589	0.140	-2	b5 - H2O, a5
818.611143	32.7					
819.073146	39.5	$C_{126}H_{155}N_{44}O_{66}P_{12}S_{12}$	819.073158	0.010	-5	w12
819.873555	48.5	$C_{129}H_{159}N_{38}O_{69}P_{12}S_{12}$	819.872678	-1.070	-5	a13 - B(A) - OC2H2, a13 - B(G) - C2H2
823.084380	6.1	$C_{106}H_{127}N_{33}O_{54}P_9S_9$	823.083963	-0.510	-4	a10 - 5H
823.335441	24.8	$C_{106}H_{128}N_{33}O_{54}P_9S_9$	823.335782	0.410	-4	a10, b10 - H2O
823.834661	31.6	$C_{106}H_{128}N_{32}O_{55}P_9S_9$	823.833879	-0.950	-4	b10 - NH2
824.469533	7.3	$C_{129}H_{156}N_{41}O_{68}P_{12}S_{12}$	824.470735	1.460	-5	z13 - B(G) - C2H4, a13 - B(T) - C2H4
827.062913	10.1	$C_{155}H_{187}O_{82}N_{47}P_{15}S_{15}$	827.063591	0.820	-6	x15 - B(G) - NH3, c15 - B(dG) - NH3
828.563397	8.8	$C_{100}H_{120}N_{38}O_{52}P_{10}S_{10}$	828.563113	-0.340	-4	w10 - B(T) - CH4
829.082651	7.1	$C_{80}H_{96}N_{25}O_{40}P_7S_7$	829.082555	-0.120	-3	z8 - B(G) - CH3
830.120195	46.1	$C_{56}H_{68}N_{18}O_{26}P_4S_4$	830.120096	-0.120	-2	y5 - H2O, z5
835.593455	6.6	$C_{106}H_{127}N_{40}O_{51}P_9S_9$	835.593157	-0.360	-4	z10, y10 - H2O
837.081668	6.8	$C_{79}H_{98}N_{24}O_{43}P_7S_7$	837.081662	-0.010	-3	b8 - B(T) - C2H2
839.124861	5.5	$C_{56}H_{70}N_{18}O_{27}P_4S_4$	839.125105	0.290	-2	y5
840.095850	12.7	$C_{106}H_{129}N_{40}O_{52}P_9S_9$	840.095798	-0.060	-4	y10
852.074567	6.3	$C_{160}H_{193}O_{82}N_{53}P_{15}S_{15}$	852.074581	0.020	-6	c15 - H2O, x15 - H2O
855.284879	5.8	$C_{136}H_{166}N_{43}O_{70}P_{12}S_{12}$	855.285580	0.820	-5	a13, b13 - H2O
860.286765	34.6	$C_{136}H_{165}N_{46}O_{69}P_{12}S_{12}$	860.286877	0.130	-5	z13, y13 - H2O
860.484189	56.8	$C_{136}H_{164}N_{45}O_{70}P_{12}S_{12}$	860.483790	-0.460	-5	y13 - NH3
864.081945	11.7	$C_{106}H_{130}N_{40}O_{54}P_{10}S_{10}$	864.081669	-0.320	-4	w10
868.076643	6.9	$C_{110}H_{132}N_{33}O_{58}P_{10}S_{10}$	868.075117	-1.760	-4	a11 - B(G) - CH3
875.086203	16.7	$C_{56}H_{71}N_{14}O_{31}P_5S_5$	875.085889	-0.360	-2	d5
877.085364	8.7	$C_{56}H_{67}N_{18}O_{28}P_5S_5$	877.084015	-1.540	-2	x5 - 5H
877.588195	7.3	$C_{56}H_{68}N_{18}O_{28}P_5S_5$	877.587927	-0.300	-2	x5 - 4H
881.085550	5.4	$C_{167}H_{205}N_{54}O_{86}P_{15}S_{15}$	881.087262	1.940	-6	M - 6H - T, M - 6H - dU
881.250438	6.8	$C_{167}H_{204}N_{53}O_{87}P_{15}S_{15}$	881.251265	0.940	-6	M - 6H - dC, M

						- 6H - mC
881.429088	58.8	$C_{86}H_{103}N_{26}O_{44}P_7S_7$	881.428391	-0.790	-3	a8 - 4H
881.583007	5.4	$C_{109}H_{132}N_{40}O_{56}P_{10}S_{10}$	881.583039	0.040	-4	y11 - B(T) - C2H4
881.763510	68.2	$C_{86}H_{104}N_{26}O_{44}P_7S_7$	881.764149	0.720	-3	a8, b8 - H2O
883.431617	8.0	$C_{86}H_{101}N_{30}O_{41}P_7S_7$	883.432175	0.630	-3	z8 - 6H
883.578160	6.0	$C_{111}H_{130}N_{39}O_{56}P_{10}S_{10}$	883.578221	0.070	-4	z11 - 7H
884.440325	50.4	$C_{86}H_{104}N_{30}O_{41}P_7S_7$	884.440183	-0.160	-3	y8 - H2O, z8
887.097332	97.3	$C_{56}H_{71}N_{18}O_{29}P_5S_5$	887.097122	-0.240	-2	w5
887.598954	86.1					
901.757653	7.1	$C_{172}H_{209}N_{56}O_{88}P_{15}S_{15}$	901.758475	0.910	-6	M - 8H
901.924796	19.4	$C_{172}H_{210}N_{56}O_{88}P_{15}S_{15}$	901.926446	1.830	-6	M - 7H
902.094315	188.1	$C_{172}H_{211}N_{56}O_{88}P_{15}S_{15}$	902.094417	0.110	-6	M - 6H
905.588478	6.9	$C_{116}H_{138}N_{37}O_{59}P_{10}S_{10}$	905.588520	0.050	-4	a11 - NH2
906.092077	220.4	$C_{61}H_{73}N_{14}O_{31}P_5S_5$	906.093440	1.500	-2	a6 - B(T) - H2O
906.564274	9.1	$C_{111}H_{135}N_{35}O_{61}P_{11}S_{11}$	906.565030	0.830	-4	d11 - 5H
906.592128	223.2	$C_{59}H_{76}N_{11}O_{35}P_5S_5$	906.590670	-1.610	-2	b6 - B(dA) - C2H2
907.592153	7.1	$C_{114}H_{140}N_{38}O_{60}P_{10}S_{10}$	907.592067	-0.090	-4	b11 - C2H2
909.086355	285.7	$C_{143}H_{172}O_{72}N_{49}P_{13}S_{13}$	909.085902	-0.500	-5	z14 - 6H
910.089567	94.0	$C_{61}H_{75}N_{11}O_{34}P_5S_5$	910.089300	-0.290	-2	a6 - 3H
911.747919	6.2	$C_{85}H_{103}N_{30}O_{43}P_8S_8$	911.749279	1.490	-3	x8 - CH3
913.082720	61.6	$C_{84}H_{103}N_{30}O_{44}P_8S_8$	913.081100	-1.770	-3	w8 - C2H4
914.081927	5.3	$C_{86}H_{106}N_{26}O_{46}P_8S_8$	914.081437	-0.540	-3	c8
914.741032	13.0	$C_{85}H_{104}N_{26}O_{47}P_8S_8$	914.741008	-0.030	-3	d8 - CH3
916.578746	15.9	$C_{60}H_{72}N_{13}O_{34}P_5S_5$	916.580362	1.760	-2	b6 - B(dC) - CH4
918.740473	9.4	$C_{86}H_{104}N_{26}O_{47}P_8S_8$	918.741008	0.580	-3	d8 - 6H
919.076008	19.9	$C_{86}H_{105}N_{26}O_{47}P_8S_8$	919.076950	1.020	-3	d8 - 5H
919.749921	5.4	$C_{86}H_{107}N_{26}O_{47}P_8S_8$	919.749017	-0.980	-3	d8
922.424960	100.3	$C_{86}H_{107}N_{30}O_{44}P_8S_8$	922.424867	-0.100	-3	w8
924.092163	13.3	$C_{61}H_{75}N_{13}O_{34}P_5S_5$	924.092100	-0.070	-2	b6 - 3H
944.087042	12.0	$C_{116}H_{143}N_{42}O_{60}P_{11}S_{11}$	944.087331	0.310	-4	w11
945.088139	11.1	$C_{89}H_{107}N_{30}O_{46}P_8S_8$	945.088144	0.010	-3	z9 - B(A) - C2H4
946.424307	8.4	$C_{91}H_{111}N_{26}O_{48}P_8S_8$	946.424239	-0.070	-3	a9 - B(A), b9 - B(A) - H2O
950.083168	8.4	$C_{91}H_{108}N_{27}O_{48}P_8S_8$	950.084105	0.990	-3	b9 - B(dC) - NH3
977.617343	34.0	$C_{66}H_{80}N_{16}O_{34}P_5S_5$	977.616548	-0.810	-2	a6 - 3H
978.119841	86.4	$C_{66}H_{81}N_{16}O_{34}P_5S_5$	978.120186	0.350	-2	b6 - H2O, a6
984.069909	15.4	$C_{60}H_{74}N_{18}O_{34}P_6S_6$	984.069063	-0.860	-2	w6 - B(dA) - CH4
985.072345	6.6	$C_{90}H_{108}N_{32}O_{47}P_9S_9$	985.072868	0.530	-3	x9 - B(mC) - CH3
989.346629	5.2	$C_{126}H_{152}N_{40}O_{65}P_{11}S_{11}$	989.347044	0.420	-4	a12 - 5H
990.097992	19.1	$C_{126}H_{153}N_{39}O_{66}P_{11}S_{11}$	990.097097	-0.900	-4	b12 - NH2
991.106680	12.8	$C_{96}H_{115}N_{31}O_{48}P_8S_8$	991.106645	-0.040	-3	a9 - 4H
991.441706	32.2	$C_{96}H_{116}N_{31}O_{48}P_8S_8$	991.442404	0.700	-3	b9 - H2O, a9
992.073403	10.4	$C_{121}H_{146}N_{41}O_{65}P_{12}S_{12}$	992.072670	-0.740	-4	w12 - 7H
993.081008	15.4					
996.588782	8.4	$C_{61}H_{77}N_{21}O_{32}P_6S_6$	996.590223	1.450	-2	w6 - B(T)
999.451200	31.3	$C_{96}H_{116}N_{35}O_{46}P_8S_8$	999.450076	-1.120	-3	y9 - H2O, z9
1002.132275	8.7	$C_{66}H_{79}N_{23}O_{31}P_5S_5$	1002.131022	-1.250	-2	z6 - 3H
1002.634141	19.9	$C_{66}H_{80}N_{23}O_{31}P_5S_5$	1002.634661	0.520	-2	z6, y6 - H2O
1010.080030	8.4	$C_{125}H_{152}N_{40}O_{67}P_{12}S_{12}$	1010.080959	0.920	-4	c12 - CH3
1011.640641	9.4	$C_{66}H_{82}N_{23}O_{32}P_5S_5$	1011.640217	-0.420	-2	y6
1015.190122	6.9	$C_{36}H_{45}O_{17}N_{10}P_2S_2$	1015.189179	-0.930	-1	b3
1022.183983	11.2	$C_{36}H_{42}O_{15}N_{13}P_2S_2$	1022.184547	0.550	-1	y3 - H2O, z3
1024.093613	16.8	$C_{126}H_{156}N_{44}O_{66}P_{12}S_{12}$	1024.093267	-0.340	-4	w12
1025.094590	32.0	$C_{129}H_{160}N_{38}O_{69}P_{12}S_{12}$	1025.092667	-1.880	-4	a13 - B(A) - OC2H2, a13 - B(G) - C2H2
1030.839351	7.2	$C_{129}H_{157}N_{41}O_{68}P_{12}S_{12}$	1030.840238	0.860	-4	z13 - B(G) - C2H4, a13 -

						B(T) - C2H4
1035.098226	19.5	$C_{66}H_{84}N_{16}O_{37}P_6S_6$	1035.097486	-0.710	-2	d6
1037.099635	6.8	$C_{96}H_{118}N_{35}O_{49}P_9S_9$	1037.098818	-0.790	-3	w9 - 4H
1037.434876	39.4	$C_{96}H_{119}N_{35}O_{49}P_9S_9$	1037.434760	-0.110	-3	w9
1038.100170	56.6	$C_{129}H_{160}N_{44}O_{67}P_{12}S_{12}$	1038.099820	-0.340	-4	y13 - B(T) - OC2H2
1040.196102	20.4	$C_{36}H_{44}O_{16}N_{13}P_2S_2$	1040.195661	-0.420	-1	y3
1051.086725	12.9	$C_{100}H_{118}N_{30}O_{53}P_9S_9$	1051.086731	0.010	-3	a10 - B(dC) - CH4
1052.096373	13.2	$C_{165}H_{201}N_{53}O_{87}P_{15}S_{15}$	1052.096713	0.320	-5	M - 5H - C2H4 - dC, M - 5H - C2H4 - mC
1052.504927	6.1	$C_{167}H_{207}N_{51}O_{87}P_{15}S_{15}$	1052.504874	-0.050	-5	M - 5H - G, M - 5H - dG
1056.101745	5.7	$C_{101}H_{123}N_{31}O_{52}P_9S_9$	1056.102493	0.710	-3	b10 - B(T) - H2O, a10 - B(T)
1056.903430	6.3	$C_{167}H_{203}N_{54}O_{86}P_{15}S_{15}$	1056.901585	-1.750	-5	M - 8H - dU, M - 8H - T
1059.109268	8.4	$C_{66}H_{82}N_{23}O_{34}P_6S_6$	1059.108048	-1.150	-2	w6 - 3H
1059.612243	61.7	$C_{66}H_{83}N_{23}O_{34}P_6S_6$	1059.611961	-0.270	-2	w6
1069.358294	12.3	$C_{136}H_{167}N_{43}O_{70}P_{12}S_{12}$	1069.358794	0.470	-4	b13 - H2O
1069.858925	21.7	$C_{136}H_{167}N_{42}O_{71}P_{12}S_{12}$	1069.856892	-1.900	-4	b13 - NH2
1075.110815	59.2	$C_{71}H_{88}N_{16}O_{38}P_6S_6$	1075.110594	-0.210	-2	b7 - B(G) - H2O, a7 - B(G)
1075.611640	100.2	$C_{136}H_{166}N_{46}O_{69}P_{12}S_{12}$	1075.610552	-1.010	-4	y13 - H2O, z13
1082.107996	22.4	$C_{172}H_{209}N_{56}O_{88}P_{15}S_{15}$	1082.110061	1.910	-5	M - 8H
1082.309583	14.7	$C_{172}H_{210}N_{56}O_{88}P_{15}S_{15}$	1082.311626	1.890	-5	M - 7H
1082.512783	41.3	$C_{172}H_{211}N_{56}O_{88}P_{15}S_{15}$	1082.513191	0.380	-5	M - 6H
1082.714713	356.6	$C_{172}H_{212}N_{56}O_{88}P_{15}S_{15}$	1082.714756	0.040	-5	M - 5H
1082.715179						
1084.115955	352.1	$C_{71}H_{90}N_{16}O_{39}P_6S_6$	1084.115876	-0.070	-2	b7 - B(G)
1085.118256	15.6	$C_{34}H_{44}O_{19}N_{10}P_3S_3$	1085.117017	-1.140	-1	d3 - C2H2
1087.111447	187.4	$C_{71}H_{86}N_{19}O_{37}P_6S_6$	1087.109922	-1.400	-2	a7 - 3H, z7 - B(dA) - NH2
1088.112539	354.4	$C_{70}H_{86}N_{20}O_{37}P_6S_6$	1088.111459	-0.990	-2	y7 - B(G) - CH4
1091.105565	339.3	$C_{71}H_{88}N_{16}O_{40}P_6S_6$	1091.105508	-0.050	-2	b7 - 4H
1092.107710	215.4	$C_{71}H_{84}N_{21}O_{36}P_6S_6$	1092.107714	0.000	-2	z7 - B(mC) - NH2
1095.101352	213.3	$C_{35}H_{42}O_{19}N_{10}P_3S_3$	1095.101367	0.010	-1	d3 - CH4
1096.101538	85.3	$C_{71}H_{86}N_{18}O_{39}P_6S_6$	1096.103026	1.360	-2	b7 - 4H
1097.781767	23.2	$C_{106}H_{128}N_{33}O_{54}P_9S_9$	1097.781043	-0.660	-3	a10 - 4H
1098.116546	64.2	$C_{106}H_{129}N_{33}O_{54}P_9S_9$	1098.116802	0.230	-3	b10 - H2O, a10
1099.094584	41.2	$C_{136}H_{165}N_{46}O_{71}P_{13}S_{13}$	1099.092511	-1.890	-4	x13 - 7H
1102.151792	22.5					
1111.133940	75.4	$C_{36}H_{46}O_{19}N_{10}P_3S_3$	1111.132668	-1.140	-1	d3
1114.459680	27.5	$C_{106}H_{128}N_{40}O_{51}P_9S_9$	1114.459785	0.090	-3	y10 - H2O, z10
1136.139998	292.1	$C_{36}H_{45}O_{18}N_{13}P_3S_3$	1136.139149	-0.750	-1	w3
1137.142785	101.7					
1150.131743	35.5	$C_{39}H_{47}N_9O_{20}P_3S_3$	1150.131784	0.040	-1	a4 - B(dC) - OC2H2
1150.635111	62.9	$C_{76}H_{93}N_{21}O_{39}P_6S_6$	1150.635024	-0.080	-2	b7 - H2O, a7
1152.444294	22.1	$C_{106}H_{131}N_{40}O_{54}P_{10}S_{10}$	1152.444469	0.150	-3	w10
1162.648561	22.4	$C_{76}H_{93}N_{25}O_{37}P_6S_6$	1162.646532	-1.740	-2	z7, y7 - H2O
1171.651965	8.4	$C_{76}H_{95}N_{25}O_{38}P_6S_6$	1171.651814	-0.130	-2	y7
1191.160843	13.9	$C_{41}H_{50}N_{10}O_{20}P_3S_3$	1191.158882	-1.650	-1	b4 - B(T) - H2O, a4 - B(T), y4 - B(dG) - NH3
1212.789212	9.1	$C_{116}H_{140}N_{38}O_{59}P_{10}S_{10}$	1212.790753	1.270	-3	a11 - 4H
1213.792166	11.4	$C_{116}H_{141}N_{37}O_{60}P_{10}S_{10}$	1213.790824	-1.110	-3	b11 - NH2
1219.624336	36.4	$C_{76}H_{96}N_{25}O_{40}P_7S_7$	1219.623558	-0.640	-2	w7
1220.124821	40.4	$C_{116}H_{138}N_{42}O_{57}P_{10}S_{10}$	1220.126358	1.260	-3	z11 - 6H
1247.624622	16.6	$C_{81}H_{100}N_{21}O_{43}P_7S_7$	1247.625158	0.430	-2	a8 - B(G), b8 - B(G) - H2O
1248.127243	19.8	$C_{79}H_{99}N_{24}O_{42}P_7S_7$	1248.128399	0.930	-2	b8 - B(T) - OC2H2

1259.118979	31.6	$C_{116}H_{144}N_{42}O_{60}P_{11}S_{11}$	1259.119050	0.060	-3	w11
1266.781359	13.4	$C_{119}H_{149}N_{35}O_{65}P_{11}S_{11}$	1266.782927	1.240	-3	b12 - B(A) - OC2H2
1272.102168	11.6	$C_{40}H_{50}N_{11}O_{21}P_{4}S_{4}$	1272.102155	-0.010	-1	x4 - B(dG) - CH3
1272.770892	10.5	$C_{120}H_{143}N_{37}O_{64}P_{11}S_{11}$	1272.771022	0.100	-3	a12 - B(dC) - CH4
1316.194430	87.0	$C_{46}H_{55}N_{12}O_{22}P_{3}S_{3}$	1316.193985	-0.340	-1	a4 - 2H
1317.200900	151.6	$C_{46}H_{56}N_{12}O_{22}P_{3}S_{3}$	1317.201261	0.270	-1	b4 - H2O, a4
1318.204373	55.0					
1322.646805	17.8	$C_{86}H_{104}N_{26}O_{44}P_{7}S_{7}$	1322.646224	-0.440	-2	a8 - 3H
1323.149057	36.9	$C_{86}H_{105}N_{26}O_{44}P_{7}S_{7}$	1323.149862	0.610	-2	b8 - H2O, a8
1335.212899	21.4	$C_{46}H_{58}N_{12}O_{23}P_{3}S_{3}$	1335.212374	-0.390	-1	b4
1341.223974	31.9	$C_{46}H_{56}N_{16}O_{20}P_{3}S_{3}$	1341.223727	-0.180	-1	y4 - H2O, z4
1353.393705	68.1	$C_{172}H_{212}N_{56}O_{88}P_{15}S_{15}$	1353.393445	-0.190	-4	M - 5H
1353.645306	233.7	$C_{172}H_{213}N_{56}O_{88}P_{15}S_{15}$	1353.645264	-0.030	-4	M - 4H
1353.646583			1353.645264	-0.974		
1365.791868	23.8	$C_{126}H_{157}N_{44}O_{66}P_{12}S_{12}$	1365.793265	1.020	-3	w12
1366.635676	38.0	$C_{86}H_{105}N_{30}O_{42}P_{8}S_{8}$	1366.634286	-1.020	-2	x8 - H2O
1367.127699	46.2	$C_{86}H_{104}N_{29}O_{43}P_{8}S_{8}$	1367.126294	-1.030	-2	x8 - NH3
1370.127455	84.8	$C_{84}H_{104}N_{30}O_{44}P_{8}S_{8}$	1370.125288	-1.580	-2	w8 - C2H4
1371.131351	37.5	$C_{84}H_{106}N_{30}O_{44}P_{8}S_{8}$	1371.132839	1.090	-2	w8 - C2H2
1371.627848	20.4	$C_{86}H_{107}N_{26}O_{46}P_{8}S_{8}$	1371.625793	-1.500	-2	c8
1375.124564	48.1	$C_{131}H_{161}N_{38}O_{69}P_{12}S_{12}$	1375.125798	0.900	-3	a13 - B(A) - H2O, a13 - 5H
1378.614239	25.0	$C_{86}H_{105}N_{26}O_{47}P_{8}S_{8}$	1378.615151	0.660	-2	d8 - 5H
1384.141093	87.3	$C_{86}H_{108}N_{30}O_{44}P_{8}S_{8}$	1384.140939	-0.110	-2	w8
1395.129924	25.6	$C_{131}H_{163}N_{41}O_{70}P_{12}S_{12}$	1395.132394	1.770	-3	y13 - 4H
1431.158185	43.8	$C_{46}H_{59}N_{12}O_{25}P_{4}S_{4}$	1431.155861	-1.620	-1	d4
1455.179913	106.2	$C_{46}H_{59}N_{16}O_{23}P_{4}S_{4}$	1455.178328	-1.090	-1	w4
1556.655824	19.6	$C_{96}H_{120}N_{35}O_{49}P_{9}S_{9}$	1556.655777	-0.030	-2	w9
1557.157232	49.2	$C_{148}H_{178}O_{74}N_{51}P_{13}S_{13}$	1557.157113	-0.080	-3	z14 - 6H
1568.143761	19.2	$C_{146}H_{182}O_{76}N_{48}P_{14}S_{14}$	1568.143026	-0.470	-3	c14 - OC2H2
1636.218643	40.5	$C_{56}H_{68}N_{14}O_{28}P_{4}S_{4}$	1636.217178	-0.900	-1	a5 - 2H
1637.224842	78.6	$C_{56}H_{69}N_{14}O_{28}P_{4}S_{4}$	1637.225003	0.100	-1	b5 - H2O, a5
1650.145239	27.2	$C_{51}H_{65}N_{15}O_{28}P_{5}S_{5}$	1650.142610	-1.590	-1	w5 - B(mC)
1805.192593	135.2	$C_{172}H_{214}N_{56}O_{88}P_{15}S_{15}$	1805.196111	1.950	-3	M - 3H
1805.192594	135.2	$C_{59}H_{74}N_{14}O_{32}P_{5}S_{5}$	1805.189620	-1.650	-1	a6 - B(T) - C2H2
1805.196082						
1812.187044	60.8	$C_{116}H_{140}N_{37}O_{59}P_{10}S_{10}$	1812.184592	-1.350	-2	a11 - NH2
1820.180831	70.8	$C_{61}H_{75}N_{11}O_{34}P_{5}S_{5}$	1820.178052	-1.530	-1	a6 - 3H
1821.189437	44.7	$C_{116}H_{142}N_{37}O_{60}P_{10}S_{10}$	1821.189600	0.090	-2	b11 - NH2
			Abs mean error	0.679		
			Abs mean std dev	0.532		

Table S10 showing peak list, signal-to-noise ratio (S/N), elemental composition, and possible assignments with mass errors (ppm) of the $[M-8H]^{8-}$ danvatirsen precursor ion by 2D-EDD-MS.

Measured *mz*	S/N	Elemental Composition	Theoretical *mz*	Error (ppm)	Charge	Assignment
409.552864	51.3	$C_{29}H_{35}O_{15}N_5P_2S_2$	409.552864	0.000	-2	a3 - B(dA) - OC2H2
598.806110	12.7	$C_{74}H_{92}N_{25}O_{39}P_7S_7$	598.805499	-1.020	-4	w7 - OC2H2
658.066901	30.7	$C_{106}H_{125}N_{33}O_{54}P_9S_9$	658.064150	-4.180	-5	a10 - 7H
659.439887	74.3	$C_{167}H_{204}N_{51}O_{88}P_{15}S_{15}$	659.437182	-4.102	-8	M - 8H - A
659.940365	223.3	$C_{32}H_{15}N_3P_5S_2$	659.940098	0.400		
662.272560	7.5	$C_{17}H_{36}N_{21}O_4S_2$	662.270605	2.950		
674.091208	15.8	$C_{66}H_{81}N_{23}O_{32}P_5S_5$	674.091053	-0.230	-3	y6
675.200277	11.4	$C_{148}H_{178}O_{77}N_{47}P_{14}S_{14}$	675.198830	-2.140	-7	c14 - NH2
677.695695	107.2	$CH_2N_7O_{16}P_{10}$	677.693971	2.540		
677.857359	15.0	$C_{106}H_{129}N_{33}O_{56}P_{10}S_{10}$	677.857433	0.110	-5	c10
678.258016	10.5	$C_{127}H_{210}N_{27}O_{76}P_2$	678.258007	0.010		
681.260142	10.4	$C_{106}H_{130}N_{33}O_{57}P_{10}S_{10}$	681.258091	-3.010	-5	d10
691.065761	28.7	$C_{106}H_{129}N_{40}O_{54}P_{10}S_{10}$	691.063881	-2.720	-5	w10
691.568994	6.6	$C_{86}H_{106}N_{30}O_{44}P_8S_8$	691.566831	-3.130	-4	w8
692.068673	8.0	$C_{64}H_{80}N_{23}O_{33}P_6S_6$	692.068694	0.030	-3	w6 - OC2H2
709.564247	18.3	$C_{91}H_{110}N_{26}O_{48}P_8S_8$	709.566360	2.980	-4	b9 - B(A) - H2O, a9 - B(A)
710.067257	48.5	$C_{111}H_{132}N_{40}O_{56}P_{10}S_{10}$	710.066542	-1.010	-5	y11 - 8H
716.737698	14.3	$C_{136}H_{164}N_{46}O_{69}P_{12}S_{12}$	716.737851	0.210	-6	z13, y13 - H2O
727.474935	33.2	$C_{116}H_{139}N_{38}O_{59}P_{10}S_{10}$	727.473216	-2.360	-5	a11, b11 - H2O
743.329966	78.8	$C_{96}H_{115}N_{31}O_{48}P_8S_8$	743.329984	0.020	-4	b9 - H2O, a9
747.569034	29.9	$C_{51}H_{59}N_{10}O_{27}P_4S_4$	747.569771	0.990	-2	a5 - B(dC) - NH2
751.641619	38.4	$C_{166}H_{202}N_{51}O_{88}P_{15}S_{15}$	751.640257	-1.810	-7	M - 7H - CH3 - A, M - 7H - CH3 - dA
753.835147	13.4	$C_{96}H_{117}N_{35}O_{47}P_8S_8$	753.838241	4.100	-4	y9
755.214411	40.6	$C_{167}H_{203}N_{53}O_{87}P_{15}S_{15}$	755.214409	0.000	-7	M - 7H - dC, M - 7H - mC
766.753215	75.7	$C_{76}H_{92}N_{21}O_{39}P_6S_6$	766.754257	1.360	-3	a7, b7 - H2O
772.935341	66.9	$C_{172}H_{209}N_{56}O_{88}P_{15}S_{15}$	772.935914	0.740	-7	M - 8H
777.826157	24.3	$C_{96}H_{118}N_{35}O_{49}P_9S_9$	777.824250	-2.450	-4	w9
791.275195	21.9	$C_{126}H_{151}N_{40}O_{65}P_{11}S_{11}$	791.276180	1.240	-5	a12 - 6H
796.882497	20.7	$C_{125}H_{151}N_{44}O_{64}P_{11}S_{11}$	796.879766	-3.430	-5	y12 - CH3
809.410002	30.8	$C_{153}H_{186}O_{80}N_{48}P_{14}S_{14}$	809.406947	-3.770	-6	a15 - B(dA) - OC2H2, a15 - B(dG) - C2H2, z15 - B(dA) - OC2H2, z15 - B(G) - C2H2
812.747347	33.6	$C_{76}H_{95}N_{25}O_{40}P_7S_7$	812.746613	-0.900	-3	w7
819.072246	196.5	$C_{126}H_{155}N_{44}O_{66}P_{12}S_{12}$	819.073048	0.980	-5	w12
823.334659	38.0	$C_{106}H_{128}N_{33}O_{54}P_9S_9$	823.335782	1.360	-4	a10, b10 - H2O
824.086475	14.5	$C_{105}H_{127}N_{33}O_{55}P_9S_9$	824.082692	-4.590	-4	b10 - CH3
830.118236	26.1	$C_{56}H_{68}N_{18}O_{26}P_4S_4$	830.119822	1.910	-2	y5 - H2O, z5
840.095692	29.8	$C_{106}H_{129}N_{40}O_{52}P_9S_9$	840.095661	-0.040	-4	y10
860.285558	197.4	$C_{136}H_{165}N_{46}O_{69}P_{12}S_{12}$	860.286877	1.530	-5	z13, y13 - H2O
881.764445	42.9	$C_{86}H_{104}N_{26}O_{44}P_7S_7$	881.764332	-0.130	-3	a8, b8 - H2O
884.440009	64.9	$C_{86}H_{104}N_{30}O_{41}P_7S_7$	884.440000	-0.010	-3	y8 - H2O, z8
902.094008	255.3	$C_{172}H_{211}N_{56}O_{88}P_{15}S_{15}$	902.094417	0.450	-6	M - 6H
915.845901	17.2	$C_{116}H_{139}N_{41}O_{58}P_{10}S_{10}$	915.844959	-1.030	-4	y11 - NH3
944.086479	14.5	$C_{116}H_{143}N_{42}O_{60}P_{11}S_{11}$	944.087331	0.900	-4	w11
991.449236	18.3	$C_{94}H_{116}N_{35}O_{46}P_8S_8$	991.449893	0.660	-3	y9 - OC2H2
999.449869	12.3	$C_{96}H_{116}N_{35}O_{46}P_8S_8$	999.449893	0.020	-3	y9 - H2O, z9
			Abs mean	1.510		

error	
Abs mean std dev	1.370

Table S11 showing peak list, signal-to-noise ratio (S/N), elemental composition, and possible assignments with mass errors (ppm) of the $[M-7H]^{7-}$ danvatirsen precursor ion by 2D-EDD-MS.

Measured *mz*	S/N	Elemental Composition	Theoretical *mz*	Error (ppm)	Charge	Assignment
609.300805	32.7	$C_{36}H_{52}P_3S$	609.300807	0.003		
657.593534	19.1	$C_{46}H_{54}N_{12}O_{22}P_3S_3$	657.593354	-0.270	-2	a4 - 3H
706.072033	8.9	$C_{66}H_{82}N_{23}O_{34}P_6S_6$	706.072032	0.000	-3	w6
727.082478	10.6	$C_{46}H_{58}N_{16}O_{23}P_4S_4$	727.085251	3.810	-2	w4
752.069594	6.7	$C_{141}H_{173}O_{74}N_{46}P_{13}S_{13}$	752.069657	0.080	-6	y14 - B(dA) - OC2H2, b14 - B(T) - C2H2
753.783044	88.9	$C_{167}H_{205}N_{51}O_{88}P_{15}S_{15}$	753.786390	4.440	-7	M - 7H - dA, M - 7H - A
767.087703	7.4	$C_{51}H_{60}N_{15}O_{25}P_4S_4$	767.086728	-1.270	-2	z5 - 3H
769.226156	5.7	$C_{143}H_{173}O_{75}N_{46}P_{14}S_{14}$	769.226448	0.380	-6	c14 - 8H, x14 - 8H, x14 - B(dA) - H2O
771.224893	13.6	$C_{143}H_{171}O_{75}N_{47}P_{14}S_{14}$	771.224443	-0.580	-6	x14 - B(mC) - NH2
773.076333	1329.8	$C_{146}H_{179}O_{76}N_{48}P_{13}S_{13}$	773.079889	4.600	-6	M - 7H
775.094056	28.4	$C_{51}H_{62}N_{16}O_{25}P_4S_4$	775.095816	2.270	-2	y5 - 4H
775.426043	14.8	$C_{76}H_{92}N_{24}O_{38}P_6S_6$	775.425876	-0.220	-3	y7 - NH2
791.874770	9.5	$C_{125}H_{150}N_{40}O_{66}P_{11}S_{11}$	791.873708	-1.340	-5	b12 - CH4
823.081745	8.3	$C_{106}H_{127}N_{33}O_{54}P_9S_9$	823.083826	2.530	-4	a10 - 5H
857.909713	22.3	$C_{160}H_{196}O_{84}N_{53}P_{15}S_{15}$	857.910041	0.380	-6	w15, d15
864.081054	23.0	$C_{106}H_{130}N_{40}O_{54}P_{10}S_{10}$	864.081532	0.550	-4	w10
881.426771	74.5	$C_{86}H_{103}N_{26}O_{44}P_7S_7$	881.428208	1.630	-3	a8 - 4H
881.583616	19.0	$C_{111}H_{134}N_{37}O_{57}P_{10}S_{10}$	881.583375	-0.270	-4	z11 - 5H
897.282449	11.1	$C_{141}H_{173}O_{75}N_{43}P_{13}S_{13}$	897.280727	-1.920	-5	a14 - B(dA) - C2H2
901.927253	84.8	$C_{172}H_{210}N_{56}O_{88}P_{15}S_{15}$	901.926537	-0.790	-6	M - 7H
905.590383	11.7	$C_{116}H_{138}N_{37}O_{59}P_{10}S_{10}$	905.588657	-1.910	-4	a11 - NH2
909.596910	36.7	$C_{114}H_{140}N_{42}O_{57}P_{10}S_{10}$	909.598818	2.100	-4	y11 - OC2H2
910.342717	38.6	$C_{115}H_{139}N_{38}O_{60}P_{10}S_{10}$	910.340111	-2.860	-4	b11 - CH3
922.426746	89.1	$C_{86}H_{107}N_{30}O_{44}P_8S_8$	922.424867	-2.040	-3	w8
944.086516	12.2	$C_{116}H_{143}N_{42}O_{60}P_{11}S_{11}$	944.087331	0.860	-4	w11
986.324995	13.7	$C_{121}H_{149}N_{38}O_{66}P_{12}S_{12}$	986.324962	-0.030	-4	w12 - B(A) - NH2, d12 - 5H
989.598976	28.5	$C_{126}H_{153}N_{40}O_{65}P_{11}S_{11}$	989.599000	0.020	-4	b12 - H2O, a12
991.442573	20.5	$C_{96}H_{116}N_{31}O_{48}P_8S_8$	991.442587	0.010	-3	b9 - H2O, a9
995.600420	21.5	$C_{126}H_{153}N_{44}O_{63}P_{11}S_{11}$	995.604617	4.220	-4	z12, y12 - H2O
996.106329	21.3	$C_{94}H_{114}N_{35}O_{47}P_8S_8$	996.109647	3.330	-3	y9 - C2H4
1024.091555	75.4	$C_{126}H_{156}N_{44}O_{66}P_{12}S_{12}$	1024.093130	1.540	-4	w12
1037.434604	29.0	$C_{96}H_{119}N_{35}O_{49}P_9S_9$	1037.434577	-0.030	-3	w9
1052.504799	19.8	$C_{167}H_{207}N_{51}O_{87}P_{15}S_{15}$	1052.504874	0.070	-5	M - 5H - G, M - 5H - dG
1059.613228	9.9	$C_{66}H_{83}N_{23}O_{34}P_6S_6$	1059.611961	-1.200	-2	w6
1066.083472	13.2	$C_{129}H_{161}N_{44}O_{70}P_{13}S_{13}$	1066.084284	0.760	-4	w13 - B(T) - C2H2
1069.611823	11.2	$C_{134}H_{166}N_{46}O_{69}P_{12}S_{12}$	1069.610552	-1.190	-4	y13 - OC2H2
1075.608471	38.5	$C_{136}H_{166}N_{46}O_{69}P_{12}S_{12}$	1075.610415	1.810	-4	y13 - H2O, z13
1082.309867	35.7	$C_{172}H_{210}N_{56}O_{88}P_{15}S_{15}$	1082.311626	1.630	-5	M - 7H
1115.454933	24.5	$C_{105}H_{127}N_{40}O_{52}P_9S_9$	1115.455481	0.490	-3	y10 - CH3
1219.622982	17.4	$C_{76}H_{96}N_{25}O_{40}P_7S_7$	1219.623284	0.250	-2	w7
1259.113350	11.0	$C_{157}H_{195}N_{50}O_{82}P_{15}S_{15}$	1259.113205	-0.120	-4	M - 4H - 3T
1556.655498	10.3	$C_{96}H_{120}N_{35}O_{49}P_9S_9$	1556.655503	0.000	-2	w9
			Abs mean error	1.324		
			Abs mean std dev	1.207		

Table S12 showing peak list, signal-to-noise ratio (S/N), elemental composition, and possible assignments with mass errors (ppm) of the $[M-6H]^{6-}$ danvatirsen precursor ion by 2D-EDD-MS.

Measured *mz*	S/N	Elemental Composition	Theoretical *mz*	Error (ppm)	Charge	Assignment
773.228418	13.0	$C_{143}H_{171}O_{74}N_{49}P_{14}S_{14}$	773.226315	-2.720	-6	x14 - 9H
782.649897	12.4	$C_{120}H_{143}N_{37}O_{66}P_{12}S_{12}$	782.649965	0.090	-5	c12 - B(dC) - CH4
817.605421	12.1	$C_{56}H_{67}N_{14}O_{28}P_{4}S_{4}$	817.604950	-0.580	-2	a5 - 3H
876.913662	13.5	$C_{166}H_{202}N_{51}O_{88}P_{15}S_{15}$	876.913542	-0.140	-6	M - 6H - CH4 - A, M - 6H - CH4 - dA
877.080841	16.5	$C_{111}H_{132}N_{37}O_{56}P_{10}S_{10}$	877.080734	-0.120	-4	z11 - 7H
877.579937	12.4	$C_{111}H_{132}N_{36}O_{57}P_{10}S_{10}$	877.578694	-1.420	-4	z11 - B(A) - NH3, a11 - 6H
880.078501	23.2	$C_{111}H_{130}N_{38}O_{56}P_{10}S_{10}$	880.077590	-1.030	-4	z11 - B(mC) - NH3
880.410501	12.3	$C_{81}H_{101}N_{28}O_{42}P_{8}S_{8}$	880.410558	0.060	-3	w8 - B(T)
887.095694	26.9	$C_{86}H_{104}N_{26}O_{45}P_{7}S_{7}$	887.095788	0.110	-3	b8 - 5H
899.424283	17.5	$C_{172}H_{209}N_{55}O_{88}P_{15}S_{15}$	899.424630	0.390	-6	M - 6H - NH2
899.591417	33.9	$C_{171}H_{208}N_{56}O_{88}P_{15}S_{15}$	899.590596	-0.910	-6	M - 6H - CH3
901.087073	13.9	$C_{59}H_{71}N_{14}O_{32}P_{5}S_{5}$	901.083346	-4.140	-2	a6 - B(T) - C2H4
901.276073	7.5	$C_{142}H_{169}O_{74}N_{45}P_{13}S_{13}$	901.276603	0.590	-5	a14 - B(mC) - CH4
901.476671	14.2	$C_{142}H_{170}O_{74}N_{45}P_{13}S_{13}$	901.478168	1.660	-5	a14 - B(mC) - CH3
902.568150	145.7	$C_{111}H_{135}N_{35}O_{60}P_{11}S_{11}$	902.566438	-1.900	-4	d11 - B(T) - NH2, c11 - B(dC)
902.587107	3375.9	$C_{114}H_{136}N_{38}O_{59}P_{10}S_{10}$	902.585513	-1.770	-4	a11 - C2H4
902.753808	2819.6	$C_{84}H_{104}N_{30}O_{42}P_{8}S_{8}$	902.753766	-0.050	-3	x8 - OC2H2
903.087350	1280.0	$C_{114}H_{138}N_{38}O_{59}P_{10}S_{10}$	903.089289	2.150	-4	a11 - C2H2
903.587386	188.2	$C_{59}H_{74}N_{13}O_{33}P_{5}S_{5}$	903.590730	3.700	-2	b6 - B(dC) - OC2H2
904.087315	15.3	$C_{143}H_{173}O_{73}N_{46}P_{13}S_{13}$	904.084605	-3.000	-5	a14 - 6H, z14 - 6H
906.084658	166.8	$C_{143}H_{171}O_{72}N_{48}P_{13}S_{13}$	906.083722	-1.030	-5	z14 - B(T) - NH2
906.583893	73.7	$C_{61}H_{72}N_{13}O_{32}P_{5}S_{5}$	906.585448	1.720	-2	a6 - B(dC) - H2O, a6 - B(T) - NH3
909.082939	38.1	$C_{61}H_{73}N_{11}O_{34}P_{5}S_{5}$	909.081475	-1.610	-2	a6 - 5H
909.587429	13.2	$C_{61}H_{74}N_{11}O_{34}P_{5}S_{5}$	909.585387	-2.240	-2	a6 - 4H
912.082526	6.8	$C_{60}H_{75}N_{11}O_{35}P_{5}S_{5}$	912.086483	4.340	-2	b6 - B(dA) - CH3
922.424191	17.9	$C_{86}H_{107}N_{30}O_{44}P_{8}S_{8}$	922.424684	0.530	-3	w8
977.614026	20.8	$C_{66}H_{80}N_{16}O_{34}P_{5}S_{5}$	977.616274	2.300	-2	a6 - 3H
978.616772	38.2	$C_{66}H_{80}N_{15}O_{35}P_{5}S_{5}$	978.612468	-4.400	-2	b6 - NH3
989.845384	12.5	$C_{126}H_{152}N_{39}O_{66}P_{11}S_{11}$	989.845141	-0.250	-4	b12 - NH3
1000.111740	11.9	$C_{95}H_{114}N_{35}O_{47}P_{8}S_{8}$	1000.109830	-1.910	-3	y9 - CH4
1024.090111	14.7	$C_{96}H_{117}N_{30}O_{51}P_{9}S_{9}$	1024.087696	-2.360	-3	d9 - NH2
1024.589666	31.6	$C_{129}H_{158}N_{38}O_{69}P_{12}S_{12}$	1024.588754	-0.890	-4	a13 - B(G) - C2H4
1029.685528	24.8	$C_{161}H_{195}N_{50}O_{86}P_{15}S_{15}$	1029.686496	0.940	-5	M - 5H - CH3 - 2dC
1031.091806	16.4	$C_{131}H_{160}N_{38}O_{69}P_{12}S_{12}$	1031.092530	0.700	-4	a13 - 6H, a13 - B(A) - H2O
1038.094524	57.1	$C_{131}H_{160}N_{40}O_{69}P_{12}S_{12}$	1038.094204	-0.310	-4	a13 - B(mC), b13 - B(mC) - H2O, b13 - B(T) - NH3
1059.609903	25.6	$C_{66}H_{83}N_{23}O_{34}P_{6}S_{6}$	1059.611687	1.680	-2	w6
1076.604313	22.3	$C_{70}H_{87}N_{16}O_{39}P_{6}S_{6}$	1076.604138	-0.160	-2	b7 - B(G) - CH3
1083.075702	11.9	$C_{100}H_{121}N_{31}O_{54}P_{10}S_{10}$	1083.075830	0.120	-3	c10 - B(T) - CH4
1083.105795	218.9	$C_{71}H_{88}N_{16}O_{39}P_{6}S_{6}$	1083.107777	1.830	-2	b7 - 4H, a7 - B(dA), b7 -

1098.110131	27.4	$C_{71}H_{84}N_{23}O_{35}P_6S_6$	1098.113057	2.660	-2	B(dA) - H2O z7 - 5H
1098.444620	16.9	$C_{106}H_{128}N_{32}O_{55}P_9S_9$	1098.444807	0.170	-3	b10 - NH3
1098.776345	25.7	$C_{105}H_{127}N_{33}O_{55}P_9S_9$	1098.776557	0.190	-3	b10 - CH4
1151.130421	11.3	$C_{76}H_{92}N_{20}O_{40}P_6S_6$	1151.127306	-2.710	-2	b7 - NH3
1151.630570	12.5	$C_{76}H_{93}N_{20}O_{40}P_6S_6$	1151.630945	0.330	-2	b7 - NH2
1153.433485	36.1	$C_{109}H_{132}N_{33}O_{58}P_{10}S_{10}$	1153.433307	-0.150	-3	a11 - B(G) - C2H4
1214.115839	12.4	$C_{153}H_{186}O_{80}N_{48}P_{14}S_{14}$	1214.110146	-4.690	-4	a15 - B(dG) - C2H4, z15 - B(G) - C2H4
1259.106145	21.1	$C_{81}H_{95}N_{23}O_{43}P_7S_7$	1259.108669	2.000	-2	a8 - 5H
1260.104192	30.1	$C_{119}H_{145}N_{35}O_{64}P_{11}S_{11}$	1260.107522	2.640	-3	a12 - B(G) - C2H4
1329.108128	10.4	$C_{41}H_{51}N_{13}O_{22}P_4S_4$	1329.111043	2.190	-1	w4 - 2H
1354.116802	39.3	$C_{126}H_{154}N_{44}O_{64}P_{12}S_{12}$	1354.122164	3.960	-3	x12 - H2O
1554.823124	9.4	$C_{146}H_{179}O_{75}N_{51}P_{13}S_{13}$	1554.824509	0.890	-3	y14 - C2H4
			Abs mean error	1.649		
			Abs mean std dev	1.238		

Table S13 showing peak list, intensity, elemental composition, and possible assignments with mass errors (ppm) of danvatirsen using 2D-UVPD-MS of one scan denoised (sane rank 10) $[M-8H]^{8-}$ precursor ion (note, noise peaks were included to highlight the effect of denoising).

Measured *mz*	Intensity	Elemental Composition	Theoretical *mz*	Error (ppm)	Charge	Assignment
225.560984	1740907.0					
225.564220	2472008.7					
338.157749	13274609.6					
338.167652	1797371.3					
338.210996	4110571.8					
338.220422	29988344.8					
338.229929	4303612.9					
338.274284	3284753.3					
338.283048	33029211.8					
338.292211	4172903.8					
338.344567	7808075.3					
338.400417	2226435.6					
338.408269	14765760.8					
338.416373	2010386.8					
338.462275	1705712.6					
338.470583	22471378.7					
338.478993	1819578.4					
338.533041	18285322.3					
338.543014	2596628.7					
338.595163	7180541.1					
338.657361	2040699.1					
338.658672	1770008.4					
338.720658	1827530.5					
339.866196	2043140.2					
541.254272	2122260.4					
601.395200	3262256.6					
609.308001	3269107.6	$C_{76}H_{98}N_{25}O_{40}P_7S_7$	609.308004	0.005	-4	w7
657.563909	2050827.9					
657.593028	3516477.7					
659.438177	3118306.4					
659.565777	4440149.1					
659.689513	11226883.5					
659.815492	10058763.5					
659.938761	4761343.7					
673.823356	1858546.2					
673.948559	1788057.1					
674.072977	2127635.3					
674.091895	4450368.8	$C_{66}H_{84}N_{23}O_{32}P_5S_5$	674.090870	-1.521	-3	y6
674.323228	17057623.6					
674.448454	37431606.0					
674.535295	2237489.1					
674.547643	1847308.9					
674.573701	56003409.2					
674.659884	2463284.7					
674.698669	68020902.2					
674.722873	2284603.4					
674.735919	1879203.5					
674.784842	2426621.4					
674.799736	2130285.2					

674.823907	61461539.8					
674.909703	2818066.0					
674.924147	2034247.5					
674.948773	49187437.3					
675.073537	30691037.1					
675.198378	22544211.6					
675.323175	13207262.5					
675.447751	9029169.8					
675.573842	4935024.4					
675.692539	2450861.8					
676.065599	2180528.4					
676.078851	2806215.3					
676.100736	2088056.5					
676.118543	2148572.7					
676.135623	2144589.3					
676.145012	2129377.5					
676.153393	1780000.6					
676.163327	2344002.6					
676.172063	2537847.0					
676.181199	2301547.2					
676.205386	4392098.2					
676.217123	3406627.8					
676.223742	3589781.0					
676.235204	5127274.1					
676.252274	6424758.7					
676.260528	5780503.6					
676.271499	9646054.7					
676.282903	20794313.8					
676.295290	18312119.5					
676.320085	580758939.5	$C_{172}H_{217}N_{56}O_{88}P_{15}S_{15}$	676.318994	1.613	-8	M - 8H
676.344300	22219809.5					
676.361034	16823494.2					
676.378127	14278468.8					
676.384332	15371881.0					
676.408048	62122979.4					
676.419374	45851471.6					
676.445526	1339304528.0					
676.468426	50599789.7					
676.479270	30373811.2					
676.486515	37018959.8					
676.496340	23991641.2					
676.505292	28265522.1					
676.514315	27569320.6					
676.532633	111341706.7					
676.543660	66244585.8					
676.570704	2111879384.0					
676.591682	77138646.0					
676.602485	38581643.8					
676.611245	56017752.0					
676.619805	35327541.9					
676.629566	38745838.0					
676.638135	37636107.8					
676.657391	134932471.9					
676.678822	78977449.1					
676.695805	2350566485.0					
676.712388	98535232.1					

676.735977	57888830.8					
676.743430	38234956.9					
676.753984	39163516.0					
676.762387	36657561.8					
676.782190	135459408.5					
676.798742	59106797.0					
676.820847	1997254297.0					
676.860725	46553219.5					
676.878336	28267141.3					
676.886779	27660163.1					
676.907227	102686862.1					
676.922568	47285898.1					
676.945876	1411811359.0					
676.969582	43747164.1					
676.985820	31085876.2					
677.003045	17155473.3					
677.010916	18459769.4					
677.021893	17805846.9					
677.032659	71536844.3					
677.046896	33613996.8					
677.070880	963963387.5					
677.094343	31652416.6					
677.111923	20645959.6					
677.137091	12670373.2					
677.157634	46554603.2					
677.171432	20250897.5					
677.195922	576079625.1					
677.219610	20068370.3					
677.236802	16159955.5					
677.256205	6415461.7					
677.264974	5188949.7					
677.282556	26628410.7					
677.296078	10506439.6					
677.320901	325071932.9					
677.362171	6998622.8					
677.381444	3350581.1					
677.389528	3739625.4					
677.407635	15733209.3					
677.423273	8847064.1					
677.446092	198508193.9					
677.468717	6911910.0					
677.487278	5293095.1					
677.506029	2699582.5					
677.523533	4005081.7					
677.537127	2163110.9					
677.545980	3643438.6					
677.571123	105726269.4					
677.611501	2599049.5					
677.623226	2910797.4					
677.635895	1997353.2					
677.696235	50105838.7					
677.722206	2848457.4					
677.736037	3087685.5					
677.820732	22422530.6					
677.852572	2792964.6	$C_{106}H_{132}N_{32}O_{57}P_{10}S_{10}$	677.852671	0.146	-5	d10 - NH3
677.946112	12247984.5					
678.071480	5548593.8					

678.196834	3536380.4					
678.445351	2181286.8					
678.569593	2856942.2					
678.693505	2741105.0					
678.819365	2729577.9					
679.068017	10559204.8					
679.193229	22486782.5					
679.279147	2241412.7					
679.318342	32462966.6					
679.404787	2142412.0					
679.443163	30698988.8					
679.568268	23207713.5					
679.693517	13508959.9					
679.817806	10603946.2					
679.942452	6982888.2					
680.068072	1945161.3					
681.064186	1764848.4					
681.314001	2140946.8					
681.440422	1812207.1					
681.563700	2356112.3					
681.689731	1751704.1					
682.395270	1796795.4					
690.736136	2533836.5					
690.835788	2695622.2					
690.936476	2756918.8					
691.036522	1769547.3					
727.677437	2355547.0					
743.583622	3098158.1					
747.737399	2852872.5					
767.091580	2938220.4					
767.921573	2204085.1					
767.931630	3340896.7					
768.252927	2608376.5					
768.263279	5783073.2					
770.042671	2527418.5					
773.226061	2336579.4					
773.367236	3279493.3					
773.610044	1995741.1					
773.653205	3660735.6					
778.786238	2204170.1					
778.794185	2261073.8					
779.507799	2753107.3					
791.478522	4869501.5	$C_{126}H_{157}N_{40}O_{65}P_{11}S_{11}$	791.477745	-0.982	-5	a12 - 2H, b12 - H2O
791.683635	5265322.7					
809.244518	2646074.0					
809.412924	2200580.8					
809.583106	2998124.0					
819.077302	2653843.8					
819.275993	9287857.0	$C_{130}H_{165}N_{38}O_{68}P_{12}S_{12}$	819.275150	-1.029	-5	a13 - B(G) - OCH2
819.476652	10400434.6	$C_{126}H_{162}N_{44}O_{66}P_{12}S_{12}$	819.476178	-0.578	-5	w12 + 2H
819.678017	5160140.4					
860.490217	9847022.2					
860.689693	14997227.1	$C_{136}H_{172}N_{46}O_{69}P_{12}S_{12}$	860.690007	0.365	-5	z13
860.890018	10153079.3	$C_{136}H_{173}N_{46}O_{69}P_{12}S_{12}$	860.891572	1.805	-5	z13 + 1H
861.088815	1995140.7					

863.542342	19006860.3					
863.741353	1864584.5					
863.909425	2606768.0					
864.169711	8249064.0					
864.297983	3699198.2					
876.752174	3013012.7					
876.915628	3664065.8					
881.253695	1825487.9					
884.441921	2120013.3	$C_{86}H_{107}N_{30}O_{41}P_7S_7$	884.440000	-2.172	-3	y8 - H2O, z8 - 2H
884.779648	2827743.2					
901.760280	22683477.4					
901.860281	3628281.9					
901.927567	47903549.5					
901.958943	2013722.9					
902.125126	2549807.9					
902.257288	38775853.6					
902.272893	9774832.1					
902.364671	2078123.6					
902.418674	4541622.7					
902.430634	4835952.5					
902.512413	2404363.3					
903.088727	12502482.5	$C_{143}H_{183}N_{43}O_{75}P_{13}S_{13}$	903.088442	-0.316	-5	b14 - B(G) + 1H, a14 - B(A) + 2H
903.103364	5340552.8					
987.350404	4244072.5					
987.492629	2520404.9					
1029.430911	1801827.3	$C_{96}H_{122}N_{31}O_{51}P_9S_9$	1029.427088	-3.714	-3	d9
1353.889270	8821596.1					
1354.140405	12422843.9					
1354.394871	21162897.4					
1354.630836	11556415.2					
1354.886262	9707054.5					
1355.129563	8243090.6					
1382.888983	4191613.4					
			Abs mean error	1.254		
			Abs mean std dev	1.193		

Table S14 showing peak list, intensity, elemental composition, and possible assignments with mass errors (ppm) of danvatirsen using 2D-UVPD-MS of one scan denoised (sane rank 10) [M-7H]$^{7-}$ precursor ion (note, noise peaks were included to highlight the effect of denoising).

Measured *mz*	Intensity	Elemental Composition	Theoretical *mz*	Error (ppm)	Charge	Assignment
255.866784	3610139.0					
383.685103	6199175.2					
383.741385	3294117.3					
383.785554	1752580.9					
383.841110	4109102.5					
383.852558	32505181.8					
383.864384	3734794.7					
383.896892	5396112.4					
383.907975	37886865.6					
383.916111	2055700.2					
383.920011	4260967.5					
383.952070	4188873.8					
383.975584	3866152.2					
384.008704	2717526.8					
384.019105	22050820.7	$C_{61}H_{75}N_{14}O_{34}P_6S_6$	384.019125	0.052	-5	c6 - B(T) - 4H
384.031185	2687780.2					
384.074861	11964750.5					
384.130517	4955578.4					
386.682179	3568937.3					
386.753409	4976781.3					
386.826135	2222056.2	$C_{60}H_{77}N_{18}O_{32}P_6S_6$	386.826749	1.587	-5	x6 - B(dA) - OCH2
601.619497	1734570.2					
676.537841	7016826.7					
676.687643	2052718.8					
676.696758	4411751.5					
676.703219	3615941.3					
676.910633	1837422.4					
728.078860	1877272.6	$C_{116}H_{147}N_{38}O_{59}P_{10}S_{10}$	728.077801	-1.455	-5	a11 + 1H
753.789362	1916603.0					
753.932308	2846057.6					
754.076928	2843069.7					
765.486704	5470247.4					
765.598616	18593178.6					
765.620524	1928865.2					
765.656558	1864457.9					
765.672307	1919043.1	$C_{120}H_{150}N_{41}O_{61}P_{11}S_{11}$	765.671473	-1.089	-5	z12 - B(dC) - OCH2
765.709871	35555376.1					
765.767521	1907022.3					
765.782199	2098160.3					
765.799707	2178243.7					
765.821254	51036461.4					
765.853199	2909196.2					
765.867040	2371613.8					
765.877658	3872165.7					
765.886565	3071598.8					
765.898209	3082407.7					
765.932594	60995960.3					
765.961240	3547901.4					
765.973399	2369344.2					

765.987063	3572177.4					
766.006912	2597221.9					
766.020135	2963440.7					
766.043676	52661330.8					
766.101621	3560714.2	$C_{24}H_{33}N_{8}O_{13}P_{2}S_{2}$	766.101098	-0.683	-1	w2 + 1H
766.108141	3853459.5					
766.121009	2542394.1					
766.154759	45232279.5					
766.184661	3348837.5					
766.217333	3783983.4					
766.240870	2212025.4					
766.299480	2677083.8					
766.331287	3304060.1					
766.349189	2768007.0					
766.376922	24945135.7					
766.407599	2070950.1					
766.441326	2951904.9					
766.487377	17654784.0					
766.515635	2466585.8					
766.525831	2368096.8					
766.537297	2061383.2					
766.550353	2449211.0					
766.559308	2120471.4	$C_{96}H_{117}N_{31}O_{50}P_{9}S_{9}$	766.559987	0.886	-4	c9 - 4H
766.570383	2259530.8					
766.599021	13251050.3					
766.617538	2218308.5					
766.629099	2633976.1					
766.664168	2337360.2	$C_{121}H_{149}N_{36}O_{65}P_{11}S_{11}$	766.662765	-1.830	-5	b12 - B(dC) - NH3
766.674746	2803935.7	$C_{121}H_{155}N_{39}O_{62}P_{11}S_{11}$	766.677051	3.006	-5	z12 - B(G) + 1H
766.690267	2702162.2					
766.709871	10302585.7					
766.727220	2298247.2					
766.736073	2971896.1					
766.753360	6747767.9	$C_{76}H_{95}N_{21}O_{39}P_{6}S_{6}$	766.754257	1.170	-3	a7 - 2H, b7 - H2O
766.771698	2397581.4					
766.786353	3099421.9					
766.804256	2473975.0					
766.821253	6274008.7					
766.854889	3120224.6					
766.875622	3944690.7	$C_{121}H_{154}N_{38}O_{63}P_{11}S_{11}$	766.873854	-2.305	-5	a12 - B(T) + 2H
766.893792	3310180.6					
766.914077	3759782.2					
766.931663	4293272.8					
766.945012	3489362.9					
766.953696	3877626.5					
766.972928	3986802.1					
766.986408	3736664.8					
767.006257	4161383.1					
767.021789	4587019.4					
767.041195	4943503.7					
767.056040	4440188.1	$C_{71}H_{91}N_{20}O_{40}P_{7}S_{7}$	767.056382	0.446	-3	w7 - B(dA) - 2H
767.114789	4995983.6					
767.128457	5098560.3					
767.148610	6199769.4					
767.178183	6631435.9					

767.211453	14350596.5					
767.229466	11360473.6					
767.241873	11618142.8					
767.262443	222047526.9	$C_{121}H_{150}N_{35}O_{66}P_{11}S_{11}$	767.262699	0.334	-5	b12 - B(A) - 4H
767.285734	16870471.9					
767.294989	14728073.6					
767.309831	16195969.8					
767.324213	42034796.5					
767.341398	31702363.4					
767.373809	716474776.7					
767.403635	32071767.9					
767.435111	73860612.1					
767.450305	46150230.4					
767.462600	52019126.3					
767.485102	1265463328.0					
767.507995	62891969.5					
767.546637	93318894.0					
767.596192	1630032964.0					
767.626958	63195576.9					
767.641596	42681428.6					
767.657797	103963924.9					
767.672803	63905629.8					
767.687654	79314927.2					
767.707599	1690442984.0					
767.768268	104091117.3					
767.787465	51123877.9					
767.849930	55160123.4					
767.879429	87610450.2					
767.896258	45970103.8					
767.930045	1119068508.0					
767.957614	46585594.5					
767.969230	22576520.5					
767.990770	67230563.8					
768.015884	27977467.8					
768.041067	803717200.6					
768.072482	30581480.7	$C_{96}H_{123}N_{31}O_{50}P_9S_9$	768.071724	-0.987	-4	c9 + 2H
768.101996	51124579.1					
768.119892	24950635.1					
768.152501	575466569.2					
768.181931	25714051.4					
768.197221	12122105.0					
768.213772	34202749.0					
768.228870	14851754.8					
768.242053	16719047.9					
768.263569	375054403.4					
768.325351	21962500.2					
768.344234	10093619.5					
768.374648	225181697.6					
768.404996	8933948.6					
768.436558	12055206.8					
768.455349	5435290.6					
768.485937	127447492.6					
768.514035	5404641.1					
768.527727	3639340.7					

768.549147	7324950.5					
768.570284	3546163.0					
768.625365	3858409.2					
768.633849	2940872.2					
768.658831	4602198.4					
768.680634	2541468.5					
768.708337	40545200.4					
768.744452	2123320.3					
768.756252	2637375.0					
768.785846	5096026.8					
768.819846	21464261.7					
768.848213	2356250.8					
768.861058	2239916.5					
768.883468	2239120.3					
768.907781	5448108.0					
768.930841	10853449.9					
768.956831	3124943.6					
768.977440	1743895.0					
769.001672	1750704.1					
769.042430	8408158.8					
769.058421	2078191.4					
769.123518	1939109.5					
769.151150	5413774.9					
769.168391	4216262.8					
769.192862	2310585.3					
769.214269	2024984.7					
769.239337	2228859.8					
769.288731	3766648.3					
769.348754	1818052.7					
769.375186	10234975.2					
769.405938	2164215.5					
769.446332	1776330.1					
769.485397	7742042.9					
769.500301	1879278.4					
769.526629	2179282.7					
769.561908	1838636.5					
769.596858	6795918.5					
769.705747	14079550.7					
769.770836	3143169.2					
769.816281	32895378.3	$C_{95}H_{118}N_{35}O_{48}P_9S_9$	769.817560	1.661	-4	x9 - CH3
769.882409	3400520.3					
769.892109	2195158.0					
769.906108	2711467.7					
769.927434	56965789.5					
769.949600	3591183.2					
769.987954	4555290.0					
770.014085	2469194.1					
770.038721	63744751.3					
770.072269	3006868.8	$C_{121}H_{154}N_{38}O_{64}P_{11}S_{11}$	770.072837	0.738	-5	b12 - B(T) + 1H
770.093603	6328890.7					
770.118141	2582463.2					
770.150107	69517503.3					
770.180385	3474300.3					
770.192682	2350741.3					
770.206681	4324717.2					
770.238158	5340077.5					
770.261286	62787818.8					
770.283049	3909304.0					
770.335092	2306589.0					

770.348724	2811847.9					
770.372227	55627949.5					
770.392852	3976690.0					
770.405508	2712759.1					
770.451933	2257352.4					
770.483733	36138896.2					
770.524356	3155632.4					
770.540315	2559077.7					
770.560418	2234250.8					
770.594812	23699270.0					
770.633909	1784288.4					
770.650968	2221250.5					
770.684956	2108053.0					
770.705494	14604235.7					
770.726334	1980493.1					
770.766188	1939049.0					
770.798907	24187683.1					
770.893512	2966109.0					
770.921240	4562337.3					
770.942588	46568039.5					
770.962921	2799686.3					
771.035352	5590677.4					
771.049159	3666918.0					
771.085193	59925657.0					
771.106770	2398310.6					
771.142471	2327309.7					
771.177397	4700517.9					
771.194566	2794168.9					
771.227948	58908944.5					
771.250695	2798139.7					
771.269583	2161028.1	$C_{121}H_{150}N_{41}O_{62}P_{11}S_{11}$	771.270456	1.132	-5	z12 - B(dC) - 2H, y12 - B(dC) - H2O, y12 - B(T) - NH3
771.320716	2557635.0					
771.371094	54764215.9					
771.422563	1822834.7					
771.514000	38396693.5					
771.591661	12613808.5					
771.622774	1831336.4					
771.656592	26473407.7					
771.701560	20897044.0					
771.746666	3179007.9					
771.756016	3483587.1					
771.795643	12751182.4					
771.814571	24295743.9					
771.891348	2083498.0					
771.923591	25783260.5					
771.944881	6454570.9					
771.982481	2936939.4					
772.034775	25023418.8					
772.084804	7680307.7	$C_{76}H_{95}N_{21}O_{40}P_6S_6$	772.085896	1.414	-3	b7 - 2H
772.145567	18372652.0					
772.229021	2115000.0					
772.257316	10922988.3					
772.368337	11085910.6					
772.416546	1955829.0					
772.479130	5815891.0					
772.592757	2907802.0					
772.705938	2033221.1					

773.000791	1952490.0					
773.031008	9392357.1					
773.045547	5551434.7					
773.081488	127140707.3	$C_{172}H_{217}N_{56}O_{88}P_{15}S_{15}$	773.079889	2.068	-7	M - 7H
773.104331	5961721.8					
773.135185	5553249.1					
773.144849	4685361.8					
773.158703	4756291.1					
773.174790	19313981.0					
773.190599	9888102.8					
773.224298	245561126.4					
773.255039	10401533.2					
773.267655	4471998.2					
773.290371	6069013.7					
773.316615	19448748.2					
773.336597	10023743.5					
773.367293	296464981.4					
773.399528	9724953.4					
773.419626	6574535.0					
773.459749	15575045.0					
773.475287	8333916.7					
773.510381	255206763.6					
773.563158	7404690.9					
773.574686	4550694.5	$C_{96}H_{121}N_{35}O_{48}P_9S_9$	773.573428	-1.626	-4	x9
773.587258	4687840.0					
773.601978	12713446.9					
773.620623	7363045.5					
773.653336	173606790.6					
773.683711	6840504.6					
773.705953	4676020.8					
773.720107	3487264.2					
773.744849	10407017.5					
773.796714	99049298.4					
773.849733	3530281.7					
773.887351	7373780.9					
773.905343	3502723.6					
773.940377	57736249.1					
773.967572	2456300.0					
773.994553	1814567.1					
774.033026	4645389.8					
774.082282	23038479.1					
774.225045	10214788.3					
774.367563	5544808.1					
811.076521	3087090.5					
817.541991	1829962.6					
838.161227	3411936.0					
838.289210	8026811.8					
838.663364	2149612.8					
863.547208	2107692.7					
864.293701	3903138.8	$C_{136}H_{174}N_{46}O_{70}P_{12}S_{12}$	864.292120	-1.829	-5	y13 + 2H
902.750562	2065263.0					
908.535000	2468512.2					
908.699141	5235373.3					
908.871934	5017438.0					
909.041090	2628007.0					
958.041309	2042295.6					
958.186695	2220046.3					

967.333684	1864564.3					
967.470591	4661105.1					
967.613112	5874718.9					
967.758174	5589847.4					
984.192873	5086973.0					
984.336008	7175087.2					
984.479466	4749622.4					
986.481750	3661598.7					
986.622582	11994536.6					
986.766019	12423686.2					
986.896634	5962200.5					
986.915472	4606523.7					
986.962939	3561800.9					
986.991586	2741168.2					
1024.346270	378492.0	$C_{126}H_{161}N_{44}O_{66}P_{12}S_{12}$	1024.345086	1.156	-4	w12
1082.311151	2150783.8	$C_{172}H_{215}N_{56}O_{88}P_{15}S_{15}$	1082.311626	0.439	-5	M - 7H
1082.512964	5040763.8	$C_{172}H_{216}N_{56}O_{88}P_{15}S_{15}$	1082.513191	0.210	-5	M - 5H - 4H
1082.913438	2786627.3					
			Abs mean error	1.287		
			Abs mean std dev	0.371		

Table S15 showing peak list, intensity, elemental composition, and possible assignments with mass errors (ppm) of danvatirsen using 2D-UVPD-MS of one scan and no denoising of [M-8H]$^{8-}$ precursor ion (note, noise peaks were included to highlight the effect of denoising).

**Measured *mz***	**Intensity**	**Elemental Composition**	**Theoretical *mz***	**Error (ppm)**	**Charge**	**Assignment**
225.564620	4432978.2					
338.157835	11445873.4					
338.211348	3663335.9					
338.220331	25879588.2					
338.229994	3355645.6					
338.274159	3407926.9					
338.283009	30083773.9					
338.292559	4503527.9					
338.345512	14004153.0					
338.399633	3570902.7					
338.408158	16306357.6					
338.462176	3122436.1					
338.470632	22844711.0					
338.479713	2978684.4					
338.533138	18663184.5					
338.595374	7109467.6					
338.658348	4542706.1					
338.720810	3274095.5					
383.685875	3005234.7					
383.741421	3047159.4					
383.796819	4790834.8					
383.852803	5536303.5					
383.908368	5491036.3					
384.019223	3684546.1	$C_{61}H_{75}N_{14}O_{34}P_6S_6$	384.019125	-0.255	-5	c6 - B(T) - 4H
386.611177	4820919.8					
386.682538	5937741.5					
386.753958	6352443.1					
386.825573	8437067.5					
386.897146	5341620.1					
386.968404	3150199.8					
431.773017	4730673.5					
431.898246	5619314.3					
432.023441	4464920.4					
451.215020	3808466.6					
451.298462	3288824.8					
451.381653	4227436.6					
493.670986	3145803.4					
541.054566	5112410.2					
541.254315	8222990.8					
541.454155	3680355.6					
599.619888	4344628.6					
601.061588	8656425.0					
601.174444	5866803.3					
601.284159	16500222.7					
601.395476	34268726.7					
601.507165	8021247.4					
601.618941	6016479.6					
601.729300	9369557.5					
601.839552	7788598.0					
601.951839	5511524.5					
609.308719	3498098.6	$C_{76}H_{98}N_{25}O_{40}P_7S_7$	609.308004	-	-4	w7

				1.173		
627.759577	4309770.9					
627.850219	9953375.3					
627.940275	4212455.1					
628.032667	4878836.6					
628.122957	7767706.6					
628.213923	8919933.7					
657.594697	4015166.8					
658.097654	3147957.1	$C_{46}H_{57}N_{12}O_{22}P_3S_3$	658.096992	-1.006	-2	a4 - 2H, b4 - H2O
659.438524	3071434.2					
659.565209	4559483.4					
659.689487	10067982.4					
659.815102	10005065.3					
659.938912	5867082.1					
660.065225	3170077.0					
674.323209	8000287.7					
674.448375	15905799.5					
674.573862	22584435.9					
674.698952	29111250.8					
674.824210	27052144.4					
674.948450	22368125.9					
675.073082	13090907.2					
675.198264	12758975.4					
675.323832	6824854.3					
675.447059	6258253.2					
675.573081	3518445.4					
676.203057	3619655.6					
676.235137	3746144.8					
676.252071	4930792.5					
676.260528	4361064.3					
676.283233	15564328.4					
676.295047	13978909.4					
676.320091	449150480.4	$C_{172}H_{217}N_{56}O_{88}P_{15}S_{15}$	676.318994	1.622	-8	M - 8H
676.344306	17375037.8					
676.361047	13575618.6					
676.377876	11799169.8					
676.384426	12660860.3					
676.408122	51817222.3					
676.419286	38577327.3					
676.445508	1125225782.0					
676.468474	42438058.5					
676.479285	25792906.1					
676.486520	31552394.5					
676.496293	20415786.4					
676.505410	23584962.5					
676.514228	23659985.2					
676.532749	96512871.7					
676.543426	57886550.8					
676.570707	1854525468.0					
676.591763	67891764.3					
676.602591	34444926.7					
676.611250	50285717.1					
676.619765	31839898.8					
676.629611	34173202.1					
676.638140	33858821.0					
676.657565	123383411.1					

676.678658	72503957.9
676.695817	2194204109.0
676.712473	92581427.3
676.735930	53893085.2
676.743475	35533788.2
676.753936	35644811.2
676.762426	33900224.8
676.782225	123855911.6
676.798739	55555879.8
676.820855	1881691596.0
676.860665	43596738.0
676.878265	26271431.8
676.886799	25808664.7
676.907262	96441757.6
676.922539	44874404.5
676.945864	1367774834.0
676.969571	41636412.8
676.985673	30560242.8
677.003062	16845822.4
677.010939	18294069.7
677.022231	17719845.6
677.032647	72450755.7
677.046796	33768785.6
677.070872	979973286.7
677.094309	32020874.3
677.111897	21296088.3
677.136865	12897723.1
677.144009	9559570.0
677.157598	48633516.6
677.171331	21147556.3
677.195905	609568604.6
677.219668	21197933.7
677.236566	17549782.8
677.256868	6681177.4
677.282545	29079974.9
677.296051	11749361.1
677.320893	368877443.3
677.356302	6548642.7
677.362266	8076596.7
677.370223	4052381.7
677.381813	4043024.1
677.389304	4311724.4
677.407740	17570670.6
677.423289	9723876.0
677.446116	224005489.8
677.468612	7681294.4
677.487274	5827285.7
677.506046	3246821.6
677.524244	4992724.3
677.535502	3766772.5
677.545883	4228614.9
677.571105	123068616.8
677.611658	3213044.1
677.625464	3709773.3
677.634607	3568458.2
677.653864	3176321.5
677.696212	63732994.3

677.723426	3545545.5					
677.735208	3868673.0					
677.820878	30403183.3					
677.946207	16751425.8					
678.071266	7971883.2					
678.196739	5036525.6					
678.445709	4016997.9					
678.569970	5463928.6					
678.693885	5241741.7					
678.819348	5408154.5					
678.944997	3872184.4					
679.068047	24832625.0					
679.114395	4429997.1	$C_{46}H_{59}N_{16}O_{21}P_3S_3$	679.113507	-1.308	-2	y4
679.153132	3502811.8					
679.193252	52406402.9					
679.279432	4768727.1					
679.318261	75526728.3					
679.404702	5168359.4					
679.443330	79771192.2					
679.530104	4421684.3					
679.568303	66169948.9					
679.655051	3253925.7					
679.693372	43781992.0					
679.818021	32391928.0					
679.943034	20613256.0					
680.068052	8183523.7					
680.193433	4785558.0					
680.318176	3892605.5					
681.063513	11632771.4					
681.188856	18496986.8					
681.314435	18693358.4					
681.439185	21443351.0					
681.564119	9565200.0					
681.689650	10176818.7					
681.813423	5387611.2					
681.939208	3786467.5					
682.062928	4821061.4					
682.189181	3129427.3					
689.038488	3808385.1					
689.237404	2960416.9					
690.536404	3796913.0					
690.637266	7171002.3					
690.737707	10067253.2					
690.835657	7314165.4					
690.937544	21346003.6					
691.038111	14722509.6					
691.134381	4841259.0					
691.236439	11396104.8					
691.436154	4001922.2					
727.674371	2958368.1					
743.583147	3318186.7					
747.738727	3279538.1					
751.935257	2959282.8					
752.480514	3337064.5					
752.703341	3912309.1					
753.933122	4763156.9					
754.074165	4391564.8					
754.218145	6446065.6					
754.360903	4905642.0					

754.503212	3918422.1					
754.642698	3241700.4					
755.949066	3085865.7					
765.713901	3015591.0					
765.821212	4636514.9					
765.931197	5354531.1					
766.756875	3774977.1					
767.091342	4751358.5					
767.327565	4075465.6					
767.372695	6662063.8					
767.438712	5727800.7					
767.485757	49111102.8					
767.507485	3439208.1					
767.548203	6149434.1					
767.596468	39688613.5					
767.660429	7650297.3					
767.708862	40650420.7					
767.772509	4609873.6					
767.820346	15820792.6	$C_{96}H_{122}N_{31}O_{50}P_9S_9$	767.819768	-0.753	-4	c9 + 1H
767.883219	7571085.8					
767.930113	52225490.5					
767.994344	4621573.5					
768.041399	28432899.3					
768.152488	11003381.3					
768.263684	24557870.4					
768.376086	7405030.5					
768.487577	4633303.9					
768.597278	3803506.4	$C_{51}H_{65}N_{15}O_{25}P_4S_4$	768.598191	1.188	-2	z5 - B(dC)
770.037994	4613908.7					
771.086252	6162871.1					
771.229439	3639209.2					
771.371454	4141501.4					
771.480254	3051724.1					
771.512642	3112167.3					
771.810447	3834877.5					
772.079459	3128801.6					
772.146820	3648785.7					
772.258881	3185871.0					
772.369548	4125983.9					
772.937599	3873390.9					
773.035226	3706450.5					
773.081950	13595266.5					
773.177474	5216293.9					
773.224417	40518666.5					
773.321132	7585378.5	$C_{96}H_{120}N_{35}O_{48}P_9S_9$	773.321472	0.440	-4	w9 - H2O
773.367808	60433856.8					
773.463261	10037742.9					
773.511871	59768503.3					
773.533829	4299998.9					
773.606560	10726315.5					
773.654091	83322937.5					
773.699648	3166241.0					
773.749997	7011783.9					
773.796710	13536684.3					
773.891166	5934787.2					
773.939372	39965491.1					
774.083324	19880479.9					
774.227015	11857823.3					
774.366218	5327461.7					

774.511674	5821579.8					
774.796242	3259120.1					
776.507992	4873977.6					
776.651848	7272871.6					
776.794065	10244021.7					
776.939979	3965695.4					
777.220008	3267660.9					
778.645883	3848342.9					
778.788603	7766015.7					
778.931841	6200034.2					
779.073554	6717275.9					
779.220980	3276865.4					
779.358924	3467473.4					
779.503896	6413121.0					
779.649536	3877925.5					
791.480491	4957579.2					
791.680195	5084228.8					
791.880441	7388811.7	$C_{126}H_{159}N_{40}O_{65}P_{11}S_{11}$	791.880875	0.548	-5	a12
792.080059	4727376.4					
809.245586	3188481.8					
809.581634	3653078.4					
817.020900	4317702.0					
818.111884	3223753.8					
819.075977	3781586.2					
819.476516	8527175.5	$C_{126}H_{162}N_{44}O_{66}P_{12}S_{12}$	819.476178	-0.412	-5	w12 + 2H
819.677058	5419794.0					
824.089062	3233847.2					
838.411777	3431233.0					
838.537347	4435107.5					
846.663462	3741691.8	$C_{130}H_{165}N_{41}O_{70}P_{13}S_{13}$	846.664127	0.785	-5	x13 - B(A) - OCH2
846.791853	3629964.7					
846.916257	5467475.8					
847.040939	3450616.5					
847.164643	3345989.1					
847.289799	4148191.0					
847.413415	3975436.1	$C_{81}H_{98}N_{27}O_{41}P_7S_7$	847.413451	0.042	-3	y8 - B(dC) - 4H
860.489615	9124759.4					
860.689859	14405027.4	$C_{136}H_{172}N_{46}O_{69}P_{12}S_{12}$	860.690007	0.172	-5	z13
861.295973	3020640.5					
861.548676	3338284.6					
861.798825	3434080.8					
861.924666	3844193.1					
862.049519	4802487.8					
863.296088	12759855.3					
863.363628	3661256.2					
863.420977	27455807.5					
863.489387	6715713.5					
863.546631	70618663.7					
863.613198	7146466.5					
863.670950	56701990.6					
863.698056	3253742.5					
863.737574	10682828.9					
863.795006	31811137.4					
863.865869	5350471.7					
863.923245	29593047.1					
863.988833	4372746.3					

864.047536	30018786.0					
864.110425	3784236.5					
864.173549	43117996.7					
864.235732	3123675.6					
864.298399	27580100.5					
864.421056	6699241.2					
864.547593	5507905.3					
866.295316	4193850.2					
866.417793	3178036.7					
866.545327	5155109.7					
866.669188	3226038.5					
866.795660	3161346.2					
867.044390	3155638.7					
868.287636	3286253.3					
868.412479	3498312.4					
868.541329	5402233.4					
868.666835	4027127.6					
868.792992	4058035.3					
868.918110	4225133.6					
876.918359	3996472.5					
877.086806	5093324.0					
877.253681	3496399.8					
881.424382	2979311.2					
881.763584	3258398.1	$C_{86}H_{107}N_{26}O_{44}P_7S_7$	881.764150	0.642	-3	b8 - H2O, a8 - 2H
884.778014	3323110.3					
887.099082	3286911.0					
887.601312	3051364.7	$C_{56}H_{74}N_{18}O_{29}P_5S_5$	887.600761	-0.621	-2	w5 + 1H
899.933646	3546896.0					
900.102401	3203339.7					
900.266378	3775906.6					
901.760290	17253985.4					
901.860425	3075151.3					
901.927539	39659553.8					
902.032055	3632398.6					
902.094362	54426803.6					
902.125287	3090601.0					
902.199346	5273139.5					
902.261185	51670610.6					
902.288316	3391364.5					
902.363916	8869288.8					
902.428900	50566165.0					
902.529753	6169131.9					
902.598009	46777023.9					
902.699884	4626140.1					
902.764983	25456028.9					
902.865066	3999736.7					
902.930351	26276175.8					
903.032390	4451918.8					
903.096045	37038239.8					
903.198117	3173038.1					
903.264690	13219065.6					
903.430834	13155618.9					
903.597342	11467493.4					
906.257419	3866925.9					
906.929135	3003223.2					
908.753519	5207769.1					
909.088996	3230425.1	$C_{116}H_{142}N_{38}O_{59}P_{10}S_{10}$	909.089289	0.322	-4	a11 - 4H
909.200776	3358145.0					

909.255000	5205431.8					
922.759516	2983369.2	$C_{86}H_{111}N_{30}O_{44}P_{8}S_{8}$	922.760626	1.203	-3	w8 + 1H
967.902186	3698548.3					
968.044343	3265220.3					
984.908281	3410037.1					
985.054354	2951388.9					
985.198640	3158064.3					
985.337275	3211890.8					
986.766406	4841165.6					
986.910824	15449642.8					
987.054617	8954735.1					
987.120747	3797546.3					
987.195943	19475001.5					
987.262958	3194815.5					
987.340632	19123306.7					
987.402788	3268537.8					
987.483562	31847439.1					
987.626065	22278877.5					
987.767688	10665400.8					
987.912080	11273522.3					
988.055396	9337844.1					
988.198773	3236598.0					
990.049628	4432301.6					
990.338864	3349697.0					
990.479634	3010650.7					
992.474910	3412538.6					
993.332789	3130027.9	$C_{121}H_{155}N_{41}O_{65}P_{12}S_{12}$	993.332314	-0.478	-4	w12 - B(dC) + 2H
1075.110783	3795588.7	$C_{71}H_{90}N_{16}O_{38}P_{6}S_{6}$	1075.110319	-0.432	-2	b7 - B(G) - H2O
1075.613227	2951800.4	$C_{71}H_{91}N_{16}O_{38}P_{6}S_{6}$	1075.614232	0.934	-2	a7 - B(G)
1082.714322	7106031.2	$C_{172}H_{217}N_{56}O_{88}P_{15}S_{15}$	1082.714756	0.401	-5	M - 5H
1082.914554	5968386.2					
1083.113763	9363014.9					
1083.314616	10429991.8					
1083.517601	13720642.1					
1083.717231	4125360.4					
1084.316315	3253062.3					
1084.515983	5229828.9					
1087.711784	4392981.0					
1087.913466	3327117.0					
1090.503205	4306057.1					
1091.108012	4152528.7					
1136.141184	3152910.8	$C_{36}H_{46}N_{13}O_{18}P_{3}S_{3}$	1136.138600	-2.274	-1	w3
1151.391638	5036415.6					

1151.562715	5714845.0					
1151.728448	11301959.5					
1151.893627	6484243.1					
1152.063362	11121553.6					
1152.227750	7846277.0					
1152.390795	5152490.3					
1152.561408	5474172.8					
1152.729334	7238225.8					
1152.895675	3983254.1					
1353.890804	17725725.2					
1354.141868	24843253.8					
1354.392663	31627753.6					
1354.642650	28219927.4					
1354.890543	19147704.5					
1355.142549	18588466.7					
1355.390811	5421681.9					
1355.640789	4497913.3					
1382.066501	3677089.1					
1382.472310	5281479.5	$C_{131}H_{170}N_{38}O_{70}P_{12}S_{12}$	1382.473087	0.562	-3	b13 - B(G) + 2H
1382.679646	5580804.9					
1382.874948	6145954.1					
1383.073935	5358626.2					
1383.274940	3489990.7					
1383.473644	3775984.9					
1861.767207	4008873.3					
1861.907206	7424810.1					
			Abs mean error	0.836		
			Abs mean std dev	0.561		

Table S16 showing peak list, intensity, elemental composition, and possible assignments with mass errors (ppm) of danvatirsen using 2D-UVPD-MS of one scan and no denoising of $[M-7H]^{7-}$ precursor ion (note, noise peaks were included to highlight the effect of denoising).

**Measured *mz***	**Intensity**	**Elemental Composition**	**Theoretical *mz***	**Error (ppm)**	**Charge**	**Assignment**
257.743018	3054017.6					
257.838549	4470294.5					
257.885957	4440145.0					
338.220557	4536924.0					
338.283188	8243577.1					
338.345524	4541607.9					
338.408288	3758850.7					
338.470372	3859704.5					
338.533449	3730898.6					
383.741607	4629790.9					
383.796928	3932545.8					
383.853130	3907723.9					
383.908333	8106623.7					
386.539011	6105768.6					
386.610846	9267559.3					
386.671008	4181881.4					
386.682377	25209057.9					
386.694857	4064044.6					
386.742643	4852775.4					
386.754026	32154720.5					
386.766252	4894855.4					
386.825202	21541311.3					
386.837830	3623072.6					
386.896866	18987130.9					
386.909253	3313955.6					
386.967973	6591003.0					
387.111120	9901828.0					
387.182522	5892286.1					
387.254094	3675868.3					
431.773069	3096885.6					
431.835562	4168550.4	$C_{70}H_{86}N_{18}O_{38}P_6S_6$	431.834731	-1.924	-5	a7 - B(dC) - CH3
431.897815	4548378.6					
451.299125	3199415.0					
451.381818	3909722.6					
493.671343	3096599.1					
541.054834	3822943.9					
541.154239	3226515.8					
541.558064	3112684.4					
601.062245	4380772.9					
601.173569	11183086.7					
601.285089	15183211.9					
601.394608	19315669.2					
601.505789	25583675.3					
601.616886	14428755.7					
601.728315	12399125.9					
601.838914	4895505.1					
601.951441	8095568.1					
602.061745	5360282.7	$C_{96}H_{117}N_{35}O_{47}P_8S_8$	602.062878	1.882	-5	y9 - 4H
627.669575	3232722.6					
627.759070	6592230.0					
627.849781	5104171.8					

627.940575	8710254.6					
628.033012	5637448.0					
628.122889	3371172.4					
628.214427	6858322.0					
658.096656	3459640.5	$C_{46}H_{57}N_{12}O_{22}P_3S_3$	658.096992	0.511	-2	a4 - 2H, b4 - H2O
674.449289	4831828.8					
674.574816	4556358.6					
674.699396	6832336.4					
674.824621	3796775.0					
674.947002	3879218.2					
675.198501	4038278.8					
676.285094	3269783.1					
676.320460	15551372.8					
676.408716	8884701.3					
676.445731	73087061.5					
676.533198	10231596.6					
676.570584	68691221.6					
676.659715	6648565.7					
676.695159	34438424.9					
676.784033	7201544.9					
676.821493	51598120.0					
676.910040	5379938.8					
676.948449	11878370.1					
677.036246	3857524.8					
677.070495	41917446.8					
677.159310	3697258.5					
677.196747	27866366.9					
677.320401	10866290.4					
677.446515	5288286.2					
677.572423	6276702.2					
677.697586	3186574.0					
677.821797	3185150.8					
679.067430	3066444.9					
679.193390	4274700.7					
679.319817	3197339.3					
679.445885	3005778.1					
679.568462	3365939.6					
679.817879	4533856.0					
679.946564	3161114.6					
681.189117	5738013.5					
681.315107	5244729.8					
681.438593	5101994.4					
681.564584	7208783.6					
681.689206	6500138.4					
681.815089	3870640.1					
690.535601	16609825.2					
690.635542	10384274.5					
690.736866	25747169.9					
690.836519	26804443.9					
690.934715	19968697.2					
691.035930	19076640.4					
691.137942	7118024.1					
691.236319	10507995.0					
691.335550	8622694.8					
691.438021	5224004.4					
693.136206	3280168.8					
752.077346	3085700.5					
752.588969	3210466.5					
752.701511	4076241.8					
752.812697	4295192.4					

752.923264	3461498.7					
753.146383	3211561.2					
753.788654	7345333.4					
753.932869	17422002.3					
754.075353	23308653.3					
754.218061	12780099.7					
754.361153	10235168.8					
754.503512	5141778.4					
754.645626	4093328.0					
754.788757	4739214.9					
755.647109	3517469.1					
765.709599	5724461.3					
765.818701	3830052.2					
765.933097	6110335.4					
766.043618	5593989.0					
766.153461	4756891.1					
766.488695	3280794.1					
766.756694	3510616.3					
767.374521	25601728.5					
767.485537	36202389.7					
767.595153	57795763.0					
767.661739	5616130.5					
767.707687	84590585.9					
767.774450	4464286.9					
767.819951	28933444.3	$C_{96}H_{122}N_{31}O_{50}P_9S_9$	767.819768	-0.238	-4	c9 + 1H
767.885116	4744660.9					
767.927401	17248571.7					
767.996047	4314929.1					
768.039620	21597257.3					
768.106318	3080580.4	$C_{51}H_{66}N_{16}O_{24}P_4S_4$	768.106183	-0.176	-2	z5 - B(T)
768.152220	34310910.8					
768.264170	9055329.6					
768.376738	10869930.4					
768.800416	3758458.3					
768.820115	3221256.2					
769.927350	4305951.7					
770.038463	6963822.2					
770.149222	8157867.2					
770.261029	10106314.5					
770.373057	9609075.6					
770.483732	4974667.0					
770.594598	5777336.3					
770.703384	3687287.5					
770.798818	3878533.8					
770.942369	17549308.0					
771.084975	25017550.0					
771.228421	29087617.4					
771.370711	31061934.6					
771.486215	3422544.6					
771.513797	23583326.7					
771.591547	7413004.7					
771.656488	21175190.5					
771.702004	19395433.4					
771.756455	3202968.4					
771.796379	11102816.8					
771.814831	26510497.7					
771.923881	30397259.0					
771.944883	9501654.6					

771.983577	3178561.7					
772.034625	33634421.4					
772.145692	28637608.8					
772.226790	3272657.3					
772.235932	3433782.1					
772.257570	22152962.6					
772.368136	16950607.1					
772.415600	3316792.9					
772.479845	11932098.8					
772.593078	7928718.8					
772.704641	5870014.0					
772.763002	4996962.0					
772.797152	3571085.4					
772.819161	3938595.1					
772.838074	3199515.0					
772.857160	3034624.6					
772.873790	3120125.9	$C_{121}H_{154}N_{39}O_{64}P_{11}S_{11}$	772.873452	-0.437	-5	y12 - B(A)
772.891317	3577380.1					
772.906459	3613432.9					
772.930157	3395018.0					
772.944777	5176003.9					
772.952976	4758900.2					
772.967082	5496038.6					
773.002006	6776294.0					
773.031651	27394671.8					
773.044918	20443934.3					
773.060239	25253770.6					
773.081304	544244552.7	$C_{172}H_{217}N_{56}O_{88}P_{15}S_{15}$	773.079889	1.830	-7	M - 7H
773.134779	21425911.2					
773.145180	19256027.4					
773.158053	21364662.7					
773.175169	70352008.6					
773.190466	48945930.3					
773.224486	1291497066.0					
773.255051	53273660.0					
773.268617	26489516.8					
773.278823	39491350.4					
773.290586	33643003.1					
773.317375	111483969.7					
773.336008	64310615.4					
773.367399	1956203527.0					
773.399689	64954835.9					
773.420525	44841276.0					
773.435755	31806716.4					
773.460283	141616830.2					
773.473812	76827231.6					
773.510628	2159600873.0					
773.533755	82603847.0					
773.563875	61875542.0					
773.574002	38707803.8	$C_{96}H_{121}N_{35}O_{48}P_9S_9$	773.573428	-0.742	-4	x9
773.587629	38408955.4					
773.603098	137907798.4					
773.620103	73584558.6					
773.653476	1870845730.					

	0
773.684224	69499731.3
773.707775	45233435.6
773.720204	34081108.1
773.745161	121203503.0
773.771013	44580805.9
773.796324	1369702825.0
773.828507	41609164.8
773.849391	35744197.2
773.888484	85774045.7
773.905512	38477708.5
773.939281	961637709.0
773.968162	36531780.1
773.982234	16002425.0
773.993364	26497284.5
774.004315	16558688.1
774.031013	54694584.2
774.050888	23926512.7
774.081924	632774415.0
774.113776	20627469.2
774.135308	15555129.6
774.150192	7548584.6
774.174139	33139467.4
774.190593	13193571.1
774.225084	368702019.5
774.278173	10262201.3
774.288616	6237152.6
774.303097	5139427.4
774.317581	20354733.0
774.336332	9044983.3
774.367625	233899670.6
774.398841	7403915.3
774.422325	4911178.5
774.459967	10854660.0
774.510935	128665095.1
774.531660	5414047.7
774.563952	3028036.0
774.598839	5184823.9
774.653703	72817224.8
774.747840	6654400.4
774.796872	38008384.5
774.940277	19460013.4
775.082998	14836975.3
775.105197	3312431.3
775.224193	6146969.4
775.251424	5675569.4
775.346295	3462931.2
775.365444	5923559.3
775.415799	3692257.4
775.509368	4980711.1
775.651729	7430275.8
775.683100	4393480.9
775.794940	9467539.2
775.852504	3661733.0
775.937916	7970665.4
776.082241	5718293.9
776.170329	3172952.3
776.221483	42733631.5
776.274389	3167824.0

776.313517	6640415.1					
776.330479	3953219.8					
776.364678	95115493.5					
776.393825	4363988.6					
776.420185	2995037.9					
776.456378	10263386.9					
776.482621	4214725.2					
776.507488	128444299.7					
776.528721	6747355.6					
776.561027	4120818.8					
776.570451	3049638.1					
776.599104	10050151.7					
776.617371	5923226.6					
776.650423	142473468.4					
776.681658	5843923.9					
776.705166	3573768.8					
776.742400	10604913.4					
776.758314	4571394.7					
776.793336	121760019.6					
776.846784	3511206.7					
776.885502	7354180.2					
776.905564	3455114.8					
776.935997	90752218.0					
777.028900	4735807.7					
777.079187	58290809.8					
777.172440	3119100.6					
777.221843	36278248.0					
777.364284	19305211.8					
777.507942	11971205.6					
777.649732	4196602.6					
778.079414	3957644.9					
778.502388	26005226.8					
778.590434	4024816.9					
778.645592	44797787.1					
778.742568	8270869.9					
778.788833	74074741.7					
778.880027	5466502.9					
778.931657	66026108.9					
779.022518	4498825.3					
779.074242	52826020.1					
779.165931	4082560.0					
779.217231	37897437.8					
779.360142	26094613.4					
779.502775	13932460.8					
779.645547	8753264.3					
779.789467	5190608.6					
785.240832	4020643.9					
819.474598	3167699.5	$C_{126}H_{162}N_{44}O_{66}P_{12}S_{12}$	819.476178	1.928	-5	w12 + 2H
819.675744	3306360.9					
838.286818	3596167.7					
838.540387	3326377.7					
847.164697	5544884.8					
847.290049	3106252.4					
848.041220	3516253.7					
858.078859	3348134.4					
858.245931	4301522.7					
861.424225	3174948.0					
861.545404	4917167.5					
861.675885	3351916.1					

861.799066	4338150.2					
861.926247	7133959.6					
862.047084	4679740.8					
862.173959	6549358.4					
862.298911	3900412.8					
863.294908	6857935.7					
863.421897	18436727.9					
863.485038	3725184.6	$C_{136}H_{170}N_{46}O_{70}P_{12}S_{12}$	863.485860	0.952	-5	y13 - 2H
863.550536	15423093.6					
863.606030	3038803.3					
863.673384	35359392.5					
863.738775	4649346.2					
863.797382	23715647.9					
863.862342	5749217.7					
863.923008	20216200.7					
863.989063	5384521.0					
864.045614	33730529.3					
864.110900	3755997.9					
864.170644	31426406.1					
864.297230	10268272.1					
864.423670	4386946.8					
864.548533	6652309.5					
864.671123	4265382.1					
866.419410	5060901.6					
866.545614	4554690.8					
866.794296	4026703.1					
866.921676	4045925.6					
868.286379	3092324.5					
879.921558	3122296.7					
880.256587	3299492.1					
881.429472	3629105.4					
882.098480	5568694.9					
882.433468	3944008.3					
887.099613	5274598.1					
887.599379	3594033.9	$C_{56}H_{74}N_{18}O_{29}P_5S_5$	887.600761	1.557	-2	w5 + 1H
902.094651	5175429.0					
902.201155	4101773.7					
902.261157	11544671.9					
902.369096	3747892.0					
902.428117	34385801.5					
902.530565	4255616.4					
902.598862	23433822.2					
902.694875	5172260.1					
902.764576	35348980.2					
902.863224	3718916.9					
902.933822	7416807.1					
903.095854	28958001.7					
903.261844	21830635.0					
903.430702	4806344.7					
905.925429	3073164.0					
906.260135	5627168.8					
908.753685	5611756.3					
909.594171	3155045.0	$C_{116}H_{144}N_{38}O_{59}P_{10}S_{10}$	909.593201	-1.066	-4	a11 - 2H, b11 - H2O
909.845609	4348319.5					
922.425050	5918341.6	$C_{86}H_{110}N_{30}O_{44}P_8S_8$	922.424684	-0.397	-3	w8
923.095746	4241424.0	$C_{61}H_{77}N_{14}O_{33}P_5S_5$	923.096179	0.469	-2	b6 - B(T) - 2H
967.902837	4151589.1					

968.044500	3734020.7					
968.333185	3043272.2					
978.621335	3440072.3					
986.765884	5960020.0					
986.909570	8953948.3					
987.050642	17746801.1					
987.192503	13556670.8					
987.341248	26739502.1					
987.484432	23429293.1					
987.624034	14436886.9					
987.768381	16992280.7					
987.910174	13805289.2					
988.052659	12029292.8					
988.194762	4052850.4					
988.338898	3481989.4					
988.483293	3825607.2	$C_{157}H_{200}N_{41}O_{84}P_{15}S_{15}$	988.482997	-0.299	-5	M - 5H - H2O - 3G
990.476305	3320268.7					
990.624617	3067207.2					
992.757896	3719915.1					
1024.345217	3783891.7	$C_{126}H_{161}N_{44}O_{66}P_{12}S_{12}$	1024.345086	-0.128	-4	w12 + 1H
1052.303290	5622486.1	$C_{167}H_{211}N_{51}O_{87}P_{15}S_{15}$	1052.303309	0.018	-5	M - 5H - 4H - G
1052.503999	7405075.9					
1052.702795	7833578.0					
1052.904320	5294575.1					
1055.901830	3100235.0					
1075.110112	3267878.0	$C_{71}H_{90}N_{16}O_{38}P_{6}S_{6}$	1075.110319	0.193	-2	b7 - B(G) - H2O
1075.863462	3326007.0					
1076.362789	3645160.8					
1082.312570	10466432.4	$C_{172}H_{215}N_{56}O_{88}P_{15}S_{15}$	1082.311626	-0.872	-5	M - 7H
1082.615485	3083246.9					
1082.912544	36286291.5					
1083.017721	3002794.0					
1083.113847	36452864.1					
1083.315136	17896598.9					
1083.421040	3103759.3					
1083.516125	21661937.4					
1083.717240	12776943.2					
1083.915098	10887387.2					
1084.112361	4803423.8					

1084.312837	5945151.7					
1087.713033	5082201.7					
1087.906141	4270487.7					
1088.107623	3107436.5	$C_{71}H_{88}N_{18}O_{38}P_6S_6$	1088.105568	-1.889	-2	b7 - B(T) - NH3, b7 - B(dC) - H2O
1091.308416	3665822.8					
1136.137297	3295350.0	$C_{36}H_{46}N_{13}O_{18}P_3S_3$	1136.138600	1.147	-1	w3
1151.561761	3741146.2					
1151.729595	6737100.1					
1151.894022	17000089.1					
1152.061501	18328471.2					
1152.233627	5446595.1					
1152.389530	8271059.4					
1152.725244	3471172.8					
1353.890642	5310646.4					
1354.135808	6076402.0					
1354.389012	4319268.8					
1354.639589	4682717.7					
1355.140188	5207944.7					
1382.476989	3860447.3					
1382.672225	6238887.5					
1382.868528	6115882.6					
1861.939050	3760599.8					
			Abs mean error	0.959		
			Abs mean std dev	0.630		

Table S17 showing peak list, intensity, elemental composition, and possible assignments with mass errors (ppm) of danvatirsen using 2D-UVPD-MS of eight scans accumulated denoised (sane rank 10) [M-8H]$^{8-}$ precursor ion (note, noise peaks were included to highlight the effect of denoising).

Measured *mz*	Intensity	Elemental Composition	Theoretical *mz*	Error (ppm)	Charge	Assignment
225.438894	11759881.8					
225.480452	20634793.2					
225.522342	22940480.3					
225.560402	15049862.9					
225.564129	30139274.8					
225.606010	31214371.5					
225.647282	15226010.1					
225.689377	11952232.0					
255.829077	19316912.4					
255.866098	23910769.7					
255.903160	25059992.8					
255.940238	24048392.3					
255.977294	17413053.6					
256.014316	13404631.2					
257.694575	12111649.4					
257.742248	24770340.7					
257.789898	27844872.7					
257.832177	11282582.8					
257.837472	30063962.0					
257.879829	10832184.7					
257.885183	26087329.1					
257.932820	18189140.3					
287.809916	12224024.4					
287.851524	19088202.0					
287.893375	23352918.3					
287.935039	23404561.1					
287.976749	18066402.6					
288.018354	12791091.4					
300.757856	12114244.0					
300.813646	14876660.7					
300.869299	14157373.4					
300.925009	11930000.1					
338.149141	10712040.1					
338.157663	87496660.8					
338.211932	25313685.0					
338.220417	179438609.1					
338.229914	25158880.2					
338.274382	42462733.5					
338.282946	239275059.3					
338.289055	12630329.4					
338.292419	29952086.6					
338.336868	19068334.8					
338.345227	123987189.9					
338.354953	14619717.5					
338.399557	25423334.6					
338.408134	136304451.1					
338.417649	16033759.5					
338.462448	23211803.7					
338.470541	164508897.3					
338.480134	18904252.7					
338.524719	18800334.1					
338.532957	116778531.4					

338.542190	13894130.8					
338.595473	50540979.9					
338.658052	22870862.8					
338.720632	28422035.2					
338.783508	19795351.5					
339.531323	10858874.0					
339.656903	15955030.8					
339.719531	22426611.3					
339.782073	19636334.9					
339.843803	11417560.0					
345.416177	11205086.2					
345.465623	10793357.8					
383.685380	14716396.0					
383.741213	17070903.4					
383.796586	17608415.3					
383.852286	24222443.1					
383.907967	24711214.2					
383.963316	16825734.4					
386.610815	19282546.1					
386.682605	26562856.6					
386.753211	24873864.5					
386.825391	27988552.3					
386.897146	13175529.8					
386.968469	14930597.8					
387.039541	11078221.4					
431.709955	15912016.4					
431.772121	17833350.7					
431.898385	17286147.8					
431.960695	16153512.0					
432.023703	16884895.7					
432.086242	12546612.9					
451.047579	13153659.7					
451.215157	16094341.2					
451.298468	10755289.6					
451.381117	15957339.9					
451.465164	14513489.1					
493.528031	12893472.7					
493.599935	11644258.1					
493.671163	12400816.6					
541.153697	12374991.5					
541.355479	10756654.8					
601.062029	14595940.1					
601.171233	25327542.0					
601.282872	40403789.2					
601.394917	92949580.8					
601.506636	82231459.5					
601.617600	58733483.0					
601.728632	52108203.6					
601.839887	31549002.7					
601.949923	22619237.6					
602.172615	12713756.9					
603.948997	14164293.4					
604.059083	13391735.6					
605.500416	14636254.1					
609.559319	13537658.8	$C_{76}H_{99}N_{25}O_{40}P_7S_7$	609.559960	1.052	-4	w7 + 1H
609.809664	10899912.7					
627.666897	11054005.1					
627.849803	24703999.1					
628.032160	15285161.7					
628.122443	20155409.5					

628.211554	15632809.5					
657.594107	16071412.9					
657.814485	13297545.3					
657.939406	11246564.8					
658.096570	19999589.3	$C_{46}H_{57}N_{12}O_{22}P_3S_3$	658.096992	0.641	-2	a4 - 2H, b4 - H2O
659.438772	15934196.6					
659.564405	31480666.9					
659.689051	56689889.6					
659.814158	71027739.8					
659.939718	38934465.7					
660.064439	30666402.6					
660.190322	17446197.9					
660.315540	12373038.4					
674.322732	58835771.6					
674.426800	19389511.2	$C_{66}H_{85}N_{23}O_{32}P_5S_5$	674.426811	0.016	-3	y6 + 1H
674.447992	126915113.7					
674.573359	223545789.8					
674.663555	12075475.4					
674.698183	243971952.9					
674.785443	13397877.6					
674.798655	10617579.7					
674.823262	238662689.2					
674.910511	13483108.4					
674.948469	198142699.3					
675.037127	11218940.4					
675.073201	140015943.5					
675.197719	99927066.2					
675.323510	62319977.8					
675.448337	41352931.9					
675.573971	20435996.4					
675.695040	13328474.6					
675.895646	11420838.4					
676.065715	13189434.3					
676.075682	12308493.9					
676.092080	10842609.9					
676.100429	11111300.4					
676.110337	11349554.3					
676.118526	12715203.9					
676.128294	12116991.3					
676.136328	13168017.5					
676.145405	13258734.0					
676.154486	14586209.5					
676.163228	13046430.0					
676.171489	15235822.9					
676.182074	16363957.0					
676.200437	20054946.6					
676.206624	21674916.1					
676.216863	20947177.6					
676.224569	21557275.7					
676.234155	27540640.5					
676.243095	25811434.0					
676.260331	43927533.2					
676.271067	57832387.5					
676.284199	266512846.3					
676.319538	3711907137.0	$C_{172}H_{217}N_{56}O_{88}P_{15}S_{15}$	676.318994	0.804	-8	M - 8H
676.344221	122748138.1					
676.357581	105215032.7					
676.364999	86789533.3					
676.377036	93934400.6					

676.384853	118898764.8
676.394530	139707029.2
676.409694	681588094.7
676.445016	8946301569.0
676.468470	313852942.8
676.479144	227731385.2
676.486099	222884341.3
676.495918	175606497.7
676.507075	180599524.6
676.534611	1100705827.0
676.570285	14380099779.0
676.591569	511561143.3
676.603100	336986336.0
676.610839	359014971.1
676.620157	276203590.0
676.629859	261079230.4
676.638203	272598834.7
676.659515	1304192660.0
676.695440	17367096566.0
676.726580	353435851.9
676.735440	411562048.5
676.744341	308235577.6
676.754013	277297230.9
676.762469	295354426.1
676.784195	1201765070.0
676.820452	15681695238.0
676.860069	352465332.8
676.868327	260396222.5
676.878288	226356326.1
676.886846	247095927.5
676.897894	261595134.5
676.908859	942932701.8
676.922612	390204927.6
676.945455	11780666661.0
676.984792	246655934.5
677.002517	154393241.2
677.011312	170076783.2
677.021109	180209311.3
677.033985	664340382.2
677.046859	281418317.0
677.070428	8134591761.0
677.094059	233801278.3
677.109566	160945079.8
677.136844	125694943.2
677.144908	110006933.3
677.158949	455099725.8
677.171020	182582823.0
677.195462	5328473713.0
677.219211	172919024.3
677.235790	128669115.7
677.259646	60592558.5
677.269382	62354637.2
677.283984	272667448.5
677.295124	107350327.9
677.320477	3226159269.0
677.356370	58459941.3

677.361785	62646460.9					
677.370367	42223713.5					
677.389604	36580606.4					
677.408965	154163562.6					
677.422944	75908193.5					
677.445660	1864000626.0					
677.468202	63876438.1					
677.480807	37466371.6					
677.486267	38981509.7					
677.495574	25988168.9					
677.506891	22988654.2					
677.523063	41734965.4					
677.536474	55327023.7					
677.544742	35182583.7					
677.570750	1004292209.0					
677.605407	21451896.3					
677.612047	25253645.6					
677.624463	29162445.6					
677.635066	32143943.7					
677.658773	27185132.0					
677.670561	21100586.1					
677.695757	508171581.0					
677.718316	22570678.5					
677.735862	22667412.9					
677.745075	11447705.9					
677.754819	11147948.8					
677.783352	25030779.3					
677.796265	11388058.9					
677.820548	272107067.6					
677.856666	37590108.6	$C_{106}H_{134}N_{33}O_{56}P_{10}S$ 10	677.857433	1.132	-5	c10
677.908060	12948281.7					
677.924985	11547625.5					
677.945790	136367762.4					
678.054865	27482765.4					
678.071362	68274249.5					
678.196496	40678460.0					
678.257760	22100285.6					
678.320804	31551900.7					
678.444724	38127797.1					
678.461484	13748390.7					
678.569144	45229537.7					
678.693954	47326681.8					
678.819043	39278509.2					
678.944755	33658333.9					
679.031491	33309319.4					
679.067609	521054386.8					
679.088456	22697357.7					
679.109895	32356576.1					
679.115862	30150081.3					
679.136317	15111139.4					
679.156502	75507154.7					
679.192894	1121813027.0					
679.233697	30738420.3					
679.242638	23875919.4					
679.251840	21604404.3					
679.261018	21643683.6					
679.281731	104481654.8					
679.318004	1551603804.0					
679.351247	30298244.6					

679.358896	37228438.4
679.367644	30116232.8
679.377614	25943381.7
679.386449	26152981.1
679.406588	116181943.1
679.443111	1665021285.0
679.477068	29503998.8
679.484400	40143350.2
679.492913	28943202.0
679.502865	25981848.1
679.512120	25165927.8
679.531512	101597466.4
679.568159	1454425778.0
679.590065	52408377.6
679.603188	25677605.1
679.609115	35138598.6
679.621090	22555202.2
679.637228	20783647.9
679.656553	76154575.9
679.693064	1065668903.0
679.716039	36186730.5
679.734039	22374825.1
679.743518	15822832.5
679.754435	12409352.5
679.767175	12649881.1
679.781435	49003125.6
679.792399	20871344.3
679.818020	708900705.2
679.856743	13389746.4
679.906228	28847986.7
679.919178	12229698.3
679.942976	408325173.6
680.031053	13233507.7
680.067943	220644153.7
680.193143	123681833.8
680.317936	61615172.0
680.443133	31140700.6
680.567642	13393086.8
680.695638	10529179.8
681.062511	65960806.0
681.188086	148978038.0
681.277277	14089977.3
681.313049	181916727.0
681.402808	16046823.2
681.438250	163797937.3
681.527091	11746415.9
681.563568	159464226.1
681.655196	14862742.7
681.688237	121524792.4
681.813069	79257822.0
681.938496	60774134.5
682.063365	44962694.0
682.189190	30739064.1
682.315078	17995287.0
682.393571	11842820.2
682.441055	13083775.1
682.562024	17503484.3
682.728178	12759628.7
689.239118	16368977.8
690.434825	11744994.7

690.534668	32984040.6					
690.634684	71673669.2					
690.698291	14692772.1					
690.734291	107736935.6					
690.798875	15918413.3					
690.835456	140156056.1					
690.896913	13256806.4					
690.933217	71607449.0					
691.034986	56522436.9					
691.135091	60528765.5					
691.236486	29588016.3					
691.336755	24719777.6					
691.437744	12941558.8					
691.536513	11385122.5					
692.732939	11331566.9					
693.034607	13171241.5					
693.234360	12545221.7					
727.874134	11470268.4					
728.076194	10994359.4	$C_{71}H_{93}N_{16}O_{40}P_6S_6$	728.075556	-0.876	-3	b7 - B(dA) + 1H
743.330883	20099105.9	$C_{96}H_{119}N_{31}O_{48}P_8S_8$	743.329984	-1.209	-4	a9 - 2H, b9 - H2O
743.581969	18751672.4					
743.831196	14154047.8					
747.403952	10525565.7					
747.571049	14093538.6	$C_{51}H_{61}N_{10}O_{27}P_4S_4$	747.569771	-1.710	-2	a5 - B(dC) - NH3
747.739094	14147142.8					
751.788679	10522301.3					
752.479474	10795332.2					
752.700694	11319363.1					
754.071483	12832265.7					
754.361293	16764074.2					
754.504564	20615324.8					
756.234557	11248140.2					
765.820788	13216401.4					
765.928777	15265388.8					
766.754813	21400666.4	$C_{76}H_{95}N_{21}O_{39}P_6S_6$	766.754257	-0.725	-3	a7 - 2H, b7 - H2O
767.090072	25679505.9					
767.328480	10870517.4					
767.374060	95589773.4					
767.421659	22242280.7					
767.441890	20584379.3					
767.485528	170532977.5					
767.553228	16827398.5					
767.595889	203220826.8					
767.661685	21401555.9					
767.706556	194829219.4					
767.760032	12644328.9					
767.771620	12862505.9					
767.817120	119023306.8					
767.880746	11726289.3					
767.927148	72998483.9					
767.993652	16624008.9					
768.041224	127453290.2					
768.154166	65256243.5					
768.264819	57959751.5					
768.375062	58846487.3					
768.482947	14854398.6					

769.927028	22566059.5					
770.039040	55332377.4					
770.151122	27761336.5					
770.260115	27381435.4					
770.371894	23922016.1					
770.481477	11952994.3					
770.705026	12655137.3					
770.943865	11937421.5					
771.085485	14104429.4					
771.811723	11100856.5					
772.937302	19553552.6					
773.081377	83841335.2					
773.179428	23110321.9					
773.221892	114279276.9					
773.319835	37879631.6	$C_{96}H_{120}N_{35}O_{48}P_9S_9$	773.321472	2.117	-4	w9 - H2O
773.365251	208624448.9					
773.461746	46286790.2					
773.508565	200306047.2					
773.609642	23828721.6					
773.654269	327312237.0					
773.686418	11190123.8					
773.751538	14201239.8					
773.795456	144616478.8					
773.939885	116487333.7					
774.080739	82800964.4					
774.223956	53876855.3					
774.366453	25057397.1					
774.510048	13631332.2					
776.221913	23871472.9					
776.363803	16221016.4					
776.508195	34803242.1					
776.651119	36390154.6					
776.793857	39831187.2					
776.935706	28614738.2					
777.079887	31446013.1					
777.222317	25395824.3					
777.364834	15172564.6					
778.644374	20145643.4					
778.788685	20256346.5					
778.931123	13069299.8					
779.074677	21318611.6					
779.217346	13510469.8					
779.361339	15122213.5					
779.501722	11942639.0					
779.788329	11122074.5					
791.478732	31005382.4	$C_{126}H_{157}N_{40}O_{65}P_{11}S_{11}$	791.477745	-1.247	-5	a12 - 2H, b12 - H2O
791.679388	39306896.2					
791.879582	41998413.0	$C_{126}H_{159}N_{40}O_{65}P_{11}S_{11}$	791.880875	1.633	-5	a12
792.080093	31520186.4					
792.280796	17177177.6					
809.246303	16644133.9					
809.412342	23216125.3					
809.579503	19044517.5					
809.746416	16467742.3					
812.747574	12870647.3	$C_{76}H_{98}N_{25}O_{40}P_7S_7$	812.746430	-1.408	-3	w7
818.107316	16011662.3	$C_{56}H_{70}N_{14}O_{28}P_4S_4$	818.108589	1.556	-2	a5 - 2H, b5 - H2O
819.275073	52596087.8	$C_{130}H_{165}N_{38}O_{68}P_{12}S$	819.275150	0.094	-5	a13 - B(G) -

		12				OCH2
819.475355	64369570.3	$C_{126}H_{162}N_{44}O_{66}P_{12}S_{12}$	819.476178	1.004	-5	w12 + 2H
819.675503	47806977.1					
819.876282	17703045.9					
823.837336	13942329.4					
840.345743	11777147.7	$C_{106}H_{134}N_{40}O_{52}P_9S_9$	840.347617	2.230	-4	y10 + 1H
846.915599	12597742.3					
847.039170	15354322.8					
860.488881	69397072.9					
860.689231	81249951.9	$C_{136}H_{172}N_{46}O_{69}P_{12}S_{12}$	860.690007	0.902	-5	z13
861.090208	34738544.0					
861.290736	14139747.8					
861.549009	14333955.6					
861.670962	11743795.8					
861.798485	12390528.4					
861.923809	13691754.1					
862.047268	15417829.2					
862.173728	18705763.6					
863.295052	48621105.1					
863.422678	67101568.7					
863.546842	149716915.4					
863.613880	17506733.7					
863.671945	198630692.7					
863.738329	26302576.3					
863.796472	160819029.4					
863.863499	19471556.7					
863.920402	98944582.4					
863.988252	19054082.2					
864.046350	133101730.6					
864.113713	15242110.1					
864.171555	123970255.3					
864.297026	84985989.4					
864.423167	51105170.6					
864.545709	16347984.5					
864.796836	11574765.8					
866.170126	11205622.0					
866.293670	13299150.7					
866.419250	36824019.5					
866.542522	19828941.7					
866.667197	29689839.7					
866.794667	21501471.5					
866.919828	14627965.8					
867.044321	22128299.6					
868.164424	11102665.6					
876.751483	17414368.2					
876.919118	23027610.7					
877.085663	21365685.1					
877.252823	21270245.0					
877.419610	13403425.6					
879.585372	13079132.7	$C_{55}H_{70}N_{18}O_{29}P_5S_5$	879.585111	-0.297	-2	w5 - CH3
879.751081	14340136.5					
880.254453	12233948.4					
881.250354	12827221.0					
881.419977	15443818.8					
881.761280	22197794.6					
882.099530	24182612.9					
882.434797	10603844.1	$C_{86}H_{109}N_{26}O_{44}P_7S_7$	882.436033	1.401	-3	a8

884.775025	18390093.1					
885.106853	15700664.7					
887.097234	18021759.8	$C_{56}H_{73}N_{18}O_{29}P_5S_5$	887.096848	-0.435	-2	w5
899.597965	13288820.7					
899.765799	15149942.2					
901.695091	11478893.7					
901.759553	122778873.3					
901.862887	24223042.1					
901.898376	12002471.4					
901.926704	303927230.3					
901.958898	13331222.1					
902.029647	37200899.8					
902.093255	400320716.2	$C_{61}H_{77}N_{11}O_{33}P_5S_5$	902.091568	-1.870	-2	a6 - B(dA) - H2O
902.124231	19917386.3					
902.196690	44944276.3					
902.260192	376572874.8					
902.306329	11322104.3					
902.362648	48543755.7					
902.427629	397904510.0					
902.471546	12349761.9					
902.529871	22798121.4					
902.699833	16372616.5					
902.762951	232565056.8					
902.868574	15362958.7					
902.927711	100337238.1					
903.093095	74022499.9					
903.261488	48022782.5					
903.430430	32397493.9					
903.595352	17466982.6					
903.763802	17404714.2					
905.422936	10755858.9					
905.590564	25794471.1					
905.757559	32705607.4					
905.923863	32808430.6					
906.258433	36141482.7					
906.424145	24764250.2					
906.592660	24523279.4	$C_{115}H_{144}N_{38}O_{59}P_{10}S_{10}$	906.593201	0.597	-4	b11 - OCH2
906.756599	12987119.9					
908.420432	13396279.1					
908.586620	14270513.7					
908.751229	15828301.7					
908.921424	16875758.2					
909.088063	11416212.5	$C_{116}H_{142}N_{38}O_{59}P_{10}S_{10}$	909.089289	1.349	-4	a11 - 4H
922.426338	14966400.4	$C_{86}H_{110}N_{30}O_{44}P_8S_8$	922.424684	-1.793	-3	w8
958.472703	10977358.2					
985.197706	12217350.2					
985.343104	12316712.4					
986.764560	14788277.2					
986.910194	70640366.5					
987.054373	95871928.5					
987.197583	89834473.2					
987.265537	10929505.4					
987.339235	63632860.1					
987.482081	53574058.1					
987.625826	56153590.8					

987.766926	33654666.8					
987.909402	28935358.3					
988.051253	17882166.5					
988.197215	14403989.7					
990.196256	12650624.9					
990.333454	11899469.8					
990.481376	27181195.8					
990.623133	18573180.6					
990.767010	10900212.2					
1082.714816	30309872.0	$C_{172}H_{217}N_{56}O_{88}P_{15}S_{15}$	1082.714756	-0.055	-5	M - 5H
1082.914806	29851596.4					
1083.114002	73156029.5					
1083.311899	57383994.4					
1083.515980	47861205.6					
1083.714804	31053941.0					
1083.916631	30976967.3					
1084.114541	20420216.0	$C_{71}H_{92}N_{16}O_{39}P_6S_6$	1084.115602	0.979	-2	b7 - B(G), a7 - B(dA) + 1H
1084.311467	13569367.1					
1084.517429	13212094.5					
1087.310274	16784253.0					
1087.509011	11913049.3					
1087.711365	12913778.6					
1087.909101	12245671.9					
1088.112408	23228179.9					
1136.133823	10712712.6					
1151.394088	12282233.0					
1151.559459	14788016.2					
1151.729448	22131812.5					
1151.895972	45249656.7					
1152.061420	39570943.3					
1152.227059	41326302.8					
1152.395379	19584780.9					
1152.562791	27995687.2					
1152.729598	15200096.2					
1155.72491	16103813.3					

1			
1155.891147	14244689.0		
1156.059516	11956896.0		
1353.639485	10985717.6		
1353.888713	25053416.2		
1354.138878	17623126.5		
1354.388153	20485819.9		
1354.638781	18375871.6		
1354.891306	25336053.4		
1355.138745	12319478.2		
1359.635277	17646862.6		
1359.884047	13157710.4		
1360.133001	12078421.2		
1382.874313	12593353.7		
1383.070901	16593731.3		
1861.939305	19587310.2		
		Abs mean error	1.137
		Abs mean std dev	0.583

Table S18 showing peak list, intensity, elemental composition, and possible assignments with mass errors (ppm) of danvatirsen using 2D-UVPD-MS of eight scans accumulated denoised (sane rank 10) [M-7H]$^{7-}$ precursor ion (note, noise peaks were included to highlight the effect of denoising).

Measured *mz*	Intensity	Elemental Composition	Theoretical *mz*	Error (ppm)	Charge	Assignment
257.838424	20418300.2					
257.885993	18954883.9					
338.345496	19843117.6					
383.906832	17600333.8					
383.962441	23400248.7					
386.539385	46162125.0					
386.599523	22250355.7					
386.610572	81275075.9					
386.671224	33975331.3					
386.682332	152304949.3					
386.694558	17824673.3					
386.742680	40383695.2					
386.753884	222242482.6					
386.766051	26371694.0					
386.814040	40791443.1					
386.825362	182379635.2					
386.837682	19728741.3					
386.885086	22547274.7					
386.896645	112698181.3					
386.968167	43485391.9					
387.039690	65234864.7					
387.111155	66467387.3					
387.182570	43044557.6					
387.254056	19100942.7					
388.180853	23779861.4					
388.323988	26649484.9					
388.395359	32599042.5					
388.466915	22129958.6					
601.061295	31577605.1					
601.172952	114693357.9					
601.285123	83567964.7					
601.395328	145168596.6					
601.507292	79735865.3					
601.617696	25533326.8					
601.728672	47559978.1					
601.840651	43133191.5					
603.837275	16066612.9					
604.059850	20520937.3					
627.758825	22279152.6					
627.850963	24471178.4					
627.941962	17386690.8					
628.122600	20869765.0					
674.697369	17550213.6					
674.823429	17912579.6					
676.321314	79012197.0					
676.410989	35410569.1					
676.445753	92452409.2					
676.535907	49281608.9					
676.572636	147917752.5					
676.660289	51212116.8					
676.695032	213841477.9					
676.785779	40760860.4					

676.821787	287855330.4
676.912797	21624964.5
676.945880	170107020.9
677.036868	19217923.2
677.071898	132467033.7
677.197315	59124758.5
677.319022	48462359.2
677.447704	37223692.8
677.572394	21789816.6
679.193883	28785367.4
679.318553	29384829.4
679.444238	29091965.1
679.569597	18810489.1
679.693585	31768745.0
679.819332	29049859.7
681.189870	16791443.8
681.313407	22896832.8
681.688943	19386241.3
681.938815	18842291.8
690.535961	43624701.0
690.636556	83275014.2
690.699427	21430985.5
690.736041	75731415.5
690.799510	17070488.5
690.836818	73494187.1
690.937792	89589300.8
691.037592	76356158.9
691.137367	47377160.7
691.235957	30343871.0
751.788701	17129400.1
752.590809	15950605.1
753.788413	54524397.9
753.931944	126047911.4
754.074721	165751402.2
754.217835	132704510.1
754.361130	71340888.0
754.504178	38134998.5
754.646501	30718001.5
754.789436	15828765.5
765.708789	22267989.4
765.821042	16547398.0
765.934030	23303124.7
767.217714	20525266.4
767.258496	99362653.6
767.327912	52466042.6
767.371602	357468581.6
767.397075	18152165.3
767.419807	24598081.7
767.439221	80232210.7
767.483055	749962300.4
767.508962	32131968.1
767.528633	38839398.8
767.549520	85380607.1
767.594361	1080569177.0
767.637124	49646696.2
767.660665	106296754.2
767.705341	1082796598.0
767.747291	52776343.2
767.771345	90095053.4
767.816579	960070601.5

767.856742	44495703.9					
767.883971	88660842.0					
767.927772	788764229.5					
767.966171	34527683.9					
767.995776	64621649.5					
768.039155	566678711.3					
768.076524	32487673.9					
768.150080	382110409.4					
768.186855	21528595.2					
768.220888	28612327.1					
768.261914	259293766.0					
768.298431	15835215.3					
768.322467	16705306.3					
768.331194	18402795.4					
768.372608	149889959.2					
768.484979	114175904.3					
768.708263	40484169.8					
768.797340	19704965.9					
768.819419	20489727.1					
768.940096	20750725.8					
769.703627	50393228.5					
769.815310	153630613.5					
769.879990	22789302.8					
769.926686	282690849.4					
769.950099	15827633.2					
769.992229	30032759.7					
770.017016	16821452.4					
770.037968	424580442.4					
770.059563	26214864.7	$C_{71}H_{90}N_{23}O_{38}P_7S_7$	770.060238	0.877	-3	w7 - B(T) - 2H
770.103788	33385802.1					
770.149176	440233127.6					
770.180263	16591603.4					
770.193013	16600459.8					
770.214809	29256824.8					
770.260575	426549810.8					
770.283284	22457631.3					
770.325708	24689915.3					
770.349309	17876809.8					
770.371681	408008645.0					
770.393095	24202076.2					
770.405740	17169564.7					
770.430634	19527805.9					
770.482989	303684770.0					
770.594096	224630913.7					
770.705359	156802280.2					
770.813931	78921762.7					
770.924730	55692369.1					
770.942873	130269623.9					
771.036929	38313377.2					
771.084510	196398584.4					
771.149822	15950313.8					
771.182905	19341915.4					
771.227435	227707500.9					
771.264195	17749815.7					
771.328237	18258530.5					
771.370525	254576192.6					
771.472611	22613322.5					
771.513531	222571391.2					
771.589656	77628791.4					
771.656074	159582165.0					

771.700673	147330760.2					
771.744728	20805394.8					
771.755524	21301228.7					
771.797329	104568613.1					
771.813374	192461369.1					
771.840420	17538519.3					
771.870804	21912765.8					
771.890060	17735157.1					
771.923109	230840423.3					
771.968257	17359032.3					
771.984785	23816533.4					
772.005905	17634835.2					
772.033960	236975603.9					
772.083538	73170961.9					
772.116412	16504288.6					
772.145104	213382488.2					
772.213344	26041772.9					
772.257137	171946766.4					
772.323129	24292717.9	$C_{96}H_{124}N_{31}O_{51}P_9S_9$	772.322409	-0.932	-4	d9 + 2H
772.368214	181968522.5					
772.416628	26187707.5					
772.436881	17349309.3					
772.479647	138475133.4					
772.548597	15881323.5					
772.592172	106110657.3					
772.662820	17524560.8					
772.675378	15893467.3					
772.703535	81782795.1					
772.721707	20686678.6					
772.733195	19000890.0					
772.761746	37202500.8					
772.780108	21233263.9					
772.816531	67114025.9					
772.837050	24173604.4					
772.849679	24036362.1					
772.860888	24061408.5					
772.886943	24686811.9					
772.913185	33035282.6					
772.931194	42205925.6					
772.944082	41352566.3					
772.953165	38780417.8					
772.967127	40673204.9					
772.975488	39502920.3					
773.001209	64043153.6					
773.013450	67348590.9					
773.033820	338419726.7					
773.080678	4334640920.0	$C_{172}H_{217}N_{56}O_{88}P_{15}S_{15}$	773.079889	1.021	-7	M - 7H
773.122035	143228451.8					
773.134550	162580579.6					
773.145046	154854387.3					
773.157826	190312316.0					
773.177031	834897457.7					
773.223861	10074010261.0					
773.255057	375664185.1					
773.268236	273777491.0					
773.278956	295359946.8					
773.290526	275122752.8					

773.366887	15856668303.0
773.386826	931262990.5
773.399614	436685264.9
773.417899	354074853.2
773.441335	301643741.4
773.463095	1374813013.0
773.510113	17444916642.0
773.551591	383723847.0
773.563388	453283181.2
773.587127	365551983.6
773.605710	1213611048.0
773.619280	620144913.0
773.652860	15607582125.0
773.684376	513266925.0
773.698429	317220376.5
773.708000	303809549.4
773.719870	294718778.0
773.747459	913136244.7
773.795807	11243680861.0
773.848347	258311655.9
773.858319	210695564.4
773.890313	667414017.7
773.904252	330831474.4
773.938756	7960275218.0
773.967999	281007198.1
773.982382	168641523.9
773.993178	193301441.3
774.004723	150944656.4
774.033608	443317673.4
774.050696	196477322.2
774.081452	5089737398.0
774.113218	148483708.9
774.133276	117202640.4
774.149289	67636068.7
774.176001	284896373.0
774.224531	3107999612.0
774.277340	77573069.4
774.289472	56150794.3
774.302350	49374040.4
774.319241	172704444.1
774.336788	72075043.1
774.367116	1889863539.0
774.399112	52905034.7
774.421701	33846975.0
774.434935	24955793.7
774.464062	94146099.7
774.510377	1053690591.0
774.563540	21348859.5
774.583682	39890352.0
774.605171	42829536.1
774.653161	602765818.3
774.686327	23021836.7
774.703082	26218119.6
774.746668	44253526.0
774.796146	312534314.6
774.823822	19209506.5

774.886091	16743757.2
774.939835	154624208.3
775.082930	124919379.9
775.104968	22484866.1
775.224358	47908280.4
775.249363	42033908.0
775.345035	24812042.5
775.364710	44293535.2
775.414407	33766669.3
775.507936	47870275.3
775.580764	22201703.7
775.650773	59494077.8
775.683261	28878201.3
775.747700	16240522.5
775.794144	60170795.9
775.851897	25224095.5
775.938176	55795808.2
776.081931	52227287.4
776.173374	61278723.7
776.198458	30330097.6
776.221086	763181671.2
776.253707	24669608.2
776.273830	35132560.0
776.284256	28174854.1
776.296644	31652893.4
776.316874	128582026.2
776.364297	1715108008.0
776.394608	68077512.8
776.408465	45689506.4
776.420107	44035321.2
776.430674	46447014.0
776.459565	185342447.5
776.507164	2448713372.0
776.559310	63354165.5
776.571419	56288525.7
776.583067	46967412.0
776.602267	186418839.9
776.616355	97724307.4
776.650015	2561250836.0
776.681098	88120679.1
776.703849	53881439.4
776.717754	48525776.1
776.744746	174225560.2
776.793065	2247368425.0
776.835395	43172039.2
776.846254	56242318.8
776.857967	45624050.5
776.870948	42471176.3
776.887500	132731027.9
776.904832	62160523.0
776.935716	1685634174.0
776.967550	50032312.8
776.987657	35862624.0
777.009675	26891128.6
777.030223	91954246.4
777.043129	44204536.2
777.078818	1156090529.0
777.108350	41398196.8
777.124149	21215022.1
777.132922	26427182.9

777.145144	21374786.9					
777.172898	58770226.1					
777.221693	717384032.2					
777.275010	17552765.9					
777.315722	32066784.5					
777.364339	397523312.9					
777.458580	17394664.9					
777.507511	207164678.8					
777.650338	106624908.5					
777.793601	54793831.3					
777.936525	33981801.5					
778.079709	31275593.0					
778.501752	183436533.6					
778.579742	26625210.0					
778.591975	27301723.6					
778.644881	373923185.1					
778.745850	66681019.6					
778.788034	480816821.1					
778.883586	37547448.0					
778.930920	526394417.8					
778.953624	23929056.2					
778.983537	17082831.0					
779.026015	32327647.2					
779.073715	437755261.4					
779.169126	23001843.8					
779.216293	294135353.8					
779.236879	35778409.8					
779.312352	16340064.1					
779.359344	218888488.9					
779.419078	18203144.4					
779.502341	165631735.0					
779.576213	15857164.7					
779.645521	123937370.4					
779.789226	93930025.4					
779.932579	65900060.4					
780.075893	47745062.2					
780.219252	36846721.3					
781.926606	16334942.8					
818.107523	19570031.6	$C_{56}H_{70}N_{14}O_{28}P_4S_4$	818.108589	1.303	-2	a5 - 2H, b5 - H2O
819.275617	17113993.2	$C_{130}H_{165}N_{38}O_{68}P_{12}S_{12}$	819.275150	-0.570	-5	a13 - B(G) - OCH2
819.474627	24544927.2	$C_{126}H_{162}N_{44}O_{66}P_{12}S_{12}$	819.476178	1.893	-5	w12 + 2H
823.337198	18992478.1	$C_{106}H_{132}N_{33}O_{54}P_9S_9$	823.335782	-1.720	-4	b10 - H2O, a10 - 2H
823.587193	21435512.6					
823.836964	16417339.9					
846.790968	16112235.2					
847.041412	22849771.6					
858.078070	20938921.2					
858.245256	26422512.1					
858.412626	24927683.3					
861.547175	15858404.2					
861.673564	17225979.7					
861.799307	22114962.4					
861.925084	16604723.6					
863.295211	50952106.3					
863.364804	17840397.9					
863.420738	69886690.7					
863.489259	28934006.5					

863.546419	122452002.6					
863.614566	38663979.9					
863.671535	143488494.6					
863.738590	34208294.9					
863.793287	154674530.9					
863.864536	31532378.9					
863.922918	151752254.3					
863.992750	22582877.2					
864.048708	141777562.4					
864.116886	20331907.1					
864.173645	163982367.0					
864.297991	105315361.1					
864.422716	50413201.0					
864.546540	31700461.7					
866.420306	37447829.4					
866.546060	43217974.7					
866.669855	39519090.1					
866.793902	24247019.4					
881.428923	27230849.9					
881.764032	37116876.6	$C_{86}H_{107}N_{26}O_{44}P_7S_7$	881.764150	0.134	-3	b8 - H2O, a8 - 2H
882.097217	28425847.0					
882.432957	19704412.5					
887.097359	36084433.9	$C_{56}H_{73}N_{18}O_{29}P_5S_5$	887.096848	-0.576	-2	w5
887.598445	16180782.7					
901.928236	21684067.3					
902.096213	65093486.1					
902.263695	99978697.4					
902.367849	23153881.9					
902.432081	108638662.8					
902.535011	22324207.3					
902.598612	102453879.3					
902.700301	28507464.3					
902.764430	135600532.4					
902.865082	18739315.5					
902.932167	105250922.8					
903.097389	119320691.1					
903.264784	74129212.0					
903.430814	64305415.2					
905.925285	17713797.1					
906.094511	39192167.6					
906.261629	27117157.4					
906.427004	31863699.9					
906.760884	18783521.5					
909.593124	23412234.1	$C_{116}H_{144}N_{38}O_{59}P_{10}S_{10}$	909.593201	0.085	-4	a11 - 2H, b11 - H2O
909.844591	19331341.4					
922.426315	32502857.3	$C_{86}H_{110}N_{30}O_{44}P_8S_8$	922.424684	-1.768	-3	w8
922.759491	35513859.4	$C_{86}H_{111}N_{30}O_{44}P_8S_8$	922.760626	1.230	-3	w8 + 1H
923.092337	26388830.2					
986.771621	22294843.5					
986.916104	26189782.6					
986.976153	17345642.4					
987.055052	72554223.0					
987.120042	19369423.3					
987.198655	80198894.4					
987.264402	20758398.7					
987.341002	85851847.9					
987.409105	18284303.3	$C_{90}H_{114}N_{30}O_{49}P_9S_9$	987.408586	-	-3	w9 - B(A) -

				0.526		CH3
987.483500	92245507.3					
987.625555	88094319.6					
987.769387	99113910.6					
987.912541	71220573.4					
988.051056	27068137.1					
988.198285	18729768.9					
990.194575	20789868.6					
990.338315	24212408.3					
1024.344266	26254518.9	$C_{126}H_{161}N_{44}O_{66}P_{12}S_{12}$	1024.345086	0.801	-4	w12 + 1H
1052.100360	18575587.5	$C_{65}H_{82}N_{23}O_{34}P_{6}S_{6}$	1052.099949	-0.391	-2	w6 - CH3
1052.502433	63754996.3					
1052.702137	62212497.5					
1052.902759	47153161.2					
1053.104112	24012500.2					
1053.304398	22885735.3					
1055.501855	15967392.9	$C_{167}H_{211}N_{51}O_{88}P_{15}S_{15}$	1055.502292	0.414	-5	M - 5H - 4H - dA
1055.700289	23732066.8					
1055.901335	21568506.8					
1075.609604	18965405.2	$C_{136}H_{170}N_{46}O_{69}P_{12}S_{12}$	1075.610415	0.754	-4	z13 - 2H, y13 - H2O
1075.861033	22088284.9					
1076.110711	34414699.3					
1076.361199	19712292.2					
1082.310413	84874754.0	$C_{172}H_{215}N_{56}O_{88}P_{15}S_{15}$	1082.311626	1.121	-5	M - 7H
1082.619180	25567295.2					
1082.711165	293698963.9					
1082.819700	29496334.1					
1082.911134	265927766.4					
1083.018430	28700199.2					
1083.112228	219096346.6					
1083.216827	21447401.0					
1083.315167	154579955.9					
1083.516072	126934842.1					
1083.718164	60674057.8					
1083.91537	42354647.1					

7						
1084.111634	29659549.7					
1084.311930	20921223.6					
1086.908482	22801339.6					
1087.106815	20289929.3					
1087.308702	24385286.1					
1087.510512	36003920.7					
1087.713612	29356786.5					
1087.912563	17318814.4					
1088.109686	17665523.0					
1136.135811	21014505.2	$C_{36}H_{46}N_{13}O_{18}P_3S_3$	1136.138600	2.455	-1	w3
1151.731023	29780909.2					
1151.896392	23466870.9					
1152.061990	52253253.1					
1152.227709	47493622.9					
1152.399086	37813837.5					
1152.563858	26845493.4					
1152.732351	18048895.3					
1155.556505	16941790.0					
1155.725942	22132995.9					
1354.141456	26749605.9					
1354.394086	28565442.8					
1354.641470	26472335.7					
1354.892961	24068795.8					
1359.886091	20624595.1					
1360.386404	16811857.2					
1382.473280	17253307.6	$C_{131}H_{170}N_{38}O_{70}P_{12}S_{12}$	1382.473087	-0.140	-3	b13 - B(G) + 2H
1382.672956	24183287.2					
			Abs mean error	1.080		
			Abs mean std dev	0.726		

Table S19 showing peak list, intensity, elemental composition, and possible assignments with mass errors (ppm) of danvatirsen using 2D-UVPD-MS of eight scans accumulated and no denoising of [M-8H]$^{8-}$ precursor ion (note, noise peaks were included to highlight the effect of denoising).

**Measured *mz***	**Intensity**	**Elemental Composition**	**Theoretical *mz***	**Error (ppm)**	**Charge**	**Assignment**
200.425558	6283539.9					
200.462841	5628581.4					
225.438893	10564576.9					
225.476471	8765118.5					
225.480426	20235333.8					
225.518172	7412878.0					
225.522299	21003522.4					
225.559906	9072240.0					
225.560642	11126083.0					
225.563882	24188506.1					
225.601777	5331210.5					
225.606030	29049693.2					
225.643932	6298006.9					
225.647212	14118482.3					
225.689457	10128796.4					
230.210639	6809572.8					
230.244376	6963468.3					
230.277589	6843291.7					
230.311181	6504221.7					
255.791807	9156258.4					
255.828731	13964739.8					
255.866139	20940972.7					
255.897366	5413032.3					
255.898092	5370569.2					
255.903066	22720304.8					
255.940279	23385758.8					
255.977210	13964772.8					
256.014432	10933694.0					
256.051422	6178323.8					
257.694434	9780754.6					
257.736907	6375802.8					
257.742072	20943680.9					
257.784429	6079372.6					
257.789770	24492470.3					
257.832056	7115408.1					
257.837401	26857685.5					
257.879661	6857935.1					
257.885291	22473433.8					
257.932675	15526231.5					
257.980557	7907829.8					
258.028325	6167080.5					
287.809935	11299606.3					
287.851290	17235836.1					
287.893096	20523761.3					
287.927728	6526855.8					
287.935076	20933946.7					
287.976753	16294621.4					
288.018365	11478101.3					
288.059933	8007292.1					
288.101617	5631749.5					
300.757844	10864341.1					
300.813637	14085221.6					

300.869185	13089753.2
300.924915	10196626.9
300.979957	7313086.5
301.035630	5361555.0
329.023385	8831407.2
329.071255	8974949.8
329.118858	8761442.4
329.166318	6757794.4
329.214359	6336887.0
338.148919	9996115.4
338.157618	86050786.4
338.166937	9357618.2
338.211984	23399455.1
338.220381	177494085.7
338.227194	12193973.4
338.229887	24982756.6
338.265311	5333752.6
338.274441	40837490.5
338.282972	238611139.4
338.288848	10798147.6
338.292424	29318420.6
338.328669	6391733.2
338.336519	16268964.8
338.345279	117302715.8
338.354877	12837458.9
338.399620	23633136.4
338.408128	131695057.9
338.417500	14667035.1
338.457906	5496055.0
338.462611	20814561.9
338.470569	163063350.6
338.480093	18049850.3
338.524765	16055415.2
338.533045	115202339.5
338.542252	12997218.8
338.587176	6452986.8
338.595476	49465456.7
338.605597	6696162.2
338.658021	21080000.7
338.720672	26919934.6
338.783535	19364295.7
338.844893	6019677.9
339.531560	10029719.0
339.594462	8439749.5
339.656739	14253734.1
339.719468	20814921.2
339.782124	18199472.0
339.843707	9182199.9
361.113056	5767694.0
383.685358	5352942.9
386.969608	7972403.8
431.769954	6341798.1
431.772778	6472232.0
450.963783	7264978.4
451.048442	10696618.4
451.382093	10076517.8
451.463471	5652740.5
493.530118	7702541.5
493.671806	6680951.7
609.309896	11550987.1

609.559242	9216438.5	$C_{76}H_{99}N_{25}O_{40}P_7S_7$	609.559960	1.178	-4	w7 + 1H
609.810216	7808747.2					
651.744645	9225509.9	$C_{66}H_{83}N_{16}O_{34}P_5S_5$	651.744365	-0.430	-3	a6 - 2H, b6 - H2O
657.564527	5743051.6					
657.595375	14006995.2					
657.814236	12567703.7					
657.938688	9221949.9					
658.096299	15484725.9	$C_{46}H_{57}N_{12}O_{22}P_3S_3$	658.096992	1.053	-2	a4 - 2H, b4 - H2O
658.192303	5537026.2					
659.438703	14657069.4					
659.564205	30084876.6					
659.688905	55570588.8					
659.781251	5890799.1					
659.814191	70581335.3					
659.939619	38193419.4					
660.064381	28268046.5					
660.190927	14735174.3					
660.317239	8068170.7					
662.438245	7356643.8					
662.560593	7694032.5					
673.699843	5609723.2					
673.820911	5656401.9					
673.826500	5576563.7					
673.945478	8492406.5					
674.071544	5450020.8					
674.089927	13901930.3	$C_{66}H_{84}N_{23}O_{32}P_5S_5$	674.090870	1.399	-3	y6
674.322631	57370597.1					
674.447981	125660984.7					
674.538726	10000074.7					
674.573342	222532358.1					
674.605544	6006663.7					
674.612821	6112957.9					
674.632176	6209525.6					
674.662890	12500210.5					
674.698158	243531417.2					
674.733746	6932359.1					
674.785529	14146881.1					
674.799090	10050048.1					
674.823208	236531015.8					
674.847057	8402166.4					
674.864656	6385417.0					
674.910776	13490518.5					
674.924748	6602609.0					
674.948454	196619717.5					
674.978997	5395263.7					
675.036969	11444742.0					
675.052909	6983351.2					
675.073115	136537023.5					
675.093744	7509758.8					
675.164049	7498853.9					
675.197688	97864741.5					
675.292823	6026325.4					
675.323435	60621294.0					
675.354785	5495965.5					
675.448433	40240666.9					
675.519134	5366127.6					
675.574090	19410015.3					
675.629496	6262650.1					
675.650416	6054593.2					

675.694376	12480324.5					
675.725929	7278682.7					
675.749610	6625046.3					
675.778449	5917186.7					
675.786051	6462040.0					
675.800513	6074806.0					
675.818630	9515328.9					
675.834668	5773140.8					
675.848449	5334317.9					
675.865393	6756735.1					
675.879516	6993720.3					
675.895760	10740931.1					
675.920726	7381275.0					
675.931816	10246538.4					
675.947918	9433826.0					
675.960880	7142499.9					
675.966952	7346501.8					
675.978491	7681420.6					
675.985431	8524541.4					
676.003043	9110422.0					
676.014338	7945611.4					
676.018911	8127329.6					
676.033839	9751354.4					
676.046927	9558692.4					
676.066302	12245651.8					
676.075894	11804414.3					
676.091773	10824859.9					
676.100839	11041833.2					
676.110154	11034627.3					
676.118670	12491458.5					
676.128009	11951069.6					
676.136391	12893707.7					
676.145538	13175443.8					
676.154305	14177985.9					
676.163771	12829779.9					
676.171431	14954588.3					
676.182275	16030382.2					
676.200704	20141186.0					
676.206440	21430644.5					
676.216953	21116880.7					
676.224473	21616397.5					
676.234203	27503131.8					
676.243079	26204517.4					
676.260339	44000645.4					
676.271087	57941805.8					
676.284175	268050987.1					
676.319520	3711390056.0	$C_{172}H_{217}N_{56}O_{88}P_{15}S_{15}$	676.318994	0.778	-8	M - 8H
676.344241	122742875.8					
676.357549	105487206.1					
676.365117	86936364.8					
676.377010	94011437.8					
676.384861	118879772.2					
676.394535	139547018.0					
676.409713	684000758.1					
676.445011	8954261760.0					
676.468475	313950146.2					
676.479138	228051872.8					
676.486103	222857652.6					
676.495905	175403236.3					

676.534596	1102491393.0
676.570276	14365938101.0
676.591562	511729878.4
676.603104	337366017.9
676.610838	359073763.6
676.620154	276488335.0
676.629861	261415017.3
676.638202	272524119.0
676.659512	1307530743.0
676.695436	17369331900.0
676.726597	353998253.5
676.735438	411528252.0
676.744338	308340133.9
676.754017	277295195.7
676.762467	295337928.6
676.784191	1205385738.0
676.820452	15686659085.0
676.860070	352688891.4
676.868327	260495042.8
676.878293	226144989.4
676.886845	246936378.1
676.897896	261729751.9
676.908856	943592409.5
676.922619	390400457.7
676.945450	11784306288.0
676.984796	246504257.2
677.002516	154335647.2
677.011314	170019123.1
677.021106	180120478.8
677.033987	663975679.1
677.046858	281469983.2
677.070426	8131876783.0
677.094075	233700629.6
677.109550	161020417.8
677.136851	125613628.1
677.144915	109970623.8
677.158954	455386749.1
677.171017	182575216.7
677.195459	5325518740.0
677.219209	172930662.1
677.235795	128509453.8
677.259638	60509101.3
677.269381	62286604.0
677.283995	272712479.9
677.295111	107390829.6
677.320473	3227589505.0
677.356426	58292797.9
677.361790	62648262.3
677.370346	42215107.6
677.389607	36529749.9
677.408965	154029837.7
677.422940	76003076.7
677.445662	1864724786.0
677.468206	63990151.6
677.480772	37626504.8
677.486251	38902577.7

677.495593	26004738.5					
677.506852	22908267.4					
677.523039	41707174.5					
677.536525	55150402.4					
677.544699	35184311.8					
677.570749	1003745871.0					
677.605419	21260108.2					
677.612073	25302091.9					
677.624320	29014274.3					
677.635141	31907027.9					
677.659242	27226723.7					
677.670504	21012722.9					
677.695758	508497537.5					
677.718442	22584648.3					
677.735855	22591814.6					
677.745202	11332449.7					
677.754869	11072978.6					
677.762433	8643321.4					
677.783332	24905138.0					
677.796048	10979116.1					
677.820557	272252350.5					
677.856681	37475759.9	$C_{106}H_{134}N_{33}O_{56}P_{10}S_{10}$	677.857433	1.109	-5	c10
677.888614	6827817.8					
677.907954	12854741.5					
677.925033	11307912.2					
677.945796	136138347.2					
678.024944	8189250.5					
678.039246	5422329.5					
678.054839	27250640.7					
678.071359	68118841.5					
678.129624	6654932.2					
678.196503	40599324.0					
678.231549	5562073.6					
678.257721	21615752.0					
678.320832	31434224.9					
678.333285	7152326.3					
678.358454	10186116.5					
678.444746	37698310.0					
678.461601	13570174.4					
678.569201	44943858.1					
678.654472	8337877.2					
678.693966	47380006.1					
678.818998	39465913.0					
678.855590	6666649.1					
678.944728	32964605.0					
678.991537	5332141.1					
679.031498	33642882.6					
679.067605	520890527.3					
679.088520	22492217.6					
679.109735	32226937.0					
679.116002	29502521.0					
679.136271	14914816.3					
679.156494	75441162.1					
679.192895	1118793539.0					
679.233661	30390334.8					
679.242705	23680310.6					
679.251812	21536236.4					
679.261058	21430605.5					
679.281579	102114727.1					

679.318018	1547762847.0
679.351317	29720430.8
679.358876	37047518.8
679.367689	29828405.8
679.377587	25847099.8
679.386301	25753625.3
679.406639	115915299.4
679.443106	1659677233.0
679.477101	29268546.0
679.484389	39939099.9
679.492994	28677524.5
679.502761	25941250.9
679.512088	25036948.7
679.531562	101846497.4
679.568150	1449540041.0
679.590059	52242164.3
679.603159	25603607.9
679.609088	34763070.4
679.620976	22627131.0
679.637290	20253757.2
679.656665	76933807.0
679.693044	1064179883.0
679.716094	35747581.5
679.733879	21749485.1
679.743717	15577824.7
679.754338	11811952.7
679.767069	12519700.3
679.781409	49481217.0
679.792518	20480297.1
679.818007	704919478.1
679.856595	13433396.6
679.872892	8148914.1
679.883324	8957084.0
679.892971	6486607.8
679.906147	29364545.9
679.919306	11166204.6
679.942997	406437641.8
679.982473	7722971.6
679.990064	6235559.3
680.000190	5841154.1
680.018541	6695175.3
680.031417	13249843.9
680.067981	218560746.9
680.123883	5471049.6
680.156362	7522427.0
680.176140	5980645.0
680.193094	121835296.2
680.209376	7794365.7
680.317918	60812421.1
680.443168	29945405.2
680.567734	12810985.8
680.695264	9470752.2
681.028774	5385960.5
681.062484	66401964.2
681.152437	9173855.1
681.187912	147773019.0
681.277232	12723385.8
681.312905	177802561.8
681.329928	7847968.1
681.403325	14875185.6

681.438155	159690318.1					
681.457022	8216506.4					
681.527707	8892202.5					
681.563403	144222874.0					
681.656144	9986318.8					
681.688346	106437895.1					
681.704897	7115469.0					
681.812794	73384331.3					
681.937548	52777004.1					
682.063506	40734526.0					
682.189023	27615023.3					
682.314498	15792067.4					
682.393667	11371508.7					
682.440207	10659373.8					
682.561340	15095652.1					
682.727340	11758538.7					
682.895621	8712312.6					
690.534144	13160516.5					
690.633217	22228587.0					
690.732166	26776322.9					
690.795078	5354786.4					
690.832070	52216398.1					
690.897282	6782820.2					
690.932856	35260976.4					
691.033791	23330967.5					
691.066093	6068146.5					
691.130161	15327810.7					
691.233932	10508235.0					
691.265306	6110901.3	$C_{106}H_{135}N_{40}O_{54}P_{10}S_{10}$	691.265336	0.043	-5	w10 + 1H
691.335777	6219438.8					
727.084806	6556542.8	$C_{46}H_{60}N_{16}O_{23}P_4S_4$	727.085251	0.612	-2	w4
727.873346	9172761.5					
735.638899	5803970.0					
735.783714	5832423.5					
743.079983	7376810.7					
743.330605	19127765.4	$C_{96}H_{119}N_{31}O_{48}P_8S_8$	743.329984	-0.835	-4	a9 - 2H, b9 - H2O
743.581269	17119895.9					
743.831480	12537551.3					
747.405274	6486820.0					
747.571234	12183073.9	$C_{51}H_{61}N_{10}O_{27}P_4S_4$	747.569771	-1.957	-2	a5 - B(dC) - NH3
747.740528	12216790.4					
756.234292	7082383.6					
766.754484	14895610.4	$C_{76}H_{95}N_{21}O_{39}P_6S_6$	766.754257	-0.296	-3	a7 - 2H, b7 - H2O
767.090639	23153471.6					
767.421282	18944377.2					
767.706835	8001143.3					
767.817845	6946879.4					
767.929163	7294697.3					
768.029243	7273050.9					
768.040284	5636748.6					
770.046890	14066709.5					
770.253840	5920132.2					
772.937332	17705766.6					
773.223449	5508254.4					
773.355865	13087360.6					
773.506481	10425160.8					

773.651522	9144505.4					
773.786355	5437728.2					
773.795281	6152600.4					
773.932829	5453761.2					
774.070193	8635289.7					
776.220645	7567384.4					
778.325844	6771042.9					
791.277064	6226487.4					
791.478748	28754417.4	$C_{126}H_{157}N_{40}O_{65}P_{11}S_{11}$	791.477745	-1.267	-5	a12 - 2H, b12 - H2O
791.679354	36897129.9					
792.079746	30438966.8					
792.281366	13491149.7					
809.077764	5841456.5					
809.246136	15890250.6					
809.412196	22815707.5					
809.579732	18316372.6					
809.746787	15766667.4					
809.914779	6771103.0					
810.077140	5501691.1					
812.748202	9202391.3	$C_{76}H_{98}N_{25}O_{40}P_7S_7$	812.746430	-2.180	-3	w7
819.074612	28569824.2	$C_{126}H_{160}N_{44}O_{66}P_{12}S_{12}$	819.073048	-1.909	-5	w12
819.220415	5550435.0					
819.274962	52290415.6	$C_{130}H_{165}N_{38}O_{68}P_{12}S_{12}$	819.275150	0.229	-5	a13 - B(G) - OCH2
819.422534	7184155.3					
819.475402	63904557.3	$C_{126}H_{162}N_{44}O_{66}P_{12}S_{12}$	819.476178	0.947	-5	w12 + 2H
819.675886	45571726.5					
819.875976	15412186.9					
820.077956	6560619.1					
823.833035	5957543.9					
823.841855	6588368.4					
830.623490	5342580.2					
840.091565	7931207.1					
840.599579	8270514.0	$C_{106}H_{135}N_{40}O_{52}P_9S_9$	840.599573	-0.007	-4	y10 + 2H
860.430327	6262493.1					
860.488880	68891577.9					
860.628366	6829201.6					
860.689167	80972890.8	$C_{136}H_{172}N_{46}O_{69}P_{12}S_{12}$	860.690007	0.976	-5	z13
860.829987	5879001.0					
861.089337	33395271.1					
861.288047	5519519.4					
876.751391	17358765.2					
876.918929	22152213.7					
877.085231	20495767.9					
877.254409	15467859.3					
879.419195	8041107.1					
879.584874	9788439.6	$C_{55}H_{70}N_{18}O_{29}P_5S_5$	879.585111	0.269	-2	w5 - CH3
879.745011	9173166.0					
879.755507	7048694.5					
880.254828	8824063.2					
881.074885	6058578.9	$C_{81}H_{104}N_{27}O_{43}P_8S_8$	881.074322	-0.639	-3	w8 - B(dC) + 1H
881.244901	6804232.5					
881.420759	9691362.0					

881.761144	15091422.2					
882.098561	13568270.9					
884.441931	7133534.2	$C_{86}H_{107}N_{30}O_{41}P_{7}S_{7}$	884.440000	-2.183	-3	y8 - H2O, z8 - 2H
884.775315	16006561.0					
885.105826	14242401.0					
887.097153	10786752.6	$C_{56}H_{73}N_{18}O_{29}P_{5}S_{5}$	887.096848	-0.344	-2	w5
899.591610	6861310.8					
899.601609	6400823.9					
899.769160	9292505.9					
901.694883	11287003.1					
901.733126	7728151.7					
901.759657	122586861.5					
901.792118	5810678.1					
901.862961	24235777.8					
901.899268	11855159.2					
901.926663	304344436.3					
901.958861	12865685.8					
901.999395	5380859.0					
902.029453	35997212.2					
902.092961	390071901.9	$C_{61}H_{77}N_{11}O_{33}P_{5}S_{5}$	902.091568	-1.544	-2	a6 - B(dA) - H2O
902.124964	19214649.9					
902.163434	5823013.7					
902.191293	28783024.9					
902.254325	263406086.6					
902.290769	14367056.8					
902.356759	30776969.3					
902.425455	219862424.5					
902.763958	13620831.1					
905.423023	8408437.1					
905.590447	24573790.6					
905.751326	14933553.4					
905.761813	14971105.5					
905.920954	9045567.5					
908.417144	5446119.8					
909.842076	5626306.0					
910.345379	6355825.2					
922.426427	10344271.6	$C_{86}H_{110}N_{30}O_{44}P_{8}S_{8}$	922.424684	-1.890	-3	w8
1000.359784	5485614.4	$C_{126}H_{160}N_{44}O_{64}P_{11}S_{11}$	1000.359214	-0.570	-4	y12 + 1H
1000.860095	5657582.0					
1015.981311	5457066.3					
1861.962043	6261963.2					
			Abs mean error	0.975		
			Abs mean std dev	0.678		

Table S20 showing peak list, intensity, elemental composition, and possible assignments with mass errors (ppm) of danvatirsen using 2D-UVPD-MS of eight scans accumulated and no denoising of [M-7H]7- precursor ion (note, noise peaks were included to highlight the effect of denoising).

Measured *mz*	Intensity	Elemental Composition	Theoretical *mz*	Error (ppm)	Charge	Assignment
257.880350	8656641.1					
257.885623	20971989.3					
319.013154	8923661.9	$C_{41}H_{52}N_{6}O_{25}P_{4}S_{4}$	319.013110	-0.138	-4	d4 - B(dA) - NH3
383.685016	16832640.0					
383.739782	18728249.9					
383.796832	30384586.8					
383.841274	11083282.4					
383.852797	48936345.1					
383.862631	9043590.6					
383.897121	14116631.4					
383.908148	75367695.8					
383.919355	12916776.0					
383.952884	12169487.7					
383.963527	78840005.0					
383.973789	9065037.8					
384.008457	10094076.3					
384.019236	44266219.5	$C_{61}H_{75}N_{14}O_{34}P_{6}S_{6}$	384.019125	-0.289	-5	c6 - B(T) - 4H
384.063865	7409762.4					
384.074677	24591650.1					
384.130362	13692752.2					
385.073607	10669592.1					
385.129184	14020356.3					
385.184926	12929334.0					
385.240197	8553282.4					
385.469652	7420791.5					
385.541151	11781058.6					
385.612926	10526686.3					
385.684355	11057577.9					
386.528174	9132113.4					
386.539149	66769154.5					
386.551490	7725611.1					
386.599373	19763618.2					
386.610730	75757626.7					
386.623652	9309687.3					
386.671337	23824795.5					
386.682138	178660383.7					
386.694210	19753646.6					
386.742571	35468281.5					
386.753658	208533367.8					
386.765844	25079126.3					
386.814330	24054091.6					
386.825046	196854423.7					
386.837024	22420827.5					
386.885373	14947688.9					
386.896552	94644305.7					
386.908615	11403562.6					
386.956956	7347168.5					
386.968370	32278126.9					
387.039433	56892624.4					
387.052119	6797378.6					

387.111064	51447387.3					
387.182483	28916837.5					
387.253943	11582320.6					
388.394884	7563984.9					
541.458285	9123272.1					
601.176134	9232661.6					
601.290031	8412681.5					
601.393193	7698256.9					
601.402285	24294772.3					
601.513525	16352078.2					
609.307192	6932970.0	$C_{76}H_{98}N_{25}O_{40}P_7S_7$	609.308004	1.333	-4	w7
657.593610	8644245.0					
676.322540	28892183.2					
676.415897	10264339.6					
676.451893	27299838.5					
676.540136	22448912.7					
676.573497	18500462.2					
676.579689	36090250.2					
676.669898	10885601.6					
676.691761	20682934.0					
676.700843	30917891.9					
676.817986	13903403.9					
676.828797	31893400.7					
676.942318	7514385.9					
676.949219	66563168.5					
677.072658	17401257.7					
677.198238	8486095.9					
679.197871	15938168.1					
679.695238	9614949.2					
679.821528	8549446.7					
690.530828	14405421.7					
690.540087	18368713.7					
690.642822	12683531.1					
690.706187	7351357.8					
690.734446	11356248.6					
690.845418	7916596.3					
691.041177	6929355.7					
691.235037	7281356.5					
727.579960	9328618.0					
751.646966	10703373.8					
751.788451	14384390.2					
751.931246	13300777.6					
752.481725	8039227.1					
753.147415	8592686.1					
753.788483	62697771.1					
753.884982	11313664.5					
753.931808	123156530.6					
753.951465	7616655.3					
754.028362	15691990.3					
754.074546	160005013.5					
754.094943	9178387.0					
754.171932	13365368.3					
754.217743	112702701.5					
754.312992	8050238.6					
754.361566	71742531.7					
754.505553	21920359.3					
754.643711	13223443.8					
755.499481	10063223.2					
755.645306	7051220.6					
756.075737	6768089.5					

765.479871	7915311.8					
765.593760	26529722.1	$C_{51}H_{67}N_{11}O_{28}P_4S_4$	765.592240	-1.985	-2	b5 - B(dC) + 2H
765.707975	48017151.3					
765.750766	7664544.7					
765.820125	75035043.0					
765.858337	7144588.2					
765.878587	7437041.6					
765.893996	7886030.1					
765.932480	84104951.2					
766.042858	72864709.7					
766.076834	7650789.0					
766.113954	9107772.8					
766.154971	61286850.0					
766.171674	7325082.6					
766.207469	7384172.0					
766.224912	9312248.7					
766.293351	7506562.5					
766.316365	7565533.6					
766.338951	7667617.1					
766.376809	32462177.8					
766.401838	7268344.6					
766.412633	8374147.2					
766.428863	9766740.7					
766.441624	6968114.6					
766.456620	7778964.5					
766.486035	25474524.5					
766.514688	7566258.4					
766.535818	7766018.4					
766.573457	7314461.5					
766.597112	20785553.7					
766.613570	9256426.5					
766.633357	8721036.8					
766.651472	7451550.8					
766.682829	9229190.5					
766.708067	21704972.7					
766.728389	10044179.1					
766.748883	24806535.7					
766.765365	14580612.4					
766.778108	10405591.5					
766.799014	10257429.4					
766.817063	16084595.8					
766.835335	10436934.1					
766.854973	11777340.1					
766.891642	11890937.1					
766.902723	11071573.8					
766.925417	16588885.4					
766.944466	14471870.9					
766.952496	12194357.4					
766.983077	13435280.1					
766.993559	12761704.2					
767.011613	13341510.0					
767.035791	16043397.6					
767.055857	14139508.9	$C_{71}H_{91}N_{20}O_{40}P_7S_7$	767.056382	0.684	-3	w7 - B(dA) - 2H
767.077162	18062552.7					
767.101050	16496119.6					
767.121941	16030045.5					
767.128311	15864570.3					
767.147197	21373936.6					
767.177479	22148388.9					

767.217496	42954910.5					
767.238327	17861632.5					
767.289453	38462160.6					
767.310868	52835980.9					
767.327883	127909438.6					
767.372752	1597296408.0					
767.439043	235828192.7					
767.484241	3090935596.0					
767.508317	149383553.8					
767.532707	144396165.5					
767.549787	296473799.4					
767.595588	4370355784.0					
767.625519	149263594.0					
767.659648	298993761.7					
767.706974	4850964395.0					
767.730388	207129070.3					
767.770250	271996942.9					
767.850051	143505734.6					
767.880756	224839656.8					
767.929403	3542675520.0					
767.958636	129578109.0					
767.992475	186022848.7					
768.018289	94368566.6					
768.040480	2832245025.0					
768.071865	92901051.1	$C_{96}H_{123}N_{31}O_{50}P_9S_9$	768.071724	-0.184	-4	c9 + 2H
768.103310	149855512.2					
768.151848	2067340777.0					
768.182878	82893894.1					
768.213425	99765770.7					
768.262999	1424182981.0					
768.289512	56278443.9					
768.326054	70412743.9					
768.374094	916677356.7					
768.405893	32778638.5					
768.426347	35068935.3					
768.436524	46338587.4					
768.457803	22635806.8					
768.485577	559048484.2					
768.516575	27280605.2					
768.549158	29974307.2					
768.624998	17971003.6					
768.634628	14896336.4					
768.648281	24943118.8					
768.666050	19164923.4					
768.682570	13137889.5					
768.708144	193710879.7					
768.738802	12785972.5					
768.757406	12139802.1					
768.768835	12389309.1					
768.788426	26466520.4					
768.819437	110746621.2					
768.848915	12021074.7					
768.858465	9803508.0					
768.882259	11061247.9					
768.907285	26696996.9					
768.930820	60587732.7					
768.958414	10887835.9					
768.968324	10157657.7					
768.980919	8866174.0					

768.995123	8229284.5					
769.016561	8734543.5					
769.041848	45116808.6					
769.058553	8728463.7					
769.071109	9294902.0	$C_{96}H_{119}N_{35}O_{47}P_9S_9$	769.070787	-0.419	-4	x9 - H2O
769.086864	12211432.0					
769.101482	8131397.9	$C_{51}H_{66}N_{15}O_{25}P_4S_4$	769.102104	0.809	-2	z5 - B(dC) + 1H
769.124220	7989118.8					
769.151848	29575117.8					
769.167239	21891305.7					
769.181344	9491329.4					
769.191818	8119120.9					
769.204073	8816240.0					
769.215088	11359133.5					
769.234984	9280790.9					
769.264392	43875160.5					
769.287272	18074480.2					
769.338137	7054019.1					
769.346219	7404672.5					
769.374580	52574687.8					
769.393855	7927584.3					
769.407359	16222515.2					
769.453743	7564177.4					
769.485565	48366837.8					
769.527056	11451068.1					
769.547817	11257007.0					
769.562719	7117618.0					
769.571908	8374497.1					
769.597019	45241287.9					
769.627516	9173164.0					
769.655658	17107589.2					
769.681551	15541833.5					
769.704335	187105987.2					
769.725500	19346545.2					
769.738418	14728458.2					
769.749624	13332675.4					
769.770521	45588900.5					
769.782527	23789055.9					
769.815529	541636313.5					
769.846845	25362972.4					
769.859518	26825125.1					
769.881401	78009414.2					
769.926818	974608374.8					
769.992104	107291552.3					
770.013999	49684271.0					
770.037974	1331252105.0					
770.058282	85193123.2					
770.102801	110840194.0					
770.149251	1396191044.0					
770.180208	57828318.0					
770.193443	47501622.5					
770.214863	117338342.8					
770.260629	1309875809.0					
770.302293	39715857.6					
770.324856	96276782.3					
770.348873	51570152.3					
770.371703	1128081442.0					
770.404547	43219341.6					
770.419272	35303783.2					

770.435235	67367726.9						
770.451696	38764653.6						
770.482898	803438372.1						
770.526041	36909979.2						
770.547548	54538251.3						
770.561151	32478625.3						
770.594173	558025937.8						
770.624109	27951578.7						
770.636740	22544922.9						
770.649713	31165401.4						
770.672721	23775605.7						
770.682854	27475728.6						
770.705225	354095055.4						
770.750295	26592208.1						
770.769358	28273946.7						
770.798553	185988162.5						
770.815517	188989500.9						
770.849621	15306968.9						
770.860813	14334605.2						
770.878213	17690321.8						
770.895659	40701806.3						
770.923572	110486174.8						
770.942559	369578644.7						
770.980862	13281431.0						
770.992618	13641908.0						
771.005781	12619569.7						
771.016878	15450638.3						
771.036280	77598193.4						
771.084472	543577456.9						
771.126825	14605080.2						
771.146477	38036198.8						
771.153089	37250889.0						
771.182080	43501095.1						
771.227253	601341133.5						
771.268057	37280967.7						
771.293474	13205237.7						
771.323280	43290161.8						
771.370465	604845356.8						
771.420146	20577993.7						
771.446862	14538050.3						
771.471151	54972146.8						
771.513448	471848614.6						
771.534543	36765999.0						
771.555100	20147096.6						
771.590729	158920597.5						
771.623515	29235249.4						
771.656242	323770068.0						
771.701134	289339255.8						
771.741391	37278930.5						
771.762474	47543238.9						
771.794372	172505956.2						
771.814035	363143112.7						
771.854246	24934745.4						
771.874715	44899390.5	$C_{121}H_{153}N_{41}O_{62}P_{11}S_{11}$	771.875151	0.565	-5		z12 - B(dC) + 1H
771.890974	35167982.4						
771.922801	413738086.2						
771.945276	103369240.5						
771.969420	26350636.9						
771.986182	45734690.5						

772.007189	28585851.9					
772.033844	410709678.9					
772.083802	132409968.2					
772.115506	26386058.0					
772.145060	358604514.4					
772.176907	20686760.0					
772.217356	42734384.0					
772.256758	281526249.3					
772.294405	12354534.0					
772.304804	11843472.3					
772.343037	14667350.4					
772.368155	268859200.8					
772.416124	43793734.1					
772.437265	19592187.9					
772.479681	187143024.5					
772.513743	20322808.5					
772.530947	13103111.9					
772.552263	15667978.9					
772.592304	137511343.3					
772.620517	18755996.0					
772.639770	19902292.0					
772.664353	21365105.6					
772.675172	20188500.3					
772.704247	102622980.2					
772.733184	25871315.3					
772.743529	20072782.0					
772.762363	49731025.5					
772.780215	26230919.9					
772.817413	77696203.0					
772.836795	31069361.6					
772.861151	32365841.6					
772.912276	42439988.7					
772.931339	50592212.8					
772.953612	54002664.9					
772.965694	50362383.2					
772.978547	49846068.9					
772.989973	58611176.2					
773.001030	69729578.9					
773.013031	80911190.0					
773.034221	390075669.8					
773.080642	4975486992.0	$C_{172}H_{217}N_{56}O_{88}P_{15}S_{15}$	773.079889	0.974	-7	M - 7H
773.122564	150767221.6					
773.134155	189543006.0					
773.145066	168246701.5					
773.157619	211224159.4					
773.177329	855352471.0					
773.223805	11105426166.0					
773.254633	413886210.5					
773.269161	282915467.9					
773.290652	284552792.2					
773.304564	312332579.1					
773.366876	16256423880.0					
773.386526	944988484.4					
773.399092	453417160.9					
773.417888	377346238.4					
773.440928	284963752.8					
773.462873	1250062511.0					

773.510121	16627272261.0
773.552492	324369401.6
773.562879	443121561.9
773.586902	339914321.9
773.605513	1071853333.0
773.619193	557531304.9
773.652864	13986650747.0
773.684067	477401894.3
773.706275	292790257.1
773.720011	263930804.5
773.747270	733837733.1
773.774632	369381294.5
773.795751	9375136039.0
773.848252	241538479.7
773.857775	175167989.9
773.890171	509646171.9
773.904635	270295985.3
773.938692	6401568808.0
773.967940	234336954.5
773.984210	122819907.5
773.992914	167931353.2
774.004552	121169710.8
774.019021	122595183.8
774.032988	301097887.9
774.051144	147128961.0
774.081459	3836077959.0
774.113338	120183719.7
774.134090	93552624.3
774.149710	54407166.6
774.175963	184915621.0
774.224548	2262759064.0
774.277749	56050367.2
774.288962	37698935.5
774.302193	33669672.0
774.319405	104125840.0
774.336227	46923187.9
774.367214	1289311257.0
774.399117	33307293.9
774.422057	19386904.9
774.434743	14766889.7
774.464072	47963348.0
774.510482	672410769.3
774.563201	12053964.3
774.587051	26216891.6
774.601942	22117511.9
774.653393	362586270.4
774.686382	14115030.6
774.703064	22577595.9
774.749732	23379708.9
774.768033	10701516.1
774.796191	167803601.6
774.823862	13583543.2
774.836049	10402153.8
774.848274	10048905.7
774.862598	9344453.2
774.889038	9954087.6
774.940510	79237403.9
774.968986	10299451.2

774.984648	10408719.4
775.006924	9601785.8
775.021369	9005660.2
775.059647	10860000.8
775.083449	58073679.1
775.105910	15282028.8
775.134867	10849784.3
775.163712	8751473.8
775.174895	11318450.7
775.193244	11264301.8
775.221109	22288754.9
775.244841	22228760.3
775.280949	7518977.1
775.303663	8775368.9
775.326808	7550637.4
775.343748	12714350.4
775.362564	20369573.6
775.395934	7111889.3
775.413052	17178275.1
775.424058	8772993.3
775.453849	7365693.4
775.483707	7755999.2
775.506411	21080625.6
775.530254	6781839.2
775.549816	6902517.1
775.579543	10666388.3
775.598227	9508745.4
775.649739	24652995.9
775.682853	14352505.2
775.710158	10045459.7
775.747457	8211004.5
775.793971	22362215.7
775.823301	8411096.3
775.851717	13426021.4
775.892760	7406569.2
775.938757	19937378.6
776.021460	6854534.2
776.047616	7375277.4
776.083881	19860086.8
776.173245	16015389.8
776.198614	11676636.9
776.221243	285498683.1
776.242662	17962018.0
776.252915	10720001.8
776.274178	15848613.2
776.298651	12438711.0
776.317414	29769921.1
776.329469	24173282.0
776.364388	594702781.1
776.394350	23806780.8
776.419178	20080487.6
776.430346	17988885.7
776.445361	19089571.8
776.460303	52060700.0
776.486022	27492300.3
776.507269	731722854.6
776.530378	37252161.2
776.540367	25177257.1
776.560343	25859645.0
776.602690	57710222.2

776.649971	725095706.5					
776.682049	27669117.2					
776.704485	21062526.6					
776.717225	14439211.0					
776.746020	47988040.5					
776.792999	607024222.5					
776.846682	18173293.9					
776.856918	11410168.4					
776.872161	12364767.1					
776.887290	32131619.5					
776.935576	440814788.8					
776.970355	14618314.8					
776.990070	9977642.7					
777.031452	20497863.1					
777.079105	291044595.9					
777.115242	6968402.4					
777.131402	7190374.8					
777.172674	13044760.4					
777.221839	183967954.8					
777.244387	7580327.7					
777.365078	79690634.3					
777.507896	37454301.7					
777.648471	17951338.7					
777.791253	6818947.9					
778.076083	9651437.6	$C_{96}H_{123}N_{35}O_{49}P_9S_9$	778.076070	-0.017	-4	w9 + 1H
778.498714	24500190.8					
778.597585	8298690.2					
778.639226	57729507.5					
778.646012	54724759.7					
778.785285	85269439.2					
778.887232	13188379.1					
778.927924	79382804.2					
779.028151	8524411.3					
779.071565	79802423.2					
779.214297	79367754.6					
779.242453	10950813.0					
779.357798	53357890.6					
779.500683	40039012.9					
779.643431	22082694.8					
779.786491	17233488.6					
818.112383	6794372.7					
819.073182	13340338.9	$C_{126}H_{160}N_{44}O_{66}P_{12}S_{12}$	819.073048	-0.164	-5	w12
823.338965	15941860.2					
823.586277	12841415.6					
823.838104	11721384.7	$C_{106}H_{134}N_{33}O_{54}P_9S_9$	823.839695	1.931	-4	a10
838.037967	17118344.9					
838.160431	13680471.0					
838.285630	14883931.8					
846.797400	6892070.3					
847.043716	8538234.6					
855.485996	7527473.0					
857.913434	9740521.8					
858.079095	22707370.8					
858.245104	24569875.9					
858.411654	24419174.5					
858.582185	11795701.8					
863.423365	9257425.5					
863.502943	9004017.1					

863.540613	30456456.1					
863.622009	16230759.7					
863.669581	28428442.4					
863.690016	6954755.0					
863.742958	24896851.4					
863.794101	11049619.2					
863.812968	10541888.2					
863.873531	15805313.7					
863.911102	10999936.5					
863.930959	59169357.9					
863.999201	9157179.8					
864.044489	28743633.6					
864.060318	34526395.1					
864.190599	18432005.4					
881.429354	30731512.7					
881.763585	36108378.6	$C_{86}H_{107}N_{26}O_{44}P_7S_7$	881.764150	0.641	-3	b8 - H2O, a8 - 2H
882.095115	28213411.9					
882.432794	17508216.6					
887.097147	38087141.8	$C_{56}H_{73}N_{18}O_{29}P_5S_5$	887.096848	-0.337	-2	w5
887.598473	20564042.2					
888.095407	15379331.4					
892.028580	7138695.0					
897.281582	9067102.2					
897.490715	8594083.1					
901.928224	12664794.7					
902.108499	9956741.9					
902.273135	13098591.9					
902.446854	9072733.1					
903.041916	6816826.7					
903.112826	17462435.3					
908.531432	14333233.3					
908.701856	17335175.0					
908.869981	13181447.1					
909.036925	10337608.1					
909.341151	11492177.5					
909.600032	10370016.7					
909.847562	13138697.4					
922.425899	32021112.2	$C_{86}H_{110}N_{30}O_{44}P_8S_8$	922.424684	-1.317	-3	w8
923.090975	24169173.5					
923.427959	14297625.2					
950.991004	6843093.9					
958.041584	9192465.5					
967.470197	9175102.6					
967.613890	12348946.2					
967.757693	10202367.7					
967.900938	8335023.4					
979.117683	7700500.8					
984.191665	12355787.1					
984.335479	15020440.6					
984.481228	15450198.8					
984.620918	9216482.2					
984.906446	8186525.1					
986.478745	7515548.8					
986.621437	30379061.6					
986.765452	50397702.1					
986.891349	14630271.3					
986.908574	37803636.5					
986.990502	7617235.7					

987.041067	25038811.2					
987.057533	23155637.9					
987.133593	9249860.6					
987.181471	9553756.6					
987.208915	14909817.2					
987.333111	15988680.1					
987.350783	17650542.9					
987.644083	20778677.0					
987.767679	27360600.0					
987.919835	9001970.1					
989.604414	7918685.6					
989.849791	11652214.7					
989.902275	16627351.2					
990.045534	6784933.6	$C_{31}H_{34}N_{10}O_{16}P_3S_3$	990.045648	0.115	-1	x3 - B(dC) - 4H
990.101709	9883677.7	$C_{126}H_{159}N_{40}O_{65}P_{11}S_{11}$	990.102912	1.215	-4	a12
990.332223	15735595.8					
1024.093695	17325694.8	$C_{126}H_{160}N_{44}O_{66}P_{12}S_{12}$	1024.093130	-0.552	-4	w12
1024.593865	23680214.9					
1024.843733	15654884.1					
1037.429469	8761831.7					
1037.765637	10246394.0					
1038.100638	8877070.9	$C_{131}H_{166}N_{41}O_{68}P_{12}S_{12}$	1038.100019	-0.596	-4	a13 - B(T)
1052.100393	20312183.6	$C_{65}H_{82}N_{23}O_{34}P_6S_6$	1052.099949	-0.422	-2	w6 - CH3
1052.501998	65765968.9					
1052.701907	57797811.6					
1052.903160	41694668.8					
1053.104488	21134328.6					
1053.303443	15914960.1					
1055.701173	21847097.9					
1055.901330	20703807.7					
1057.701470	8922578.9					
1057.900466	9687990.8					
1059.610780	8378460.2	$C_{66}H_{85}N_{23}O_{34}P_6S_6$	1059.611686	0.855	-2	w6
1075.610626	18157275.7	$C_{136}H_{170}N_{46}O_{69}P_{12}S_{12}$	1075.610415	-0.196	-4	z13 - 2H, y13 - H2O
1075.860596	21566338.9					
1076.109526	31789891.9					
1076.362494	19918459.5					
1076.60974	11074148.9					

4						
1077.115052	9716309.0					
1079.720183	9631687.1					
1080.110642	8700829.1	$C_{136}H_{172}N_{46}O_{70}P_{12}S_{12}$	1080.113056	2.235	-4	y13
1082.216992	8519980.8					
1082.310427	91427515.1	$C_{172}H_{215}N_{56}O_{88}P_{15}S_{15}$	1082.311626	1.108	-5	M - 7H
1082.420000	17807981.2					
1082.619347	27936228.5					
1082.710927	290822154.8					
1082.775347	10247466.8					
1082.820926	23952903.1					
1082.870424	12363494.5					
1082.911213	262512049.7					
1082.959359	9698080.3					
1083.016805	17718902.3					
1083.113297	217877851.8					
1083.206546	10912824.6					
1083.302553	24410733.2					
1083.331024	33347164.2					
1087.508094	9032103.1					
1087.525154	7067506.1					
1136.129913	21818470.2					
1137.140801	7075498.9					
1150.632085	11715550.0					
1151.135316	10267921.4					
1151.638871	8612569.8					
1151.898761	9908946.0					
1152.053543	15065953.7					
1382.686911	18903335.0					
			Abs mean error	0.788		
			Abs mean	0.677		

std dev

Supporting References

1. L. Chiron, M. A. v. Agthoven, B. Kieffer, C. Rolando and M.-A. Delsuc, *PNAS*, 2014, **111**, 1385-1390.
2. L. Chiron, M.-A. Coutouly, J.-P. Starck, C. Rolando and M.-A. Delsuc, *arXiv preprint arXiv:1608.06777*, 2016, 1-13.
